# Penicillin Derivatives Inhibit the SARS-CoV-2 Main Protease by Reaction with Its Nucleophilic Cysteine

**DOI:** 10.1021/acs.jmedchem.1c02214

**Published:** 2022-05-12

**Authors:** Tika R. Malla, Lennart Brewitz, Dorian-Gabriel Muntean, Hiba Aslam, C. David Owen, Eidarus Salah, Anthony Tumber, Petra Lukacik, Claire Strain-Damerell, Halina Mikolajek, Martin A. Walsh, Christopher J. Schofield

**Affiliations:** † Chemistry Research Laboratory, Department of Chemistry and the Ineos Oxford Institute for Antimicrobial Research, University of Oxford, 12 Mansfield Road, OX1 3TA Oxford, United Kingdom; ‡ Diamond Light Source Ltd., Harwell Science and Innovation Campus, OX11 0DE Didcot, United Kingdom; § Research Complex at Harwell, Harwell Science and Innovation Campus, OX11 0FA Didcot, United Kingdom

## Abstract

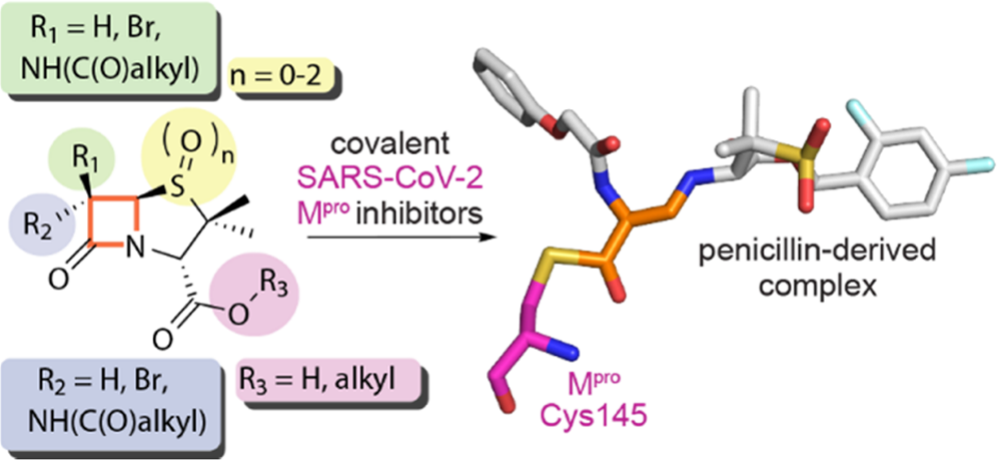

The SARS-CoV-2 main protease (M^pro^) is a medicinal chemistry target for COVID-19 treatment. Given the clinical efficacy of β-lactams as inhibitors of bacterial nucleophilic enzymes, they are of interest as inhibitors of viral nucleophilic serine and cysteine proteases. We describe the synthesis of penicillin derivatives which are potent M^pro^ inhibitors and investigate their mechanism of inhibition using mass spectrometric and crystallographic analyses. The results suggest that β-lactams have considerable potential as M^pro^ inhibitors via a mechanism involving reaction with the nucleophilic cysteine to form a stable acyl–enzyme complex as shown by crystallographic analysis. The results highlight the potential for inhibition of viral proteases employing nucleophilic catalysis by β-lactams and related acylating agents.

## Introduction

The inhibition of proteases that hydrolyze viral polyproteins to give functional proteins is a validated mechanism for antiviral chemotherapy, as exemplified by pioneering work on human immunodeficiency virus (HIV) protease and, more recently, hepatitis C virus (HCV) protease inhibitors.^1^ Thus, both the severe acute respiratory disease coronavirus-2 (SARS-CoV-2)^2^ main protease (M^pro^ or 3C-like protease, 3CL^pro^) and the papain-like protease (PL^pro^) are targets for the treatment and, possibly, prevention of coronavirus disease 2019 (COVID-19).^3−8^ M^pro^ is a particularly attractive drug target because (i) M^pro^ is vital in the SARS-CoV-2 life cycle, (ii) M^pro^ is tractable from a small-molecule inhibition perspective as a nucleophilic cysteine protease, and (iii) the structure and substrate selectivities of M^pro^ are different from human proteases,^9,10^ suggesting clinically useful selective M^pro^ inhibition should be possible.

**Figure 1:**
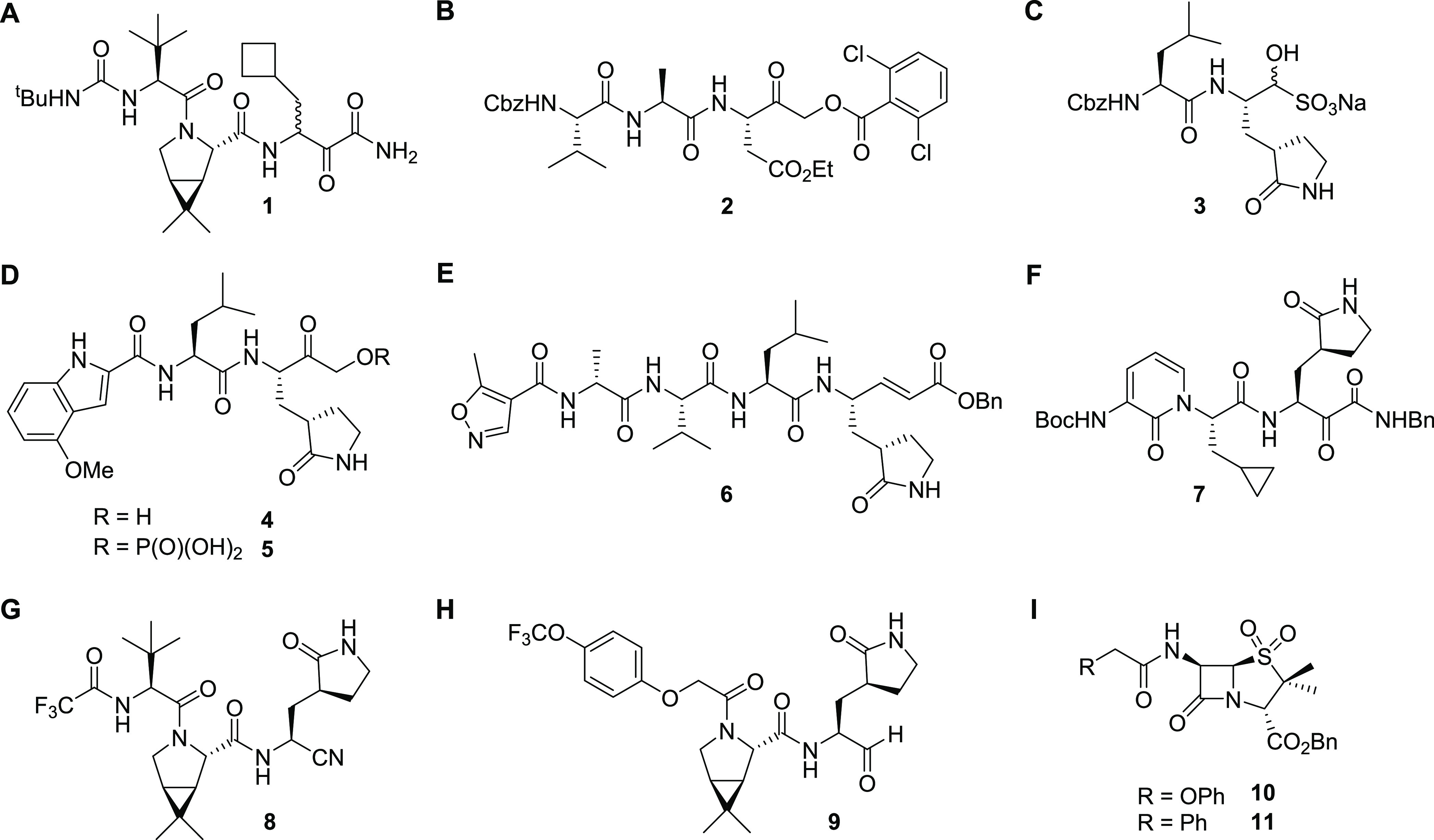
Examples of reported SARS-CoV-2 M^pro^ small-molecule inhibitors. (A) Boceprevir (**1**);^11,16^ (B) SDZ-224015 (**2**);^22^ (C) GC-376 (**3**);^11,16,21^ (D) PF-00835231 (**4**) and its prodrug PF-07304814 (**5**);^23,24^ (E) N3 (**6**);^9^ (F) α-ketoamide **7**;^10^ (G) PF-07321332 (**8**, nirmatrelvir);^25^ (H) MI-09 (**9**);^26^ and (I) penicillin V and G sulfone benzyl esters **10** and **11**.^15^

To enable the identification of small-molecule M^pro^ inhibitors for development as human therapeutics, high-throughput *in vitro* inhibition assays using recombinant viral M^pro^ have been developed.^4,9−12^ Most reported M^pro^ inhibition assays employ fluorescence-based methods, though label-free assays, which directly monitor product formation/substrate depletion using mass spectrometry (MS) and SARS-CoV-2 polyprotein peptide fragments, have been reported.^13−15^ The availability of efficient high-throughput M^pro^ inhibition assays and libraries of bioactive and safety-assessed small molecules has enabled the identification of multiple lead M^pro^ inhibitors, such as boceprevir (**1**),^11,16^ an HCV serine protease inhibitor,^17,18^ SDZ-224015 (**2**),^19^ an investigational caspase-1 inhibitor,^20^ and GC-376 (**3**),^11,16,21^ for (partially) selective inhibition of M^pro^ (Figure 1A–C).

To date, drug repurposing efforts have not yielded safe and efficient M^pro^ inhibitors for approved human clinical use. Thus, *de novo* M^pro^ inhibitor development programs have been initiated based on the structural information gained from the identified lead structures in the SARS-CoV-2 M^pro^ screening campaigns as well as from structure–activity relationship (SAR) studies with reported SARS-CoV and MERS-CoV M^pro^ inhibitors.^5,27−30^ Compounds arising from such efforts include PF-00835231 (**4**) and PF-07304814 (**5**),^23,24^ N3 (**6**),^9,31^ and the α-ketoamide **7**, which are all potent SARS-CoV-2 M^pro^ inhibitors displaying high *in vitro* and *in vivo* potency (Figure 1D–F). Novel SARS-CoV-2 M^pro^ inhibitors include compounds PF-07321332 (**8**, nirmatrelvir),^25^ which is in clinical use,^32^ and MI-09 (**9**)^26^ and structurally related molecules (Figure 1G,H).^6,7,33−37^

Most M^pro^ inhibitors work by covalent modification, in part, likely because of well-precedented mechanisms for inhibiting proteases and related enzymes by covalent reaction with nucleophilic serine or cysteine residues,^1^ although noncovalent M^pro^ inhibitors have also been reported.^38−41^ Electrophiles employed in covalently reacting M^pro^ inhibitors include, for example, nitrile, α-ketoamide, α-acyloxymethylketone, aldehyde, and Michael acceptor, amongst other functional groups (Figure 1).^42−49^ By contrast with the extensive work on alkylating agents such as SDZ-224015 (Figure 1B), work on acylating agents, such as β-lactams, which generally have good safety profiles as antibacterials, has been limited. Because of their demonstrated efficacy and safety records,^50,51^ we are particularly interested in optimizing the potential of β-lactams and related acylating agents as inhibitors of nucleophilic cysteine enzymes, in particular, M^pro^.

Recently, we reported a solid-phase extraction coupled to MS (SPE-MS) M^pro^ assay, which enabled the identification of a certain penicillin V derivative, i.e., **10**, that inhibits M^pro^ by reaction with the active site cysteine residue; by contrast, the corresponding penicillin G derivative **11** was inactive (Figure 1I).^15^ Here, we report SAR studies with penicillin derivatives, leading to the identification of efficient M^pro^ inhibitors with a penicillin scaffold; their mechanism of inhibition was investigated using MS and crystallography. The results highlight the potential of β-lactams for use as M^pro^ inhibitors working by acylation of the nucleophilic cysteine.

## Results

### Penicillin Stereochemistry Affects M^pro^ Inhibition

To enable SAR studies, an initial set of penicillin V derivatives was synthesized based on the identified penicillin V sulfone benzyl ester M^pro^ inhibitor (**10**; Figure 1I).^15^ Half-maximum inhibitory concentrations (IC_50_ values) were determined using the reported SPE-MS inhibition assay, monitoring M^pro^-catalyzed hydrolysis of an 11mer substrate peptide (TSAVLQ/SGFRK-NH_2_, “/” indicates the M^pro^ cleavage site), the sequence of which is based on the N-terminal self-cleavage site of M^pro^. However, some of the initially investigated penicillin V derivatives appeared to suppress product peptide ionization at high inhibitor concentrations, perturbing the reliability of the inhibition results. Therefore, the M^pro^ inhibition assays were performed using an extended 37mer peptide substrate based on the same M^pro^ self-cleavage site (ALNDFSNSGSDVLYQPPQTSITSAVLQ/SGFRKMAFPS-NH_2_),^15^ which was less susceptible to penicillin inhibitor-induced ion suppression. Substituting the 11mer peptide with the 37mer peptide in the SPE-MS M^pro^ inhibition assays did not affect the IC_50_ values of reported selected M^pro^ inhibitors (Supporting Information Table S1); thus, the 37mer substrate was used for subsequent IC_50_ determinations. The observed high *Z*-factors (>0.5 for each inhibition plate) indicate excellent SPE-MS assay quality using the 37mer substrate peptide (Supporting Information Figure S1).

The modified SPE-MS M^pro^ inhibition assay was used to investigate the influence of structural features of the penicillin V sulfone benzyl ester (**10**) on potency. Unlike **10**, neither commercially sourced penicillin V (**12**) nor its benzyl ester (**13**)^52^ inhibited M^pro^ efficiently, in agreement with previous results using the 11mer M^pro^ substrate^15^ (Table 1, entries 1 and 2). Stereoselective oxidation of **13** with *meta*-chloroperbenzoic acid (mCPBA) afforded the reported penicillin V (*S*)-sulfoxide benzyl ester (**14**),^52^ which is a less efficient inhibitor than the corresponding sulfone **10** (IC_50_ ∼ 22.9 μM, Table 1, entry 3), highlighting the importance of an additional *pro-R*-sulfone oxygen of **10** for M^pro^ inhibition.

**Table 1:**
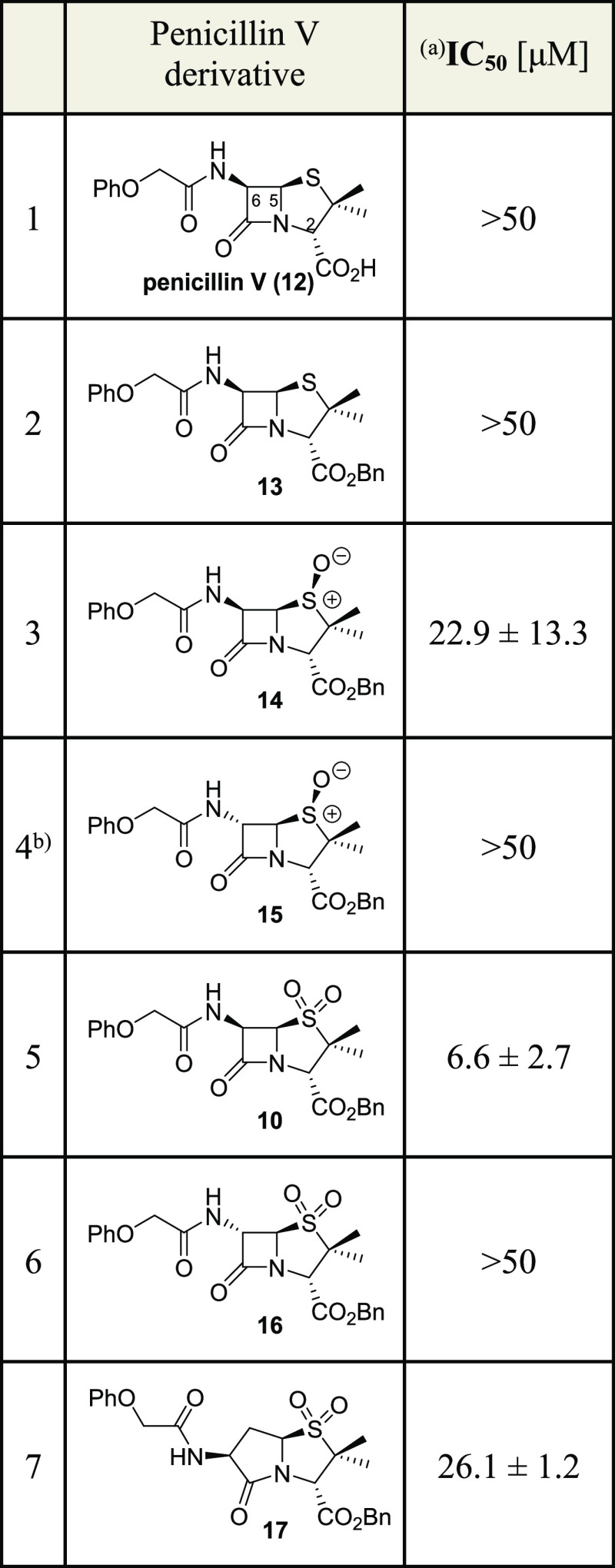
Inhibition of SARS-CoV-2 M^pro^ by Penicillin V Derivatives

a M^pro^ inhibition assays were performed using SPE-MS as described in the Experimental Section employing SARS-CoV-2 M^pro^ (0.15 μM) and a substrate (2.0 μM). Results are means of at least two independent runs, each composed of technical duplicates (*n* ≥ 2; mean ± standard deviation, SD). Representative dose–response curves are shown in Supporting Information Figure S4.

b Contains minor amounts of a decomposition product, as reported.^53^ Bn: −CH_2_Ph.

Next, the effect of the stereochemistry at the C6 stereocenter on inhibition was investigated by inversion of the (*R*)-configuration of **10** using a reported protocol.^53^ The resultant (6*S*)-penicillin V (*S*)-sulfoxide benzyl ester **15**(53) showed reduced M^pro^ inhibition compared with **10** (IC_50_ > 50 μM, Table 1, entry 4). The corresponding (6*S*)-penicillin V sulfone benzyl ester (**16**), which was obtained from **15** using KMnO_4_ as an oxidant, was also less efficient in inhibiting M^pro^ than the (6*R*)-isomer **10** (IC_50_ > 50 μM, Table 1, entry 6). Thus, the (6*R*)-configuration at the penicillin V C6 stereocenter appears to be preferred for efficient M^pro^ inhibition.

The importance of the β-lactam ring for efficient M^pro^ inhibition was investigated by preparing the corresponding γ-lactam **17**, which was synthesized using a modified literature procedure (Supporting Information Figure S2). The potency of **17** was reduced compared to the β-lactam **10** (IC_50_ ∼ 26.1 μM, Table 1, entry 7) but was not ablated. This observation may in part reflect the enhanced and/or less reversible reaction of β-lactams with nucleophiles compared to γ-lactams.^54,55^

### Penicillin Ester Group Fine-Tunes Inhibitor Potency

**Scheme 1:**
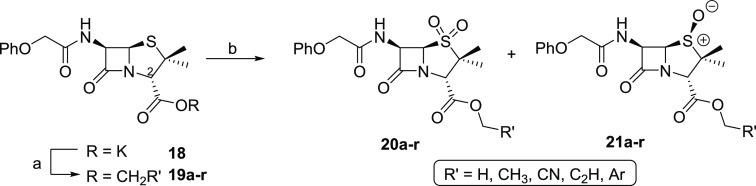
Synthesis of Penicillin V Ester Derivatives Reagents and conditions: (a) alkylhalide (1.2 equiv), DMF, rt, 59–93%; (b) mCPBA, CH_2_Cl_2_, 0 °C to rt, 89–94%. Note that the sulfoxide stereochemistry was tentatively assigned the (*S*)-configuration based on reported mCPBA-mediated penicillin ester oxidations to sulfoxides.^52,56,57^

Having identified important structural features of penicillin V sulfone benzyl ester (**10**) for efficient M^pro^ inhibition, the impact of its C2 ester group on potency was investigated. To obtain a set of varied penicillin V sulfone esters, the commercially sourced penicillin V potassium salt (**18**) was initially reacted with different alkylhalides (Scheme 1). The resultant penicillin V esters **19a–r** were oxidized using mCPBA to afford a chromatographically separable mixture of both the sulfones **20a–r** and (*S*)-sulfoxides **21a–r**.

The penicillin V sulfone esters **20a–r** were investigated for M^pro^ inhibition using the SPE-MS assay with the 37mer peptide substrate (Table 2). The preferred phenyl ring substitution pattern of the benzyl ester group for M^pro^ inhibition was investigated by fluorine atom substitution. The results reveal that a single fluorine substituent in the *ortho*-, *meta*-, or *para*-position does not substantially alter the potency (Table 2, entries 1–3). However, the presence of two fluorine *meta*-substituents, as in **20d**, appears to reduce the potency (IC_50_ ∼ 16.9 μM, Table 2, entry 4) while the corresponding isomer **20e**, which bears a fluorine substituent at both the *ortho*- and *para*-positions, is slightly more potent than the benzyl ester derivative **10** (IC_50_ ∼ 3.6 μM, Table 2, entry 5). This trend was clearer when comparing the trifluorinated benzyl ester derivatives **20f** and **20g** (Table 2, entries 6 and 7). The derivative **20f** with two fluorine substituents as the *meta*-positions and one at the *para*-position is less potent than the originally identified benzyl ester **10** (IC_50_ > 50 μM), while the derivative **20g** with two fluorine substituents as the *ortho*-positions and one at the *para*-position is more potent (IC_50_ ∼ 1.6 μM). It is unclear whether these observations reflect alterations in (hydrophobic) interactions of the fluorinated ester groups with M^pro^ and/or (less likely) enhanced β-lactam reactivity due to remote electronic effects.

**Table 2:**
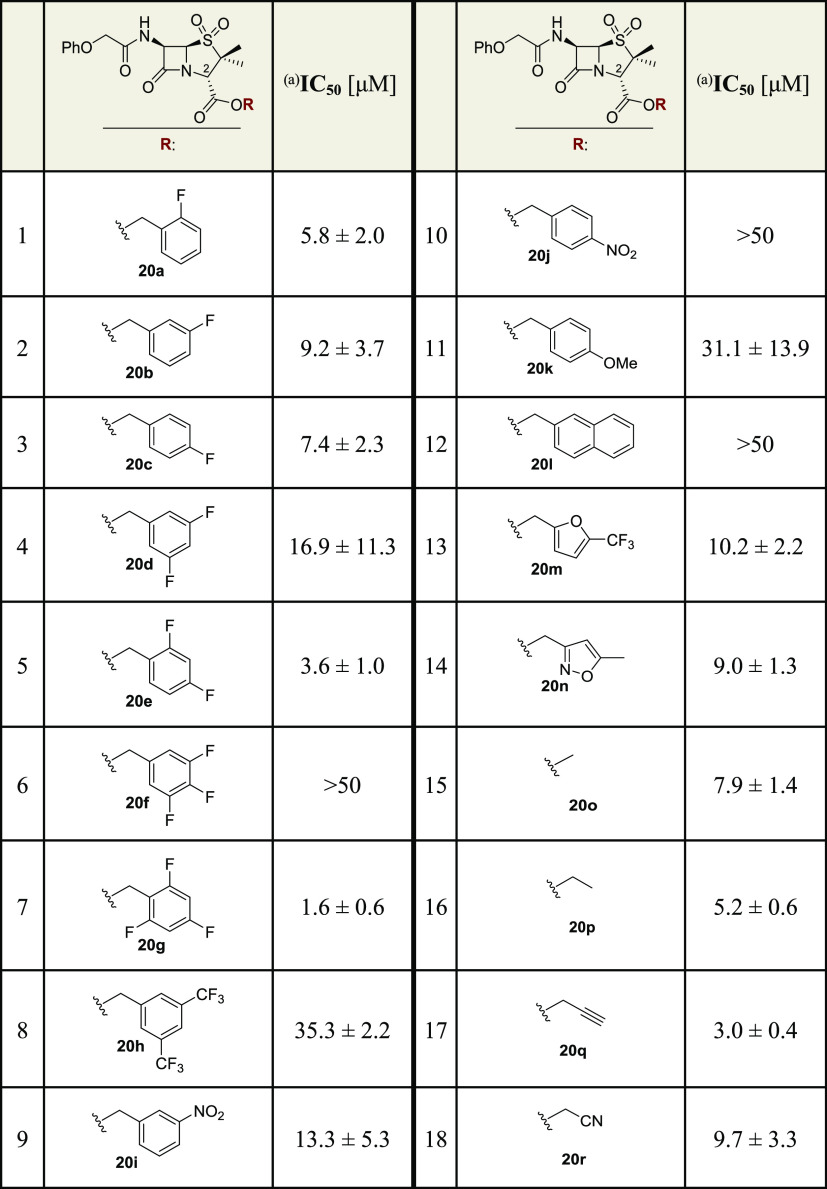
Inhibition of SARS-CoV-2 M^pro^ by Penicillin V Sulfone Esters

a M^pro^ inhibition assays were performed using SPE-MS as described in the Experimental Section employing SARS-CoV-2 M^pro^ (0.15 μM) and a substrate (2.0 μM). Results are means of at least two independent runs, each composed of technical duplicates (*n* ≥ 2; mean ± SD). Representative dose–response curves are shown in Supporting Information Figure S4.

The benzyl esters **20h** and **20i**, which bear a relatively bulky and electron-withdrawing trifluoromethyl or nitro *meta*-substituent, inhibited M^pro^ (Table 2, entries 8 and 9), while the *para*-substituted benzyl esters **20j** and **k**, as well as the 2-naphthylmethyl ester **20l**, do not, at least efficiently, inhibit M^pro^ (Table 2, entries 10–12).

The presence of a penicillin C2 benzyl ester derivative is not required for efficient M^pro^ inhibition, as apparent by the penicillin V sulfone esters **20m** and **20n** with a heteroaromatic ester group, which inhibit M^pro^, albeit with slightly reduced potency compared to **10** (Table 2, entries 13 and 14). By contrast, the corresponding alkyl esters **20o–r** inhibit with similar potency as the penicillin V sulfone benzyl ester **10** (Table 2, entries 15–18). However, in general, the corresponding penicillin V esters **19a–r** and the penicillin V (*S*)-sulfoxide esters **21a–r** did not manifest substantial levels of M^pro^ inhibition (Supporting Information Table S2), in accord with the initial SAR results (Table 1).

### Penicillin C6 Side Chain Modulates M^pro^ Inhibition

**Scheme 2:**
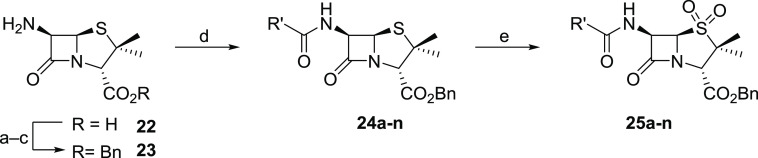
Synthesis of Penicillin Sulfone Benzyl Esters from (+)-6-Aminopenicillanic Acid (6-APA, **22**) Reagents and conditions: (a) benzylbromide, triethyl amine, CH_2_Cl_2_, 0 °C; (b) *para*-toluenesulfonic acid, acetone, rt; (c) NaHCO_3_, ethyl acetate/H_2_O, rt, 33% over three steps; (d) carboxylic acid, COMU,^58^ DMF, 0 °C to rt, 44–82%; (e) mCPBA, CH_2_Cl_2_, 0 °C to rt, 11–71%. Note that the C6 NHCbz penicillin benzyl ester **24a** was synthesized by a different sequence, as described in the Supporting Information.

A set of penicillin sulfone benzyl esters with different C6 amido groups were synthesized in three steps from commercially sourced (+)-6-aminopenicillanic acid (6-APA, **22**) to investigate the impact of the C6 side chain on M^pro^ inhibition (Scheme 2). Initially, 6-APA was transformed into its benzyl ester (**23**), which was then used in amide bond-forming reactions with an appropriate carboxylic acid using COMU^58^ as a coupling reagent. The resultant penicillin derivatives **24a–n** were oxidized with mCPBA to the penicillin sulfones **25a–n**.

The results of using the SPE-MS assay to test the penicillin sulfone benzyl esters **25a–n** for M^pro^ inhibition reveal that the presence and position of a C6 phenoxyacetyl ether oxygen is important in enabling efficient M^pro^ inhibition by the tested compounds (Table 3), in agreement with the observation that penicillin G sulfone benzyl ester **11** did not inhibit M^pro^ (Table 3, entry 2).^15^ Swapping the C6 phenoxyacetyl ether oxygen from the amide β-position to the α-position, as in urethane **25a**, abolished M^pro^ inhibition (Table 3, entry 3). The substitution of the C6 phenoxyacetyl ether oxygen for a methylene group substantially diminished M^pro^ inhibition (IC_50_ ∼ 45.7 μM, Table 3, entry 4), whereas the substitution of the penicillin V C6 side chain for the dicloxacillin C6 side chain, which does not bear an oxygen atom at the same position, abolished inhibition completely (Table 3, entry 5). Additionally, substitution of the C6 phenoxyacetyl ether oxygen for an NH group, as present in penicillin sulfone benzyl esters **25d–g**, results in loss of inhibition (Table 3, entries 6–9).

**Table 3:**
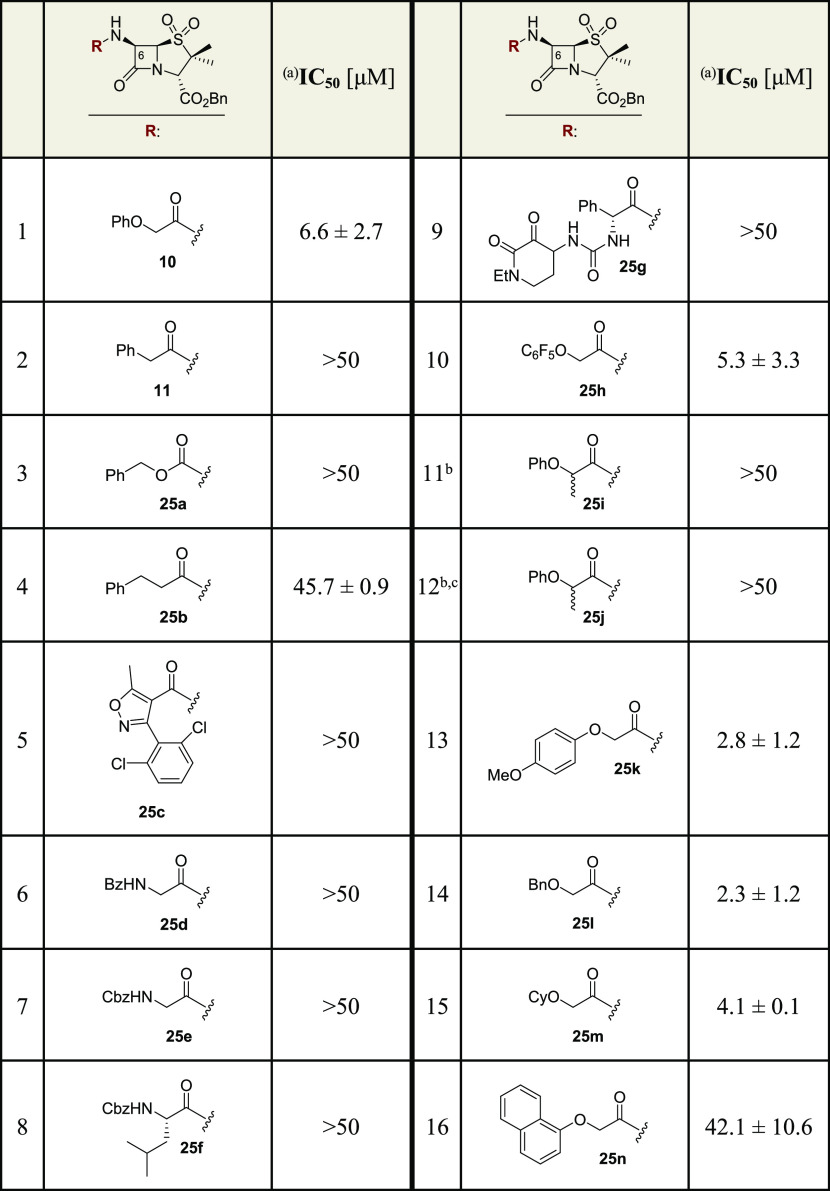
Inhibition of SARS-CoV-2 M^pro^ by Penicillin Sulfone Benzyl C6 Derivatives

a M^pro^ inhibition assays were performed using SPE-MS as described in the Experimental Section employing SARS-CoV-2 M^pro^ (0.15 μM) and a substrate (2.0 μM). Results are means of at least two independent runs, each composed of technical duplicates (*n* ≥ 2; mean ± SD). Representative dose–response curves are shown in Supporting Information Figure S4.

b Used as a 1:1 mixture of diastereomers.

c Used as the 4-fluorobenzyl ester. Bn: −CH_2_Ph, Cy: −C_6_H_11_, Bz: −C(O)C_6_H_5_, Cbz: −C(O)OCH_2_Ph.

These results suggest that the ability of the C6 phenoxyacetyl ether oxygen to function as a hydrogen bond acceptor/Lewis acid/conformation restrictor may be important for efficient M^pro^ inhibition, as supported by preliminary molecular docking studies.^15^ The effect of the Lewis acidity of the C6 phenoxyacetyl ether oxygen was probed by substituting its phenyl substituent for an electron-withdrawing pentafluorophenyl substituent (i.e. **25h**); however, this substitution did not alter M^pro^ inhibition substantially (Table 3, entry 10). By contrast, decreasing the accessibility of the C6 phenoxyacetyl ether oxygen by introducing an alkyl-substituent α to the ether oxygen, as in **25i** and **25j**, resulted in substantially reduced inhibition (Table 3, entries 11 and 12). Increasing the electron-donating capability of the phenyl ether by introducing a methoxy substituent at its *para*-position (**25k**) appeared to improve inhibition (IC_50_ ∼ 2.8 μM, Table 3, entry 13).

Modifying the steric bulk of the C6 phenoxyacetyl ether by substituting the phenyl group for a benzyl group (**25l**) appeared to improve inhibition (IC_50_ ∼ 2.3 μM, Table 3, entry 14), while its substitution by a cyclohexyl group (**25m**) did not substantially affect M^pro^ inhibition (IC_50_ ∼ 4.1 μM, Table 3, entry 15). By contrast, its substitution by a more bulky and rigid 1-naphthyl group (**25n**) resulted in substantially decreased inhibition (IC_50_ ∼ 42.1 μM, Table 3, entry 16).

### C6 Dibromo-Penicillins Are Efficient M^pro^ Inhibitors

Considering the importance of the penicillin V sulfone benzyl ester C6 side chain on inhibitor potency, the corresponding C6 mono- and dibromo-substituted penicillin sulfones **26**–**32** were prepared because such substitutions alter the reaction outcome of β-lactams with nucleophilic serine β-lactamases.^59−62^ The C6 mono- and dibrominated penicillins were synthesized from 6-APA (**22**) as reported^63−65^ and investigated for M^pro^ inhibition using the SPE-MS assay (Table 4).

**Table 4:**
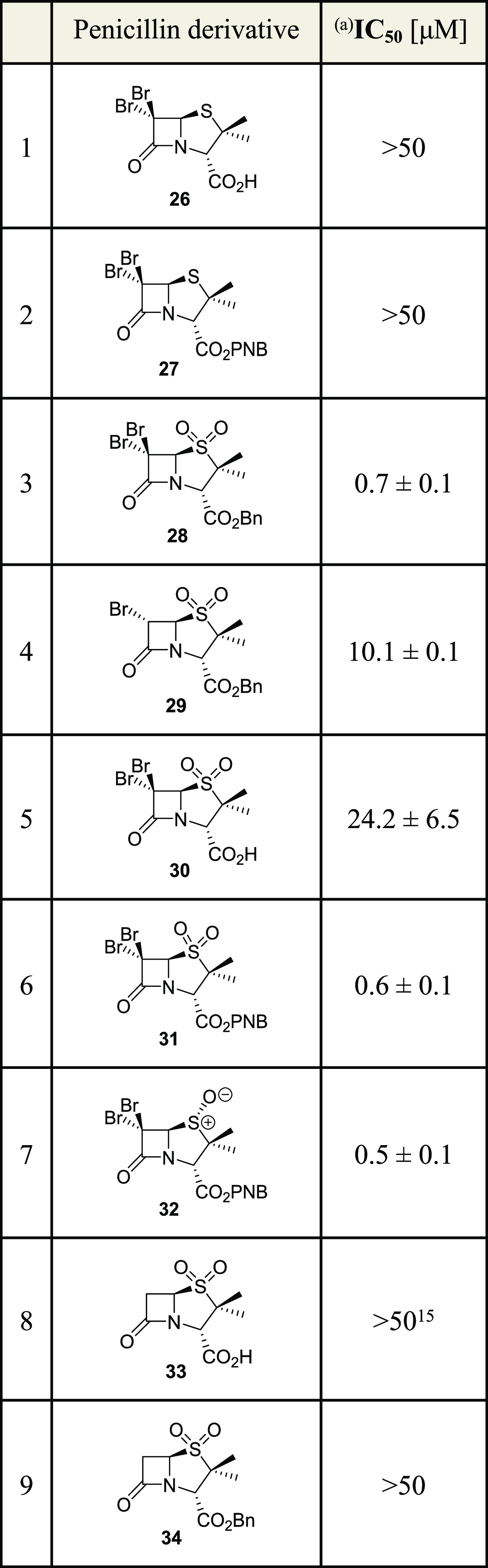
Inhibition of SARS-CoV-2 M^pro^ by C6 Mono- and Dibromo-Penicillin Derivatives

a M^pro^ inhibition assays were performed using SPE-MS as described in the Experimental Section employing SARS-CoV-2 M^pro^ (0.15 μM) and a substrate (2.0 μM). Results are means of at least two independent runs, each composed of technical duplicates (*n* ≥ 2; mean ± SD). Representative dose–response curves are shown in Supporting Information Figure S4. Bn: −CH_2_Ph; PNB: −CH_2_C_6_H_4_(4-NO_2_).

In agreement with the previous SAR studies (Table 1), neither 6,6-dibromopenicillanic acid **26** nor its *para*-nitrobenzyl ester derivative **27** inhibited M^pro^ (Table 4, entries 1 and 2). By contrast, the 6,6-dibromopenicillanic acid sulfone benzyl ester **28** was the most efficient penicillin M^pro^ inhibitor identified so far (IC_50_ ∼ 0.7 μM, Table 4, entry 3), while the corresponding 6-bromopenicillanic acid sulfone benzyl ester **29** was substantially less efficient in inhibiting M^pro^ (IC_50_ ∼ 10.1 μM, Table 4, entry 4). Surprisingly, however, the 6,6-dibromopenicillanic acid sulfone **30**, which is an intermediate in the synthesis of **28**, also showed inhibition (IC_50_ ∼ 24.2 μM, Table 4, entry 5); thus, for the first time in our SAR studies, inhibition was observed for a penicillanic acid, which is structurally closely related to sulbactam (**33**), a penam sulfone that is clinically used as a serine β-lactamase inhibitor (Table 4, entry 8).^66,67^ The importance of the C6 dibromo substituents for efficient M^pro^ inhibition is further highlighted by the observation that neither sulbactam (**33**) nor its benzyl ester derivative (**34**) displayed notable inhibition (Table 4, entries 8 and 9), in accord with the reported inability of sulbactam to inhibit M^pro^.^15^

By contrast with the previous SAR studies that showed less efficient M^pro^ inhibition of the penicillin V sulfone *para*-nitrobenzyl ester **20j** compared to the benzyl ester **10** (Table 2), 6,6-dibromopenicillanic acid sulfone *para*-nitrobenzyl ester **31** had a similar potency as the corresponding benzyl ester **28** (IC_50_ ∼ 0.6 μM, Table 4, entry 6); the 6,6-dibromopenicillaic acid (*R*)-sulfoxide *para*-nitrobenzyl ester **32** also inhibited with similar efficiency (IC_50_ ∼ 0.5 μM, Table 4, entry 7). Note that mCPBA oxidation of **27** occurs from the least hindered side to afford a (*R*)-configured sulfoxide in the absence of a C6 amido directing group. The combined results suggest that the binding mode and/or mechanism of inhibition of the C6 mono- and dibromo-penicillin derivatives **28**–**32** differs compared to those of the C6 amido penicillin V derivatives previously investigated (Tables 1–3). Note that studies with β-lactamases imply the modes of inhibition by the C6 dibromo penicillin derivatives, which are presently under investigation, might be complex.^59−62^

### Mass Spectrometric Evidence That Selected Penicillin Derivatives Inhibit by Covalent M^pro^ Modification

**Figure 2:**
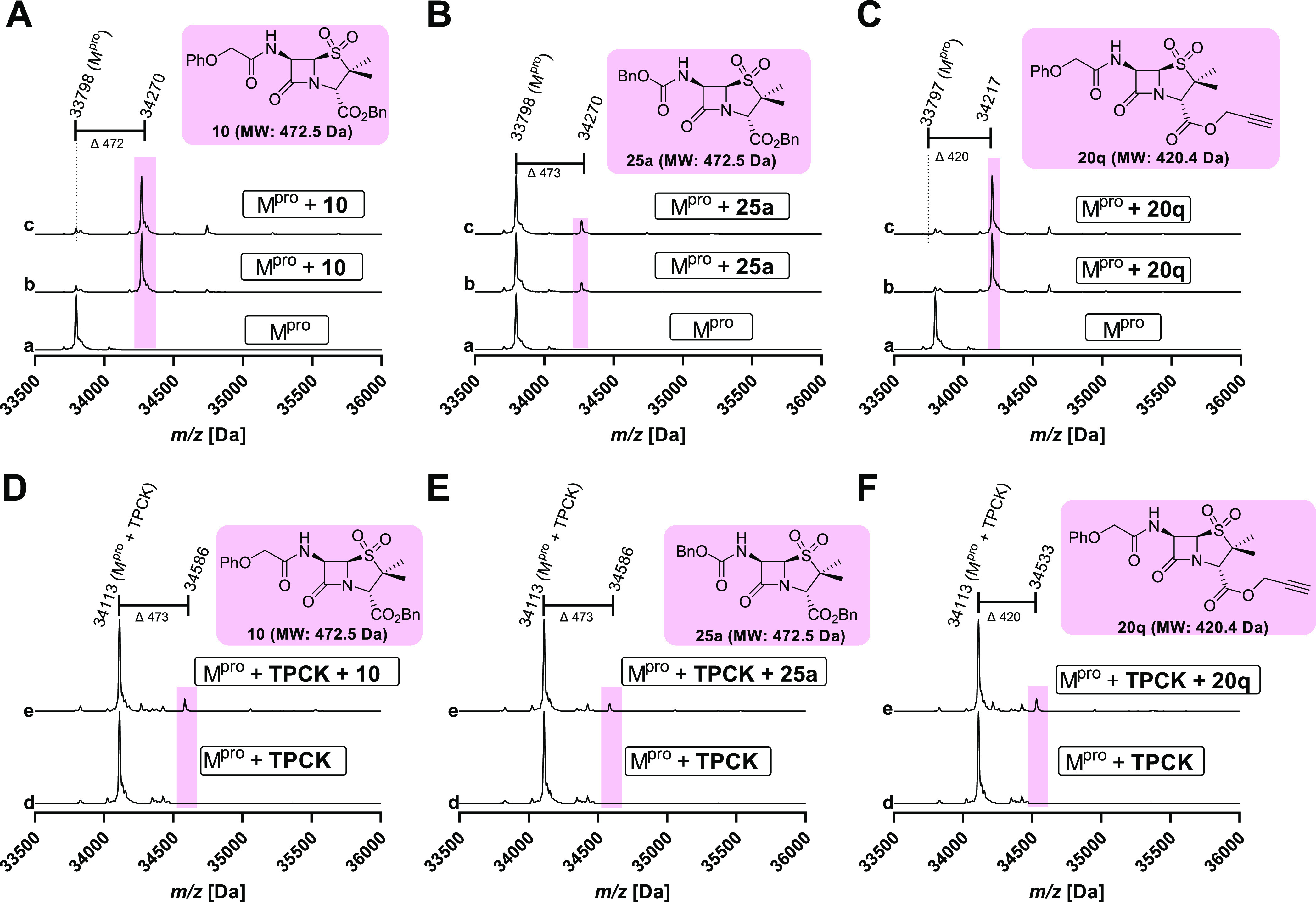
Evidence that penicillin V sulfone ester derivatives inhibit M^pro^ by selective active site cysteine covalent modification. M^pro^ assays with penicillin sulfone ester derivatives **10** (A, D), **25a** (B, E), and **20q** (C, F) were performed in the absence (A–C) or presence (D–F) of TPCK using SPE-MS as described in the Experimental Section employing SARS-CoV-2 M^pro^ (2.0 μM), penicillin sulfone ester derivatives (11 μM for b, and 33 μM for c and e), and TPCK (10 μM for d and e). The reactions were incubated for either 45 min (a–c) or 180 min (with TPCK, d and e) followed by additional 60 min (with a penicillin sulfone ester derivative, e) prior to analysis by SPE-MS. The reactions were performed in technical duplicates (Supporting Information Figure S5). Note (i) the clear evidence for the covalent reaction of **10** and **20q** but not **25a**, and (ii) that reaction is ablated by pretreatment of M^pro^ with the active site binding inhibitor TPCK.

Selected compounds were tested for reaction with M^pro^ using protein-observed MS to inform on their mechanism of inhibition (Figure 2). Initially, M^pro^ was incubated with a ∼6-fold excess of the penicillin sulfone ester for 45 min and then analyzed by MS. The results imply that penicillins **10** and **20q** modify M^pro^ by, at least predominantly, covalent reaction with a single nucleophilic protein residue, likely the active site Cys145 (Figure 2A,C). By contrast, penicillin **25a** with a C6 CbzNH group does not modify M^pro^ covalently (Figure 2B). When the inhibitor concentration was increased to ∼17-fold to account for the possibility of modifying all 12 M^pro^ cysteine or other residues, only low levels of a second covalent M^pro^ modification by the inhibitors were observed (Figure 2A–C). No evidence for fragmentation of the inhibitors once bound to M^pro^ was accrued.

To investigate the site of covalent modification, M^pro^ was first preincubated with *N*-*para*-toluenesulfonyl-l-phenylalanine chloromethyl ketone (TPCK), which is reported to selectively alkylate the active site cysteine (Cys145) versus the other 11 M^pro^ cysteine residues,^15^ then incubated with selected penicillin sulfone ester derivatives, i.e., **10**, **20q**, and **25a** (Figure 2D–F). The results reveal that the penicillin sulfone ester derivatives, in particular **10** and **20q**, do not efficiently react covalently with the active site (Cys145) TPCK-blocked M^pro^, in agreement with a mechanism involving a covalent reaction with the nucleophilic active site cysteine residue Cys145. This mode of inhibition is consistent with the results obtained for the other penicillin sulfone ester derivatives investigated in this study (Supporting Information Figure S5), while γ-lactam **17** appears to inhibit M^pro^ via a different mechanism (Supporting Information Figure S6), potentially involving noncovalent binding.

### Crystallographic Evidence That Penicillin Derivatives Selectively Inhibit M^pro^ by *S*-Acylation of Cys145

To investigate the mode of M^pro^ inhibition by penicillin derivatives, we carried out crystallographic studies and obtained a structure of M^pro^ complexed with a penicillin sulfone **20e**-derived ligand following cocrystallization (*C*2 space group, 2.0 Å resolution; Supporting Information Figure S7). The structure was solved by molecular replacement using a reported M^pro^ structure (PDB ID: 6YB7(29)) as a search model. The overall fold of the structure is similar to those previously reported for M^pro^ (RMSD = 0.41 Å for M^pro^ complexed with PF-07321332 (**8**, nirmatrelvir; PDB ID: 7VH8);^68^ Supporting Information Figure S7).

**Figure 3:**
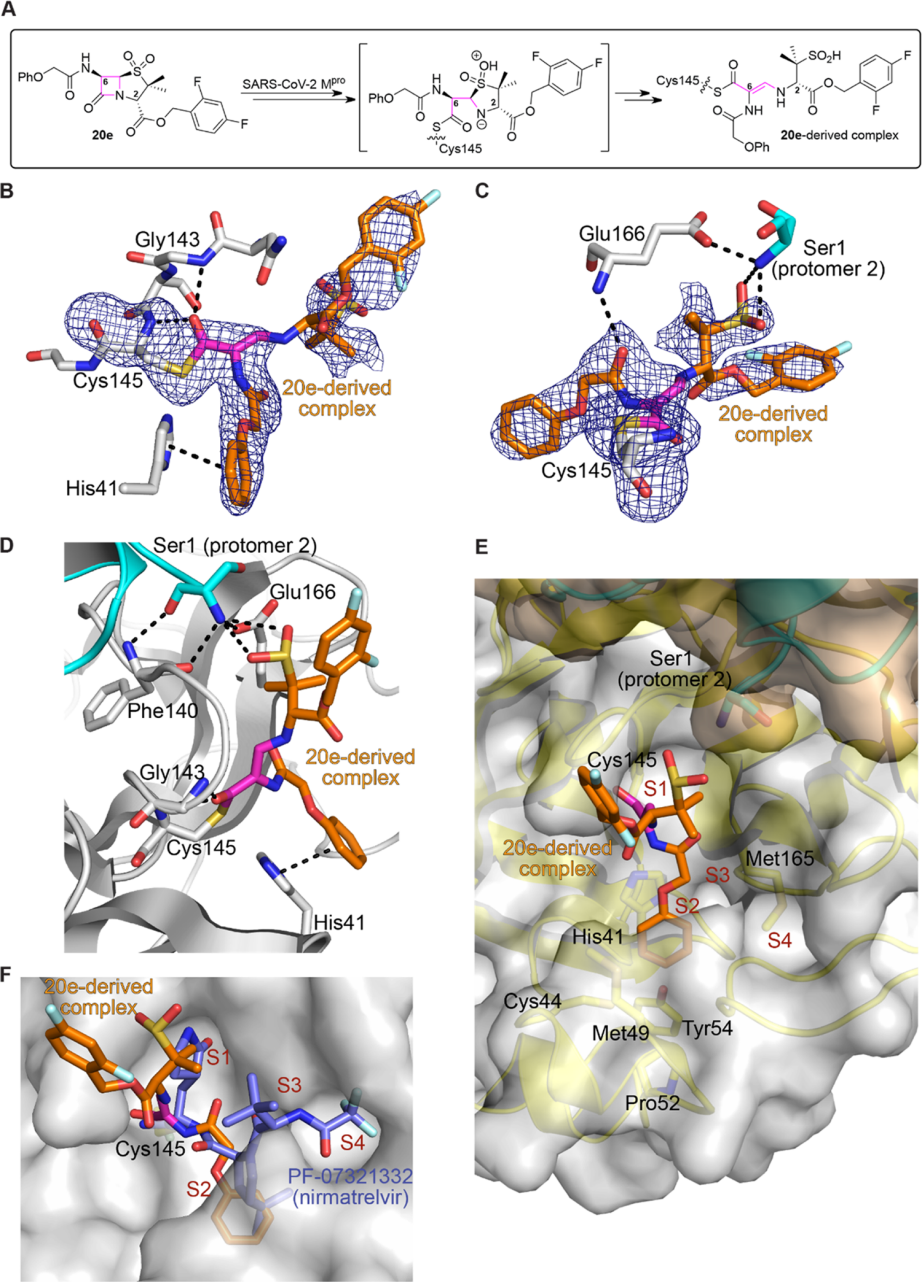
Crystallographic evidence that penicillin V sulfone ester derivatives inhibit M^pro^ by active site cysteine covalent modification. Color code: M^pro^: gray (protomer 1) and cyan (protomer 2); carbon-backbone of the **20e**-derived complex is in orange, with the β-lactam ring-derived carbon-backbone in magenta; oxygen: red; nitrogen: blue; sulfur: yellow; and fluorine: light blue. (A) Reaction of penicillin sulfone **20e** with SARS-CoV-2 M^pro^. (B, C) Representative OMIT electron density map (mF_o_-DF_c_) contoured to 2.5σ around Cys145 and the **20e**-derived complex showing clear evidence for (B) β-lactam ring opening by the active site Cys145 leading to thioester formation and (C) positioning of the SO_2_H group of the **20e**-derived complex formed by opening of the thiazolidine sulfone ring to enable interactions with the main chain amino group of Ser1 of the second M^pro^ protomer. (D) Phe140, Glu166, and the SO_2_H group of the **20e**-derived complex are positioned to interact with Ser1 of the second M^pro^ protomer. (E) C6 amido penicillin-derived side chain of the **20e**-derived complex binds in the hydrophobic S2 M^pro^ binding site. (F) Superimposition of active sites’ views of the M^pro^:**20e**-derived complex and the M^pro^:PF-07321332 (slate blue: carbon-backbone of **8**, nirmatrelvir; PDB ID: 7VH8(68)) structures.

Consistent with our MS data (Figure 2), analysis of the electron density at the M^pro^-ligand complex active site provides clear evidence for active site Cys145 *S*-acylation via a reaction with the β-lactam ring of penicillin **20e** (Figure 3A), in a manner reminiscent of the covalent reaction of β-lactamases with penicillins and l,d-transpeptidases employing a nucleophilic cysteine with carbapenems.^69,70^ The thioester carbonyl of the penicillin sulfone **20e**-derived complex (corresponding to the β-lactam carbonyl of **20e**) is positioned to interact with the main chain amino group of Cys145 and Gly143 (3.0 and 2.8 Å, respectively) (Figure 3B). Note that no evidence for acylation of other M^pro^ residues by **20e** was observed in the crystal structure, indicating a selective covalent reaction, at least under the cocrystallization conditions, consistent with the MS studies.

The amide carbonyl of the C6 penicillin-derived side chain is positioned to interact with the main chain amino group of Glu166 (3.1 Å) (Figure 3C). Further, the C6 amido penicillin-derived side chain binds in the hydrophobic S2 binding site formed *inter alia* by the side chains of His41, Cys44, Met49, Pro52, Tyr54, and Met165. The C6 side chain phenyl group is positioned to π-stack in an offset manner with the imidazole side chain of His41 (3.8 Å), which is part of the catalytic dyad; analogous interactions have been observed with other M^pro^ inhibitors.^33,71^ It could be that the C6 phenoxy ether oxygen, which is important for efficient inhibition (Table 3), helps position the phenyl group of the C6 side chain to productively interact with His41, though it may also be important in binding prior to covalent reactions leading to the crystallographically observed complex. Notably, the S1 binding pocket is not occupied in the M^pro^:**20e**-derived complex structure; substitution at the penicillin sulfone C6 position is of interest in this regard and will be explored in future work. The penicillin C2-derived ester projects out the active site, rationalizing the relatively flat SAR at this position; however, as with C6 ether oxygen, the C2 ester may be important in initial inhibitor binding.

Interestingly, the crystallographic data imply that the opening of the penicillin thiazolidine sulfone ring of **20e** via C5–S bond cleavage follows an initial covalent reaction of Cys145 with the β-lactam, to give an acyclic enamine/imine. An analogous reaction occurs during serine β-lactamase inhibition by sulbactam and tazobactam.^72,73^ We carried out trial refinements with both the enamine and imine complexes; analysis of the electron density implies the presence of a planar C5–C6 bond (penicillin numbering), suggesting the presence of the enamine, but we cannot rule out the additional partial presence of the imine tautomer. The SO_2_H group formed by opening of the thiazolidine sulfone ring projects toward the side chain of Glu166 (3.8 Å) and the main chain amino group of Ser1 (3.0 and 3.3 Å), the latter being the N-terminus of the second protomer making up the functional M^pro^ dimer (Figure 3C–E).

## Discussion

Antibacterial drugs containing a β-lactam ring are among the most successful of all small-molecule therapeutics. Their mechanism of action involves reaction with a nucleophilic serine in bacterial transpeptidases to afford stable acyl–enzyme complexes.^74^ They are also important inhibitors of serine β-lactamases, which are mechanistically related to transpeptidases.^75^ Despite the widespread use of β-lactams as antibacterials and work showing they have the potential to inhibit other classes of nucleophilic enzymes,^76,77^ including human^78−83^ and viral^84−87^ serine proteases, they have found limited utility in other therapeutic fields. The reasons for this are unclear but, at least in the case of bicyclic β-lactams such as penicillins, may in part reflect synthetic challenges and/or long-term stability issues. β-Lactams also have potential as useful inhibitors of nucleophilic cysteine enzymes, as shown, for example, by the inhibition of (i) human cathepsins by monocyclic β-lactams,^88,89^ (ii) viral cysteine proteases by spirocyclic β-lactams,^90^ and (iii) mycobacterial l,d-transpeptidases by bicyclic β-lactams.^69,70,91,92^ Although there is considerable scope for further optimization, our results highlight the potential of β-lactams as covalently reacting inhibitors of SARS-CoV-2 M^pro^ and, by implication, other (viral) nucleophilic cysteine proteases, including SARS-CoV-2 PL^pro^.

Recently, we reported MS-based SARS-CoV-2 M^pro^ and PL^pro^ assays, which monitor protease-catalyzed substrate hydrolysis and/or protease modification and which are suitable for inhibition studies.^15,93^ The MS-based M^pro^ assays enabled the identification of certain β-lactams, notably the penicillin V sulfone benzyl ester **10**, as covalently reacting M^pro^ inhibitors.^15^ In the current study, we report SAR studies that show the potency of **10** can be optimized by about 10-fold, i.e., from IC_50_ ∼ 6.5 μM for **10** to IC_50_ ∼ 0.6 μM for **28**, **31**, and **32** (Table 4). In general, for efficient M^pro^ inhibition, the penicillin sulfone oxidation state is preferred over the sulfoxide and sulfide oxidation states (Table 1); however, while the (*S*)-configured penicillin sulfoxides do not inhibit efficiently (Table 1, entries 3 and 4), the corresponding (*R*)-configured sulfoxides do inhibit (Table 4, entry 9). The importance of the oxidized sulfur in inhibition is consistent with the ring opening of the thiazolidine ring during inhibition, as supported by crystallographic studies with **20e** (Figure 3). Further, for penicillins bearing a C6 amido side chain, the (6*R*)-configuration is preferred over the (6*S*)-configuration (Table 1).

Protein-observed MS and crystallographic studies imply that, at least, some of the penam sulfones selectively react with the active site Cys145 thiol to give a stable acyl–enzyme complex (Figures 2 and 3). Crystallographic analysis revealed that, at least in the case of penicillin **20e**, β-lactam opening is followed by opening of the five-membered penicillin ring to give, at least predominantly, an acyclic enamine, a reaction precedented in the inhibition mechanisms of nucleophilic serine β-lactamases by clinically used drugs sulbactam and tazobactam.^72,73^ Although care should be taken in assuming crystallographically observed complexes necessarily reflect those relevant in solution, the structure nonetheless highlights the potential for M^pro^ inhibition via cysteine-acylation and for subsequent reaction, leading to a stable acyl–enzyme complex.

The possibility of reactions subsequent to initial noncovalent binding/acylation may contribute to the rather complex SAR, including for the C2 ester derivatives, with both small alkyl (e.g., **20o** and **20p**) and benzyl esters being potent inhibitors (Table 2). In the M^pro^:**20e**-derived complex structure, the C2 ester projects away from the active site; the interaction of the SO_2_H group formed by opening of the thiazolidine sulfone ring with the main chain amino group of Ser1 of the second M^pro^ protomer may stabilize this conformation. It is likely that the C2 ester group occupies a different conformation prior to opening of the five-membered penicillin ring; thus, it may be important in initial M^pro^ binding. Modification of the C2 ester group may enable tuning of pharmacokinetic properties, for example, to optimize cell permeability. Crystal structure analysis suggests that the penicillin C6 amido penicillin side chain binds in the P3 binding pocket (Figure 3). The C6 phenoxyacetyl ether oxygen of penicillin V sulfone derivatives are potent M^pro^ inhibitors in contrast to the penicillin G derivatives, which lack the ether oxygen (Table 3); the structure suggests the C6 phenoxy ether oxygen may help position the phenyl group of the C6 side chain to productively interact with the His41 imidazole ring, though it may also be important in binding prior to covalent reaction, leading to the crystallographically observed complex, potentially by interaction with the Asn142 side chain, as indicated by docking studies.^15^

It should be noted that a covalent reaction is not necessarily a prerequisite for useful inhibition of M^pro^ by a β-lactam.^15^ Although the efficient reaction of penicillin and related bicyclic β-lactams with transpeptidases/β-lactamases is often proposed to reflect the reactive nature of the β-lactam ring, the bicyclic β-lactam ring system is also a mimic of a strained conformation of the scissile substrate peptide bond,^74^ thus β-lactams have the potential as noncovalent M^pro^ inhibitors. Notably, a γ-lactam derivative (i.e., **17**) was less active than the analogous penicillin **10** (Table 1) but still clearly showed inhibition, suggesting lactams (and related acylating agents) other than β-lactams have potential as active site binding M^pro^ inhibitors, as reported to be the case for transpeptidases/β-lactamases^94,95^ and a viral serine protease.^96^

Interestingly, among the most potent compounds identified in our work were the C6 dibromo-penicillin sulfones **28**, **31**, and **32** (Table 4); ongoing mechanistic studies on these compounds involving protein-observed MS suggest initial covalent M^pro^ binding is followed by rapid subsequent reaction to give new species. Although the precise mechanisms of action of these compounds remain to be determined, work on penicillin C6 bromo derivatives and β-lactamase inhibition has shown that related compounds can react to give acyl–enzyme complexes that undergo subsequent rearrangements.^59−62^ These results suggest the unexploited potential for “mechanism-based” inhibition of M^pro^ and related nucleophilic cysteine enzymes, which may complement drug development efforts on mechanistically distinct substrate mimics, such as PF-07321332 (**8**, nirmatrelvir, Figures 1G and 3F).

## Conclusions

The combined results highlight the potential of β-lactams, including penicillin derivatives prepared by semisynthesis from natural products, as covalently reacting M^pro^ inhibitors, though noncovalent inhibition by them is also possible. Given the proven efficacy of β-lactams and related covalently reacting groups as antibacterials and β-lactamase inhibitors, we suggest that they should be explored as antiviral drugs.

## Experimental Section

The syntheses and characterizations of the penicillin derivatives used in this work are disclosed in the associated Supporting Information. All compounds are ≥95% pure by NMR and HPLC analysis unless stated otherwise. NMR spectra and HPLC traces are shown in the associated Supporting Information.

### M^pro^ Inhibition Assays

SPE-MS M^pro^ inhibition assays were performed as reported,^15^ however, using a 37mer peptide (ALNDFSNSGSDVLYQPPQTSITSAVLQ/SGFRKMAFPS-NH_2_) as a substrate rather than an 11mer peptide (TSAVLQ/SGFRK-NH_2_). The 37mer peptide was synthesized by solid-phase peptide synthesis as a C-terminal amide and purified by GL Biochem (Shanghai) Ltd. (Shanghai, China). Recombinant SARS-CoV-2 M^pro^ was prepared according to established procedures;^15^ note that fresh aliquots, which were not frozen more than once, were used for inhibition assays. Solutions of the inhibitors (100% DMSO) were dry-dispensed across 384-well polypropylene assay plates (Greiner) in an approximately 3-fold and 11-point dilution series (100 μM top concentration) using an ECHO 550 acoustic dispenser (Labcyte). DMSO and formic acid were used as negative and positive inhibition controls, respectively. The final DMSO concentration was kept constant at 0.5%_v/v_ throughout all experiments (using the DMSO backfill option of the acoustic dispenser). Each reaction was performed in technical duplicates in adjacent wells of the assay plates, and assays were performed in at least two independent duplicates.

In brief, the Enzyme Mixture (25 μL/well), containing M^pro^ (0.3 μM) in buffer (20 mM HEPES, pH 7.5, 50 mM NaCl), was dispensed across the inhibitor-containing 384-well assay plates with a multidrop dispenser (Thermo Fischer Scientific) at 20 °C under an ambient atmosphere. The plates were subsequently centrifuged (1000 rpm, 10 s) and incubated for 15 min at 20 °C. Note that we previously incubated M^pro^ with inhibitors for 30 or 60 min, resulting in more efficient inhibition.^15^ The substrate mixture (25 μL/well), containing ALNDFSNSGSDVLYQPPQTSITSAVLQ/SGFRKMAFPS-NH_2_ (4.0 μM) in buffer (20 mM HEPES, pH 7.5, 50 mM NaCl), was added using the multidrop dispenser. The plates were centrifuged (1000 rpm, 10 s), and after incubating for 6 min, the reaction was stopped by addition of 10%_v/v_ aqueous formic acid (5 μL/well). The plates were then centrifuged (1000 rpm, 30 s) and analyzed by MS.

MS analyses were performed using a RapidFire RF 365 high-throughput sampling robot (Agilent) attached to an iFunnel Agilent 6550 accurate mass quadrupole time-of-flight (Q-TOF) mass spectrometer operated in the positive ionization mode. Assay samples were aspirated under vacuum for 0.6 s and loaded onto a C4 solid-phase extraction (SPE) cartridge. After loading, the C4 SPE cartridge was washed with 0.1%_v/v_ aqueous formic acid to remove nonvolatile buffer salts (5.5 s, 1.5 mL/min). The peptide was eluted from the SPE cartridge with 0.1%_v/v_ aqueous formic acid in 85/15_v/v_ acetonitrile/water into the mass spectrometer (5.5 s, 1.5 mL/min), and the SPE cartridge was re-equilibrated with 0.1%_v/v_ aqueous formic acid (0.5 s, 1.25 mL/min). The mass spectrometer parameters were as follows: capillary voltage (4000 V), nozzle voltage (1000 V), fragmentor voltage (365 V), gas temperature (280 °C), gas flow (13 L/min), sheath gas temperature (350 °C), and sheath gas flow (12 L/min). For data analysis, the *m*/*z* +3 charge state of the 37mer peptide (substrate) and the *m*/*z* +1 charge state of the SGFRKMAFPS-NH_2_ C-terminal product peptide were used to extract and integrate ion chromatogram data using RapidFire Integrator software (Agilent). Data were exported into Microsoft Excel and used to calculate the % conversion using the equation: % conversion = 100 × (integral C-terminal product peptide)/(integral C-terminal product peptide + integral 37mer substrate peptide). Normalized dose–response curves (formic acid and DMSO controls) were obtained from the raw data by nonlinear regression (GraphPad Prism 9) and used to determine IC_50_-values. For compounds **15**, **16**, **25g**, **25c**, **29**, and **30**, the 11mer peptide was used as the substrate.

### Protein-Observed M^pro^ Assays

Solutions of the inhibitors (100% DMSO) were dry-dispensed across 384-well polypropylene assay plates (Greiner) (for 11 or 33 μM top concentrations) using an ECHO 550 acoustic dispenser (Labcyte). DMSO was used as a negative control. Each reaction was performed in technical duplicates. The enzyme mixture (50 μL/well), containing M^pro^ (2.0 μM) in buffer (20 mM HEPES, pH 7.5), was dispensed across the penicillin-containing 384-well assay plates with a multidrop dispenser (Thermo Fischer Scientific). The reaction mixture was incubated for 45 min at 20 °C under an ambient atmosphere prior to analysis by SPE-MS.

To investigate the importance of the covalent modification of the active site Cys145 for M^pro^ inhibition, M^pro^ (2.0 μM) was incubated with the selective Cys145-alkylating agent TPCK^15^ (10 μM) in buffer (20 mM HEPES, pH 7.5) for 3 h at 0 °C. The mixture was then dispensed across the penicillin-containing 384-well assay plates with a multidrop dispenser (Thermo Fischer Scientific) and incubated for 1 h at 20 °C under an ambient atmosphere prior to analysis by SPE-MS.

MS analyses were performed using a RapidFire RF 365 high-throughput sampling robot (Agilent) attached to an iFunnel Agilent 6550 accurate mass Q-TOF mass spectrometer using a C4 cartridge and the same parameters as described above, with the exception of the gas temperature that was reduced to 225 °C. Protein spectra were deconvoluted for the *m*/*z* range 850–1350 Da, with a resolution of 2 Da and with a 10–60 kDa cutoff using the MaxEnt1 function in Agilent MassHunter Version 7. The deconvoluted files were extracted as csv files, sorted using Enthought Canopy GUI, and normalized and plotted using GraphPad Prism 9.

### Crystallization

A frozen SARS-CoV-2 M^pro^ solution was thawed and diluted to 6 mg/mL (using 20 mM HEPES, pH 7.5, 50 mM NaCl). β-Lactam **20e** was added to the protein solution to a final concentration of 10 mM; the mixture was incubated for 2 h at ambient temperature prior to dispensing the plates. The drop composition was: 0.15 μL protein–ligand solution, 0.3 μL 11%_v/v_ PEG 4000, 0.1 M MES, pH 6.5, and 0.05 μL M^pro^ crystal seed stock. A M^pro^ crystal seed stock was prepared by crushing M^pro^ crystals with a pipette tip, suspending them in 30% PEG 4000, 5%_v/v_ DMSO, 0.1 M MES pH 6.5, and vortexing for 60 s with approximately 10 glass beads (1.0 mm diameter, BioSpec products). The reservoir solution was: 11%_v/v_ PEG 4K, 5%_v/v_ DMSO, 0.1 M MES, pH 6.5. Crystals were grown using the sitting drop vapor diffusion method at 20 °C and appeared within 24 h, reaching full size within 36 h. Crystals were looped after 1 week.

### Data Collection and Structure Determination

Diffraction data were collected on beamline I0-3 at the Diamond Light Source at 100 K using a wavelength of 0.9762 Å. Data were processed using Dials^97^ via Xia2^98^ and Aimless^99^ within CCP4i2.^100^ The datasets were phased using Molrep^101^ and the M^pro^ apo structure (PDB ID: 6YB7). Ligand restraints were generated using AceDRG.^102^ Typically, 97% of residues are in the favored regions of the Ramachandran plot, 2% in the allowed region, and 1% in high-energy conformations (2 residues). Crystal structures were manually rebuilt in Coot and refined using Refmac,^103^ Buster,^104^ and PDB_Redo (Supporting Information Table S3).^105^

The crystal structure data for SARS-CoV-2 M^pro^:**20e**-derived complex have been deposited in the Protein Data Bank (PDB) with accession code 7Z59.

## Supporting Information Available

Inhibition assays, synthesis, NMR analysis, representative dose-response curves, and crystallographic analysis; the use of 37mer, data collection, and reference statistics; general synthetic procedures; and experimental procedures (PDF)
Molecular formula strings (CSV)
