# Supplementary material for: Penicillin Derivatives Inhibit the SARS-CoV-2 Main Protease by Reaction with Its Nucleophilic Cysteine
Source: J Med Chem. 2022 May 12;65(11):7682–96. doi: 10.1021/acs.jmedchem.1c02214 (PMC9115881; doi:10.1021/acs.jmedchem.1c02214)
Supplement: Supplementary file 1 — jm1c02214_si_001.pdf [file jm1c02214_si_001.pdf]

Supporting Information

Penicillin derivatives inhibit the SARS-CoV-2 main protease by reaction with its nucleophilic cysteine

Tika R. Malla[1,#], Lennart Brewitz[1,#,*], Dorian-Gabriel Muntean[1], Hiba Aslam[1], C. David Owen[2,3], Eidarus Salah[1], Anthony Tumber[1], Petra Lukacik[2,3], Claire Strain-Damerell[2,3], Halina Mikolajek[2,3], Martin A. Walsh[2,3], and Christopher J. Schofield[1,*]

[1]Chemistry Research Laboratory, Department of Chemistry and the Ineos Oxford Institute for Antimicrobial Research, University of Oxford, 12 Mansfield Road, OX1 3TA, Oxford, United Kingdom.

[2]Diamond Light Source Ltd., Harwell Science and Innovation Campus, OX11 0DE, Didcot, United Kingdom.

[3]Research Complex at Harwell, Harwell Science and Innovation Campus, OX11 0FA, Didcot, United Kingdom.

[#]These authors contributed equally to this work.

*Email: christopher.schofield@chem.ox.ac.uk or lennart.brewitz@chem.ox.ac.uk

Table of contents

1. Supporting figures S2-S9
2. Supporting tables S10-S13
3. General synthesis information S14
4. General synthetic procedures S14-S15
5. Experimental procedures and compound characterizations S15-S46
6. References S47-S48
7. ^{1}H and ^{13}C NMR spectra of novel penicillin sulfones prepared for this study S49-S83
8. HPLC traces of selected penicillin sulfones prepared for this study S84-S94

1. Supporting figures

Supporting Figure S1. Robustness of the M^{pro} SPE-MS inhibition assays. Z'-factors[1] for inhibition assay plates analysed to determine IC_{50}-values. The Z'-factors >0.5 (grey line) indicate a stable and robust assay of high quality.[1] Z'-factors were determined according to the literature using Microsoft Excel.[1] SPE-MS inhibition assays were performed as described in the Experimental Section.

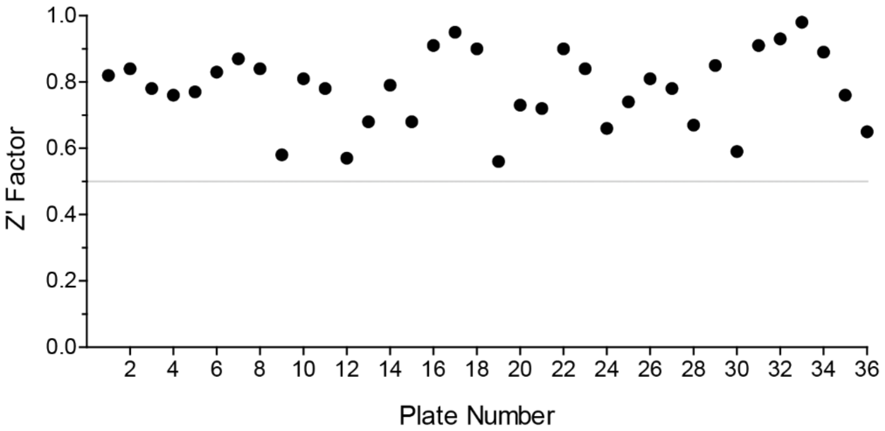

Supporting Figure S2. Synthesis of γ-lactam sulfone benzyl ester 17. The γ-lactam **17** was synthesized from *D*-penicillamine (**37**) following reported procedures for the synthesis of similar γ-lactams.[2-3] In brief, commercially-sourced *D*-penicillamine (**37**) was condensed with aldehyde **36**, which was obtained from Cbz-L-homoserine benzyl ester **35**[4] via Dess-Martin[5] oxidation,[6] to afford **38** as a single diastereomer. **38** was directly transformed into the corresponding benzyl ester **39**. The Cbz-protecting group of **39** was selectively removed under acidic conditions; following acylation of the resultant free C7 amino-group using phenoxyacetyl chloride, an oxidation of the thioether gave the corresponding sulfone **17**, the relative stereochemistry of which was determined using 2D NOESY NMR experiments (Supporting Figure S3). Note that the synthesis of an isomeric γ-lactam methyl ester bearing the penicillin G side chain at C6 instead of C7 has been reported.[7]

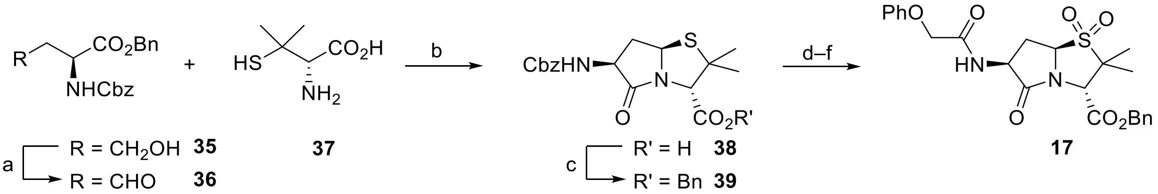

Reagents and conditions: a) Dess-Martin periodinane,[5] $NaHCO_3$, CH_2Cl_2, 0° C to rt, 88%; b) pyridine, rt, then: 120° C, 87%; c) benzylbromide, $NaHCO_3$, NaI, DMF, rt, 70%; d) 33%$_{v/v}$ HBr in AcOH, CH_2Cl_2, 0° C to rt, 89%; e) phenoxyacetyl chloride, trimethylamine, CH_2Cl_2, rt; 67%; f) mCPBA, CH_2Cl_2, rt, 56%.

Supporting Figure S3. NMR analysis of γ-lactam sulfone benzyl ester 17 supports its assigned relative stereochemistry. The 2D NOESY NMR analysis (blue arrows indicate selective nOe correlations) of the γ-lactam sulfone benzyl ester **17** reveals an intense cross peak of proton A with the protons B, while a less intense cross peak with protons C was observed. For protons C, an intense cross peak with proton D was observed, indicating close proximity of these protons. Cross peaks of proton E with protons D and G confirm the assigned relative stereochemistry. NMR spectroscopy of **17** was performed in $CDCl_3$ using a Bruker AVANCE AVIIIHD 600 machine equipped with a 5 mm BB-F/1H Prodigy N_2 cryoprobe.

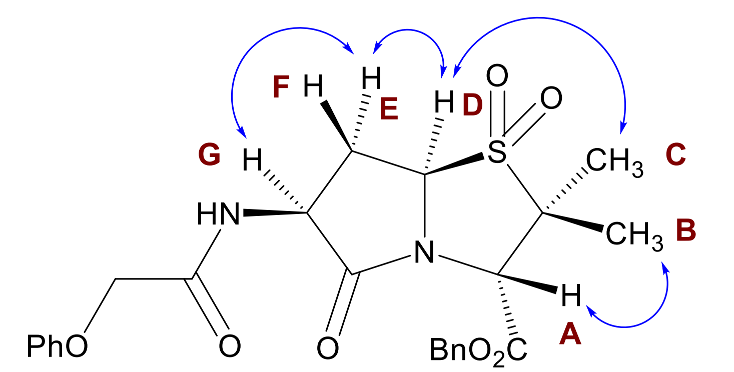

17

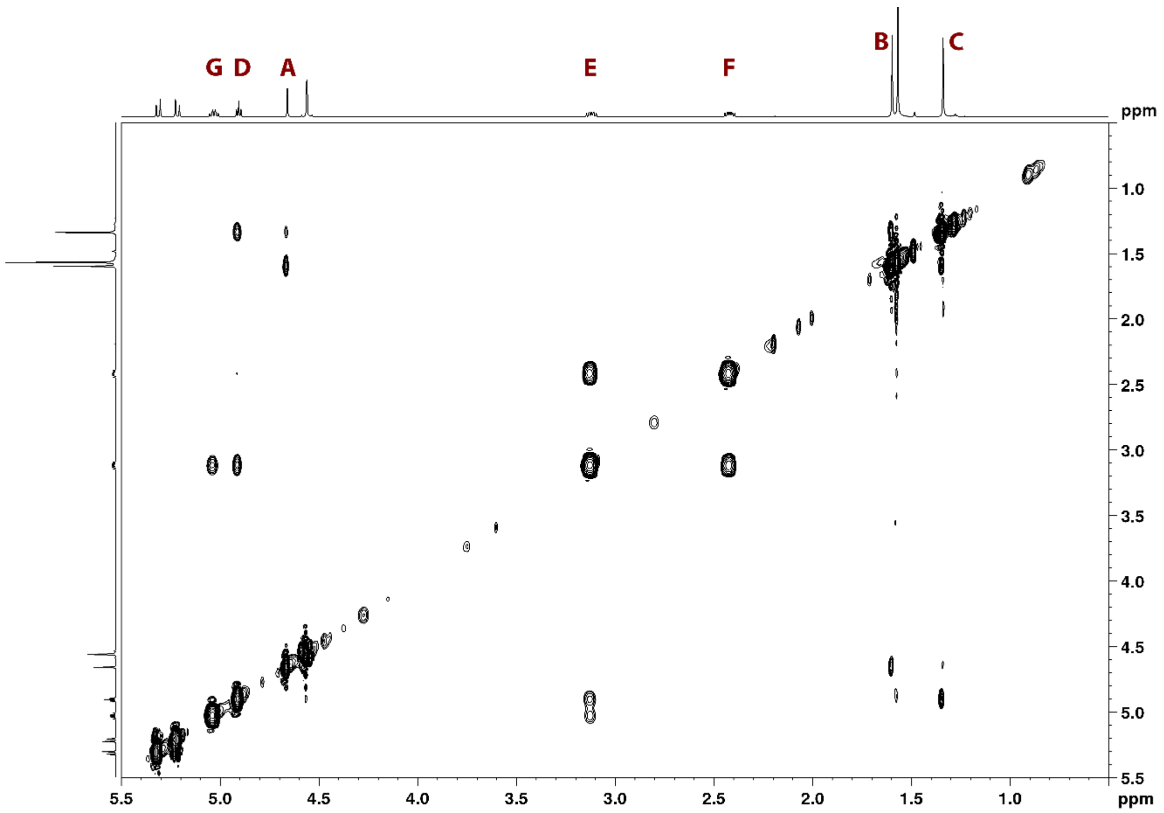

Supporting Figure S4. Representative dose response curves for the penicillin derivatives investigated in this study for M^{pro} inhibition (continues on the following page). The dose response curves are shown as mean of technical duplicates of a representative independent repeat. IC_{50} values shown in Tables 1-4 are a mean of at least two independent repeats ($n \geq 2$, mean ± SD), each composed of technical duplicates. Conditions: SPE-MS M^{pro} inhibition assays were performed using SPE-MS as described in the Experimental Section employing SARS-CoV-2 M^{pro} (0.15 μM) and substrate (2.0 μM).

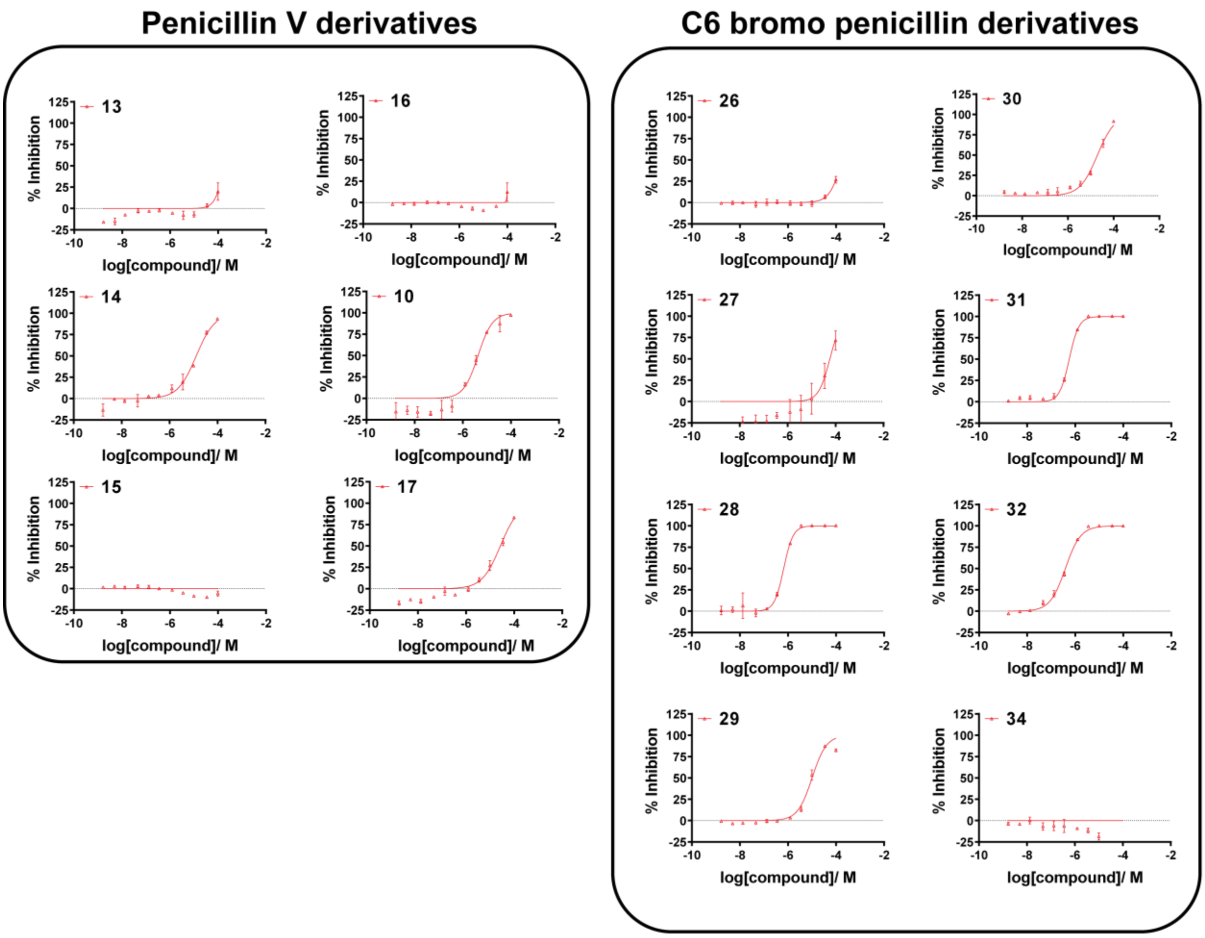

C2 penicillin derivatives

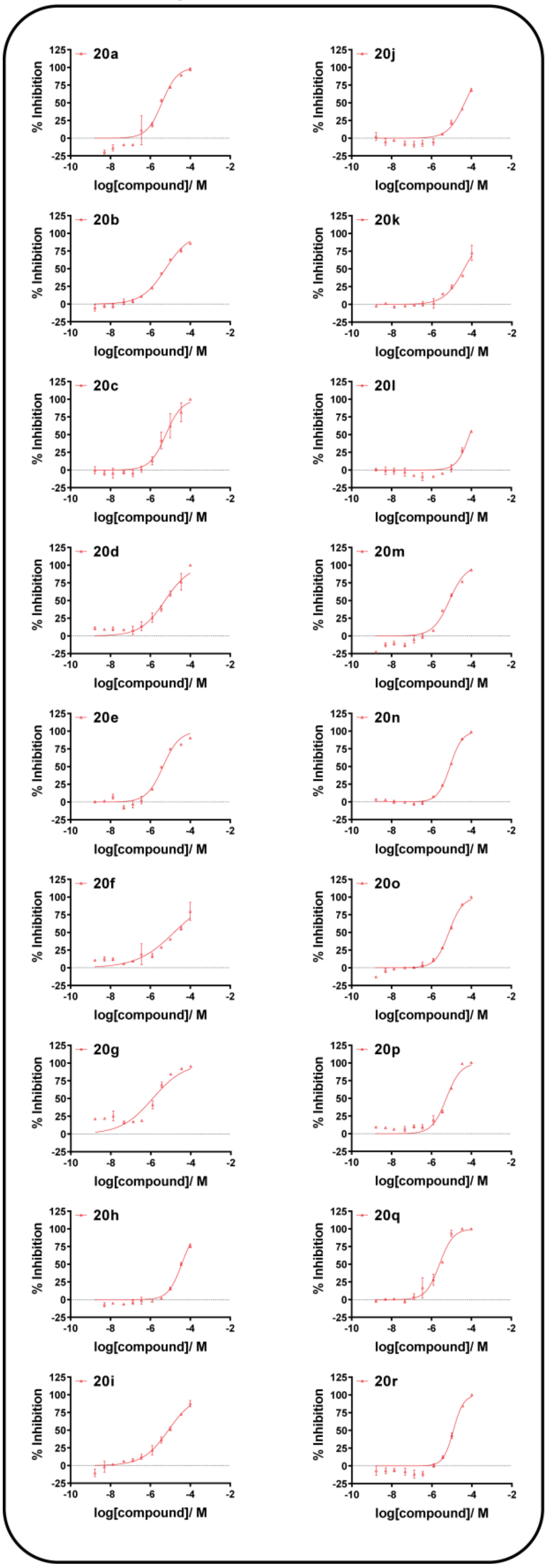

C6 penicillin derivatives

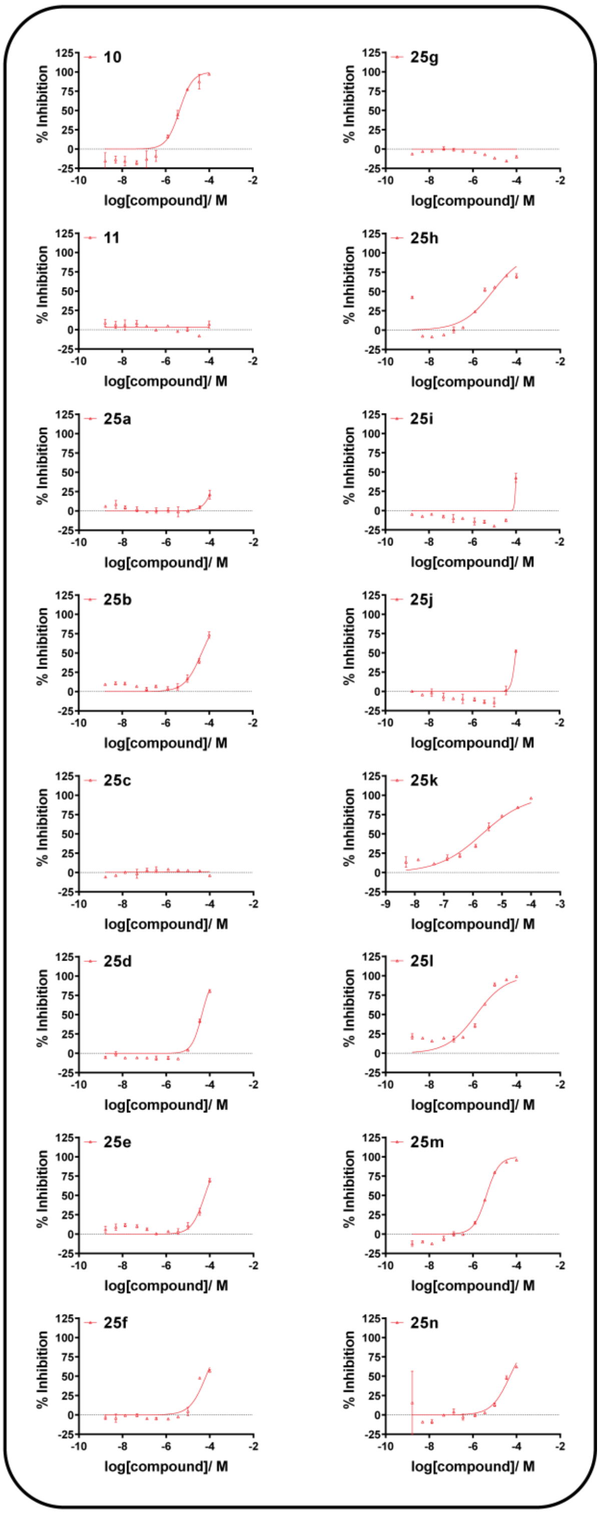

Supporting Figure S5. Penicillin V sulfone ester derivatives inhibit M^{pro} by selective active site cysteine covalent modification. The protein-observed MS studies, which were performed using penicillin V sulfone esters (**A**) **10**, (**B**) **20c**, (**C**) **20q**, (**D**) **20a**, (**E**) **20p**, (**F**) **25b**, (**G**) **25a**, and (**H**) **25m** with and without the active site cysteine (Cys145)-alkylating agent *N*-*para*-toluenesulfonyl-*L*-phenylalanine chloromethyl ketone (TPCK)[8] (10 µM) at two different penicillin concentrations (11 and 33 µM), imply that these penicillin derivatives inhibit M^{pro} selectively by covalent reaction with Cys145. Penicillins **25a** and **25b**, which do not inhibit M^{pro} efficiently (Table 3), apparently do not covalently react with Cys145. SPE-MS assays were performed as described in the Experimental Section employing SARS-CoV-2 M^{pro} (2.0 µM). Data are shown as technical duplicates (a and b).

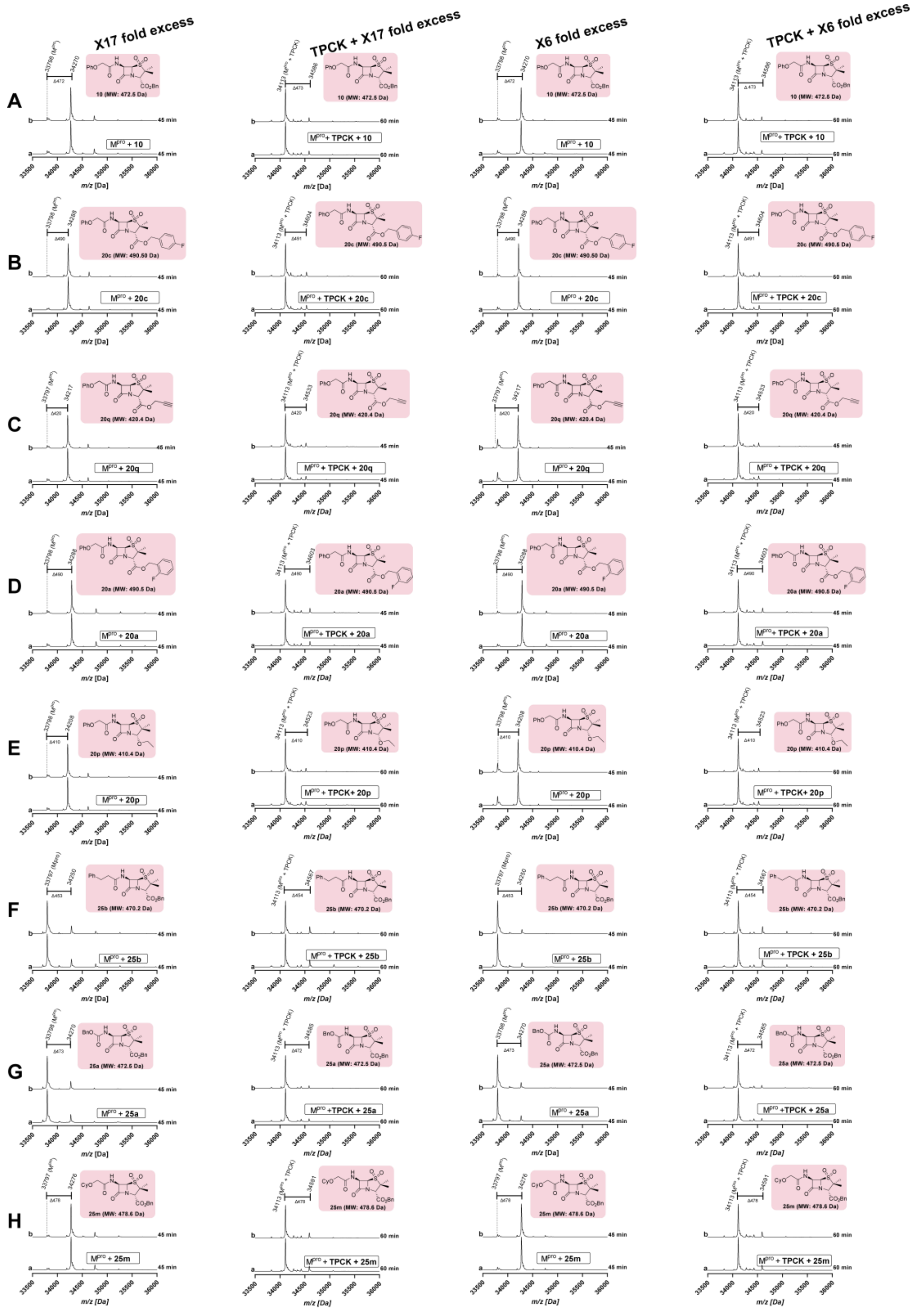

Supporting Figure S6. γ-Lactam sulfone ester 17 does not inhibit M^pro^ by selective active site cysteine covalent modification. (**A**) The γ-lactam sulfone benzyl ester **17**, which inhibits M^pro^ with reduced potency compared to the corresponding penicillin sulfone benzyl ester **10** (IC_{50} ~ 26.1 μM, Table 1, entry 7), does not selectively react with the M^pro^ active site cysteine Cys145, as supported by protein-observed MS studies performed with and without the selective Cys145-alkylating agent *N*-*para*-toluenesulfonyl-*L*-phenylalanine chloromethyl ketone (TPCK)[8] (10 μM) at two different γ-lactam concentrations (11 and 33 μM). The reaction profile of **17** with M^pro^ resembles those of penicillins **25a** and **25b** (Supporting Figure S5F and G), which do not inhibit M^pro^ efficiently (Table 3), but not the one of benzoisothiazolinone (BIT), which non-selectively undergoes covalent reactions with the 12 M^pro^ cysteine residues (**B**). The results suggest that the mode of inhibition of γ-lactam **17** is different from those of the covalently reacting β-lactam analogues (Supporting Figure S5), potentially involving non-covalent binding of M^pro^. SPE-MS assays were performed as described in the Experimental Section employing SARS-CoV-2 M^pro^ (2.0 μM). Data are shown as technical duplicates (a and b).

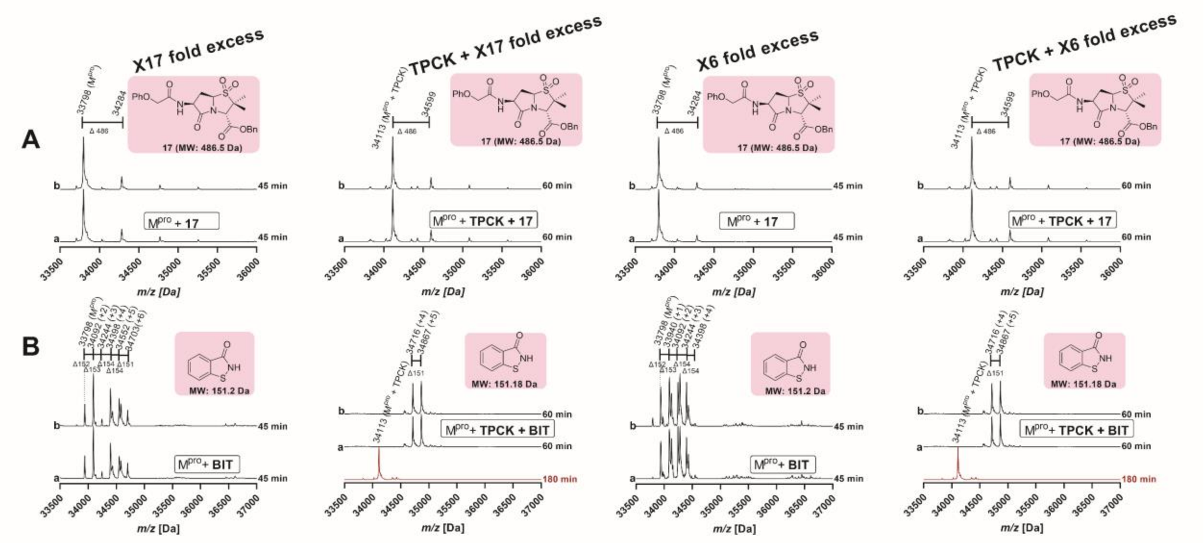

Supporting Figure S7. Crystallographic analysis of the M^pro:20e-derived complex. Color code: M^pro: grey; carbon-backbone of the **20e**-derived complex is in orange; oxygen: red; nitrogen: blue; sulfur: yellow; fluorine: light blue. **A**: Overview of the M^pro:**20e**-derived complex crystal structure. **B**: Superimposition of a view from the M^pro:**20e**-derived complex structure with one from the reported M^pro:PF-07321332 structure (pale green: M^pro; slate blue: carbon-backbone of PF-07321332, nirmatrelvir; PDB ID: 7VH8[9]) reveals similar M^pro conformations (RMSD = 0.41 Å).

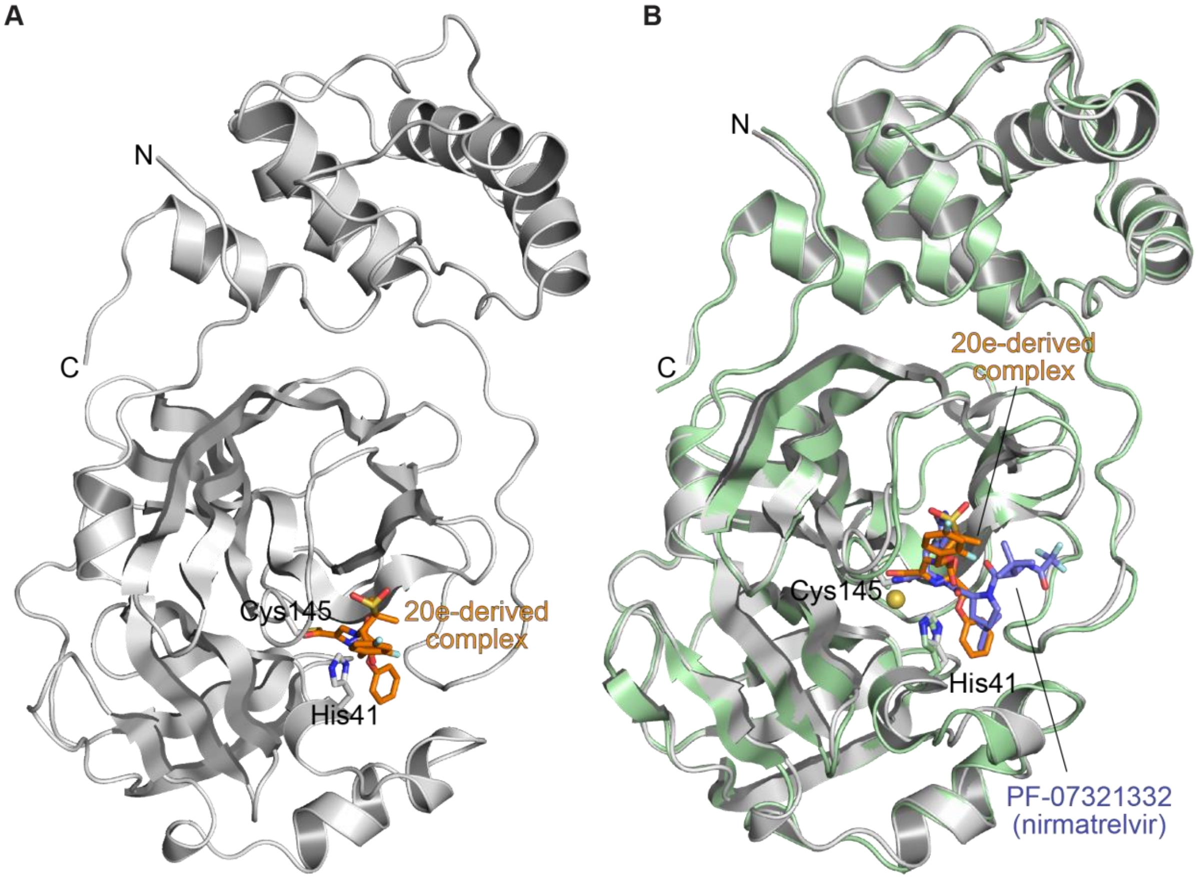

2. Supporting tables

Supporting Table S1. The use of a 37mer rather than an 11mer peptide substrate does not compromise M^{pro} SPE-MS inhibition assays. The comparison of IC_{50}-values for reported M^{pro} inhibitors,[8,10] which were determined using either an 11mer (TSAVLQ/SGFRK-NH_2) or a 37mer peptide (ALNDFSNSGSDVLYQPPQTSITSAVLQ/SGFRKMAFPS-NH_2) as substrate in the SPE-MS M^{pro} inhibition assays, reveals that the IC_{50}-values do not alter substantially depending on the identity of the substrate. However, when using the 37mer substrate, inhibitor-induced suppression of the product peptide ionization at high concentrations of certain inhibitors was minimized resulting in more accurate IC_{50}-determinations.

	**M^{pro} inhibitor**	**$^{a,b)}IC_{50}$ [µM] (11mer peptide)**	**$^{a,c)}IC_{50}$ [µM] (37mer peptide)**
**1**	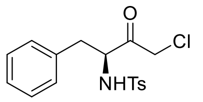 ***N-para*-tosyl-L-phenylalanine chloromethyl ketone (TPCK)**	1.7 ± 0.4	2.5 ± 0.9
**2**	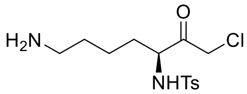 **N_{α}-*para*-tosyl-L-lysine chloromethyl ketone hydrochloride (TLCK)**	2.7 ± 0.2	3.0 ± 0.4
**3**	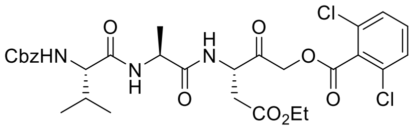 **SDZ-224015 (2)**	0.06 ± 0.01	0.05 ± 0.02
**4**	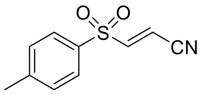 **Bay 11-7082**	3.4 ± 1.1	3.6 ± 1.5
**5**	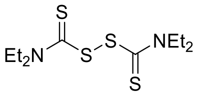 **disulfiram**	0.17 ± 0.01	0.53 ± 0.10
**6**	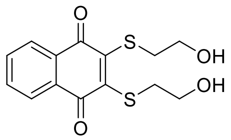 **NSC95397**	0.49 ± 0.03	0.47 ± 0.14
**7**	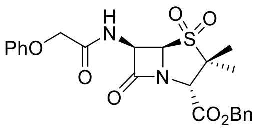 **penicillin V sulfone benzyl ester (10)**	8.7 ± 0.2 (^{d}1.5 ± 0.7)	6.6 ± 2.7

a) M^{pro} inhibition assays were performed using SPE-MS as described in the Experimental Section employing SARS-CoV-2 M^{pro} (0.15 µM) and substrate peptide (2.0 µM) in buffer (20 mM HEPES, pH 7.5, 50 mM NaCl). Data are presented as a mean of technical duplicates (n = 2; mean ± standard deviation, SD); b) using the synthetic 11-mer peptide TSAVLQ/SGFRK-NH_2; c) using the synthetic 37mer peptide ALNDFSNSGSDVLYQPPQTSITSAVLQ/SGFRKMAFPS-NH_2; d) reported IC_{50}-value using the synthetic 11mer peptide TSAVLQ/SGFRK-NH_2. Note, the enzyme-inhibitor mixture was preincubated for 30 min rather than 15 min, likely resulting in more complete cysteine acylation and thus more potent M^{pro} inhibition.[8]

Supporting Table S2. Penicillin benzyl esters and penicillin (*S*)-sulfoxide benzyl esters do not inhibit M^{pro} efficiently (continues on the following page). In general, the investigated penicillin esters and (*S*)-sulfoxide esters do not inhibit M^{pro} efficiently, in agreement with the initial SAR data (Table 1). The results thus confirm the proposed importance of the *pro*-(*R*) sulfone oxygen atom for M^{pro} inhibition. Notable exceptions are penicillin (*S*)-sulfoxide esters **21e**, **21g**, **21n**, **21p**, and **21r** which inhibit M^{pro} with IC$_{50}$s ranging from ~19.5 to ~32.1 µM (Entries 5, 7, 14, 16, and 18); Note, however, that the IC$_{50}$s are higher than those obtained for the corresponding sulfones (Table 1).

	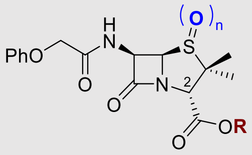		**Compound**	a)**IC$_{50}$** [µM] using **SPE-MS** **(37mer peptide)**
	**R**	**n**		
**1**	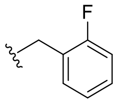	0	**19a**	47.7 ± 6.8
		1	**21a**	49.3 ± 9.9
**2**	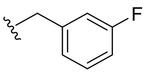	0	**19b**	>50
		1	**21b**	34.1 ± 8.8
**3**	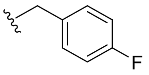	0	**19c**	>50
		1	**21c**	>50
**4**	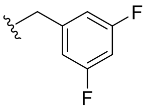	0	**19d**	>50
		1	**21d**	>50
**5**	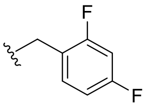	0	**19e**	>50
		1	**21e**	19.5 ± 3.7
**6**	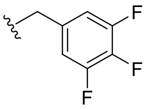	0	**19f**	>50
		1	**21f**	>50
**7**	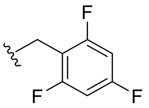	0	**19g**	>50
		1	**21g**	21.6 ± 4.6
**8**	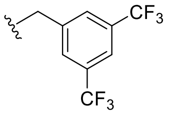	0	**19h**	>50
		1	**21h**	48.3 ± 9.6
**9**	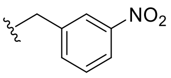	0	**19i**	not determined
		1	**21i**	>50

	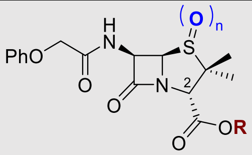 **R**	**n**	**Compound**	[a]**IC_{50}** [µM] using **SPE-MS** **(37mer peptide)**
**10**	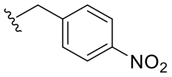	0	**19j**	>50
		1	**21j**	>50
**11**	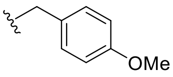	0	**19k**	45.8 ± 11.0
		1	**21k**	44.3 ± 13.7
**12**	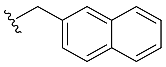	0	**19l**	not determined
		1	**21l**	41.8 ± 5.4
**13**	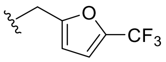	0	**19m**	not determined
		1	**21m**	43.3 ± 19.7
**14**	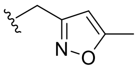	0	**19n**	not determined
		1	**21n**	31.3 ± 12.9
**15**	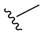	0	**19o**	>50
		1	**21o**	>50
**16**	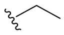	0	**19p**	not determined
		1	**21p**	28.5 ± 15.2
**17**	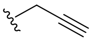	0	**19q**	not determined
		1	**21q**	42.9 ± 5.8
**18**	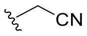	0	**19r**	not determined
		1	**21r**	32.1 ± 13.9

a) M^{pro} inhibition assays were performed using SPE-MS as described in the Experimental Section employing SARS-CoV-2 M^{pro} (0.15 µM) and a 37mer substrate peptide (2.0 µM) (ALNDFSNSGSDVLYQPPQTSITSAVLQ/SGFRKMAFPS-NH_2). Results are a mean of at least two independent runs, each composed of technical duplicates (n ≥ 2; mean ± SD).

Supporting Table S3. Data collection and refinement statistics for the SARS-CoV-2 M^{pro}:20e-derived complex.

	SARS-CoV-2 M^{pro}:**20e**-derived complex
**PDB ID**	7Z59
**Data collection**	
Space group	C2
Cell dimensions	
a, *b*, *c* (Å)	113.3, 53.8, 44.7
a, b, g (°)	90.0, 100.6, 90.0
Resolution (Å)	55.69-2.0 (2.05-2.0)
R_{merge}	0.19 (4.1)
I / s*I*	7.9 (0.7)
CC (1/2)	1.0 (0.2)
Completeness (%)	100.0 (100.0)
Redundancy	10.7 (10.8)
**Refinement**	
Resolution (Å)	55.69-2.0 (2.05-2.0)
No. reflections	18017
R_{work} / R_{free}	0.22 / 0.26
No. atoms	
Protein	2367
Ligand/ion	39
Water	131
B-factors	
Protein	34.69
Ligand/ion	35.74
Water	51.06
R.m.s. deviations	
Bond lengths (Å)	0.008
Bond angles (°)	1.49

*Values in parentheses are for highest-resolution shell.

3. General synthesis information

All reagents were from commercial sources (Sigma-Aldrich, Inc.; Fluorochem Ltd; Tokyo Chemical Industries) and used as received. Penicillin V benzyl ester (**13**),[11] penicillin V 4-nitrobenzyl ester (**19j**),[12] penicillin G benzyl ester,[13] penicillin V (*S*)-sulfoxide benzyl ester (**14**),[11] (6*S*)-penicillin V (*S*)-sulfoxide benzyl ester (**15**),[14] potassium (2*S*,5*R*,6*R*)-3,3-dimethyl-7-oxo-6-(*rac*-2-phenoxypropanamido)-4-thia-1-azabicyclo[3.2.0]heptane-2-carboxylate,[15] 4-benzyl (2*S*,5*R*,6*R*)-3,3-dimethyl-7-oxo-6-(*rac*-2-phenoxypropanamido)-4-thia-1-azabicyclo[3.2.0]heptane-2-carboxylate,[15] (2*S*,5*R*)-3,3-dimethyl-7-oxo-4-thia-1-azabicyclo[3.2.0]heptane-2-carboxylic acid (6,6-dibromopenicillanic acid, **26**),[16] (2*S*,5*R*)-3,3-dimethyl-7-oxo-4-thia-1-azabicyclo[3.2.0]heptane-2-carboxylic acid 4,4-dioxide (6,6-dibromopenicillanic acid *S*,*S*-dioxide, **30**),[16] were synthesized as reported. Anhydrous solvents (Sigma-Aldrich, Inc.) were kept under an atmosphere of nitrogen.
Purifications were performed using a Biotage Isolera One purification machine (wavelength monitored: 254 and 280 nm) equipped with pre-packed Biotage® SNAP KP-Sil, Biotage® SNAP Ultra or Biotage® SFär flash chromatography cartridges. The cartridge type and size as well as solvent gradients (in column volumes, CV) used, are specified in the individual experimental procedures. HPLC grade solvents (Sigma-Aldrich Inc.) were used for purifications, reaction work-ups, and extractions.
Thin layer chromatography (TLC) was carried out using Merck silica gel 60 F_{254} TLC plates and visualized using UV light. Melting points (m.p.) were determined using a Stuart SMP-40 automated melting point apparatus. Optical rotation (α) measurements were performed using a Unipol (Schmidt Haensch) polarimeter operated at 25 °C and using a wavelength (λ) of 589 nm (sodium D-lines). Infrared (IR) spectroscopy was performed using a Bruker Tensor-27 Fourier transform infrared (FT-IR) spectrometer. High-resolution mass spectrometry (HRMS) was performed using electrospray ionization (ESI) mass spectrometry (MS) in the positive or negative ionization mode employing a Thermo Scientific Exactive mass spectrometer (ThermoFisher Scientific); data are presented as a mass-to-charge ratio (*m/z*).
Nuclear magnetic resonance (NMR) spectroscopy was performed using a Bruker AVANCE AVIIIHD 600 machine equipped with a 5 mm BB-F/1H Prodigy N_2 cryoprobe. Chemical shifts for protons are reported in parts per million (ppm) downfield from tetramethylsilane and are referenced to residual protium in the NMR solvent ($CDCl_3$: δ = 7.28 ppm). For ^{13}C NMR, chemical shifts are reported in the scale relative to the NMR solvent ($CDCl_3$: δ = 77.00 ppm). For ^{19}F NMR, chemical shifts are reported in the scale relative to $CFCl_3$. NMR data are reported as follows: chemical shift, multiplicity (s: singlet, d: doublet, dd: doublet of doublets, t: triplet, q: quartet, m: multiplet, br: broad signal), coupling constant (*J*, Hz; accurate to 0.1 Hz), and integration. The number of C-atoms in brackets indicates overlapping signals in ^{13}C NMR; chemical shift numbers in brackets indicate close signals that can be differentiated taking into account the second decimal numbers. All compounds are >95% pure by NMR and HPLC analysis unless stated otherwise, NMR spectra are shown in Section 7 of the Supporting Information and HPLC traces are shown in Section 8 of the Supporting Information.

4. General synthetic procedures

General Procedure A. To a solution of commercially-sourced penicillin V potassium salt (**18**, 1.0 equiv.) in anhydrous DMF (0.6 M) was added a commercially-sourced alkyl bromide (1.15 equiv.) at ambient temperature under an ambient atmosphere. The reaction mixture was stirred for 2 h, then poured onto ice water (20 mL/mmol). The mixture was extracted with diethyl ether. The organic phase was washed twice with saturated aqueous $NaHCO_3$ solution, dried over anhydrous Na_2SO_4, filtered, evaporated, and purified by column chromatography to afford the corresponding penicillin V esters.

General Procedure B. To a solution of a penicillin V ester (1.0 equiv.) in anhydrous dichloromethane (0.1 M) was added commercially-sourced *meta*-chlorperbenzoic acid (mCPBA, 2.2 equiv.) under an ambient atmosphere at 0 °C. The reaction mixture was stirred at ambient temperature overnight (14 – 18 h) and was then washed sequentially with 10%$_{w/v}$ aqueous sodium metabisulfite solution, saturated aqueous $NaHCO_3$ solution, and brine. The organic phase was dried over anhydrous Na_2SO_4, filtered, evaporated, and purified by column chromatography to afford the corresponding pure penicillin sulfone and sulfoxide esters. Note that most of the penicillin sulfone and sulfoxides were isolated as oils or amorphous solids though some have been described to be solids. This is likely due to the comparably small scale of the reaction, which did not allow to perform triturations or recrystallizations efficiently.

General Procedure C. To a solution of (+)-6-aminopenicillanic acid benzyl ester **23** (1.0 equiv.) in anhydrous DMF (0.125 M) was added sequentially a commercially-sourced carboxylic acid (1.2 equiv.), Hünig's base (2.0 equiv.), and (1-cyano-2-ethoxy-2-oxoethylidenaminooxy)dimethylamino-morpholino-carbenium hexafluorophosphate (COMU)[17] (1.1 equiv.) at 0 °C under an atmosphere of nitrogen gas. The reaction mixture was slowly warmed to ambient temperature while stirring overnight (~16 h) under an atmosphere of nitrogen gas. The reaction mixture was then diluted with ethyl acetate and washed twice with aqueous HCl solution (1.0 M), twice with saturated aqueous $NaHCO_3$ solution, and finally with brine. The organic phase was dried over anhydrous Na_2SO_4, filtered, evaporated, and purified by column chromatography to afford the corresponding (+)-6-aminopenicillanic carboxamide benzyl ester.

5. Experimental procedures and compound characterizations

4-Benzyl (2*S*,5*R*,6*R*)-3,3-dimethyl-7-oxo-6-(2-phenoxyacetamido)-4-thia-1-azabicyclo[3.2.0]heptane-2-carboxylate 4,4-dioxide (10). According to General Procedure B, penicillin sulfone ester **10** (80 mg, 34%) was obtained from penicillin V benzyl ester (**13**)[11] (200 mg, 0.5 mmol), following column chromatography (10 g KP-Sil cartridge; 36 mL/min; initially, 100%$_{v/v}$ cyclohexane (3 CV), followed by a linear gradient (25 CV): 0%$_{v/v}$ → 100%$_{v/v}$ ethyl acetate in cyclohexane). The analytical data are in agreement with those reported.[18]

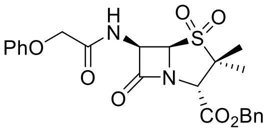

White amorphous solid; ^{1}H NMR (600 MHz, 300 K, $CDCl_3$): δ = 8.17 (d, J = 10.7 Hz, 1H), 7.45−7.38 (m, 5H), 7.34−7.31 (m, 2H), 7.05−7.03 (m, 1H), 6.95−6.94 (m, 2H), 6.19 (dd, J = 10.7 Hz, 1H), 5.32 (d, J = 11.9 Hz, 1H), 5.21 (d, J = 11.9 Hz, 1H), 4.79 (d, J = 4.6 Hz, 1H), 4.60−4.54 (m, 3H), 1.58 (s, 3H), 1.28 ppm (s, 3H); ^{13}C NMR (150 MHz, 300 K, $CDCl_3$): δ = 173.5, 168.3, 166.4, 156.8, 134.2, 129.7, 129.1, 128.9(3), 128.9, 122.3, 114.9, 68.4, 67.0, 65.7, 64.8, 63.9, 56.2, 20.0, 17.9 ppm; IR (film): ṽ = 3397, 3033, 2975, 1806, 1756, 1699, 1599, 1520, 1495, 1458, 1322, 1289, 1213, 1171, 1116, 1065 cm^{-1}; HRMS (ESI): *m/z* calculated for $C_{23}H_{23}O_7N_2S$ [M−H]$^-$: 471.1231, found: 471.1227; $[\alpha]_D^{25}$ = +109.0 (c = 1.2, $CHCl_3$).

4-Benzyl (2*S*,5*R*,6*R*)-3,3-dimethyl-7-oxo-6-(2-phenylacetamido)-4-thia-1-azabicyclo[3.2.0]heptane-2-carboxylate 4,4-dioxide (11).[19] According to General Procedure B, penicillin G sulfone **11** (40 mg, 34%) was obtained from penicillin G benzyl ester[13] (212 mg, 0.5 mmol), following column chromatography (10 g KP-Sil cartridge; 36 mL/min; initially, 100% cyclohexane (3 CV), followed by a linear gradient (25 CV): 0%→100% ethyl acetate in cyclohexane). White amorphous solid; ^{1}H NMR (600 MHz, 300 K, $CDCl_3$): δ = 7.42−7.37 (m, 7H), 7.33−7.31 (m, 1H), 7.27−7.26 (m, 2H), 6.85 (d, J = 10.4 Hz, 1H), 6.13 (dd, J = 10.4, 4.6 Hz, 1H), 5.29 (d, J = 12.0 Hz, 1H), 5.18 (d, J = 12.0 Hz, 1H), 4.71 (d, J = 4.6 Hz, 1H), 3.66 (d, J = 16.0 Hz, 1H), 3.63 (d, J = 16.0 Hz, 1H), 4.46 (s, 1H), 1.51 (s, 3H), 1.24 ppm (s, 3H); ^{13}C NMR (150 MHz, 300 K, $CDCl_3$): δ = 173.9, 170.7, 166.4, 134.2, 133.2, 129.4 (2C), 129.1 (3C), 128.9 (2C), 128.8(7) (2C), 127.7, 68.3, 65.7, 64.7, 63.8, 57.0,

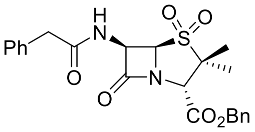

43.3, 20.0, 17.8 ppm; IR (film): ṽ = 3379, 3064, 3032, 2979, 1803, 1755, 1685, 1512, 1497, 1456, 1377, 1321, 1285, 1211, 1170, 1117, 1067, 1029 cm^{-1}; HRMS (ESI): *m/z* calculated for $C_{23}H_{24}O_6N_2SNa$ $[M+Na]^+$: 479.1247, found: 479.1245; $[\alpha]_D^{25}$ = +104.5 (c = 0.2, $CHCl_3$).

4-Benzyl (2*S*,5*R*,6*S*)-3,3-dimethyl-7-oxo-6-(2-phenoxyacetamido)-4-thia-1-azabicyclo[3.2.0]heptane-2-carboxylate 4,4-dioxide (16). To a solution of (6*S*)-penicillin V (*S*)-sulfoxide benzyl ester (**15**)[14] (127 mg, 0.28 mmol) in aqueous acetic acid (9.2 mL, 4:1$_{v/v}$, acetic acid:water) was added a solution of potassium permanganate (47 mg, 0.2 mmol, 0.7 equiv.) in water (3.0 mL) at 0 °C under an ambient atmosphere dropwise over 1 h. After completion of the addition, the reaction mixture was stirred for 90 min at 0 °C, then aqueous H_2O_2 (30%$_{w/v}$) was added. The mixture was extracted three times with dichloromethane. The combined organic extracts were sequentially washed with saturated aqueous $NaHCO_3$ solution and brine, dried over anhydrous Na_2SO_4, filtered, evaporated, then purified by column chromatography (10 g Sfär cartridge; 40 mL/min; initially, 100% cyclohexane (3 CV), followed by a linear gradient (15 CV): 0%→100% ethyl acetate in cyclohexane) to afford C6-epi penicillin sulfone **16** (125 mg, 95%). White amorphous solid; ^{1}H NMR (600 MHz, 300 K, $CDCl_3$): δ = 7.41−7.35 (m, 8H), 7.09−7.07 (m, 1H), 6.95 (d, *J* = 8.6 Hz, 2H), 5.45 (dd, *J* = 8.3, 1.3 Hz, 1H), 5.33 (d, *J* = 12.0 Hz, 1H), 5.21 (d, *J* = 12.0 Hz, 1H), 4.79 (s, 1H), 4.58 (s, 2H), 4.46 (s, 1H), 1.57 (s, 3H), 1.29 ppm (s, 3H); ^{13}C NMR (150 MHz, 300 K, $CDCl_3$): δ = 168.8, 168.3, 166.3, 156.8, 134.3, 129.9, 129.0, 128.8, 128.7(8), 122.6, 114.7, 68.5, 68.3, 67.0, 63.4, 62.8, 58.2, 19.8, 18.6 ppm; IR (film): ṽ = 3385, 3063, 3037, 2978, 2942, 2917, 1796, 1753, 1690, 1599, 1525, 1495, 1457, 1441, 1317, 1215, 1179, 1157, 1116, 1084, 1064 cm^{-1}; HRMS (ESI): *m/z* calculated for $C_{23}H_{25}O_7N_2S$ $[M+H]^+$: 473.1377, found: 473.1376; $[\alpha]_D^{25}$ = +136.0 (c = 1.0, $CHCl_3$).

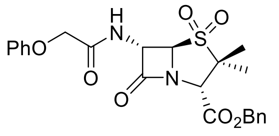

Benzyl (3*S*,6*S*,7a*R*)-6-(((benzyloxy)carbonyl)amino)-2,2-dimethyl-5-oxohexahydropyrrolo[2,1-*b*]thiazole-3-carboxylate (39). To a suspension of Cbz-L-homoserine benzyl ester **35**[4] (3.49 g, 10.0 mmol, 1.0 equiv.) and $NaHCO_3$ (3.36 g, 40.0 mmol, 4.0 equiv.) in dichloromethane (HPLC grade, 150 mL) was slowly added Dess-Martin periodinane[5] (8.62 g, 20.0 mmol, 2.0 equiv.) at 0 °C under an ambient atmosphere. The reaction mixture was stirred for 5 min at 0 °C and for 2 h at ambient temperature before being poured into an ice-cold aqueous sodium phosphate buffer solution (pH = 7) containing $Na_2S_2O_3$•$5H_2O$ (12 g). The mixture was stirred for 30 min at ambient temperature and then extracted three times with dichloromethane. The combined organic extracts were washed three times with saturated aqueous $NaHCO_3$ solution, dried over anhydrous Na_2SO_4, filtered, evaporated, then purified by column chromatography (50 g Sfär cartridge; 115 mL/min; initially, 100% cyclohexane (3 CV), followed by a linear gradient (15 CV): 0%→30% ethyl acetate in cyclohexane) to afford aldehyde **36** (3.0 g, 88%),[6] which was directly used in the following reaction.

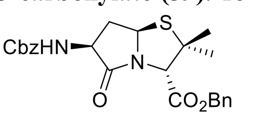

To a solution of aldehyde **36** (2.64 g, 7.72 mmol, 1.0 equiv.) in anhydrous pyridine (10 mL) was added D-penicillamine **37** (1.39 g, 9.28 mmol, 1.2 equiv.) under ambient atmosphere and temperature. The reaction mixture was stirred for 5 h at ambient temperature and then for 12 h at 120 °C. The reaction mixture was cooled to ambient temperature and the pyridine was evaporated. Ethyl acetate was added to the residue, the resultant mixture was washed three times with aqueous HCl solution (1 M); the aqueous phase was discarded. The remaining organic phase was washed three time with aqueous Na_2CO_3 solution (1 M); the organic phase was discarded. The combined basic aqueous extracts were carefully acidified to pH = 2 at 0 °C by the dropwise addition of an aqueous HCl solution (4 M) and were then extracted five times with ethyl acetate. The combined organic extracts were washed twice with brine, dried over anhydrous Na_2SO_4, filtered, and evaporated to afford analytically pure γ-lactam **38** (2.5 g, 87%) which was used in the following reaction without further purification.

To a solution of γ-lactam **38** (3.0 g, 8.34 mmol, 1.0 equiv.), $NaHCO_3$ (3.5 g, 41.7 mmol, 5.0 equiv.), and NaI (125 mg, 0.83 mmol, 0.1 equiv.) in anhydrous DMF (30 mL) was added dropwise benzyl bromide (1.09 mL, 9.17

mmol, 1.1 equiv.) under an atmosphere of N_2 gas at ambient temperature. The reaction mixture was stirred for 18 h at ambient temperature, then was diluted with water and extracted three times with ethyl acetate. The organic phase was washed twice with saturated aqueous $NaHCO_3$ solution, then with water and brine. It was dried over anhydrous Na_2SO_4, filtered, evaporated, and purified by column chromatography (50 g Sfär cartridge; 115 mL/min; initially, 100% cyclohexane (3 CV), followed by a linear gradient (15 CV): 0%→35% ethyl acetate in cyclohexane) to afford γ-lactam benzyl ester **39** (2.7 g, 70%). Clear colorless oil; ^{1}H NMR (600 MHz, 300 K, $CDCl_3$): δ = 7.41−7.33 (m, 10H), 5.44−5.40 (m, 2H), 5.23 (d, J = 12.1 Hz, 1H), 5.19 (d, J = 12.1 Hz, 1H), 5.14 (s, 2H), 4.68−4.64 (m, 2H), 3.22−3.18 (m, 1H), 2.13−2.06 (m, 1H), 1.57 (s, 3H), 1.41 ppm (s, 3H); ^{13}C NMR (150 MHz, 300 K, $CDCl_3$): δ = 170.8, 167.8, 155.9, 136.0, 134.8, 128.7, 128.6, 128.5(9), 128.5, 128.2, 128.1, 67.4, 67.3, 67.1, 62.3, 57.8, 54.5, 39.2, 31.3, 25.9 ppm; IR (film): ṽ = 3322, 3065, 3034, 2968, 2935, 1703, 1586, 1532, 1499, 1455, 1402, 1343, 1278, 1248, 1218, 1185, 1156, 1060, 1028, 1004 cm^{-1}; HRMS (ESI): m/z calculated for $C_{24}H_{26}O_5N_2SNa$ [M+Na]$^+$: 477.1455, found: 477.1455; $[\alpha]_D^{25}$ = +96.0 (c = 1.2, $CHCl_3$).

Benzyl (3*S*,6*S*,7a*R*)-6-amino-2,2-dimethyl-5-oxohexahydropyrrolo[2,1-*b*]thiazole-3-carboxylate (40). To a

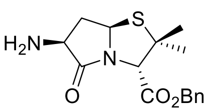

solution of γ-lactam **39** (796 mg, 1.75 mmol, 1.0 equiv.) in anhydrous dichloromethane (20 mL) was added 33%$_{v/v}$ HBr in AcOH (3.0 mL) at 0 °C under an ambient atmosphere. The reaction mixture was stirred for 2 h at 0 °C before it was diluted with dichloromethane. The mixture was extracted twice with water and one time with aqueous HCl solution (1 M), the organic phase was discarded. The combined aqueous extracts were carefully basified with $NaHCO_3$ (s) to pH = 8-9 at 0 °C, then extracted three times with dichloromethane. The combined organic extracts were dried over anhydrous Na_2SO_4, filtered, evaporated, and purified by column chromatography (10 g Sfär cartridge; 36 mL/min; initially, 100% dichloromethane (3 CV), followed by a linear gradient (20 CV): 0%→30% diluted methanol (9:1$_{v/v}$, dichloromethane in methanol) in dichloromethane) to afford γ-lactam **40** (496 mg, 89%). Clear yellow oil; ^{1}H NMR (600 MHz, 300 K, $CDCl_3$): δ = 7.41−7.35 (m, 5H), 5.39 (dd, J = 7.8, 6.2 Hz, 1H), 5.23 (d, J = 12.2 Hz, 1H), 5.19 (d, J = 12.2 Hz, 1H), 4.62 (s, 1H), 3.94 (dd, J = 11.1, 8.0 Hz, 1H), 3.00 (ddd, J = 12.8, 7.6, 6.0 Hz, 1H), 1.93−1.88 (m, 1H), 1.64 (s, 3H), 1.45 ppm (s, 3H); ^{13}C NMR (150 MHz, 300 K, $CDCl_3$): δ = 175.0, 168.1, 134.9, 128.7 (2C), 128.6 (3C), 67.4, 67.3, 62.1, 57.9, 55.2, 40.1, 31.3, 26.0 ppm; IR (film): ṽ = 3368, 3304, 3033, 2970, 2931, 1743, 1705, 1498, 1455, 1402, 1372, 1344, 1267, 1214, 1177, 1136, 1029 cm^{-1}; HRMS (ESI): m/z calculated for $C_{16}H_{21}O_3N_2S$ [M+H]$^+$: 321.1267, found: 321.1268; $[\alpha]_D^{25}$ = +144.0 (c = 1.1, $CHCl_3$).

Benzyl (3*S*,6*S*,7a*R*)-2,2-dimethyl-5-oxo-6-(2-phenoxyacetamido)hexahydropyrrolo[2,1-*b*]thiazole-3-carboxylate (41).

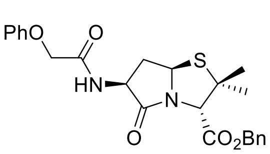

To a solution of γ-lactam **40** (150 mg, 0.47 mmol, 1.0 equiv.) in anhydrous dichloromethane (5.0 mL) were added sequentially commercially-sourced phenoxyacetyl chloride (71 µL, 0.52 mmol, 1.1 equiv.) and triethylamine (71 µL, 0.52 mmol, 2.0 equiv.) under an ambient atmosphere and temperature. The reaction mixture was stirred for 2 h at ambient temperature then diluted with dichloromethane. The mixture was washed twice with water and one time with saturated aqueous $NaHCO_3$ solution, the combined aqueous phases were extracted with dichloromethane. The combined organic phases were dried over anhydrous Na_2SO_4, filtered, evaporated, then purified by column chromatography (10 g Sfär cartridge; 36 mL/min; initially, 100% cyclohexane (3 CV), followed by a linear gradient (17 CV): 0%→45% ethyl acetate in cyclohexane) to afford γ-lactam **41** (143 mg, 67%). Clear pale yellow oil; ^{1}H NMR (600 MHz, 300 K, $CDCl_3$): δ = 7.41−7.32 (m, 7H), 7.19 (d, J = 6.0 Hz, 1H), 7.05 (t, J = 7.4 Hz, 1H), 6.96 (d, J = 8.0 Hz, 2H), 5.45 (dd, J = 8.2, 6.0 Hz, 1H), 5.24 (d, J = 12.1 Hz, 1H), 5.20 (d, J = 12.1 Hz, 1H), 4.88 (ddd, J = 11.2, 7.7, 6.5 Hz, 1H), 4.65 (s, 1H), 4.55 (s, 2H), 3.25 (ddd, J = 12.6, 7.8, 5.9 Hz, 1H), 2.15−2.10 (m, 1H), 1.61 (s, 3H), 1.43 ppm (s, 3H); ^{13}C NMR (150 MHz, 300 K, $CDCl_3$): δ = 170.5, 168.7, 167.8, 157.1, 134.8, 129.8, 128.7, 128.6(7), 128.6, 122.2, 114.8, 67.5, 67.3, 67.2, 62.4, 58.1, 53.0, 38.6, 31.2, 26.0 ppm; IR (film): ṽ = 3297, 3066, 3036, 2980, 2933, 1716, 1674, 1599, 1589, 1533,

1495, 1456, 1404, 1339, 1269, 1241, 1215, 1176, 1136, 1124, 1083, 1063, 963 cm^{-1}; HRMS (ESI): *m/z* calculated for $C_{24}H_{26}O_5N_2SNa$ $[M+Na]^+$: 477.1455, found: 477.1456; $[\alpha]_D^{25}$ = +121.0 (c = 0.9, $CHCl_3$).

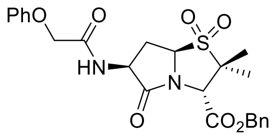

Benzyl (3*S*,6*S*,7a*R*)-2,2-dimethyl-5-oxo-6-(2-phenoxyacetamido)hexahydropyrrolo[2,1-*b*]thiazole-3-carboxylate 1,1-dioxide (17). To a solution of γ-lactam **41** (40 mg, 0.09 mmol, 1.0 equiv.) in anhydrous dichloromethane (5.0 mL) was added mCPBA (23.3 mg, 0.14 mmol, 1.5 equiv.) under an ambient atmosphere and temperature. The reaction mixture was stirred for 18 h at ambient temperature before it was diluted with dichloromethane. The mixture was washed twice with saturated aqueous $NaHCO_3$ solution and once with both water and brine. The combined aqueous phases were extracted with dichloromethane. The combined organic extracts were dried over anhydrous Na_2SO_4, filtered, evaporated, and purified by column chromatography (5 g Sfär cartridge; 18 mL/min; initially, 100% cyclohexane (3 CV), followed by a linear gradient (12 CV): 0%→100% ethyl acetate in cyclohexane) to afford γ-lactam **17** (24 mg, 56%). Clear colorless oil; ^{1}H NMR (600 MHz, 300 K, $CDCl_3$): δ = 7.43−7.39 (m, 5H), 7.34 (t, J = 7.8 Hz, 2H), 7.17 (brd, J = 7.0 Hz, 1H), 7.05 (t, J = 7.4 Hz, 1H), 6.96 (d, J = 8.5 Hz, 2H), 5.31 (d, J = 12.0 Hz, 1H), 5.22 (d, J = 12.0 Hz, 1H), 5.05−5.01 (m, 1H), 4.91 (t, J = 6.9 Hz, 1H), 4.66 (s, 1H), 4.59−4.53 (m, 2H), 3.12 (ddd, J = 13.6, 8.4, 7.4 Hz, 1H), 2.42 (ddd, J = 13.6, 9.8, 6.8 Hz, 1H), 1.60 (s, 3H), 1.34 ppm (s, 3H); ^{13}C NMR (150 MHz, 300 K, $CDCl_3$): δ = 172.4, 168.7, 166.4, 157.0, 134.3, 129.8 (2C), 129.1, 128.9 (4C), 122.3, 114.8 (2C), 68.3, 67.2, 67.1, 62.5, 61.4, 50.7, 27.0, 20.2, 20.1 ppm; IR (film): ṽ = 3339, 3066, 3035, 2980, 2948, 1732, 1677, 1600, 1532, 1495, 1457, 1383, 1324, 1292, 1267, 1243, 1213, 1188, 1175, 1144, 1121, 1110 cm^{-1}; HRMS (ESI): *m/z* calculated for $C_{24}H_{26}O_7N_2SNa$ $[M+Na]^+$: 509.1353, found: 509.1352; $[\alpha]_D^{25}$ = +118.0 (c = 1.0, $CHCl_3$).

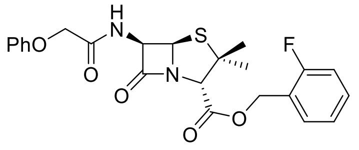

2-Fluorobenzyl (2*S*,5*R*,6*R*)-3,3-dimethyl-7-oxo-6-(2-phenoxyacetamido)-4-thia-1-azabicyclo[3.2.0]heptane-2-carboxylate (19a). According to General Procedure A, penicillin V ester **19a** (602 mg, 94%) was obtained from commercially sourced 2-fluorobenzylbromide and penicillin V potassium salt (540 mg, 1.4 mmol), following column chromatography (10 g Sfär cartridge; 40 mL/min; initially, 100% cyclohexane (3 CV), followed by a linear gradient (20 CV): 0%→100% ethyl acetate in cyclohexane). Clear colorless oil; ^{1}H NMR (600 MHz, 300 K, $CDCl_3$): δ = 7.43−7.37 (m, 2H), 7.36−7.33 (m, 3H), 7.20−7.17 (m, 1H), 7.14−7.10 (m, 1H), 7.07−7.04 (m, 1H), 6.95−6.94 (m, 2H), 5.76 (dd, J = 9.2, 4.3 Hz, 1H), 5.60 (d, J = 4.3 Hz, 1H), 5.30 (d, J = 12.2 Hz, 1H), 5.25 (d, J = 12.2 Hz, 1H), 4.59 (d, J = 15.1 Hz, 1H), 4.55 (d, J = 15.1 Hz, 1H), 4.50 (s, 1H), 1.59 (s, 3H), 1.45 ppm (s, 3H); ^{19}F NMR (565 MHz, 300 K, $CDCl_3$): δ = −117.5 ppm (m, 1F); ^{13}C NMR (150 MHz, 300 K, $CDCl_3$): δ = 173.0, 167.8, 167.3, 161.2 (d, J = 249.0 Hz), 156.9, 131.3 (d, J = 3.3 Hz), 131.0 (d, J = 8.5 Hz), 129.8, 124.3 (d, J = 3.4 Hz), 122.4, 121.9 (d, J = 15.1 Hz), 115.7 (d, J = 21.0 Hz), 114.8, 70.4, 67.8, 67.2, 64.9, 61.5 (d, J = 3.6 Hz), 58.1, 31.8, 26.7 ppm; IR (film): ṽ = 3373, 3066, 3044, 2970, 2933, 1785, 1747, 1694, 1619, 1599, 1590, 1518, 1494, 1457, 1373, 1297, 1237, 1205, 1180, 1155, 1130, 1082, 1061, 1027, 963 cm^{-1}; HRMS (ESI): *m/z* calculated for $C_{23}H_{23}O_5N_2FSNa$ $[M+Na]^+$: 481.1204, found: 481.1204; $[\alpha]_D^{25}$ = +118.3 (c = 0.1, $CHCl_3$).

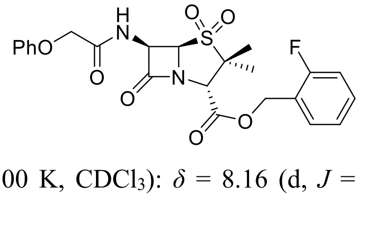

2-Fluorobenzyl (2*S*,5*R*,6*R*)-3,3-dimethyl-7-oxo-6-(2-phenoxyacetamido)-4-thia-1-azabicyclo[3.2.0]heptane-2-carboxylate 4,4-dioxide (20a). According to General Procedure B, penicillin V sulfone **20a** (329 mg, 56%) was obtained from penicillin V ester **19a** (550 mg, 1.2 mmol), following column chromatography (10 g Sfär cartridge; 40 mL/min; initially, 100% cyclohexane (3 CV), followed by a linear gradient (20 CV): 0%→100% ethyl acetate in cyclohexane). White amorphous solid; ^{1}H NMR (600 MHz, 300 K, $CDCl_3$): δ = 8.16 (d, J = 10.6 Hz, 1H), 7.45−7.40 (m, 2H), 7.34−7.31 (m, 2H), 7.22−7.19 (m, 1H),

7.16−7.13 (m, 1H), 7.06−7.03 (m, 1H), 6.95−6.93 (m, 2H), 6.19 (dd, J = 10.7, 4.6 Hz, 1H), 5.38 (d, J = 12.0 Hz, 1H), 5.24 (d, J = 12.0 Hz, 1H), 4.80 (d, J = 4.6 Hz, 1H), 4.60−4.55 (m, 3H), 1.59 (s, 3H), 1.33 ppm (s, 3H); ^{19}F NMR (565 MHz, 300 K, $CDCl_3$): δ = −117.4 ppm (m, 1F); ^{13}C NMR (150 MHz, 300 K, $CDCl_3$): δ = 173.5, 168.3, 166.3, 161.3 (d, J = 249.7 Hz), 156.8, 131.5 (d, J = 3.3 Hz), 131.4 (d, J = 8.5 Hz), 129.7, 124.5 (d, J = 3.9 Hz), 122.3, 121.4 (d, J = 15.0 Hz), 115.8 (d, J = 20.9 Hz), 114.9, 67.0, 65.7, 64.8, 63.9, 62.5 (d, J = 3.4 Hz), 56.2, 20.0, 17.8 ppm; IR (film): $\tilde{\nu}$ = 3406, 2980, 1806, 1758, 1699, 1619, 1599, 1520, 1495, 1458, 1376, 1322, 1289, 1237, 1210, 1170, 1116, 1065, 955 cm^{-1}; HRMS (ESI): m/z calculated for $C_{23}H_{24}O_7N_2FS$ [M+H]$^+$: 491.1283, found: 491.1283; $[\alpha]_D^{25}$ = +89.1 (c = 1.15, $CHCl_3$).

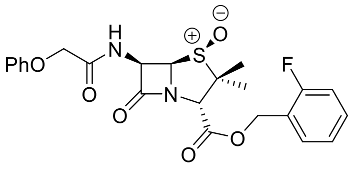

In addition to the penicillin V sulfone **20a**, the corresponding penicillin V sulfoxide **21a** (153 mg, 27%) was isolated in purified form as a single sulfoxide diastereomer. Note, the sulfoxide was tentatively assigned the (S)-configuration based on literature reports on peracid (including mCPBA)-mediated penicillin ester oxidations to sulfoxides.[11,20-21] Clear colorless oil; ^{1}H NMR (600 MHz, 300 K, $CDCl_3$): δ = 8.26 (d, J = 10.5 Hz, 1H), 7.44−7.39 (m, 2H), 7.33−7.30 (m, 2H), 7.20 (t, J = 7.5 Hz, 1H), 7.13 (t, J = 9.1 Hz, 1H), 7.04−7.01 (m, 1H), 6.95 (d, J = 8.6 Hz, 2H), 6.12 (dd, J = 10.4, 4.6 Hz, 1H), 5.39 (d, J = 12.0 Hz, 1H), 5.23 (d, J = 12.0 Hz, 1H), 5.04 (dd, J = 4.6, 0.7 Hz, 1H), 4.72 (s, 1H), 4.56 (s, 2H), 1.70 (s, 3H), 1.15 ppm (s, 3H); ^{19}F NMR (565 MHz, 300 K, $CDCl_3$): δ = −117.4 ppm (m, 1F); ^{13}C NMR (150 MHz, 300 K, $CDCl_3$): δ = 173.1, 168.2, 167.6, 161.3 (d, J = 249.5 Hz), 157.0, 131.5 (d, J = 3.3 Hz), 131.2 (d, J = 8.5 Hz), 129.7, 124.4 (d, J = 3.4 Hz), 122.2, 121.7 (d, J = 14.9 Hz), 115.7 (d, J = 21.0 Hz), 114.9, 76.6 (d, J = 3.7 Hz), 75.4, 67.1, 66.4, 62.2 (d, J = 3.5 Hz), 55.5, 19.3, 18.4 ppm; IR (film): $\tilde{\nu}$ = 3368, 2973, 1794, 1752, 1693, 1599, 1519, 1495, 1458, 1373, 1350, 1290, 1235, 1207, 1157, 1133, 1112, 1062, 1038, 1022 cm^{-1}; HRMS (ESI): m/z calculated for $C_{23}H_{23}O_6N_2FSNa$ [M+Na]$^+$: 497.1153, found: 497.1152; $[\alpha]_D^{25}$ = +127.7 (c = 1.0, $CHCl_3$).

3-Fluorobenzyl (2*S*,5*R*,6*R*)-3,3-dimethyl-7-oxo-6-(2-phenoxyacetamido)-4-thia-1-azabicyclo[3.2.0]heptane-2-carboxylate 4,4-dioxide (20b). According to General Procedure A, 3-fluorobenzyl (2S,5R,6R)-3,3-dimethyl-7-oxo-6-(2-phenoxyacetamido)-4-thia-1-azabicyclo[3.2.0]heptane-2-carboxylate (459 mg, 71%) along with some impurities was obtained from commercially-sourced 3-fluorobenzylbromide and penicillin V potassium salt (540 mg, 1.4 mmol), following column chromatography (10 g Sfär cartridge; 40 mL/min; initially, 100% cyclohexane (3 CV), followed by a linear gradient (20 CV): 0%→100% ethyl acetate in cyclohexane). The mixture was used without further purification to afford, according to General Procedure B, penicillin V sulfone **20b** (352 mg, 72%), following column chromatography (10 g Sfär cartridge; 40 mL/min; initially, 100% cyclohexane (3 CV), followed by a linear gradient (20 CV): 0%→100% ethyl acetate in cyclohexane). Clear colorless oil; ^{1}H NMR (600 MHz, 300 K, $CDCl_3$): δ = 8.17 (d, J = 10.6 Hz, 1H), 7.42−7.38 (m, 1H), 7.34−7.31 (m, 2H), 7.18 (d, J = 7.6 Hz, 1H), 7.12−7.09 (m, 2H), 7.05 (t, J = 7.4 Hz, 1H), 6.94 (d, J = 8.0 Hz, 2H), 6.20 (dd, J = 10.7, 4.6 Hz, 1H), 5.29 (d, J = 12.2 Hz, 1H), 5.20 (d, J = 12.2 Hz, 1H), 4.80 (d, J = 4.6 Hz, 1H), 4.61−4.55 (m, 3H), 1.60 (s, 3H), 1.32 ppm (s, 3H); ^{19}F NMR (565 MHz, 300 K, $CDCl_3$): δ = −111.8 ppm (m, 1F); ^{13}C NMR (150 MHz, 300 K, $CDCl_3$): δ = 173.5, 168.3, 166.4, 161.3 (d, J = 247.7 Hz), 156.8, 136.5 (d, J = 7.6 Hz), 130.6 (d, J = 8.5 Hz), 129.7, 124.3 (d, J = 3.1 Hz), 122.4, 116.1 (d, J = 21.1 Hz), 115.7 (d, J = 22.0 Hz), 114.9, 67.4 (d, J = 1.8 Hz), 67.0, 65.7, 64.7, 63.9, 56.3, 20.1, 17.9 ppm; IR (film): $\tilde{\nu}$ = 3407, 3068, 2980, 2940, 1806, 1758, 1699, 1593, 1520, 1492, 1456, 1376, 1322, 1289, 1241, 1209, 1171, 1116, 1081, 1065, 981 cm^{-1}; HRMS (ESI): m/z calculated for $C_{23}H_{23}O_7N_2FSNa$ [M+Na]$^+$: 513.1102, found: 513.1100; $[\alpha]_D^{25}$ = +95.4 (c = 1.40, $CHCl_3$).

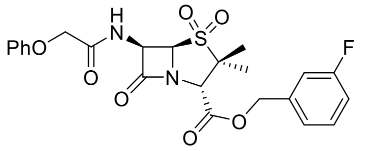

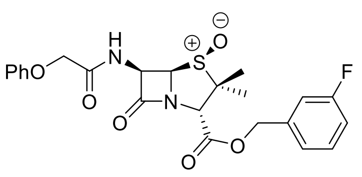

In addition to the penicillin V sulfone **20b**, the corresponding penicillin V sulfoxide **21b** (75 mg, 16%) was isolated in purified form as a single sulfoxide diastereomer. Note, the sulfoxide was tentatively assigned the (*S*)-configuration based on literature reports on peracid (including mCPBA)-mediated penicillin ester oxidations to sulfoxides.[11,20-21] Clear colorless oil; ^{1}H NMR (600 MHz, 300 K, $CDCl_3$): δ = 8.26 (d, *J* = 10.5 Hz, 1H), 7.41−7.37 (m, 1H), 7.33−7.30 (m, 2H), 7.19 (d, *J* = 7.6 Hz, 1H), 7.12−7.08 (m, 2H), 7.03 (t, *J* = 7.4 Hz, 1H), 6.95 (d, *J* = 7.9 Hz, 2H), 6.13 (dd, *J* = 10.5, 4.7 Hz, 1H), 5.29 (d, *J* = 12.2 Hz, 1H), 5.19 (d, *J* = 12.2 Hz, 1H), 5.05 (d, *J* = 4.7 Hz, 1H), 4.73 (s, 1H), 4.56 (s, 2H), 1.71 (s, 3H), 1.13 ppm (s, 3H); ^{19}F NMR (565 MHz, 300 K, $CDCl_3$): δ = −112.0 ppm (m, 1F); ^{13}C NMR (150 MHz, 300 K, $CDCl_3$): δ = 173.1, 168.2, 167.6, 162.8 (d, *J* = 247.3 Hz), 157.0, 136.9 (d, *J* = 7.5 Hz), 130.5 (d, *J* = 8.0 Hz), 129.7, 124.3 (d, *J* = 3.1 Hz), 122.2, 115.9 (d, *J* = 20.9 Hz), 115.7 (d, *J* = 22.0 Hz), 114.9, 76.6 (d, *J* = 3.4 Hz), 75.4, 67.1 (2C), 66.4, 55.5, 19.5, 18.5 ppm; IR (film): ṽ = 3368, 3068, 2979, 1793, 1751, 1694, 1592, 1519, 1492, 1457, 1374, 1292, 1243, 1207, 1158, 1080, 1062, 1039, 1022 cm^{-1}; HRMS (ESI): *m/z* calculated for $C_{23}H_{24}O_6N_2FS$ [M+H]$^+$: 475.1334, found: 475.1331; $[\alpha]_D^{25}$ = +149.6 (c = 0.3, $CHCl_3$).

4-Fluorobenzyl (2*S*,5*R*,6*R*)-3,3-dimethyl-7-oxo-6-(2-phenoxyacetamido)-4-thia-1-azabicyclo[3.2.0]heptane-2-carboxylate (19c). According to General Procedure A, penicillin V ester **19c** (337 mg, 53%) was obtained from commercially-sourced 4-fluorobenzylbromide and penicillin V potassium salt (540 mg, 1.4 mmol), following column chromatography (10 g Ultra cartridge; 36 mL/min; initially, 100% cyclohexane (3 CV), followed by a linear gradient (25 CV): 0%→80% ethyl acetate in cyclohexane). Clear colorless oil; ^{1}H NMR (600 MHz, 300 K, $CDCl_3$): δ = 7.39−7.32 (m, 5H), 7.09−7.03 (m, 3H), 6.94 (d, *J* = 8.6 Hz, 2H), 5.74 (dd, *J* = 9.2, 4.2 Hz, 1H), 5.58 (d, *J* = 4.2 Hz, 1H), 5.20−5.15 (m, 2H), 4.58 (d, *J* = 15.2 Hz, 1H), 4.54 (d, *J* = 15.1 Hz, 1H), 4.49 (s, 1H), 1.58 (s, 3H), 1.41 ppm (s, 3H); ^{19}F NMR (565 MHz, 300 K, $CDCl_3$): δ = −112.5 ppm (m, 1F); ^{13}C NMR (150 MHz, 300 K, $CDCl_3$): δ = 172.9, 167.8, 167.3, 161.8 (d, *J* = 248.1 Hz), 156.9, 130.8 (d, *J* = 8.0 Hz), 130.5 (d, *J* = 3.2 Hz), 129.8, 122.3, 115.7 (d, *J* = 21.4 Hz), 114.7, 70.3, 67.8, 67.1, 66.7, 64.7, 58.1, 31.9, 26.7 ppm; IR (film): ṽ = 3370, 3066, 3044, 2970, 2935, 1783, 1744, 1691, 1600, 1512, 1494, 1458, 1373, 1295, 12225, 1203, 1182, 1155, 1130, 1082, 1061, 1027, 962 cm^{-1}; HRMS (ESI): *m/z* calculated for $C_{23}H_{23}O_5N_2FSNa$ [M+Na]$^+$: 481.1204, found: 481.1202; $[\alpha]_D^{25}$ = +112.5 (c = 1.0, $CHCl_3$).

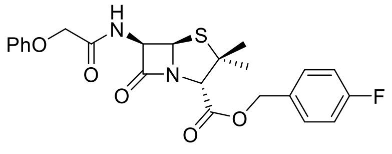

4-Fluorobenzyl (2*S*,5*R*,6*R*)-3,3-dimethyl-7-oxo-6-(2-phenoxyacetamido)-4-thia-1-azabicyclo[3.2.0]heptane-2-carboxylate 4,4-dioxide (20c). According to General Procedure B, penicillin V sulfone **20c** (178 mg, 49%) was obtained from penicillin V ester **19c** (337 mg, 0.74 mmol), following column chromatography (10 g Ultra cartridge; 36 mL/min; initially, 100% cyclohexane (3 CV), followed by a linear gradient (20 CV): 0%→100% ethyl acetate in cyclohexane). White amorphous solid; ^{1}H NMR (600 MHz, 300 K, $CDCl_3$): δ = 8.16 (d, *J* = 10.6 Hz, 1H), 7.40−7.38 (m, 2H), 7.34−7.31 (m, 2H), 7.12−7.09 (m, 2H), 7.04 (t, *J* = 7.3 Hz, 1H), 6.94 (d, *J* = 8.4 Hz, 2H), 6.19 (dd, *J* = 10.7, 4.6 Hz, 1H), 5.28 (d, *J* = 11.9 Hz, 1H), 5.18 (d, *J* = 11.9 Hz, 1H), 4.79 (d, *J* = 4.6 Hz, 1H), 4.60−4.55 (m, 2H), 4.55 (s, 1H), 1.58 (s, 3H), 1.28 ppm (s, 3H); ^{19}F NMR (565 MHz, 300 K, $CDCl_3$): δ = −111.7 ppm (m, 1F); ^{13}C NMR (150 MHz, 300 K, $CDCl_3$): δ = 173.5, 168.3, 166.4, 163.1 (d, *J* = 248.7 Hz), 156.8, 131.1 (d, *J* = 8.3 Hz), 130.1 (d, *J* = 3.2 Hz), 129.7, 122.3, 115.9 (d, *J* = 21.9 Hz), 114.8, 67.6, 67.0, 65.7, 64.7, 63.9, 56.2, 20.1, 17.9 ppm; IR (film): ṽ = 3406, 2980, 1806, 1758, 1699, 1619, 1599, 1520, 1495, 1458, 1376, 1322, 1289, 1237, 1210, 1170, 1116, 1065, 955 cm^{-1}; HRMS (ESI): *m/z* calculated for $C_{23}H_{24}O_7N_2FS$ [M+H]$^+$: 491.1283, found: 491.1283; $[\alpha]_D^{25}$ = +100.2 (c = 1.15, $CHCl_3$).

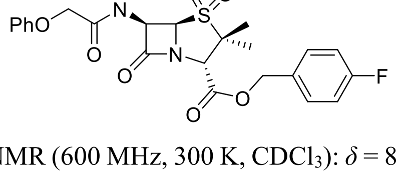

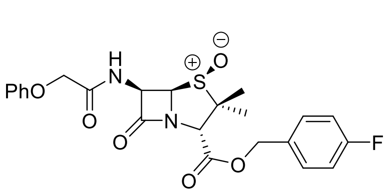

In addition to the penicillin V sulfone **20c**, the corresponding penicillin V sulfoxide **21c** (73 mg, 21%) was isolated in purified form as a single sulfoxide diastereomer. Note, the sulfoxide was tentatively assigned the (*S*)-configuration based on literature reports on peracid (including mCPBA)-mediated penicillin ester oxidations to sulfoxides.[11,20-21] Clear colorless oil; ^{1}H NMR (600 MHz, 300 K, $CDCl_3$): δ = 8.26 (d, J = 10.5 Hz, 1H), 7.40 (dd, J = 8.1, 5.5 Hz, 2H), 7.33−7.30 (m, 2H), 7.10 (t, J = 8.4 Hz, 2H), 7.04−7.02 (m, 1H), 6.95 (d, J = 8.6 Hz, 2H), 6.12 (dd, J = 10.5, 4.6 Hz, 1H), 5.29 (d, J = 11.9 Hz, 1H), 5.17 (d, J = 12.0 Hz, 1H), 5.03 (d, J = 4.7 Hz, 1H), 4.71 (s, 1H), 4.56 (s, 2H), 1.69 (s, 3H), 1.09 ppm (s, 3H); ^{19}F NMR (565 MHz, 300 K, $CDCl_3$): δ = −112.1 ppm (m, 1F); ^{13}C NMR (150 MHz, 300 K, $CDCl_3$): δ = 173.1, 168.2, 167.7, 163.0 (d, J = 248.6 Hz), 157.0, 131.0 (d, J = 8.0 Hz), 130.5 (d, J = 3.3 Hz), 129.7, 122.2, 115.8 (d, J = 21.9 Hz), 114.9, 76.6, 75.3, 67.2, 67.1, 66.3, 55.5, 19.5, 18.5 ppm; IR (film): ṽ = 3370, 3067, 2974, 1793, 1749, 1692, 1600, 1513, 1496, 1457, 1441, 1291, 1225, 1207, 1174, 1156, 1132, 1081, 1062, 1038, 1021 cm^{-1}.

3,5-Difluorobenzyl (2*S*,5*R*,6*R*)-3,3-dimethyl-7-oxo-6-(2-phenoxyacetamido)-4-thia-1-azabicyclo[3.2.0]heptane-2-carboxylate (19d). According to General Procedure A, penicillin V ester **19d** (475 mg, 71%) was obtained from commercially-sourced 2,4-difluorobenzylbromide and penicillin V potassium salt (540 mg, 1.4 mmol), following column chromatography (10 g Sfär cartridge; 40 mL/min; initially, 100% cyclohexane (3 CV), followed by a linear gradient (20 CV): 0%→100% ethyl acetate in cyclohexane). Clear colorless oil; ^{1}H NMR (600 MHz, 300 K, $CDCl_3$): δ = 7.35−7.33 (m, 3H), 7.05 (t, J = 7.3 Hz, 1H), 6.95−6.90 (m, 4H), 6.84−6.80 (m, 1H), 5.77 (dd, J = 9.2, 4.3 Hz, 1H), 5.60 (d, J = 4.3 Hz, 1H), 5.19 (d, J = 12.7 Hz, 1H), 5.15 (d, J = 12.7 Hz, 1H), 4.59 (d, J = 15.2 Hz, 1H), 4.56 (d, J = 15.2 Hz, 1H), 4.53 (s, 1H), 1.61 (s, 3H), 1.46 ppm (s, 3H); ^{19}F NMR (565 MHz, 300 K, $CDCl_3$): δ = −108.5 ppm (m, 2F); ^{13}C NMR (150 MHz, 300 K, $CDCl_3$): δ = 173.0, 167.8, 167.2, 163.1 (dd, J = 249.9, 12.4 Hz), 156.9, 138.3 (t, J = 9.3 Hz), 129.8, 122.4, 114.7, 111.1 (dd, J = 20.7, 5.5 Hz), 104.2 (t, J = 25.3 Hz), 70.3, 67.8, 67.1, 65.9, 64.7, 58.2, 31.9, 26.7 ppm; IR (film): ṽ = 3377, 3064, 2972, 2933, 1784, 1748, 1692, 1627, 1599, 1518, 1494, 1460, 1373, 1353, 1321, 1296, 1241, 1204, 1181, 1155, 1120, 1082, 1061, 958 cm^{-1}; HRMS (ESI): *m/z* calculated for $C_{23}H_{21}O_5N_2F_2S$ [M−H]$^{-}$: 475.1145, found: 475.1132; $[\alpha]_D^{25}$ = +104.0 (c = 0.2, $CHCl_3$).

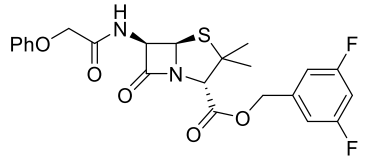

3,5-Difluorobenzyl (2*S*,5*R*,6*R*)-3,3-dimethyl-7-oxo-6-(2-phenoxyacetamido)-4-thia-1-azabicyclo[3.2.0]heptane-2-carboxylate 4,4-dioxide (20d). According to General Procedure B, penicillin V sulfone **20d** (270 mg, 53%) was obtained from penicillin V ester **19d** (477 mg, 1.0 mmol), following column chromatography (10 g Sfär cartridge; 40 mL/min; initially, 100% cyclohexane (3 CV), followed by a linear gradient (20 CV): 0%→100% ethyl acetate in cyclohexane). White amorphous solid; ^{1}H NMR (600 MHz, 300 K, $CDCl_3$): δ = 8.17 (d, J = 10.6 Hz, 1H), 7.35−7.32 (m, 2H), 7.06−7.04 (m, 1H), 6.95−6.91 (m, 4H), 6.88−6.84 (m, 1H), 6.21 (dd, J = 10.7, 4.6 Hz, 1H), 5.24 (d, J = 12.5 Hz, 1H), 5.19 (d, J = 12.5 Hz, 1H), 4.81 (d, J = 4.6 Hz, 1H), 4.61−4.56 (m, 3H), 1.62 (s, 3H), 1.35 ppm (s, 3H); ^{19}F NMR (565 MHz, 300 K, $CDCl_3$): δ = −108.1 ppm (m, 2F); ^{13}C NMR (150 MHz, 300 K, $CDCl_3$): δ = 173.5, 168.3, 166.3, 163.2 (dd, J = 250.6, 12.3 Hz), 156.8, 137.7 (t, J = 8.8 Hz), 129.8, 122.4, 114.9, 111.5 (dd, J = 20.6, 5.5 Hz), 104.6 (t, J = 25.1 Hz), 67.0, 66.7, 65.7, 64.7, 63.9, 56.3, 20.2, 17.9 ppm; IR (film): ṽ = 3402, 3090, 3066, 2986, 2941, 1806, 1759, 1698, 1627, 1599, 1519, 1495, 1463, 1322, 1289, 1209, 1170, 1118, 1065, 953 cm^{-1}; HRMS (ESI): *m/z* calculated for $C_{23}H_{22}O_7N_2F_2SNa$ [M+Na]$^{+}$: 531.1008, found: 531.1008; $[\alpha]_D^{25}$ = +93.2 (c = 1.0, $CHCl_3$).

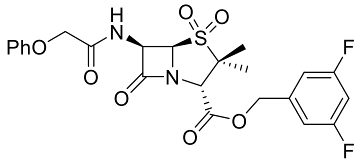

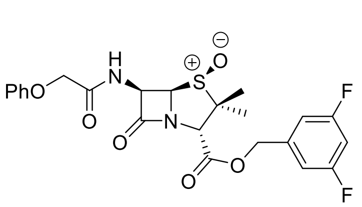

In addition to the penicillin V sulfone **20d**, penicillin V sulfoxide **21d** (214 mg, 43%) was isolated in purified form as a single sulfoxide diastereomer. Note, the sulfoxide was tentatively assigned the (*S*)-configuration based on literature reports on peracid (including mCPBA)-mediated penicillin ester oxidations to sulfoxides.[11,20-21] Clear colorless oil; ^{1}H NMR (600 MHz, 300 K, $CDCl_3$): δ = 8.26 (d, *J* = 10.5 Hz, 1H), 7.33−7.31 (m, 2H), 7.05−7.02 (m, 1H), 6.96−6.94 (m, 2H), 6.94−6.92 (m, 2H), 6.84 (tt, *J* = 13.3, 2.3 Hz, 1H), 6.14 (dd, *J* = 10.5, 4.7 Hz, 1H), 5.25 (d, *J* = 12.5 Hz, 1H), 5.18 (d, *J* = 12.5 Hz, 1H), 5.06 (d, *J* = 4.7 Hz, 1H), 4.75 (s, 1H), 4.56 (s, 2H), 1.73 (s, 3H), 1.17 ppm (s, 3H); ^{19}F NMR (565 MHz, 300 K, $CDCl_3$): δ = −108.3 ppm (m, 2F); ^{13}C NMR (150 MHz, 300 K, $CDCl_3$): δ = 173.2, 168.3, 167.6, 163.2 (dd, *J* = 250.3, 12.6 Hz), 157.0, 138.1 (t, *J* = 9.3 Hz), 129.7, 122.2, 114.9, 111.4 (dd, *J* = 20.7, 5.5 Hz), 104.4 (t, *J* = 25.2 Hz), 76.6, 75.3, 67.1, 66.4, 66.3, 55.6, 19.5, 18.6 ppm; IR (film): ṽ = 3370, 3067, 2973, 2939, 1793, 1753, 1691, 1627, 1599, 1518, 1494, 1462, 1374, 1351, 1322, 1291, 1205, 1158, 1121, 1062, 1041, 1022, 954 cm^{-1}; HRMS (ESI): *m/z* calculated for $C_{23}H_{22}O_6N_2F_2S$ $[M+H]^+$: 515.1059, found: 515.1060; $[\alpha]_D^{25}$ = +128.2 (c = 1.35, $CHCl_3$).

2,4-Difluorobenzyl (2*S*,5*R*,6*R*)-3,3-dimethyl-7-oxo-6-(2-phenoxyacetamido)-4-thia-1-azabicyclo[3.2.0]heptane-2-carboxylate (19e). According to General Procedure A, penicillin V ester **19e** (497 mg, 75%) was obtained from commercially-sourced 2,4-difluorobenzylbromide and penicillin V potassium salt (540 mg, 1.4 mmol), following column chromatography (10 g Sfär cartridge; 40 mL/min; initially, 100% cyclohexane (3 CV), followed by a linear gradient (20 CV): 0%→100% ethyl acetate in cyclohexane). Clear colorless oil; ^{1}H NMR (600 MHz, 300 K, $CDCl_3$): δ = 7.43−7.40 (m, 1H), 7.35−7.32 (m, 3H), 7.05 (t, *J* = 7.4 Hz, 1H), 6.95−6.94 (m, 2H), 6.92−6.86 (m, 2H), 5.76 (dd, *J* = 9.2, 4.3 Hz, 1H), 5.59 (d, *J* = 4.3 Hz, 1H), 5.25 (d, *J* = 12.2 Hz, 1H), 5.20 (d, *J* = 12.2 Hz, 1H), 4.59 (d, *J* = 15.2 Hz, 1H), 4.55 (d, *J* = 15.1 Hz, 1H), 4.49 (s, 1H), 1.59 (s, 3H), 1.43 ppm (s, 3H); ^{19}F NMR (565 MHz, 300 K, $CDCl_3$): δ = −107.7 (m, 1F), −112.9 ppm (m, 1F); ^{13}C NMR (150 MHz, 300 K, $CDCl_3$): δ = 173.0, 167.8, 167.3, 163.5 (dd, *J* = 251.5, 11.9 Hz), 161.5 (dd, *J* = 252.1, 12.1 Hz), 156.9, 132.5 (dd, *J* = 9.9, 5.1 Hz), 129.8, 122.4, 118.0 (dd, *J* = 15.1, 4.0 Hz), 114.8, 111.6 (dd, *J* = 21.5, 3.7 Hz), 104.3 (t, *J* = 25.3 Hz), 70.3, 67.8, 67.2, 64.8, 60.9 (d, *J* = 3.2 Hz), 58.1, 31.8, 26.7 ppm; IR (film): ṽ = 3342, 3071, 2972, 2931, 1786, 1745, 1690, 1622, 1600, 1509, 1496, 1458, 1435, 1285, 1240, 1205, 1180, 1155, 1142, 1102, 1083, 1062, 962 cm^{-1}; HRMS (ESI): *m/z* calculated for $C_{23}H_{22}O_5N_2F_2SNa$ $[M+Na]^+$: 499.1110, found: 499.1110; $[\alpha]_D^{25}$ = +123.3 (c = 0.2, $CHCl_3$).

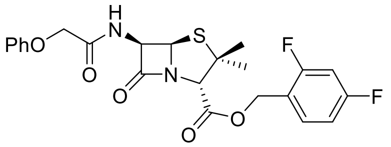

2,4-Difluorobenzyl (2*S*,5*R*,6*R*)-3,3-dimethyl-7-oxo-6-(2-phenoxyacetamido)-4-thia-1-azabicyclo[3.2.0]heptane-2-carboxylate 4,4-dioxide (20e). According to General Procedure B, penicillin V sulfone **20e** (335 mg, 66%) was obtained from penicillin V ester **19e** (476 mg, 1.0 mmol), following column chromatography (10 g Sfär cartridge; 40 mL/min; initially, 100% cyclohexane (3 CV), followed by a linear gradient (20 CV): 0%→100% ethyl acetate in cyclohexane). Clear colorless oil; ^{1}H NMR (600 MHz, 300 K, $CDCl_3$): δ = 8.16 (d, *J* = 10.6 Hz, 1H), 7.43−7.39 (m, 1H), 7.34−7.31 (m, 2H), 7.05−7.03 (m, 1H), 6.94−6.89 (m, 4H), 6.19 (ddd, *J* = 10.6, 4.6, 0.9 Hz, 1H), 5.32 (d, *J* = 12.0 Hz, 1H), 5.20 (d, *J* = 12.0 Hz, 1H), 4.80 (dd, *J* = 4.6, 1.2 Hz, 1H), 4.60−4.54 (m, 3H), 1.58 (s, 3H), 1.33 ppm (s, 3H); ^{19}F NMR (565 MHz, 300 K, $CDCl_3$): δ = −106.8 (m, 1F), −112.8 ppm (m, 1F); ^{13}C NMR (150 MHz, 300 K, $CDCl_3$): δ = 173.5, 168.3, 166.3, 163.7 (dd, *J* = 252.0, 12.0 Hz), 161.6 (dd, *J* = 252.2, 12.2 Hz), 156.8, 132.7 (dd, *J* = 9.9, 4.6 Hz), 129.7, 122.3, 117.6 (dd, *J* = 14.9, 3.8 Hz), 114.8, 111.8 (dd, *J* = 21.4, 3.6 Hz), 104.4 (t, *J* = 25.2 Hz), 67.0, 65.7, 64.7, 63.9, 61.8 (d, *J* = 3.0 Hz), 56.2, 20.0, 17.8 ppm; IR (film): ṽ = 3402, 3079, 2980, 1806, 1758, 1699,

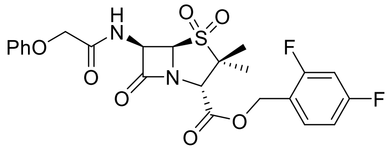

1622, 1601, 1510, 1496, 1460, 1436, 1377, 1350, 1323, 1284, 1240, 1209, 1169, 1143, 1117, 1102, 1081, 1065 cm^{-1}; HRMS (ESI): *m/z* calculated for $C_{23}H_{22}O_7N_2F_2SNa$ $[M+Na]^+$: 531.1008, found: 531.1006; $[\alpha]_D^{25}$ = +70.8 (c = 1.1, $CHCl_3$).

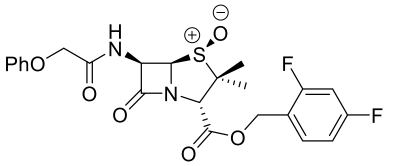

In addition to the penicillin V sulfone **20e**, penicillin V sulfoxide **21e** (166 mg, 34%) was isolated in purified form as a single sulfoxide diastereomer. Note, the sulfoxide was tentatively assigned the (*S*)-configuration based on literature reports on peracid (including mCPBA)-mediated penicillin ester oxidations to sulfoxides.[11,20-21] Clear colorless oil; ^{1}H NMR (600 MHz, 300 K, $CDCl_3$): δ = 8.26 (d, *J* = 10.4 Hz, 1H), 7.45−7.41 (m, 1H), 7.33−7.30 (m, 2H), 7.04−7.01 (m, 1H), 6.96−6.88 (m, 4H), 6.12 (dd, *J* = 10.5, 4.6 Hz, 1H), 5.33 (d, *J* = 12.1 Hz, 1H), 5.19 (d, *J* = 12.1 Hz, 1H), 5.04 (d, *J* = 4.7 Hz, 1H), 4.70 (s, 1H), 4.56 (s, 2H), 1.70 (s, 3H), 1.15 ppm (s, 3H); ^{19}F NMR (565 MHz, 300 K, $CDCl_3$): δ = −107.3 (m, 1F), −112.9 ppm (m, 1F); ^{13}C NMR (150 MHz, 300 K, $CDCl_3$): δ = 173.1, 168.3, 167.6, 163.6 (dd, *J* = 251.9, 12.0 Hz), 161.6 (dd, *J* = 252.0, 12.1 Hz), 157.0, 132.6 (dd, *J* = 9.9, 5.3 Hz), 129.7, 122.2, 117.9 (dd, *J* = 14.9, 3.8 Hz), 114.9, 111.7 (dd, *J* = 21.6, 3.7 Hz), 104.3 (t, *J* = 25.3 Hz), 76.6, 75.4, 67.1, 66.3, 61.5 (d, *J* = 3.0 Hz), 55.5, 19.4, 18.4 ppm; IR (film): ṽ = 3369, 3072, 2980, 2939, 1794, 1753, 1693, 1622, 1601, 1509, 1496, 1459, 1436, 1374, 1284, 1207, 1157, 1142, 1102, 1080, 1063, 1038, 1022 cm^{-1}; HRMS (ESI): *m/z* calculated for $C_{23}H_{23}O_6N_2F_2S$ $[M+H]^+$: 493.1239, found: 493.1235; $[\alpha]_D^{25}$ = +132.0 (c = 1.10, $CHCl_3$).

3,4,5-Trifluorobenzyl (2*S*,5*R*,6*R*)-3,3-dimethyl-7-oxo-6-(2-phenoxyacetamido)-4-thia-1-azabicyclo[3.2.0]heptane-2-carboxylate (19f). According to General Procedure A, penicillin V ester **19f** (550 mg, 79%) was obtained from commercially-sourced 3,4,5-trifluorobenzylbromide and penicillin V potassium salt (540 mg, 1.4 mmol), following column chromatography (10 g Sfär cartridge; 40 mL/min; initially, 100% cyclohexane (3 CV), followed by a linear gradient (20 CV): 0%→100% ethyl acetate in cyclohexane). White amorphous solid; ^{1}H NMR (600 MHz, 300 K, $CDCl_3$): δ = 7.36−7.33 (m, 3H), 7.07−7.02 (m, 3H), 6.96−6.94 (m, 2H), 5.77 (dd, *J* = 9.2, 4.3 Hz, 1H), 5.60 (d, *J* = 4.3 Hz, 1H), 5.15 (d, *J* = 12.5 Hz, 1H), 5.11 (d, *J* = 12.5 Hz, 1H), 4.60 (d, *J* = 15.2 Hz, 1H), 4.45 (d, *J* = 15.2 Hz, 1H), 4.52 (s, 1H), 1.61 (s, 3H), 1.45 ppm (s, 3H); ^{19}F NMR (565 MHz, 300 K, $CDCl_3$): δ = −132.8 (m, 2F), −159.4 ppm (m, 1F); ^{13}C NMR (150 MHz, 300 K, $CDCl_3$): δ = 173.0, 167.8, 167.2, 156.9, 151.3 (ddd, *J* = 251.5, 10.1, 4.4 Hz), 140.0 (dt, *J* = 252.9, 14.8 Hz), 130.8 (td, *J* = 7.5, 4.6 Hz), 129.8, 122.4, 114.8, 112.8 (dd, *J* = 16.8, 4.6 Hz), 70.4, 67.8, 67.2, 65.7, 64.7, 58.2, 31.9, 26.8 ppm; IR (film): ṽ = 3358, 2980, 1786, 1749, 1695, 1624, 1599, 1533, 1495, 1451, 1351, 1297, 1238, 1205, 1181, 1156, 1082, 1053, 1006, 965 cm^{-1}; HRMS (ESI): *m/z* calculated for $C_{23}H_{21}O_5N_2F_3SNa$ $[M+Na]^+$: 517.1015, found: 517.1016; $[\alpha]_D^{25}$ = +114.0 (c = 0.05, $CHCl_3$).

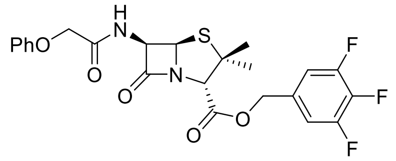

3,4,5-Tifluorobenzyl (2*S*,5*R*,6*R*)-3,3-dimethyl-7-oxo-6-(2-phenoxyacetamido)-4-thia-1-azabicyclo[3.2.0]heptane-2-carboxylate 4,4-dioxide (20f). According to General Procedure B, penicillin V sulfone **20f** (260 mg, 49%) was obtained from penicillin V ester **19f** (500 mg, 1.0 mmol), following column chromatography (10 g Sfär cartridge; 40 mL/min; initially, 100% cyclohexane (3 CV), followed by a linear gradient (20 CV): 0%→100% ethyl acetate in cyclohexane). White amorphous solid; ^{1}H NMR (600 MHz, 300 K, $CDCl_3$): δ = 8.16 (d, *J* = 10.6 Hz, 1H), 7.34−7.32 (m, 2H), 7.06−7.03 (m, 3H), 6.94 (d, *J* = 8.6 Hz, 2H), 6.21 (dd, *J* = 10.7, 4.6 Hz, 1H), 5.19 (d, *J* = 12.4 Hz, 1H), 5.15 (d, *J* = 12.4 Hz, 1H), 4.81 (dd, *J* = 4.6, 1.2 Hz, 1H), 4.61−4.55 (m, 3H), 1.61 (s, 3H), 1.35 ppm (s, 3H); ^{19}F NMR (565 MHz, 300 K, $CDCl_3$): δ = −132.4 (m, 2F), −158.6 ppm (m, 1F); ^{13}C NMR (150 MHz, 300 K, $CDCl_3$): δ = 173.5, 168.3, 166.3, 156.8, 151.4 (ddd, *J* = 251.4, 10.0, 4.2 Hz), 140.2 (dt, *J* = 254.4, 15.0 Hz), 130.3 (m), 129.8, 122.4, 114.8, 113.1 (dd, *J* = 16.8, 4.6 Hz), 67.0, 66.5, 65.7, 64.7, 63.9, 56.3,

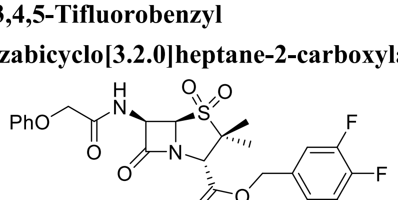

20.2, 17.9 ppm; IR (film): ṽ = 3401, 3064, 2979, 1806, 1759, 1699, 1600, 1533, 1495, 1452, 1386, 1353, 1323, 1290, 1238, 1210, 1170, 1117, 1053 cm^{-1}; HRMS (ESI): *m/z* calculated for $C_{23}H_{22}O_7N_2F_3S$ $[M+H]^+$: 527.1094, found: 527.1094; $[\alpha]_D^{25}$ = +88.9 (c = 1.20, $CHCl_3$).

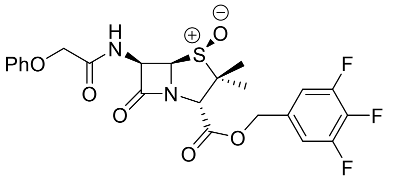

In addition to the penicillin V sulfone **20f**, penicillin V sulfoxide **21f** (196 mg, 38%) was isolated in purified form as a single sulfoxide diastereomer. Note, the sulfoxide was tentatively assigned the (*S*)-configuration based on literature reports on peracid (including mCPBA)-mediated penicillin ester oxidations to sulfoxides.[11,20-21] White amorphous solid; ^1H NMR (600 MHz, 300 K, $CDCl_3$): δ = 8.25 (d, J = 10.5 Hz, 1H), 7.31 (t, J = 7.6 Hz, 2H), 7.06−7.02 (m, 3H), 6.95 (d, J = 8.6 Hz, 2H), 6.13 (dd, J = 10.5, 4.6 Hz, 1H), 5.19 (d, J = 12.4 Hz, 1H), 5.14 (d, J = 12.4 Hz, 1H), 5.05 (dd, J = 4.7, 0.5 Hz, 1H), 4.72 (s, 1H), 4.56 (s, 2H), 1.72 (s, 3H), 1.16 ppm (s, 3H); ^{19}F NMR (565 MHz, 300 K, $CDCl_3$): δ = −132.6 (m, 2F), −159.0 ppm (m, 1F); ^{13}C NMR (150 MHz, 300 K, $CDCl_3$): δ = 173.2, 168.3, 167.6, 157.0, 151.3 (ddd, J = 251.9, 10.1, 3.6 Hz), 140.0 (dt, J = 254.0, 15.2 Hz), 130.7 (td, J = 7.7, 4.5 Hz), 129.7, 122.2, 114.8, 113.1 (dd, J = 16.9, 4.7 Hz), 76.5 (d, J = 4.0 Hz), 75.3, 67.0, 66.3, 66.1, 55.5, 19.5, 18.6 ppm; IR (film): ṽ = 3369, 3060, 2972, 2935, 1793, 1753, 1692, 1624, 1599, 1532, 1495, 1451, 1352, 1291, 1237, 1206, 1158, 1134, 1053, 1022, 967 cm^{-1}; HRMS (ESI): *m/z* calculated for $C_{23}H_{22}O_6N_2F_3S$ $[M+H]^+$: 511.1145, found: 511.1145; $[\alpha]_D^{25}$ = +132.2 (c = 1.30, $CHCl_3$).

2,4,6-Trifluorobenzyl (2*S*,5*R*,6*R*)-3,3-dimethyl-7-oxo-6-(2-phenoxyacetamido)-4-thia-1-azabicyclo[3.2.0]heptane-2-carboxylate (19g). According to General Procedure A, penicillin V ester **19g** (531 mg, 77%) was obtained from commercially-sourced 2,4,6-trifluorobenzylbromide and penicillin V potassium salt (540 mg, 1.4 mmol), following column chromatography (10 g Sfär cartridge; 40 mL/min; initially, 100% cyclohexane (3 CV), followed by a linear gradient (20 CV): 0%→100% ethyl acetate in cyclohexane). Clear colorless oil; ^1H NMR (600 MHz, 300 K, $CDCl_3$): δ = 7.35−7.31 (m, 3H), 7.05 (t, J = 7.4 Hz, 1H), 6.94 (d, J = 8.0 Hz, 2H), 6.77−6.72 (m, 2H), 5.75 (dd, J = 9.2, 4.3 Hz, 1H), 5.58 (d, J = 4.3 Hz, 1H), 5.25 (s, 2H), 4.59 (d, J = 15.2 Hz, 1H), 4.55 (d, J = 15.2 Hz, 1H), 4.48 (s, 1H), 1.58 (s, 3H), 1.48 ppm (s, 3H); ^{19}F NMR (565 MHz, 300 K, $CDCl_3$): δ = −104.7 (m, 1F), −110.8 ppm (m, 2F); ^{13}C NMR (150 MHz, 300 K, $CDCl_3$): δ = 173.0, 167.8, 167.1, 163.6 (dt, J = 252.3, 15.6 Hz), 162.1 (ddd, J = 253.1, 15.1, 10.1 Hz), 156.9, 129.8, 122.4, 114.8, 107.3 (td, J = 19.3, 4.4 Hz), 100.6 (m), 70.3, 67.8, 67.2, 64.8, 58.1, 54.6 (t, J = 3.6 Hz), 31.8, 26.7 ppm; IR (film): ṽ = 3365, 3069, 2973, 2936, 1787, 1749, 1693, 1648, 1628, 1611, 1519, 1498, 1446, 1296, 1240, 1205, 1178, 1155, 1122, 1075, 999 cm^{-1}; HRMS (ESI): *m/z* calculated for $C_{23}H_{21}O_5N_2F_3SNa$ $[M+Na]^+$: 517.1015, found: 517.1014; $[\alpha]_D^{25}$ = +117.1 (c = 0.2, $CHCl_3$).

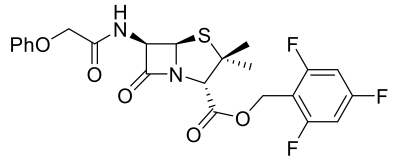

2,4,6-Tifluorobenzyl (2*S*,5*R*,6*R*)-3,3-dimethyl-7-oxo-6-(2-phenoxyacetamido)-4-thia-1-azabicyclo[3.2.0]heptane-2-carboxylate 4,4-dioxide (20g). According to General Procedure B, penicillin V sulfone **20g** (327 mg, 58%) was obtained from penicillin V ester **19g** (531 mg, 1.07 mmol), following column chromatography (10 g Sfär cartridge; 40 mL/min; initially, 100% cyclohexane (3 CV), followed by a linear gradient (20 CV): 0%→100% ethyl acetate in cyclohexane). Clear colorless oil; ^1H NMR (600 MHz, 300 K, $CDCl_3$): δ = 8.16 (d, J = 10.6 Hz, 1H), 7.32 (t, J = 7.9 Hz, 2H), 7.04 (t, J = 7.3 Hz, 1H), 6.94 (d, J = 8.2 Hz, 2H), 6.77 (t, J = 8.0 Hz, 2H), 6.19 (dd, J = 10.7, 4.6 Hz, 1H), 5.31 (d, J = 12.1 Hz, 1H), 5.28 (d, J = 12.1 Hz, 1H), 4.80 (d, J = 4.6 Hz, 1H), 4.60−4.55 (m, 3H), 1.58 (s, 3H), 1.39 ppm (s, 3H); ^{19}F NMR (565 MHz, 300 K, $CDCl_3$): δ = −103.9 (m, 1F), −110.9 ppm (m, 2F); ^{13}C NMR (150 MHz, 300 K, $CDCl_3$): δ = 173.4, 168.3, 166.2, 163.7 (dt, J = 253.4, 15.5 Hz), 162.0 (ddd, J = 253.0, 15.2, 10.0 Hz), 156.8, 129.7, 122.3, 114.8, 106.9 (td, J = 19.1, 4.6 Hz), 100.8 (m), 67.0, 65.7, 64.7,

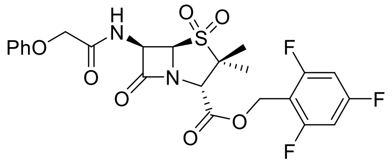

63.9, 56.3, 55.4 (t, J = 3.6 Hz), 20.1, 17.8 ppm; IR (film): ṽ = 3403, 3080, 2978, 2945, 1807, 1762, 1699, 1648, 1629, 1611, 1519, 1497, 1447, 1323, 1289, 1240, 1209, 1171, 1118, 1073, 999 cm^{-1}; HRMS (ESI): *m/z* calculated for $C_{23}H_{21}O_7N_2F_3SNa$ $[M+Na]^+$: 549.0914, found: 549.0912; $[\alpha]_D^{25}$ = +56.0 (c = 1.40, $CHCl_3$).

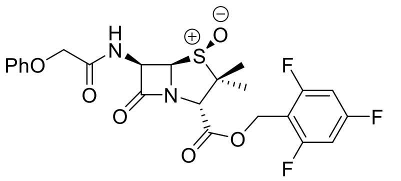

In addition to the penicillin V sulfone **20g**, penicillin V sulfoxide **21g** (69 mg, 13%) was isolated in purified form as a single sulfoxide diastereomer. Note, the sulfoxide was tentatively assigned the (*S*)-configuration based on literature reports on peracid (including mCPBA)-mediated penicillin ester oxidations to sulfoxides.[11,20-21] Clear colorless oil; ^{1}H NMR (600 MHz, 300 K, $CDCl_3$): δ = 8.25 (d, J = 10.4 Hz, 1H), 7.32 (t, J = 7.9 Hz, 2H), 7.03 (t, J = 7.4 Hz, 1H), 6.95 (d, J = 8.5 Hz, 2H), 6.76 (t, J = 8.0 Hz, 2H), 6.12 (dd, J = 10.5, 4.6 Hz, 1H), 5.32 (d, J = 11.8 Hz, 1H), 5.26 (d, J = 12.1 Hz, 1H), 5.05 (d, J = 4.6 Hz, 1H), 4.70 (s, 1H), 4.56 (s, 2H), 1.69 (s, 3H), 1.21 ppm (s, 3H); ^{19}F NMR (565 MHz, 300 K, $CDCl_3$): δ = −104.3 (m, 1F), −110.9 ppm (m, 2F); ^{13}C NMR (150 MHz, 300 K, $CDCl_3$): δ = 173.0, 168.3, 167.5, 163.6 (m), 162.0 (m), 157.0, 129.7, 122.2, 114.9, 107.2 (m), 100.7 (m), 76.6, 75.4, 67.1, 66.4, 55.5, 55.2 (t, J = 3.8 Hz), 19.4, 18.5 ppm; IR (film): ṽ = 3372, 3078, 2978, 1795, 1757, 1694, 1628, 1611, 1519, 1497, 1447, 1291, 1241, 1207, 1174, 1157, 1122, 1073, 1038, 1022, 999 cm^{-1}; HRMS (ESI): *m/z* calculated for $C_{23}H_{22}O_6N_2F_3S$ $[M+H]^+$: 511.1145, found: 511.1144; $[\alpha]_D^{25}$ = +87.1 (c = 0.1, $CHCl_3$).

3,5-(Bistrifluoromethyl)benzyl (2*S*,5*R*,6*R*)-3,3-dimethyl-7-oxo-6-(2-phenoxyacetamido)-4-thia-1-azabicyclo[3.2.0]heptane-2-carboxylate 4,4-dioxide (20h). According to General Procedure A, 3,5-bistrifluoromethylbenzyl (2*S*,5*R*,6*R*)-3,3-dimethyl-7-oxo-6-(2-phenoxyacetamido)-4-thia-1-azabicyclo[3.2.0]heptane-2-carboxylate (763 mg, 95%) along with some impurities was obtained from commercially-sourced 3,5-(bistrifluoromethyl)benzylbromide and penicillin V potassium salt (540 mg, 1.4 mmol), following column chromatography (10 g Ultra cartridge; 36 mL/min; initially, 100% cyclohexane (3 CV), followed by a linear gradient (20 CV): 0%→80% ethyl acetate in cyclohexane). The mixture was used in the next step without further purification to afford, according to General Procedure B, penicillin V sulfone **20h** (456 mg, 58%), following column chromatography (10 g Ultra cartridge; 36 mL/min; initially, 100% cyclohexane (3 CV), followed by a linear gradient (20 CV): 0%→100% ethyl acetate in cyclohexane). White amorphous solid; ^{1}H NMR (600 MHz, 300 K, $CDCl_3$): δ = 8.16 (d, J = 10.6 Hz, 1H), 7.94 (s, 1H), 7.86 (s, 2H), 7.35−7.31 (m, 2H), 7.06−7.04 (m, 1H), 6.95−6.94 (m, 2H), 6.21 (dd, J = 10.6, 4.6 Hz, 1H), 5.39−5.34 (m, 2H), 4.82 (d, J = 4.6 Hz, 1H), 4.61 (s, 1H), 4.61−4.55 (m, 2H), 1.62 (s, 3H), 1.35 ppm (s, 3H); ^{19}F NMR (565 MHz, 300 K, $CDCl_3$): δ = −63.0 ppm (s, 6F); ^{13}C NMR (150 MHz, 300 K, $CDCl_3$): δ = 173.5, 168.3, 166.4, 156.8, 136.7, 132.5 (q, J = 33.8 Hz), 129.8, 128.7 (q, J = 3.4 Hz), 123.0 (m), 122.9 (q, J = 272.9 Hz), 122.4, 114.9, 67.0, 66.4, 65.7, 64.7, 63.9, 56.3, 20.2, 17.9 ppm; IR (film): ṽ = 3400, 2980, 1808, 1761, 1699, 1600, 1521, 1496, 1465, 1394, 1370, 1324, 1279, 1173, 1134, 1065 cm^{-1}; HRMS (ESI): *m/z* calculated for $C_{25}H_{22}O_7N_2F_6SNa$ $[M+Na]^+$: 631.0944, found: 631.0939; $[\alpha]_D^{25}$ = +75.7 (c = 1.0, $CHCl_3$).

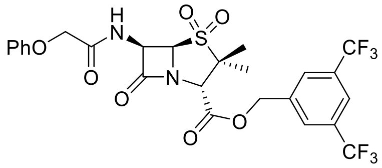

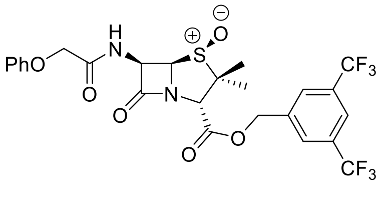

In addition to the penicillin V sulfone **20h**, penicillin V sulfoxide **21h** (183 mg, 24%) was isolated in purified form as a single sulfoxide diastereomer. Note, the sulfoxide was tentatively assigned the (*S*)-configuration based on literature reports on peracid (including mCPBA)-mediated penicillin ester oxidations to sulfoxides.[11,20-21] White amorphous solid; ^{1}H NMR (600 MHz, 300 K, $CDCl_3$): δ = 8.25 (d, J = 10.5 Hz, 1H), 7.92 (s, 1H), 7.87 (s, 2H), 7.32 (t, J = 7.8 Hz, 2H), 7.03 (t, J = 7.2 Hz, 1H), 6.95 (d, J = 8.5 Hz, 2H), 6.14 (dd, J = 10.5, 4.7 Hz, 1H), 5.39 (d, J = 12.7 Hz, 1H), 5.34 (d, J = 12.7 Hz, 1H), 5.06 (d, J = 4.7 Hz, 1H), 4.77 (s, 1H), 4.56 (s, 2H), 1.73 (s, 3H), 1.16 ppm (s, 3H); ^{19}F NMR (565 MHz, 300 K, $CDCl_3$): δ = −63.0 ppm (s, 6F); ^{13}C NMR (150 MHz, 300 K, $CDCl_3$):

δ = 173.2, 168.3, 167.7, 157.0, 137.1, 132.4 (q, J = 33.7 Hz), 129.7, 128.7 (br), 122.9 (q, J = 272.8 Hz), 122.9 (m), 122.2, 114.9, 76.6, 75.3, 67.1, 66.3, 66.1, 55.6, 19.4, 18.6 ppm; IR (film): ṽ = 3375, 3065, 2978, 1796, 1757, 1692, 1600, 1517, 1496, 1465, 1453, 1393, 1371, 1348, 1278, 1243, 1203, 1174, 1132, 1112, 1081, 1062, 1041 cm^{-1}; HRMS (ESI): *m/z* calculated for $C_{25}H_{22}O_6N_2F_6SNa$ $[M+Na]^+$: 593.1176, found: 593.1175; $[\alpha]_D^{25}$ = +125.0 (c = 1.25, $CHCl_3$).

3-Nitrobenzyl (2*S*,5*R*,6*R*)-3,3-dimethyl-7-oxo-6-(2-phenoxyacetamido)-4-thia-1-azabicyclo[3.2.0]heptane-2-carboxylate (19i). According to General Procedure A, penicillin V ester **19i** (335 mg, 49%) was obtained from

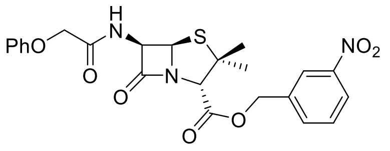

commercially-sourced 3-nitrobenzylbromide and penicillin V potassium salt (540 mg, 1.4 mmol), following column chromatography (10 g Ultra cartridge; 36 mL/min; initially, 100% cyclohexane (3 CV), followed by a linear gradient (20 CV): 0%→50% ethyl acetate in cyclohexane). Clear colorless oil; ^{1}H NMR (600 MHz, 300 K, $CDCl_3$): δ = 8.28 (s, 1H), 8.26 (d, J = 9.0 Hz, 1H), 7.74 (d, J = 7.6 Hz, 1H), 7.61 (t, J = 7.9 Hz, 1H), 7.36−7.33 (m, 3H), 7.07−7.05 (m, 1H), 6.95 (d, J = 8.6 Hz, 2H), 5.77 (dd, J = 9.2, 4.3 Hz, 1H), 5.61 (d, J = 4.3 Hz, 1H), 5.33 (d, J = 12.6 Hz, 1H), 5.29 (d, J = 12.6 Hz, 1H), 4.61−4.54 (m, 2H), 4.54 (s, 1H), 1.61 (s, 3H), 1.46 ppm (s, 3H); ^{13}C NMR (150 MHz, 300 K, $CDCl_3$): δ = 173.0, 167.8, 167.2, 156.9, 148.4, 136.7, 134.3, 129.9, 129.8, 123.7, 123.4, 122.4, 114.8, 70.4, 67.9, 67.2, 66.0, 64.8, 58.2, 31.9, 26.8 ppm; IR (film): ṽ = 3367, 3071, 3040, 2971, 2933, 1788, 1746, 1690, 1599, 1531, 1495, 1458, 1442, 1352, 1298, 1240, 1206, 1182, 1156, 1083, 1062 cm^{-1}; HRMS (ESI): *m/z* calculated for $C_{23}H_{23}O_7N_3SNa$ $[M+Na]^+$: 508.1149, found: 508.1148; $[\alpha]_D^{25}$ = +102.1 (c = 0.1, $CHCl_3$).

3-Nitrobenzyl (2*S*,5*R*,6*R*)-3,3-dimethyl-7-oxo-6-(2-phenoxyacetamido)-4-thia-1-azabicyclo[3.2.0]heptane-2-carboxylate 4,4-dioxide (20i). According to General Procedure B, penicillin V sulfone **20i** (140 mg, 44%) was

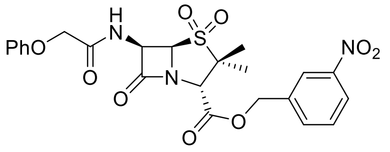

obtained from penicillin V ester **19i** (300 mg, 0.62 mmol), following column chromatography (10 g Ultra cartridge; 36 mL/min; initially, 100% cyclohexane (3 CV), followed by a linear gradient (20 CV): 0%→100% ethyl acetate in cyclohexane). Clear colorless oil; ^{1}H NMR (600 MHz, 300 K, $CDCl_3$): δ = 8.28−8.27 (m, 2H), 8.16 (d, J = 10.6 Hz, 1H), 7.74 (d, J = 7.6 Hz, 1H), 7.65−7.62 (m, 1H), 7.33 (t, J = 8.0 Hz, 2H), 7.05 (t, J = 7.4 Hz, 1H), 6.94 (d, J = 8.1 Hz, 2H), 6.21 (dd, J = 10.6, 4.6 Hz, 1H), 5.37−5.33 (m, 2H), 4.82 (d, J = 4.6 Hz, 1H), 4.61−4.55 (m, 3H), 1.62 (s, 3H), 1.36 ppm (s, 3H); ^{13}C NMR (150 MHz, 300 K, $CDCl_3$): δ = 173.5, 168.3, 166.4, 156.8, 148.5, 136.2, 134.5, 130.1, 129.7, 124.0, 123.5, 122.4, 114.8, 67.0, 66.8, 65.7, 64.7, 63.9, 56.3, 20.2, 17.9 ppm; IR (film): ṽ = 3403, 3076, 2980, 1806, 1759, 1699, 1599, 1530, 1495, 1460, 1441, 1352, 1322, 1290, 1211, 1170, 1117, 1065 cm^{-1}; HRMS (ESI): *m/z* calculated for $C_{23}H_{23}O_9N_3SNa$ $[M+Na]^+$: 540.1047, found: 540.1045; $[\alpha]_D^{25}$ = +84.9 (c = 0.5, $CHCl_3$).

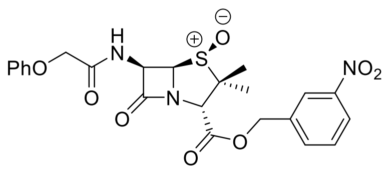

In addition to the penicillin V sulfone **20i**, the corresponding penicillin V sulfoxide **21i** (54 mg, 17%) was isolated in purified form as a single sulfoxide diastereomer. Note, the sulfoxide was tentatively assigned the (*S*)-configuration based on literature reports on peracid (including mCPBA)-mediated penicillin ester oxidations to sulfoxides.[11,20-21] White amorphous solid; ^{1}H NMR (600 MHz, 300 K, $CDCl_3$): δ = 8.28−8.25 (m, 3H), 7.76 (d, J = 7.6 Hz, 1H), 7.63 (t, J = 7.8 Hz, 1H), 7.33−7.31 (m, 2H), 7.03 (t, J = 7.4 Hz, 1H), 6.95 (d, J = 8.0 Hz, 2H), 6.14 (dd, J = 10.5, 4.8 Hz, 1H), 5.37 (d, J = 12.6 Hz, 1H), 5.33 (d, J = 12.5 Hz, 1H), 5.06 (d, J = 4.7 Hz, 1H), 4.76 (s, 1H), 4.56 (s, 2H), 1.74 (s, 3H), 1.18 ppm (s, 3H); ^{13}C NMR (150 MHz, 300 K, $CDCl_3$): δ = 173.2, 168.3, 167.7, 157.0, 148.5, 136.5, 134.5, 130.0, 129.7, 123.9, 123.4, 122.2, 114.9, 76.6, 75.3, 67.1, 66.5, 66.4, 55.6, 19.5, 18.7 ppm; IR (film): ṽ = 3371, 3069, 3042, 2971, 2937, 1794, 1753, 1692, 1599, 1530, 1494, 1459, 1441, 1351, 1292, 1206, 1158, 1132, 1081, 1062, 1039, 1022 cm^{-1}; HRMS (ESI): *m/z* calculated for $C_{23}H_{23}O_8N_3SNa$ $[M+Na]^+$: 524.1098, found: 524.1097; $[\alpha]_D^{25}$ =

+145.9 (c = 0.1, $CHCl_3$).

4-Nitrobenzyl (2*S*,5*R*,6*R*)-3,3-dimethyl-7-oxo-6-(2-phenoxyacetamido)-4-thia-1-azabicyclo[3.2.0]heptane-2-carboxylate 4,4-dioxide (20j). According to General Procedure B, penicillin V sulfone **20j** (200 mg, 53%) was obtained from penicillin V 4-nitrobenzyl ester (**19j**)[12] (334 mg, 0.73 mmol), following column chromatography (10 g Ultra cartridge; 36 mL/min; initially, 100% cyclohexane (3 CV), followed by a linear gradient (20 CV): 0%→100% ethyl acetate in cyclohexane). Clear colorless oil; ^{1}H NMR (600 MHz, 300 K, $CDCl_3$): δ = 8.29 (d, J = 8.6 Hz, 2H), 8.16 (d, J = 10.6 Hz, 1H), 7.57 (d, J = 8.5 Hz, 2H), 7.33 (t, J = 7.9 Hz, 2H), 7.05 (t, J = 7.4 Hz, 1H), 6.94 (d, J = 8.2 Hz, 2H), 6.21 (dd, J = 10.6, 4.6 Hz, 1H), 5.36 (d, J = 12.8 Hz, 1H), 5.33 (d, J = 12.8 Hz, 1H), 4.82 (d, J = 4.6 Hz, 1H), 4.61−4.55 (m, 3H), 1.62 (s, 3H), 1.36 ppm (s, 3H); ^{13}C NMR (150 MHz, 300 K, $CDCl_3$): δ = 173.5, 168.3, 166.3, 156.8, 148.2, 141.0, 129.8, 129.2, 124.1, 122.4, 114.8, 67.0, 66.7, 65.7, 64.7, 63.9, 56.3, 20.2, 17.9 ppm; IR (film): ṽ = 3400, 2980, 1804, 1759, 1697, 1600, 1520, 1494, 1460, 1440, 1346, 1321, 1289, 1210, 1168, 1115, 1064 cm^{-1}; HRMS (ESI): *m/z* calculated for $C_{23}H_{24}O_9N_3S$ [M+H]$^+$: 518.1228, found: 518.1226; $[\alpha]_D^{25}$ = +78.5 (c = 0.5, $CHCl_3$).

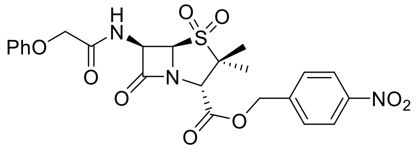

In addition to the penicillin V sulfone **20j**, the corresponding penicillin V sulfoxide **21j** (120 mg, 33%) was isolated in purified form as a single sulfoxide diastereomer. The analytical data are in agreement with those reported.[12] White amorphous solid; ^{1}H NMR (600 MHz, 300 K, $CDCl_3$): δ = 8.29 (d, J = 8.5 Hz, 2H), 8.26 (d, J = 10.6 Hz, 1H), 7.58 (d, J = 8.5 Hz, 2H), 7.32 (t, J = 7.9 Hz, 2H), 7.04 (t, J = 7.4 Hz, 1H), 6.96 (d, J = 8.1 Hz, 2H), 6.14 (dd, J = 10.5, 4.6 Hz, 1H), 5.37 (d, J = 12.9 Hz, 1H), 5.33 (d, J = 12.9 Hz, 1H), 5.06 (d, J = 4.7 Hz, 1H), 4.76 (s, 1H), 4.57 (s, 2H), 1.73 (s, 3H), 1.17 ppm (s, 3H); ^{13}C NMR (150 MHz, 300 K, $CDCl_3$): δ = 173.2, 168.3, 167.7, 157.0, 148.2, 141.4, 129.7, 129.1, 124.1, 122.2, 114.9, 76.6, 75.3, 67.1, 66.4, 66.3(6), 55.6, 19.5, 18.7 ppm; IR (film): ṽ = 2981, 1794, 1750, 1687, 1600, 1558, 1520, 1492, 1458, 1438, 1374, 1346, 1291, 1239, 1208, 1110, 1063, 1039, 1019 cm^{-1}; HRMS (ESI): *m/z* calculated for $C_{23}H_{24}O_8N_3S$ [M+H]$^+$: 502.1279, found: 502.1277; $[\alpha]_D^{25}$ = +140.9 (c = 0.5, $CHCl_3$).

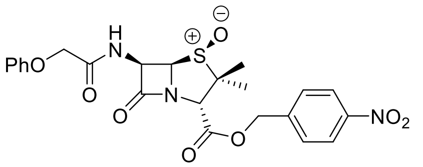

4-Methoxybenzyl (2*S*,5*R*,6*R*)-3,3-dimethyl-7-oxo-6-(2-phenoxyacetamido)-4-thia-1-azabicyclo[3.2.0]heptane-2-carboxylate (19k). According to General Procedure A, penicillin V ester **19k** (227 mg, 34%) was obtained from commercially-sourced 4-methoxybenzylbromide and penicillin V potassium salt (540 mg, 1.4 mmol), following column chromatography (10 g Sfär cartridge; 40 mL/min; initially, 100% cyclohexane (3 CV), followed by a linear gradient (20 CV): 0%→50% ethyl acetate in cyclohexane). Clear colorless oil; ^{1}H NMR (600 MHz, 300 K, $CDCl_3$): δ = 7.36−7.31 (m, 5H), 7.05 (t, J = 7.4 Hz, 1H), 6.95−6.93 (m, 2H), 6.92−6.90 (m, 2H), 5.75 (dd, J = 9.2, 4.3 Hz, 1H), 5.59 (d, J = 4.3 Hz, 1H), 5.17 (d, J = 11.8 Hz, 1H), 5.13 (d, J = 11.8 Hz, 1H), 4.59 (d, J = 15.1 Hz, 1H), 4.55 (d, J = 15.2 Hz, 1H), 4.48 (s, 1H), 3.84 (s, 3H), 1.57 (s, 3H), 1.41 ppm (s, 3H); ^{13}C NMR (150 MHz, 300 K, $CDCl_3$): δ = 173.0, 167.8, 167.5, 160.0, 156.9, 130.6, 129.8, 126.8, 122.4, 114.8, 114.1, 70.4, 67.8, 67.4, 67.2, 64.9, 58.1, 55.3, 32.0, 26.8 ppm; IR (film): ṽ = 3369, 2969, 2937, 1785, 1742, 1694, 1613, 1600, 1516, 1495, 1458, 1373, 1302, 1247, 1205, 1175, 1156, 1130, 1082, 1061, 1030 cm^{-1}; HRMS (ESI): *m/z* calculated for $C_{24}H_{26}O_6N_2SNa$ [M+Na]$^+$: 493.1404, found: 493.1398.

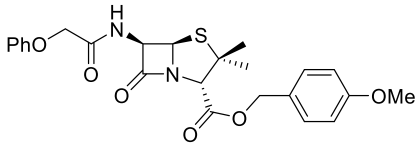

4-Methoxybenzyl (2*S*,5*R*,6*R*)-3,3-dimethyl-7-oxo-6-(2-phenoxyacetamido)-4-thia-1-azabicyclo[3.2.0]heptane-2-carboxylate 4,4-dioxide (20k). According to General Procedure B, penicillin V sulfone **20k** (123 mg, 52%) was obtained from penicillin V ester **19k** (220 mg, 0.47 mmol), following column chromatography (10 g Sfär cartridge; 40 mL/min; initially, 100% cyclohexane (3 CV), followed by a linear gradient (20 CV): 0%→100% ethyl acetate in cyclohexane). Clear colorless oil; ^{1}H NMR (600 MHz, 300 K, $CDCl_3$): δ = 8.16 (d, J = 10.6 Hz, 1H), 7.34−7.31 (m, 4H), 7.04 (t, J = 7.4 Hz, 1H), 6.95−6.92 (m, 4H), 6.18 (dd, J = 10.7, 4.6 Hz, 1H), 5.27 (d, J = 11.7 Hz, 1H), 5.13 (d, J = 11.7 Hz, 1H), 4.78 (d, J = 4.6 Hz, 1H), 4.60−4.54 (m, 2H), 4.53 (s, 1H), 3.84 (s, 3H), 1.56 (s, 3H), 1.26 ppm (s, 3H); ^{13}C NMR (150 MHz, 300 K, $CDCl_3$): δ = 173.5, 168.3, 166.4, 160.2, 156.8, 130.8, 129.7, 126.4, 122.3, 114.9, 114.2, 68.2, 67.0, 65.7, 64.8, 63.9, 56.2, 55.3, 20.0, 17.9 ppm; IR (film): ṽ = 3403, 2980, 1806, 1754, 1699, 1613, 1600, 1516, 1495, 1462, 1441, 1377, 1322, 1303, 1290, 1246, 1211, 1170, 1116, 1065, 1031 cm^{-1}; HRMS (ESI): *m/z* calculated for $C_{24}H_{26}O_8N_2SNa$ $[M+Na]^+$: 525.1302, found: 525.1299; $[\alpha]_D^{25}$ = +87.7 (c = 1.25, $CHCl_3$).

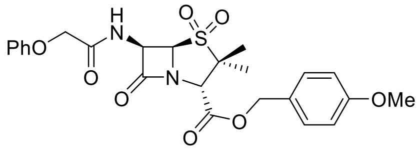

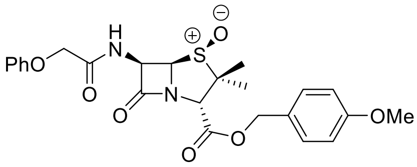

In addition to the penicillin V sulfone **20k**, the corresponding penicillin V sulfoxide **21k** (16 mg, 7%) was isolated in purified form as a single sulfoxide diastereomer. Note, the sulfoxide was tentatively assigned the (*S*)-configuration based on literature reports on peracid (including mCPBA)-mediated penicillin ester oxidations to sulfoxides.[11,20-21] Clear colorless oil; ^{1}H NMR (600 MHz, 300 K, $CDCl_3$): δ = 8.26 (d, J = 10.5 Hz, 1H), 7.35−7.30 (m, 4H), 7.03 (t, J = 7.3 Hz, 1H), 6.96−6.91 (m, 4H), 6.11 (dd, J = 10.5, 4.6 Hz, 1H), 5.27 (d, J = 11.7 Hz, 1H), 5.12 (d, J = 11.7 Hz, 1H), 5.03 (d, J = 4.6 Hz, 1H), 4.69 (s, 1H), 4.55 (s, 2H), 3.84 (s, 3H), 1.68 (s, 3H), 1.08 ppm (s, 3H); ^{13}C NMR (150 MHz, 300 K, $CDCl_3$): δ = 173.1, 168.2, 167.7, 160.1, 157.0, 130.8, 129.7, 126.8, 122.2, 114.9, 114.2, 76.6, 75.4, 67.8, 67.1, 66.3, 55.5, 55.3, 19.5, 18.4 ppm; IR (film): ṽ = 3377, 2971, 2937, 1793, 1749, 1693, 1613, 1600, 1516, 1495, 1461, 1373, 1292, 1247, 1208, 1175, 1062, 1034, 949 cm^{-1}; HRMS (ESI): *m/z* calculated for $C_{24}H_{27}O_7N_2S$ $[M+H]^+$: 487.1533, found: 487.1531; $[\alpha]_D^{25}$ = +126.4 (c = 0.1, $CHCl_3$).

Naphthalen-2-ylmethyl (2*S*,5*R*,6*R*)-3,3-dimethyl-7-oxo-6-(2-phenoxyacetamido)-4-thia-1-azabicyclo[3.2.0]heptane-2-carboxylate (19l). According to General Procedure A, penicillin V ester **19l** (284 mg, 41%) was obtained from commercially-sourced 2-(bromomethyl)naphthalene and penicillin V potassium salt (540 mg, 1.4 mmol), following column chromatography (25 g KP-Sil cartridge; 50 mL/min; initially, 100% cyclohexane (3 CV), followed by a linear gradient (15 CV): 0%→80% ethyl acetate in cyclohexane). Clear colorless oil; ^{1}H NMR (600 MHz, 300 K, $CDCl_3$): δ = 7.89−7.87 (m, 4H), 7.55−7.53 (m, 2H), 7.48 (dd, J = 8.4, 1.2 Hz, 1H), 7.35−7.33 (m, 3H), 7.05 (t, J = 7.4 Hz, 1H), 6.94 (d, J = 8.5 Hz, 2H), 5.76 (dd, J = 9.2, 4.3 Hz, 1H), 5.61 (d, J = 4.3 Hz, 1H), 5.40−5.36 (m, 2H), 4.59 (d, J = 15.2 Hz, 1H), 4.55 (d, J = 15.2 Hz, 1H), 4.54 (s, 1H), 1.59 (s, 3H), 1.44 ppm (s, 3H); ^{13}C NMR (150 MHz, 300 K, $CDCl_3$): δ = 173.0, 167.8, 167.5, 156.9, 133.3, 133.1, 132.0, 129.8, 128.7, 128.2, 128.1, 127.8, 126.6, 126.5, 126.0, 122.4, 114.8, 70.5, 67.8, 67.7, 67.2, 64.9, 58.1, 32.0, 26.8 ppm; IR (film): ṽ = 3369, 3056, 2971, 1785, 1745, 1694, 1599, 1517, 1494, 1458, 1440, 1297, 1241, 1204, 1181, 1155, 1127, 1082, 1061, 1028 cm^{-1}; HRMS (ESI): *m/z* calculated for $C_{27}H_{26}O_5N_2SNa$ $[M+Na]^+$: 513.1455, found: 513.1450.

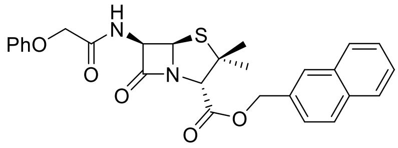

Naphthalen-2-ylmethyl (2*S*,5*R*,6*R*)-3,3-dimethyl-7-oxo-6-(2-phenoxyacetamido)-4-thia-1-azabicyclo[3.2.0]heptane-2-carboxylate 4,4-dioxide (20l). According to General Procedure B, penicillin V sulfone **20l** (140 mg, 53%) was obtained from penicillin V ester **19l** (250 mg, 0.51 mmol), following column chromatography (10 g Ultra cartridge; 36 mL/min; initially, 100% cyclohexane (3 CV), followed by a linear gradient (20 CV): 0%→100% ethyl acetate in cyclohexane). Clear colorless oil; ^{1}H NMR (600 MHz, 300 K, $CDCl_3$): δ = 8.17 (d, J = 10.6 Hz, 1H), 7.91−7.88 (m, 4H), 7.56−7.55 (m, 2H), 7.48 (d, J = 8.4 Hz, 1H), 7.32 (t, J = 8.0 Hz, 2H), 7.04 (t, J = 7.4 Hz, 1H), 6.94 (d, J = 8.0 Hz, 2H), 6.19 (dd, J = 10.7, 4.6 Hz, 1H), 5.49 (d, J = 11.9 Hz, 1H), 5.37 (d, J = 11.9 Hz, 1H), 4.79 (d, J = 4.6 Hz, 1H), 4.60−4.55 (m, 3H), 1.58 (s, 3H), 1.29 ppm (s, 3H); ^{13}C NMR (150 MHz, 300 K, $CDCl_3$): δ = 173.5, 168.3, 166.5, 156.8, 133.4, 133.1, 131.5, 129.7, 128.9, 128.6, 128.1, 127.8, 126.9, 126.7, 126.0, 122.3, 114.9, 68.6, 67.0, 65.7, 64.8, 64.0, 56.2, 20.1, 17.9 ppm; IR (film): ṽ = 3402, 3059, 2981, 1806, 1755, 1699, 1600, 1519, 1495, 1461, 1440, 1322, 1288, 1241, 1210, 1171, 1116, 1065 cm^{-1}; HRMS (ESI): *m/z* calculated for $C_{27}H_{27}O_7N_2S$ $[M+H]^+$: 523.1533, found: 523.1533; $[\alpha]_D^{25}$ = +88. (c = 1.0, $CHCl_3$).

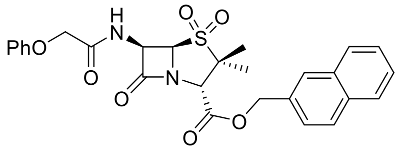

In addition to the penicillin V sulfone **20l**, the corresponding penicillin V sulfoxide **21l** (68 mg, 26%) was isolated in purified form as a single sulfoxide diastereomer. Note, the sulfoxide was tentatively assigned the (*S*)-configuration based on literature reports on peracid (including mCPBA)-mediated penicillin ester oxidations to sulfoxides.[11,20-21] Clear colorless oil; ^{1}H NMR (600 MHz, 300 K, $CDCl_3$): δ = 8.26 (d, J = 10.5 Hz, 1H), 7.90−7.87 (m, 4H), 7.56−7.54 (m, 2H), 7.49 (dd, J = 8.4, 1.3 Hz, 1H), 7.31 (t, J = 8.0 Hz, 2H), 7.03 (t, J = 7.4 Hz, 1H), 6.96−6.94 (m, 2H), 6.13 (dd, J = 10.5, 4.6 Hz, 1H), 5.50 (d, J = 12.0 Hz, 1H), 5.36 (d, J = 12.0 Hz, 1H), 5.04 (d, J = 4.6 Hz, 1H), 4.75 (s, 1H), 4.56 (s, 2H), 1.70 (s, 3H), 1.09 ppm (s, 3H); ^{13}C NMR (150 MHz, 300 K, $CDCl_3$): δ = 173.1, 168.2, 167.8, 157.0, 133.3, 133.1, 131.9, 129.7, 128.8, 128.5, 128.1, 127.8, 126.8, 126.6, 126.1, 122.2, 114.9, 76.6, 75.4, 68.2, 67.1, 66.4, 55.5, 19.5, 18.5 ppm; IR (film): ṽ = 3378, 3058, 2979, 1794, 1750, 1692, 1599, 1515, 1494, 1462, 1441, 1392, 1370, 1344, 1291, 1241, 1206, 1157, 1130, 1062, 1039, 1021 cm^{-1}; HRMS (ESI): *m/z* calculated for $C_{27}H_{27}O_6N_2S$ $[M+H]^+$: 507.1584, found: 507.1583; $[\alpha]_D^{25}$ = +118.0 (c = 0.5, $CHCl_3$).

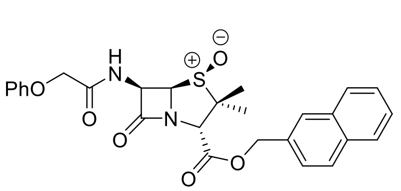

(5-(Trifluoromethyl)furan-2-yl)methyl (2*S*,5*R*,6*R*)-3,3-dimethyl-7-oxo-6-(2-phenoxyacetamido)-4-thia-1-azabicyclo[3.2.0]heptane-2-carboxylate (19m). According to General Procedure A, penicillin V ester **19m** (72 mg, 10%) was obtained from commercially-sourced 2-(bromomethyl)-5-(trifluoromethyl)furan and penicillin V potassium salt (540 mg, 1.4 mmol), following column chromatography (5 g Sfär cartridge; 18 mL/min; initially, 100% cyclohexane (3 CV), followed by a linear gradient (20 CV): 0%→100% ethyl acetate in cyclohexane). Clear colorless oil; ^{1}H NMR (600 MHz, 300 K, $CDCl_3$): δ = 7.36−7.32 (m, 3H), 7.06 (t, J = 7.4 Hz, 1H), 6.95−6.94 (m, 2H), 6.80−6.79 (m, 1H), 6.55 (d, J = 3.3 Hz, 1H), 5.77 (dd, J = 9.2, 4.3 Hz, 1H), 5.59 (d, J = 4.3 Hz, 1H), 5.27 (d, J = 13.3 Hz, 1H), 5.15 (d, J = 13.3 Hz, 1H), 4.59 (d, J = 15.2 Hz, 1H), 4.56 (d, J = 15.2 Hz, 1H), 4.50 (s, 1H), 1.60 (s, 3H), 1.41 ppm (s, 3H); ^{19}F NMR (565 MHz, 300 K, $CDCl_3$): δ = −64.4 ppm (s, 3F); ^{13}C NMR (150 MHz, 300 K, $CDCl_3$): δ = 173.1, 167.8, 167.1, 156.9, 151.0, 142.7 (q, J = 42.9 Hz), 129.8, 122.4, 118.7 (q, J = 267.3 Hz), 114.8, 112.4 (q, J = 2.9 Hz), 112.1, 70.2, 67.7, 67.2, 64.8, 58.1, 58.0(7), 31.6, 26.5 ppm; IR (film): ṽ = 3373, 2970, 2931, 1785, 1751, 1692, 1600, 1565, 1518, 1494, 1441, 1388, 1317, 1177, 1132, 1109, 1083, 1061, 1023 cm^{-1}; HRMS (ESI): *m/z* calculated for $C_{22}H_{21}O_6N_2F_3SNa$ $[M+Na]^+$: 521.0965, found: 521.0964.

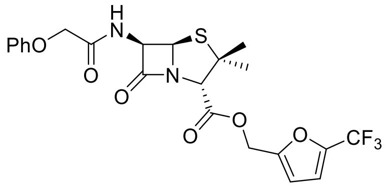

(5-(Trifluoromethyl)furan-2-yl)methyl (2*S*,5*R*,6*R*)-3,3-dimethyl-7-oxo-6-(2-phenoxyacetamido)-4-thia-1-azabicyclo[3.2.0]heptane-2-carboxylate 4,4-dioxide (20m). According to General Procedure B, penicillin V sulfone **20m** (29 mg, 39%) was obtained from penicillin V ester **19m** (70 mg, 0.14 mmol), following column chromatography (5 g Sfär cartridge; 18 mL/min; initially, 100% cyclohexane (3 CV), followed by a linear gradient (20 CV): 0%→100% ethyl acetate in cyclohexane). Clear colorless oil; ^{1}H NMR (600 MHz, 300 K, $CDCl_3$): δ = 8.16 (d, J = 10.6 Hz, 1H), 7.34−7.32 (m, 2H), 7.05 (t, J = 7.4 Hz, 1H), 6.94 (d, J = 8.0 Hz, 2H), 6.82 (d, J = 2.5 Hz, 1H), 6.58 (d, J = 3.3 Hz, 1H), 6.20 (dd, J = 10.7, 4.6 Hz, 1H), 5.35 (d, J = 13.3 Hz, 1H), 5.18 (d, J = 13.3 Hz, 1H), 4.81 (d, J = 4.6 Hz, 1H), 4.59 (d, J = 15.2 Hz, 1H), 4.57 (s, 1H), 4.56 (d, J = 15.1 Hz, 1H), 1.60 (s, 3H), 1.32 ppm (s, 3H); ^{19}F NMR (565 MHz, 300 K, $CDCl_3$): δ = −64.4 ppm (s, 3F); ^{13}C NMR (150 MHz, 300 K, $CDCl_3$): δ = 173.5, 168.3, 166.1, 156.8, 150.5, 142.9 (q, J = 43.2 Hz), 129.8, 122.4, 118.6 (q, J = 266.4 Hz), 114.9, 112.5 (q, J = 2.7 Hz), 112.4, 67.0, 65.7, 64.7, 63.8, 58.8, 56.3, 19.9, 17.7 ppm; IR (film): ṽ = 3402, 2980, 1806, 1762, 1698, 1600, 1566, 1519, 1495, 1461, 1440, 1387, 1317, 1288, 1217, 1173, 1136, 1111, 1082, 1064, 1023 cm^{-1}; HRMS (ESI): *m/z* calculated for $C_{22}H_{21}O_8N_2F_3SNa$ $[M+Na]^+$: 553.0863, found: 553.0861; $[\alpha]_D^{25}$ = +90.3 (c = 0.15, $CHCl_3$).

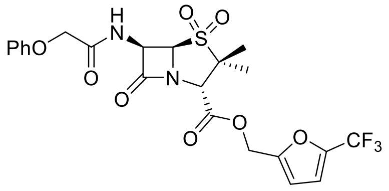

In addition to the penicillin V sulfone **20m**, the corresponding penicillin V sulfoxide **21m** (9 mg, 12%) was isolated in purified form as a single sulfoxide diastereomer. Note, the sulfoxide was tentatively assigned the (*S*)-configuration based on literature reports on peracid (including mCPBA)-mediated penicillin ester oxidations to sulfoxides.[11,20-21] Clear colorless oil; ^{1}H NMR (600 MHz, 300 K, $CDCl_3$): δ = 8.26 (d, J = 10.5 Hz, 1H), 7.33−7.30 (m, 2H), 7.03 (t, J = 7.4 Hz, 1H), 6.95 (d, J = 7.9 Hz, 2H), 6.81 (d, J = 2.5 Hz, 1H), 6.57 (d, J = 3.4 Hz, 1H), 6.13 (dd, J = 10.5, 4.7 Hz, 1H), 5.35 (d, J = 13.3 Hz, 1H), 5.17 (d, J = 13.3 Hz, 1H), 5.05 (d, J = 4.7 Hz, 1H), 4.72 (s, 1H), 4.56 (s, 2H), 1.71 (s, 3H), 1.14 ppm (s, 3H); ^{19}F NMR (565 MHz, 300 K, $CDCl_3$): δ = −64.4 ppm (s, 3F); ^{13}C NMR (150 MHz, 300 K, $CDCl_3$): δ = 173.1, 168.2, 167.4, 157.0, 150.9, 142.8 (q, J = 42.5 Hz), 129.7, 122.2, 118.7 (q, J = 267.1 Hz), 114.9, 112.5 (q, J = 2.6 Hz), 112.1, 75.4, 67.1, 66.3, 58.5, 55.5, 53.4, 19.3, 18.3 ppm; IR (film): ṽ = 3367, 3139, 2978, 2935, 1793, 1757, 1692, 1600, 1590, 1565, 1518, 1496, 1465, 1441, 1388, 1373, 1347, 1318, 1291, 1184, 1133, 1109, 1081, 1062, 1038, 1023 cm^{-1}; HRMS (ESI): *m/z* calculated for $C_{22}H_{22}O_7N_2F_3S$ $[M+H]^+$: 515.1094, found: 515.1094; $[\alpha]_D^{25}$ = +179.3 (c = 0.05, $CHCl_3$).

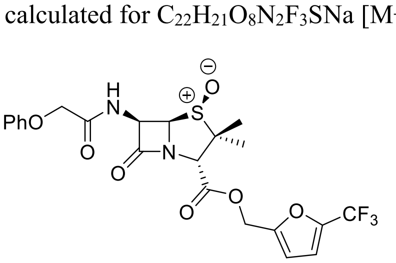

(5-Methylisoxazol-3-yl)methyl (2*S*,5*R*,6*R*)-3,3-dimethyl-7-oxo-6-(2-phenoxyacetamido)-4-thia-1-azabicyclo[3.2.0]heptane-2-carboxylate 4,4-dioxide (20n). According to General Procedure B, penicillin V sulfone **20n** (51 mg, 53%) was obtained from (5-methylisoxazol-3-yl)methyl (2*S*,5*R*,6*R*)-3,3-dimethyl-7-oxo-6-(2-phenoxyacetamido)-4-thia-1-azabicyclo[3.2.0]heptane-2-carboxylate (**19n**)[15] (86 mg, 0.20 mmol), following column chromatography (5 g Sfär cartridge; 18 mL/min; initially, 100% cyclohexane (3 CV), followed by a linear gradient (20 CV): 0%→100% ethyl acetate in cyclohexane). Clear colorless oil; ^{1}H NMR (600 MHz, 300 K, $CDCl_3$): δ = 8.17 (d, J = 10.7 Hz, 1H), 7.33 (t, J = 8.0 Hz, 2H), 7.05 (t, J = 7.4 Hz, 1H), 6.95−6.94 (m, 2H), 6.20 (dd, J = 10.7, 4.6 Hz, 1H), 6.05 (s, 1H), 5.35 (d, J = 13.0 Hz, 1H), 5.23 (d, J = 12.9 Hz, 1H), 4.82 (d, J = 4.6 Hz, 1H), 4.61−4.55 (m, 3H), 2.47 (s, 3H), 1.64 (s, 3H), 1.43 ppm (s, 3H); ^{13}C NMR (150 MHz, 300 K, $CDCl_3$): δ = 173.5, 170.9, 168.3, 166.3, 158.2, 156.8, 129.8, 122.4, 114.9, 101.1, 67.0, 65.6, 64.8, 63.9, 59.2, 56.3, 20.2, 17.7, 12.3 ppm; IR (film): ṽ = 3400, 2980, 1806, 1763, 1699, 1601, 1520, 1494, 1458, 1439, 1322, 1289, 1210, 1171, 1117, 1065 cm^{-1}; HRMS (ESI): *m/z* calculated for $C_{21}H_{23}O_8N_3SNa$ $[M+Na]^+$: 500.1098, found: 500.1096; $[\alpha]_D^{25}$ = +94.7 (c = 0.1, $CHCl_3$).

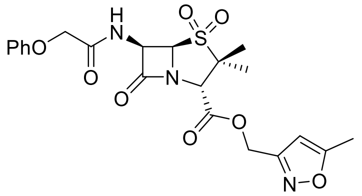

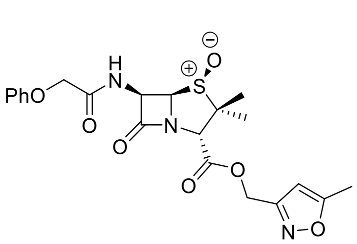

In addition to the penicillin V sulfone **20n**, the corresponding penicillin V sulfoxide **21n** (16 mg, 17%) was isolated in purified form as a single sulfoxide diastereomer. Note, the sulfoxide was tentatively assigned the (*S*)-configuration based on literature reports on peracid (including mCPBA)-mediated penicillin ester oxidations to sulfoxides.[11,20-21] Clear colorless oil; ^{1}H NMR (600 MHz, 300 K, $CDCl_3$): δ = 8.27 (d, *J* = 10.4 Hz, 1H), 7.32 (t, *J* = 7.6 Hz, 2H), 7.05−7.02 (m, 1H), 6.96 (d, *J* = 8.6 Hz, 2H), 6.13 (dd, *J* = 10.5, 4.6 Hz, 1H), 6.06 (s, 1H), 5.35 (d, *J* = 13.0 Hz, 1H), 5.22 (d, *J* = 13.0 Hz, 1H), 5.06 (d, *J* = 4.7 Hz, 1H), 4.75 (s, 1H), 4.57 (s, 2H), 2.47 (s, 3H), 1.75 (s, 3H), 1.25 ppm (s, 3H); ^{13}C NMR (150 MHz, 300 K, $CDCl_3$): δ = 173.2, 170.7, 168.3, 167.6, 158.5, 157.0, 129.7, 122.2, 114.9, 101.2, 76.6, 75.5, 67.1, 66.3, 59.0, 55.5, 19.4, 18.6, 12.3 ppm; IR (film): ṽ = 3373, 2979, 2932, 1794, 1758, 1693, 1601, 1519, 1493, 1458, 1439, 1372, 1291, 1242, 1206, 1134, 1061, 1044 cm^{-1}; HRMS (ESI): *m/z* calculated for $C_{21}H_{23}O_7N_3SNa$ $[M+Na]^+$: 484.1149, found: 484.1149.

Methyl (2*S*,5*R*,6*R*)-3,3-dimethyl-7-oxo-6-(2-phenoxyacetamido)-4-thia-1-azabicyclo[3.2.0]heptane-2-carboxylate 4,4-dioxide (20o). According to General Procedure B, penicillin V sulfone **20o** (363 mg, 55%) was

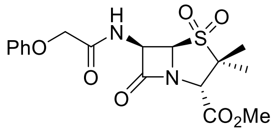

obtained from penicillin V methyl ester (**19o**)[22] (607 mg, 1.67 mmol), following column chromatography (10 g Sfär cartridge; 40 mL/min; initially, 100% cyclohexane (3 CV), followed by a linear gradient (20 CV): 0%→100% ethyl acetate in cyclohexane). White amorphous solid; ^{1}H NMR (600 MHz, 300 K, $CDCl_3$): δ = 8.18 (d, *J* = 10.6 Hz, 1H), 7.33 (t, *J* = 7.5 Hz, 2H), 7.05 (t, *J* = 7.3 Hz, 1H), 6.95 (d, *J* = 8.2 Hz, 2H), 6.20 (dd, *J* = 10.6, 4.5 Hz, 1H), 4.82 (d, *J* = 4.6 Hz, 1H), 4.58 (s, 2H), 4.55 (s, 1H), 3.86 (s, 3H), 1.64 (s, 3H), 1.43 ppm (s, 3H); ^{13}C NMR (150 MHz, 300 K, $CDCl_3$): δ = 173.5, 168.3, 167.0, 156.8, 129.7, 122.3, 114.9, 67.0, 65.7, 64.7, 64.0, 56.3, 53.2 (app. d, *J* = 4.3 Hz), 20.3, 17.9 ppm; IR (film): ṽ = 3403, 2981, 2958, 1806, 1758, 1699, 1599, 1520, 1494, 1459, 1438, 1321, 1290, 1220, 1172, 1116, 1065 cm^{-1}; HRMS (ESI): *m/z* calculated for $C_{17}H_{21}O_7N_2S$ $[M+H]^+$: 397.1064, found: 397.1064; $[\alpha]_D^{25}$ = +96.1 (c = 1.25, $CHCl_3$).

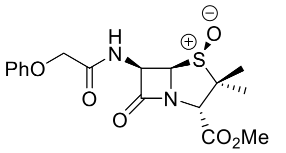

In addition to the penicillin V sulfone **20o**, the corresponding penicillin V sulfoxide **21o** (100 mg, 17%) was isolated in purified form as a single sulfoxide diastereomer. The analytical data are in agreement with those reported.[22] Clear colorless oil; ^{1}H NMR (600 MHz, 300 K, $CDCl_3$): δ = 8.28 (brd, *J* = 10.4 Hz, 1H), 7.33−7.31 (m, 2H), 7.05−7.02 (m, 1H), 6.97−6.95 (m, 2H), 6.13 (dd, *J* = 10.5, 4.7 Hz, 1H), 5.06 (d, *J* = 4.7 Hz, 1H), 4.71 (s, 1H), 4.56 (s, 2H), 3.85 (s, 3H), 1.76 (s, 3H), 1.25 ppm (s, 3H); ^{13}C NMR (150 MHz, 300 K, $CDCl_3$): δ = 173.1, 168.3, 168.2, 157.0, 129.7, 122.2, 114.9, 76.5, 75.3, 67.1, 66.5, 55.5, 53.0, 19.4, 18.7 ppm; IR (film): ṽ = 3373, 3066, 3044, 2970, 2933, 1785, 1747, 1694, 1619, 1599, 1590, 1518, 1494, 1457, 1373, 1297, 1237, 1205, 1180, 1155, 1130, 1082, 1061, 1027, 963 cm^{-1}; HRMS (ESI): *m/z* calculated for $C_{17}H_{20}O_6N_2SNa$ $[M+Na]^+$: 403.0934, found: 403.0934; $[\alpha]_D^{25}$ = +168.5 (c = 1.0, $CHCl_3$).

Ethyl (2*S*,5*R*,6*R*)-3,3-dimethyl-7-oxo-6-(2-phenoxyacetamido)-4-thia-1-azabicyclo[3.2.0]heptane-2-carboxylate 4,4-dioxide (20p). According to General Procedure B, penicillin V sulfone **20p** (87 mg, 42%) was

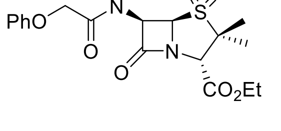

obtained from penicillin V ethyl ester (**19p**)[23] (190 mg, 0.5 mmol), following column chromatography (5 g Sfär cartridge; 18 mL/min; initially, 100% cyclohexane (3 CV), followed by a linear gradient (20 CV): 0%→100% ethyl acetate in cyclohexane). Clear colorless oil; ^{1}H NMR (600 MHz, 300 K, $CDCl_3$): δ = 8.18 (d, *J* = 10.6 Hz, 1H), 7.33 (t, *J* = 7.7 Hz, 2H), 7.06−7.04 (m, 1H), 6.95 (d, *J* = 8.5 Hz, 2H), 6.20 (dd, *J* = 10.6, 4.6 Hz, 1H), 4.82 (d, *J* = 4.6 Hz, 1H), 4.58 (s, 2H), 4.53 (s, 1H), 4.32 (q, *J* = 7.1 Hz, 2H), 1.65 (s, 3H), 1.44 (s, 3H), 1.36 ppm (t, *J* = 7.1 Hz, 3H); ^{13}C NMR (150 MHz, 300 K, $CDCl_3$): δ = 173.5, 168.3, 166.5, 156.9, 129.8, 122.3, 114.9, 67.0, 65.7, 64.7, 64.0, 62.7, 56.2, 20.3, 18.0, 14.2 ppm; IR (film): ṽ = 3406, 3069, 2984, 2946, 1805, 1754, 1700, 1600,

1520, 1496, 1465, 1442, 1373, 1321, 1291, 1216, 1175, 1117, 1081, 1066, 1008 cm^{-1}; HRMS (ESI): *m/z* calculated for $C_{18}H_{22}O_7N_2SNa$ $[M+Na]^+$: 433.1040, found: 433.1040; $[\alpha]_D^{25}$ = +91.4 (c = 0.5, $CHCl_3$).

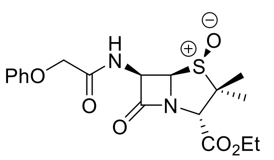

In addition to the penicillin V sulfone **20p**, the corresponding penicillin V sulfoxide **21p** (54 mg, 27%) was isolated in purified form as a single sulfoxide diastereomer. Note, the sulfoxide was tentatively assigned the (*S*)-configuration based on literature reports on peracid (including mCPBA)-mediated penicillin ester oxidations to sulfoxides.[11,20-21] Clear colorless oil; ^{1}H NMR (600 MHz, 300 K, $CDCl_3$): δ = 8.28 (brd, *J* = 10.4 Hz, 1H), 7.32 (t, *J* = 7.8 Hz, 2H), 7.03 (t, *J* = 7.3 Hz, 1H), 6.96 (d, *J* = 8.2 Hz, 2H), 6.13 (dd, *J* = 10.5, 4.6 Hz, 1H), 5.06 (d, *J* = 4.6 Hz, 1H), 4.69 (s, 1H), 4.57 (s, 2H), 4.34−4.29 (m, 2H), 1.76 (s, 3H), 1.36 (t, *J* = 7.1 Hz, 3H), 1.26 ppm (s, 3H); ^{13}C NMR (150 MHz, 300 K, $CDCl_3$): δ = 173.2, 168.2, 167.8, 157.0, 129.7, 122.2, 114.9, 76.6, 75.3, 67.1, 66.4, 62.3, 55.5, 19.5, 18.7, 14.2 ppm; IR (film): ṽ = 3370, 3059, 3043, 2977, 2938, 1793, 1749, 1691, 1599, 1590, 1518, 1496, 1465, 1443, 1395, 1371, 1347, 1293, 1211, 1174, 1133, 1080, 1062, 1038, 1015 cm^{-1}; HRMS (ESI): *m/z* calculated for $C_{18}H_{22}O_6N_2SNa$ $[M+Na]^+$: 417.1091, found: 417.1090; $[\alpha]_D^{25}$ = +182.4 (c = 0.2, $CHCl_3$).

Propinyl (2*S*,5*R*,6*R*)-3,3-dimethyl-7-oxo-6-(2-phenoxyacetamido)-4-thia-1-azabicyclo[3.2.0]heptane-2-carboxylate (19q). According to General Procedure A, penicillin V ester **19q** (123 mg, 23%) was obtained from

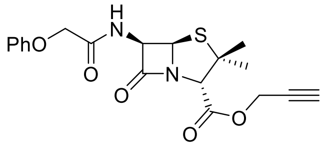

commercially-sourced propargyl bromide solution (80%$_{w/v}$ in toluene) and penicillin V potassium salt (540 mg, 1.4 mmol), following column chromatography (10 g Ultra cartridge; 36 mL/min; initially, 100% cyclohexane (3 CV), followed by a linear gradient (20 CV): 0%→100% ethyl acetate in cyclohexane). Clear colorless oil; ^{1}H NMR (600 MHz, 300 K, $CDCl_3$): δ = 7.37−7.33 (m, 3H), 7.06 (t, *J* = 7.4 Hz, 1H), 6.95 (d, *J* = 7.9 Hz, 2H), 5.78 (dd, *J* = 9.2, 4.3 Hz, 1H), 5.62 (d, *J* = 4.3 Hz, 1H), 4.85 (dd, *J* = 15.5, 2.4 Hz, 1H), 4.75 (dd, *J* = 15.5, 2.5 Hz, 1H), 4.60 (d, *J* = 15.1 Hz, 1H), 4.56 (d, *J* = 15.1 Hz, 1H), 4.51 (s, 1H), 2.54 (t, *J* = 2.4 Hz, 1H), 1.64 (s, 3H), 1.57 ppm (s, 3H); ^{13}C NMR (150 MHz, 300 K, $CDCl_3$): δ = 173.1, 167.8, 166.9, 156.9, 129.8, 122.4, 114.8, 76.5, 75.9, 70.2, 67.8, 67.2, 65.0, 58.1, 52.8, 31.7, 26.9 ppm; IR (film): ṽ = 3377, 3284, 2971, 2934, 1784, 1753, 1691, 1599, 1519, 1494, 1440, 1374, 1296, 1241, 1203, 1179, 1155, 1131, 1082, 1061, 1033, 989 cm^{-1}; HRMS (ESI): *m/z* calculated for $C_{19}H_{20}O_5N_2SNa$ $[M+Na]^+$: 411.0985, found: 411.0985.

Propinyl (2*S*,5*R*,6*R*)-3,3-dimethyl-7-oxo-6-(2-phenoxyacetamido)-4-thia-1-azabicyclo[3.2.0]heptane-2-carboxylate 4,4-dioxide (20q). According to General Procedure B, penicillin V sulfone **20q** (28.9 mg, 23%) was

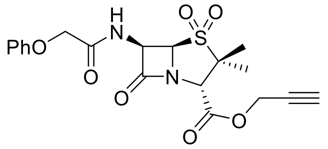

obtained from penicillin V ester **19q** (123 mg, 0.3 mmol), following column chromatography (5 g Sfär cartridge; 18 mL/min; initially, 100% cyclohexane (3 CV), followed by a linear gradient (20 CV): 0%→100% ethyl acetate in cyclohexane). White amorphous solid; ^{1}H NMR (600 MHz, 300 K, $CDCl_3$): δ = 8.17 (d, *J* = 10.6 Hz, 1H), 7.35−7.31 (m, 2H), 7.06−7.03 (m, 1H), 6.95−6.93 (m, 2H), 6.21 (dd, *J* = 10.7, 4.6 Hz, 1H), 4.93 (dd, *J* = 15.5, 2.5 Hz, 1H), 4.83 (d, *J* = 4.7 Hz, 1H), 4.76 (dd, *J* = 15.5, 2.5 Hz, 1H), 4.60−4.55 (m, 3H), 2.58 (t, *J* = 2.5 Hz, 1H), 1.66 (s, 3H), 1.47 ppm (s, 3H); ^{13}C NMR (150 MHz, 300 K, $CDCl_3$): δ = 173.4, 168.3, 165.9, 156.8, 129.7, 122.3, 114.8, 76.4, 76.1, 70.0, 66.7, 64.8, 63.7, 56.3, 53.5, 20.1, 17.8 ppm; IR (film): ṽ = 3402, 3286, 2980, 2128, 1805, 1764, 1697, 1599, 1519, 1494, 1463, 1439, 1375, 1322, 1288, 1208, 1168, 1116, 1064, 1031 cm^{-1}; HRMS (ESI): *m/z* calculated for $C_{19}H_{21}O_7N_2S$ $[M+H]^+$: 421.1064, found: 421.1063; $[\alpha]_D^{25}$ = +107.5 (c = 0.2, $CHCl_3$).

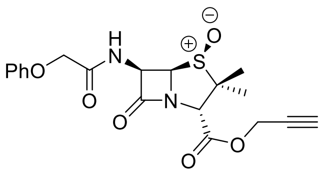

In addition to the penicillin V sulfone **20q**, the corresponding penicillin V sulfoxide **21q** (23.0 mg, 19%) was isolated in purified form as a single sulfoxide diastereomer. Note, the sulfoxide was tentatively assigned the (*S*)-configuration based on literature reports on peracid (including mCPBA)-mediated penicillin ester oxidations to sulfoxides.[11,20-21] Clear colorless oil; ^{1}H NMR (600 MHz, 300 K, $CDCl_3$): δ = 8.27 (d, J = 10.5 Hz, 1H), 7.34−7.31 (m, 2H), 7.05−7.02 (m, 1H), 6.97−6.95 (m, 2H), 6.14 (dd, J = 10.5, 4.7 Hz, 1H), 5.08 (d, J = 4.7 Hz, 1H), 4.93 (dd, J = 15.5, 2.4 Hz, 1H), 4.76 (dd, J = 15.5, 2.5 Hz, 1H), 4.73 (s, 1H), 4.57 (s, 2H), 2.56 (t, J = 2.5 Hz, 1H), 1.78 (s, 3H), 1.29 ppm (s, 3H); ^{13}C NMR (150 MHz, 300 K, $CDCl_3$): δ = 173.1, 168.3, 167.2, 157.0, 129.7, 122.2, 114.9, 76.7, 76.5, 76.1, 75.6, 67.1, 66.2, 55.6, 53.2, 19.5, 18.6 ppm; IR (film): ṽ = 3369, 3285, 2980, 2126, 1793, 1759, 1690, 1599, 1518, 1494, 1462, 1439, 1373, 1291, 1241, 1204, 1156, 1062, 1040, 1023, 986 cm^{-1}; HRMS (ESI): *m/z* calculated for $C_{19}H_{21}O_6N_2S$ $[M+H]^+$: 405.1115, found: 405.1116; $[\alpha]_D^{25}$ = +198.3 (c = 0.05, $CHCl_3$).

Cyanomethyl (2*S*,5*R*,6*R*)-3,3-dimethyl-7-oxo-6-(2-phenoxyacetamido)-4-thia-1-azabicyclo[3.2.0]heptane-2-carboxylate (19r). According to General Procedure A, penicillin V ester **19r** (180 mg, 33%) was obtained from

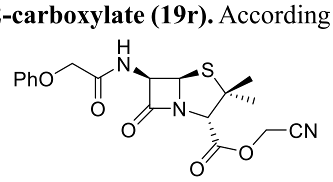

commercially-sourced bromoacetonitrile and penicillin V potassium salt (540 mg, 1.4 mmol), following column chromatography (10 g Sfär cartridge; 40 mL/min; initially, 100% cyclohexane (3 CV), followed by a linear gradient (20 CV): 0%→100% ethyl acetate in cyclohexane). Clear colorless oil; ^{1}H NMR (600 MHz, 300 K, $CDCl_3$): δ = 7.38−7.32 (m, 3H), 7.06 (t, J = 7.4 Hz, 1H), 6.96−6.93 (m, 2H), 5.80 (dd, J = 9.2, 4.3 Hz, 1H), 5.61 (d, J = 4.3 Hz, 1H), 4.90 (d, J = 15.6 Hz, 1H), 4.79 (d, J = 15.6 Hz, 1H), 4.60 (d, J = 15.2 Hz, 1H), 4.56 (d, J = 15.2 Hz, 1H), 4.54 (s, 1H), 1.65 (s, 3H), 1.57 ppm (s, 3H); ^{13}C NMR (150 MHz, 300 K, $CDCl_3$): δ = 173.1, 167.9, 166.3, 156.9, 129.9, 122.5, 114.8, 113.3, 70.0, 67.8, 67.1, 65.0, 58.1, 48.9, 31.2, 26.9 ppm; IR (film): ṽ = 3377, 3063, 3034, 2952, 2933, 1786, 1743, 1685, 1599, 1522, 1495, 1457, 1437, 1370, 1294, 1213, 1176, 1083, 1062 cm^{-1}; HRMS (ESI): *m/z* calculated for $C_{18}H_{19}O_5N_3SNa$ $[M+Na]^+$: 412.0938, found: 412.0937.

Cyanomethyl (2*S*,5*R*,6*R*)-3,3-dimethyl-7-oxo-6-(2-phenoxyacetamido)-4-thia-1-azabicyclo[3.2.0]heptane-2-carboxylate 4,4-dioxide (20r). According to General Procedure B, penicillin V sulfone **20r** (98 mg, 51%) was

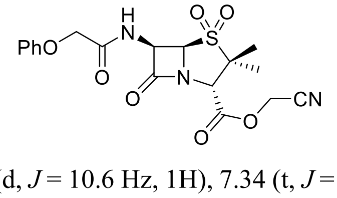

obtained from penicillin V ester **19r** (180 mg, 0.46 mmol), following column chromatography (10 g Sfär cartridge; 40 mL/min; initially, 100% cyclohexane (3 CV), followed by a linear gradient (20 CV): 0%→100% ethyl acetate in cyclohexane). Clear colorless oil; ^{1}H NMR (600 MHz, 300 K, $CDCl_3$): δ = 8.16 (d, J = 10.6 Hz, 1H), 7.34 (t, J = 7.8 Hz, 2H), 7.06 (t, J = 7.4 Hz, 1H), 6.95 (d, J = 8.3 Hz, 2H), 6.23 (dd, J = 10.6, 4.7 Hz, 1H), 4.95 (d, J = 15.6 Hz, 1H), 4.85−4.82 (m, 2H), 4.62 (s, 1H), 4.62−4.56 (m, 2H), 1.67 (s, 3H), 1.49 ppm (s, 3H); ^{13}C NMR (150 MHz, 300 K, $CDCl_3$): δ = 173.4, 168.4, 165.5, 156.8, 129.8, 122.4, 114.8, 113.0, 67.0, 65.7, 64.8, 63.5, 56.4, 49.5, 20.3, 17.8 ppm; IR (film): ṽ = 3402, 3014, 2942, 1807, 1775, 1698, 1599, 1520, 1493, 1439, 1374, 1323, 1289, 1230, 1207, 1164, 1117, 1065, 1037 cm^{-1}; HRMS (ESI): *m/z* calculated for $C_{18}H_{19}O_7N_3SNa$ $[M+Na]^+$: 444.0836, found: 444.0835; $[\alpha]_D^{25}$ = +101.5 (c = 0.4, $CHCl_3$).

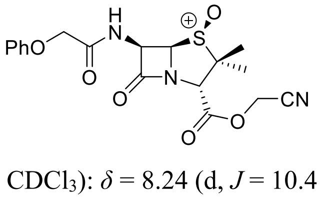

In addition to the penicillin V sulfone **20r**, the corresponding penicillin V sulfoxide **21r** (16 mg, 15%) was isolated in purified form as a single sulfoxide diastereomer. Note, the sulfoxide was tentatively assigned the (*S*)-configuration based on literature reports on peracid (including mCPBA)-mediated penicillin ester oxidations to sulfoxides.[11,20-21] Clear colorless oil; ^{1}H NMR (600 MHz, 300 K, $CDCl_3$): δ = 8.24 (d, J = 10.4 Hz, 1H), 7.33 (t, J = 7.7 Hz, 2H), 7.04 (t, J = 7.1 Hz, 1H), 6.96 (d, J = 8.5 Hz, 2H), 6.16 (dd, J = 10.5, 4.7 Hz, 1H), 5.10 (d, J = 4.7 Hz, 1H), 4.95 (d, J = 15.6 Hz, 1H), 4.82 (d, J = 15.6 Hz, 1H), 4.77 (s, 1H), 4.57 (s, 2H), 1.67 (s, 3H), 1.49 ppm (s, 3H); ^{13}C NMR (150 MHz, 300 K, $CDCl_3$): δ = 173.1, 168.3, 166.9,

157.0, 129.7, 122.3, 114.9, 113.3, 75.7, 67.1, 66.0, 55.6, 49.3, 26.9, 19.4, 18.6 ppm; IR (film): ṽ = 3377, 3016, 2938, 1794, 1771, 1690, 1599, 1519, 1493, 1458, 1438, 1373, 1292, 1240, 1202, 1154, 1043, 1020 cm^{-1}; HRMS (ESI): *m/z* calculated for $C_{18}H_{19}O_6N_3SNa$ $[M+Na]^+$: 428.0887, found: 428.0887; $[\alpha]_D^{25}$ = +161.9 (c = 0.1, $CHCl_3$).

Benzyl (2*S*,5*R*,6*R*)-6-amino-3,3-dimethyl-7-oxo-4-thia-1-azabicyclo[3.2.0]heptane-2-carboxylate (23). To a solution of commercially sourced (+)-6-aminopenicillanic acid ((+)-6-APA, 5.0 g, 23.0 mmol, 1.0 equiv.) in acetone (HPLC grade, 5.0 mL) was added triethylamine (3.5 mL, 25.3 mmol, 1.1 equiv.) under an ambient atmosphere at 0 °C. After stirring for 30 min at the same temperature, a solution of benzyl bromide (3.0 mL, 25.1 mmol, 1.09 equiv.) in acetone (HPLC grade, 9.0 mL) was added dropwise to the reaction mixture, which was then stirred for an additional 4 h at 0 °C. The reaction mixture was poured into diethyl ether (75 mL) and filtered. The filtrate was washed with saturated aqueous $NaHCO_3$ solution and water; a solution of 4-tolylsulfonic acid (4.6 g, 24.2 mmol, 1.05 equiv.) in acetone (HPLC grade, 25 mL) was then added to the organic phase. The resultant white precipitate was collected by filtration, dried in vacuum, and suspended in ethyl acetate. The suspension was washed twice with a 5%$_{w/v}$ aqueous $NaHCO_3$ solution. The organic phase was dried over anhydrous Na_2SO_4, filtered, and evaporated to afford analytically pure (+)-6-aminopenicillanic acid benzyl ester **23** (2.3 g, 33%). White solid, m.p.: 61−63 °C; ^{1}H NMR (600 MHz, 300 K, $CDCl_3$): δ = 7.41−7.36 (m, 5H), 5.53 (d, J = 4.3 Hz, 1H), 5.23−5.19 (m, 2H), 4.58 (d, J = 4.3 Hz, 1H), 4.45 (s, 1H), 1.64 (s, 3H), 1.45 ppm (s, 3H); ^{13}C NMR (150 MHz, 300 K, $CDCl_3$): δ = 177.7, 168.0, 134.8, 128.7 (3C), 128.6(6) (2C), 70.0, 69.9, 67.4, 64.0, 62.8, 31.7, 27.1 ppm; IR (film): ṽ = 3393, 3328, 3034, 2967, 2934, 1773, 1742, 1610, 1498, 1456, 1372, 1351, 1294, 1267, 1202, 1181, 1155, 1130, 1081, 1026 cm^{-1}; HRMS (ESI): *m/z* calculated for $C_{15}H_{18}O_3N_2SNa$ $[M+Na]^+$: 329.0930, found: 329.0930; $[\alpha]_D^{25}$ = +174.0 (c = 0.5, $CHCl_3$).

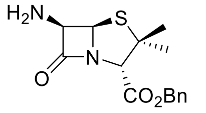

Benzyl (2*S*,5*R*,6*R*)-6-(((benzyloxy)carbonyl)amino)-3,3-dimethyl-7-oxo-4-thia-1-azabicyclo[3.2.0]heptane-2-carboxylate 4,4-dioxide (25a). To a solution of (+)-6-APA benzyl ester **23** (153 mg, 0.5 mmol, 1.0 equiv.) in anhydrous dichloromethane (5.0 mL) were sequentially added pyridine (81 µL, 1.0 mmol, 2.0 equiv.) and benzyl chloroformate (93 µL, 0.65 mmol, 1.3 equiv.) at 0 °C under an atmosphere of nitrogen gas. The reaction mixture was stirred at the same for 1 h at 0 °C and for 3 h at room temperature before being and washed with saturated aqueous $NaHCO_3$ solution. The organic phase was dried over anhydrous Na_2SO_4, filtered, evaporated, and purified by column chromatography (10 g KPSil cartridge; 36 mL/min; initially, 100% cyclohexane (3 CV), followed by a linear gradient (15 CV): 0%→100% ethyl acetate in cyclohexane) to afford the corresponding Cbz-protected (+)-6-aminopenicillanic acid benzyl ester (105 mg) together with some impurities. The mixture was used without further purification in the following reaction. According to General Procedure B, penicillin sulfone **25a** (42 mg, 40%) was obtained from Cbz-protected (+)-6-aminopenicillanic acid benzyl ester (103 mg, 0.22 mmol), following column chromatography (10 g KPSil cartridge; 36 mL/min; initially, 100% cyclohexane (3 CV), followed by a linear gradient (25 CV): 0%→100% ethyl acetate in cyclohexane). Clear colorless oil; ^{1}H NMR (600 MHz, 300 K, $CDCl_3$): δ = 7.43−7.34 (m, 10H), 6.21 (d, J = 10.7 Hz, 1H), 5.89 (dd, J = 10.8, 4.3 Hz, 1H), 5.30 (d, J = 11.9 Hz, 1H), 5.21−5.13 (m, 3H), 4.78 (d, J = 4.5 Hz, 1H), 4.52 (s, 1H), 1.56 (s, 3H), 1.27 ppm (s, 3H); ^{13}C NMR (150 MHz, 300 K, $CDCl_3$): δ = 174.1, 166.5, 155.1, 135.4, 134.2, 129.1, 128.9, 128.8(7), 128.6, 128.5, 128.3, 68.3, 68.0, 66.0, 64.8, 63.8, 59.6, 20.0, 17.8 ppm; IR (film): ṽ = 3370, 3034, 2980, 1806, 1754, 1726, 1517, 1456, 1379, 1321, 1284, 1245, 1214, 1172, 1117, 1051 cm^{-1}; HRMS (ESI): *m/z* calculated for $C_{23}H_{24}O_7N_2SNa$ $[M+Na]^+$: 495.1196, found: 495.1195; $[\alpha]_D^{25}$ = +95.4 (c = 0.2, $CHCl_3$).

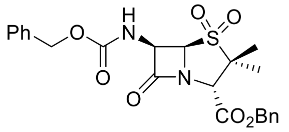

Benzyl (2*S*,5*R*,6*R*)-3,3-dimethyl-7-oxo-6-(3-phenylpropanamido)-4-thia-1-azabicyclo[3.2.0]heptane-2-carboxylate (24b). According to General Procedure C, penicillin benzyl ester **24b** (122 mg, 56%) was obtained

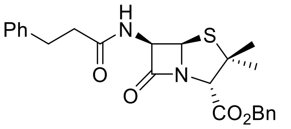

from (+)-6-APA benzyl ester **23** (153 mg, 0.5 mmol) and commercially-sourced hydrocinnamic acid, following column chromatography (10 g KPSil cartridge; 36 mL/min; initially, 100% cyclohexane (3 CV), followed by a linear gradient (20 CV): 0%→100% ethyl acetate in cyclohexane). Clear colorless oil; ^{1}H NMR (600 MHz, 300 K, $CDCl_3$): δ = 7.41−7.38 (m, 5H), 7.32−7.29 (m, 2H), 7.23−7.21 (m, 3H), 6.00 (d, J = 8.9 Hz, 1H), 5.73 (dd, J = 9.1, 4.2 Hz, 1H), 5.52 (d, J = 4.2 Hz, 1H), 5.23−5.19 (m, 2H), 4.45 (s, 1H), 2.99 (t, J = 7.8 Hz, 2H), 2.63−2.54 (m, 2H), 1.60 (s, 3H), 1.42 ppm (s, 3H); ^{13}C NMR (150 MHz, 300 K, $CDCl_3$): δ = 173.9, 171.4, 167.5, 140.3, 134.7, 128.8, 128.7, 128.6(9), 128.6, 128.3, 126.4, 70.5, 68.0, 67.5, 64.9, 58.5, 37.9, 31.7, 31.3, 27.0 ppm; IR (film): ṽ = 3306, 3062, 3032, 2980, 2931, 1783, 1747, 1661, 1529, 1498, 1455, 1373, 1352, 1298, 1266, 1204, 1184, 1156, 1131, 1077, 1028, 965 cm^{-1}; HRMS (ESI): *m/z* calculated for $C_{24}H_{26}O_4N_2SNa$ $[M+Na]^+$: 461.1505, found: 461.1498.

Benzyl (2*S*,5*R*,6*R*)-3,3-dimethyl-7-oxo-6-(3-phenylpropanamido)-4-thia-1-azabicyclo[3.2.0]heptane-2-carboxylate 4,4-dioxide (25b). According to General Procedure B, penicillin sulfone **25b** (36 mg, 28%) was

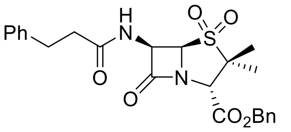

obtained from penicillin benzyl ester **24b** (120 mg, 0.27 mmol), following column chromatography (10 g KPSil cartridge; 36 mL/min; initially, 100% cyclohexane (3 CV), followed by a linear gradient (25 CV): 0%→100% ethyl acetate in cyclohexane). Clear colorless oil; ^{1}H NMR (600 MHz, 300 K, $CDCl_3$): δ = 7.42−7.38 (m, 5H), 7.31−7.29 (m, 2H), 7.23−7.20 (m, 3H), 6.98 (d, J = 10.2 Hz, 1H), 6.08 (dd, J = 10.2, 4.6 Hz, 1H), 5.31 (d, J = 12.0 Hz, 1H), 5.20 (d, J = 12.0 Hz, 1H), 4.99 (d, J = 4.6 Hz, 1H), 4.68 (s, 1H), 3.02−2.94 (m, 2H), 2.61−2.50 (m, 2H), 1.67 (s, 3H), 1.09 ppm (s, 3H); ^{13}C NMR (150 MHz, 300 K, $CDCl_3$): δ = 173.8, 171.7, 167.7, 140.3, 134.6, 129.0, 128.9, 128.8, 128.6, 128.3, 126.3, 75.4, 68.0, 66.3, 56.3, 37.9, 31.2, 19.4, 18.4 ppm; IR (film): ṽ = 3385, 3030, 2971, 1793, 1750, 1683, 1499, 1456, 1374, 1287, 1208, 1158, 1073, 1040 cm^{-1}; $[\alpha]_D^{25}$ = +172.4 (c = 0.1, $CHCl_3$).

Benzyl (2*S*,5*R*,6*R*)-6-(3-(2,6-dichlorophenyl)-5-methylisoxazole-4-carboxamido)-3,3-dimethyl-7-oxo-4-thia-1-azabicyclo[3.2.0]heptane-2-carboxylate (24c). To a solution of commercially-sourced dichloxacillin

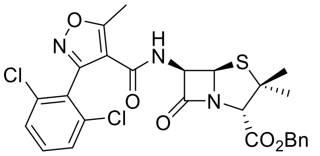

sodium salt (2.0 g, 3.9 mmol, 1.0 equiv.) in anhydrous DMF (10 mL) was added benzyl bromide (0.5 mL, 4.3 mmol, 1.1 equiv.) under an atmosphere of N_2 gas at ambient temperature. The reaction mixture was stirred overnight, then poured onto ice water. The mixture was extracted with three times with chloroform; the combined organic extracts were washed with saturated aqueous $NaHCO_3$ solution, water, and brine. The organic phase was dried over anhydrous Na_2SO_4, filtered, evaporated, and purified by column chromatography (25 g Sfär cartridge; initially, 100% cyclohexane (2 CV), followed by a linear gradient (12 CV): 0%→20% ethyl acetate in cyclohexane) to afford dichloxacillin benzyl ester **24c** (1.82 g, 82%). White amorphous solid; ^{1}H NMR (600 MHz, 300 K, $CDCl_3$): δ = 7.52−7.48 (m, 2H), 7.45−7.35 (m, 6H), 5.95 (d, J = 9.0 Hz, 1H), 5.75 (dd, J = 9.1, 4.3 Hz, 1H), 5.47 (d, J = 4.3 Hz, 1H), 5.19 (d, J = 12.7 Hz, 1H), 5.17 (d, J = 12.2 Hz, 1H), 4.38 (s, 1H), 2.84 (s, 3H), 1.42 (s, 3H), 1.36 ppm (s, 3H); ^{13}C NMR (150 MHz, 300 K, $CDCl_3$): δ = 176.0, 172.8, 167.3, 159.7, 155.8, 136.3, 136.2, 134.6, 132.2, 128.9, 128.8, 128.7, 128.6(7), 126.9, 110.6, 70.2, 67.6, 67.5, 64.9, 58.6, 32.6, 26.8, 13.6 ppm; IR (film): ṽ = 3403, 3076, 3035, 2968, 2931, 1786, 1744, 1673, 1601, 1561, 1506, 1432, 1393, 1297, 1201, 1156, 1129, 1088, 1026, 970 cm^{-1}; HRMS (ESI): *m/z* calculated for $C_{26}H_{23}O_5N_3Cl_2SNa$ $[M+Na]^+$: 582.0628, found: 582.0625; $[\alpha]_D^{25}$ = +66 (c = 1.1, $CHCl_3$).

Benzyl (2*S*,5*R*,6*R*)-6-(3-(2,6-dichlorophenyl)-5-methylisoxazole-4-carboxamido)-3,3-dimethyl-7-oxo-4-thia-1-azabicyclo[3.2.0]heptane-2-carboxylate 4,4-dioxide (25c). To a solution of a dichloxacillin benzyl ester

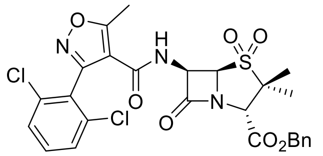

24c (1.7 g, 3.0 mmol, 1.0 equiv.) in anhydrous dichloromethane (2.0 mL) was added dropwise a solution of mCPBA (516 mg, 3.0 mmol, 1.0 equiv.) in anhydrous dichloromethane (2.0 mL) under an ambient atmosphere at 0 °C. The reaction mixture was stirred at 10 °C for 1 h, then sequentially washed with 10%$_{w/v}$ aqueous sodium metabisulfite solution, saturated aqueous $NaHCO_3$ solution, and brine. The organic phase was dried over anhydrous Na_2SO_4, filtered, evaporated, and purified by column chromatography (25 g Sfär cartridge; initially, 100% cyclohexane (2 CV), followed by a linear gradient (20 CV): 0%→50% ethyl acetate in cyclohexane) to afford dichloxacillin (*S*)-sulfoxide benzyl ester (1.25 g, 71%).

To a solution of dichloxacillin (*S*)-sulfoxide benzyl ester (100 mg, 0.17 mmol) in aqueous acetic acid (5.0 mL, 4:1$_{v/v}$, acetic acid:water) was added a solution of potassium permanganate (20 mg, 0.13 mmol, 0.7 equiv.) in water (2.0 mL) at 0 °C under an ambient atmosphere dropwise over 1 h. After completion of the addition, the reaction mixture was stirred for 90 min at 0 °C, then aqueous H_2O_2 (30%$_{w/v}$) was added; the mixture was extracted three times with dichloromethane. The combined organic extracts were sequentially washed with saturated aqueous $NaHCO_3$ solution and brine, dried over anhydrous Na_2SO_4, filtered, evaporated, and purified by column chromatography (5 g Sfär cartridge; initially, 100% cyclohexane (3 CV), followed by a linear gradient (10 CV): 0%→35% ethyl acetate in cyclohexane) to afford dichloxacillin sulfone benzyl ester **25c** (81 mg, 79%). White amorphous solid; ^{1}H NMR (600 MHz, 300 K, $CDCl_3$): δ = 7.48−7.45 (m, 2H), 7.42−7.36 (m, 6H), 6.68 (d, *J* = 10.4 Hz, 1H), 6.21 (dd, *J* = 10.4, 4.6 Hz, 1H), 5.28 (d, *J* = 12.0 Hz, 1H), 5.18 (d, *J* = 12.0 Hz, 1H), 4.68 (d, *J* = 4.7 Hz, 1H), 4.39 (s, 1H), 2.83 (s, 3H), 1.47 (s, 3H), 1.21 ppm (s, 3H); ^{13}C NMR (150 MHz, 300 K, $CDCl_3$): δ = 175.3, 173.5, 166.4, 160.2, 156.4, 135.8, 135.7, 134.2, 132.0, 129.1, 129.0, 128.9, 128.8(7), 128.6, 126.1, 110.7, 68.3, 65.6, 64.4, 63.7, 56.2, 19.9, 17.6, 13.4 ppm; IR (film): ṽ = 3397, 2981, 2889, 1808, 1756, 1678, 1600, 1561, 1506, 1456, 1433, 1395, 1381, 1323, 1287, 1199, 1166, 1118, 1085, 1069 cm^{-1}; HRMS (ESI): *m/z* calculated for $C_{26}H_{23}O_7N_3Cl_2SNa$ [M+Na]$^+$: 614.0526, found: 614.0524; $[\alpha]_D^{25}$ = +87 (c = 0.9, $CHCl_3$).

Benzyl (2*S*,5*R*,6*R*)-6-(2-benzamidoacetamido)-3,3-dimethyl-7-oxo-4-thia-1-azabicyclo[3.2.0]heptane-2-carboxylate (24d). According to General Procedure C, penicillin benzyl ester **24d** (184 mg, 81%) was obtained

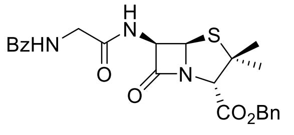

from (+)-6-APA benzyl ester **23** (153 mg, 0.5 mmol) and commercially-sourced hippuric acid, following column chromatography (10 g KP-Sil cartridge; 36 mL/min; initially, 100% cyclohexane (3 CV), followed by a linear gradient (20 CV): 0%→100% ethyl acetate in cyclohexane). White amorphous solid; ^{1}H NMR (600 MHz, 300 K, $CDCl_3$): δ = 7.82 (d, *J* = 7.9 Hz, 2H), 7.55 (t, *J* = 7.4 Hz, 1H), 7.49−7.45 (m, 2H), 7.41−7.38 (m, 5H), 6.88 (d, *J* = 9.0 Hz, 1H), 6.83 (t, *J* = 4.3 Hz, 1H), 5.72 (dd, *J* = 9.0, 4.1 Hz, 1H), 5.58 (d, *J* = 4.2 Hz, 1H), 5.21 (s, 2H), 4.50 (s, 1H), 4.26−4.18 (m, 2H), 1.61 (s, 3H), 1.43 ppm (s, 3H); ^{13}C NMR (150 MHz, 300 K, $CDCl_3$): δ = 173.0, 168.5, 167.9, 167.4, 134.6, 133.3, 132.1, 128.8, 128.7(4), 128.7, 128.6(9), 127.1, 70.4, 67.9, 67.6, 65.0, 58.7, 43.8, 32.2, 26.8 ppm; IR (film): ṽ = 3319, 3063, 3033, 2970, 2931, 1784, 1742, 1649, 1603, 1578, 1532, 1489, 1456, 1374, 1298, 1266, 1204, 1186, 1157, 1131, 1079, 1028, 994 cm^{-1}; HRMS (ESI): *m/z* calculated for $C_{24}H_{25}O_5N_3SNa$ [M+Na]$^+$: 490.1407, found: 490.1408; $[\alpha]_D^{25}$ = +156.6 (c = 1.0, $CHCl_3$).

Benzyl (2*S*,5*R*,6*R*)-6-(2-benzamidoacetamido)-3,3-dimethyl-7-oxo-4-thia-1-azabicyclo[3.2.0]heptane-2-carboxylate 4,4-dioxide (25d). According to General Procedure B, penicillin sulfone **25d** (46 mg, 31%) was obtained from penicillin ester **24d** (140 mg, 0.30 mmol), following column chromatography (10 g KP-Sil cartridge; 36 mL/min; initially, 100% cyclohexane (3 CV), followed by a linear gradient (20 CV): 0%→100% ethyl acetate in cyclohexane). Clear colorless oil; ^{1}H NMR (600 MHz, 300 K, $CDCl_3$): δ = 7.83 (d, J = 7.3 Hz, 2H), 7.54 (t, J = 7.4 Hz, 1H), 7.48−7.45 (m, 2H), 7.43−7.36 (m, 6H), 6.73 (t, J = 5.0 Hz, 1H), 6.15 (dd, J = 10.4, 4.6 Hz, 1H), 5.31 (d, J = 11.9 Hz, 1H), 5.20 (d, J = 11.9 Hz, 1H), 4.78 (d, J = 4.6 Hz, 1H), 4.54 (s, 1H), 4.27 (dd, J = 17.2, 5.4 Hz, 1H), 4.21 (dd, J = 17.2, 5.0 Hz, 1H), 1.56 (s, 3H), 1.27 ppm (s, 3H); ^{13}C NMR (150 MHz, 300 K, $CDCl_3$): δ = 173.3, 168.8, 167.8, 166.4, 134.2, 133.5, 131.9, 129.1, 128.9, 128.8(9), 128.6, 127.2, 68.4, 65.6, 64.8, 63.9, 56.9, 43.4, 20.0, 17.9 ppm; IR (film): ṽ = 3360, 3066, 3033, 2981, 1805, 1755, 1692, 1658, 1603, 1580, 1526, 1487, 1462, 1381, 1323, 1289, 1214, 1170, 1117, 1071, 995 cm^{-1}; HRMS (ESI): *m/z* calculated for $C_{24}H_{26}O_7N_3S$ [M+H]$^+$: 500.1486, found: 500.1484.

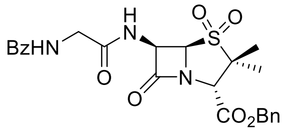

Benzyl (2*S*,5*R*,6*R*)-6-(2-(((benzyloxy)carbonyl)amino)acetamido)-3,3-dimethyl-7-oxo-4-thia-1-azabicyclo[3.2.0]heptane-2-carboxylate 4,4-dioxide (25e). According to General Procedure C, benzyl (2*S*,5*R*,6*R*)-6-(2-(((benzyloxy)carbonyl)amino)acetamido)-3,3-dimethyl-7-oxo-4-thia-1-azabicyclo[3.2.0]heptane-2-carboxylate along with some impurities (174 mg) was obtained from (+)-6-APA benzyl ester **23** (153 mg, 0.5 mmol) and commercially-sourced *N*-benzyloxycarbonylglycine, following column chromatography (10 g Sfär cartridge; 40 mL/min; initially, 100% cyclohexane (3 CV), followed by a linear gradient (20 CV): 0%→100% ethyl acetate in cyclohexane). The mixture was used in the following reaction without further purification to afford, according to General Procedure B, penicillin sulfone **25e** (55 mg, 30%), following column chromatography (10 g Sfär cartridge; 40 mL/min; initially, 100% cyclohexane (3 CV), followed by a linear gradient (20 CV): 0%→100% ethyl acetate in cyclohexane). Clear colorless oil; ^{1}H NMR (600 MHz, 300 K, $CDCl_3$): δ = 7.43−7.32 (m, 11H), 6.12 (dd, J = 10.4, 4.5 Hz, 1H), 5.32−5.30 (m, 2H), 5.21−5.14 (m, 3H), 4.77 (d, J = 4.4 Hz, 1H), 4.54 (s, 1H), 4.00−3.92 (m, 2H), 1.57 (s, 3H), 1.27 ppm (s, 3H); ^{13}C NMR (150 MHz, 300 K, $CDCl_3$): δ = 173.5, 168.9, 166.4, 156.4, 136.0, 134.2, 129.1, 128.9 (2C), 128.8(9) (2C), 128.5 (2C), 128.2 (3C), 68.4, 67.5, 65.7, 64.8, 63.9, 56.8, 44.5, 20.0, 17.9 ppm; IR (film): ṽ = 3399, 3065, 3035, 2979, 1805, 1755, 1725, 1697, 1518, 1456, 1377, 1322, 1215, 1171, 1117, 1052 cm^{-1}; HRMS (ESI): *m/z* calculated for $C_{25}H_{28}O_8N_3S$ [M+H]$^+$: 530.1592, found: 530.1588; $[\alpha]_D^{25}$ = +91.8 (c = 0.2, $CHCl_3$).

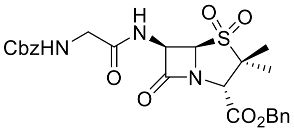

Benzyl (2*S*,5*R*,6*R*)-6-((*S*)-2-(((benzyloxy)carbonyl)amino)-4-methylpentanamido)-3,3-dimethyl-7-oxo-4-thia-1-azabicyclo[3.2.0]heptane-2-carboxylate (24f). According to General Procedure C, penicillin benzyl ester **24f** (124 mg, 44%) was obtained from (+)-6-APA benzyl ester **23** (153 mg, 0.5 mmol) and commercially-sourced Cbz-Leu-OH, following column chromatography (10 g Sfär cartridge; 40 mL/min; initially, 100% cyclohexane (3 CV), followed by a linear gradient (20 CV): 0%→100% ethyl acetate in cyclohexane). Clear colorless oil; ^{1}H NMR (600 MHz, 300 K, $CDCl_3$): δ = 7.42−7.33 (m, 11H), 6.79 (brd, J = 7.3 Hz, 1H), 5.65 (dd, J = 8.1, 3.1 Hz, 1H), 5.55 (d, J = 3.6 Hz, 1H), 5.23−5.19 (m, 2H), 5.14 (s, 2H), 5.06 (brd, J = 4.8 Hz, 1H), 4.49 (s, 1H), 4.23 (brs, 1H), 1.72−1.68 (m, 2H), 1.62 (s, 3H), 1.54−1.53 (m, 1H), 1.42 (s, 3H), 0.96−0.95 ppm (m, 6H); ^{13}C NMR (150 MHz, 300 K, $CDCl_3$): δ = 173.2, 171.6, 167.5, 156.2, 136.0, 134.7, 128.8, 128.7, 128.6(9), 128.6, 128.3, 128.1, 70.5, 67.9, 67.5, 67.3, 64.8, 58.7, 53.3, 40.7, 31.8, 26.9, 24.6, 22.9, 21.9 ppm; IR (film): ṽ = 3290, 3066, 3034, 2959, 2871, 1784, 1748, 1710, 1673, 1587, 1538, 1456, 1389, 1372, 1290, 1264, 1233, 1204, 1155, 1130, 1043, 1028 cm^{-1}.

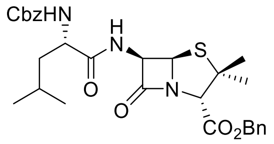

Benzyl (2*S*,5*R*,6*R*)-6-((*S*)-2-(((benzyloxy)carbonyl)amino)-4-methylpentanamido)-3,3-dimethyl-7-oxo-4-thia-1-azabicyclo[3.2.0]heptane-2-carboxylate 4,4-dioxide (25f). According to General Procedure B, penicillin

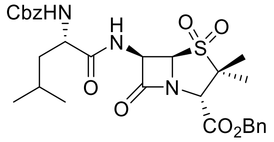

sulfone **25f** (58 mg, 45%) was obtained from penicillin ester **24f** (120 mg, 0.22 mmol), following column chromatography (10 g Sfär cartridge; 40 mL/min; initially, 100% cyclohexane (3 CV), followed by a linear gradient (20 CV): 0%→100% ethyl acetate in cyclohexane). Clear colorless oil; ^{1}H NMR (600 MHz, 300 K, $CDCl_3$): δ = 7.45−7.31 (m, 11H), 6.08 (dd, J = 9.9, 4.1 Hz, 1H), 5.31 (d, J = 11.9 Hz, 1H), 5.20 (d, J = 11.9 Hz, 1H), 5.16−5.12 (m, 2H), 5.06 (brd, J = 6.0 Hz, 1H), 4.76 (brd, J = 3.8 Hz, 1H), 4.54 (s, 1H), 4.28 (brs, 1H), 1.72−1.65 (m, 2H), 1.56 (s, 3H), 1.54−1.51 (m, 1H), 1.27 (s, 3H), 0.96−0.95 ppm (m, 6H); ^{13}C NMR (150 MHz, 300 K, $CDCl_3$): δ = 173.4, 172.2, 166.5, 156.1, 136.0, 134.2, 129.1, 128.9, 128.8(6), 128.5, 128.2, 68.3, 67.4, 65.6, 64.7, 63.8, 57.0, 53.4, 40.8, 24.6, 22.9, 21.8, 20.0, 17.8 ppm; IR (film): ṽ = 3368, 3316, 3034, 2958, 2871, 1805, 1755, 1717, 1686, 1516, 1456, 1386, 1323, 1285, 1257, 1214, 1170, 1117, 1048 cm^{-1}; HRMS (ESI): *m/z* calculated for $C_{29}H_{36}O_8N_3S$ [M+H]$^+$: 586.2218, found: 586.2213; $[\alpha]_D^{25}$ = +70.1 (c = 0.2, $CHCl_3$).

Benzyl (2*S*,5*R*,6*R*)-6-((*R*)-2-(4-ethyl-2,3-dioxopiperazine-1-carboxamido)-2-phenylacetamido)-3,3-dimethyl-7-oxo-4-thia-1-azabicyclo[3.2.0]heptane-2-carboxylate (24g). To a solution of commercially-sourced

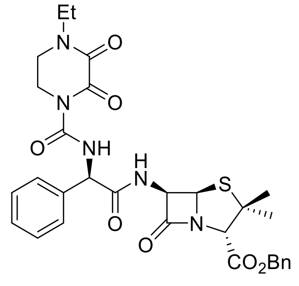

piperacillin sodium salt (3.0 g, 5.6 mmol, 1.0 equiv.) in anhydrous DMF (11 mL) was added benzyl bromide (0.71 mL, 6.1 mmol, 1.1 equiv.) under an atmosphere of N_2 gas at ambient temperature. The reaction mixture was stirred overnight, then poured onto ice water. The mixture was extracted with three times with chloroform and the combined organic extracts were washed with saturated aqueous $NaHCO_3$ solution, water, and brine. The organic extracts were dried over anhydrous Na_2SO_4, filtered, evaporated, and purified by column chromatography (50 g Sfär cartridge; initially, 100% cyclohexane (2 CV), followed by a linear gradient (15 CV): 0%→90% ethyl acetate in cyclohexane) to afford piperacillin benzyl ester **24g** (2.58 g, 76%). White amorphous solid; ^{1}H NMR (600 MHz, 300 K, $CDCl_3$): δ = 9.96 (d, J = 6.8 Hz, 1H), 7.42−7.32 (m, 10H), 6.82 (d, J = 9.0 Hz, 1H), 5.69 (dd, J = 9.0, 4.1 Hz, 1H), 5.50 (d, J = 6.8 Hz, 1H), 5.45 (d, J = 4.1 Hz, 1H), 5.18 (s, 2H), 4.38 (s, 1H), 4.18 (ddd, J = 13.7, 6.4, 3.6 Hz, 1H), 3.93−3.89 (m, 1H), 3.71−3.67 (m, 1H), 3.56 (q, J = 7.2 Hz, 2H), 3.46 (ddd, J = 13.3, 6.4, 3.7 Hz, 1H), 1.50 (s, 3H), 1.35 (s, 3H), 1.23 ppm (t, J = 7.2 Hz, 3H); ^{13}C NMR (150 MHz, 300 K, $CDCl_3$): δ = 173.4, 168.9, 167.3, 159.3, 155.8, 152.3, 136.4, 134.6, 129.2, 128.8, 128.7, 128.6(6), 128.6, 127.3, 70.4, 68.0, 67.5, 64.7, 58.7, 58.6, 43.5, 42.5, 40.7, 31.6, 26.7, 12.1 ppm; IR (film): ṽ = 3291, 3063, 3034, 2978, 2937, 1782, 1714, 1681, 1511, 1457, 1392, 1365, 1324, 1282, 1184 cm^{-1}; HRMS (ESI): *m/z* calculated for $C_{30}H_{33}O_7N_5SNa$ [M+Na]$^+$: 630.1993, found: 630.1990; $[\alpha]_D^{25}$ = +73 (c = 1.4, $CHCl_3$).

Benzyl (2*S*,4*S*,5*R*,6*R*)-6-((*R*)-2-(4-ethyl-2,3-dioxopiperazine-1-carboxamido)-2-phenylacetamido)-3,3-dimethyl-7-oxo-4-thia-1-azabicyclo[3.2.0]heptane-2-carboxylate 4-oxide (42). To a solution of a piperacillin

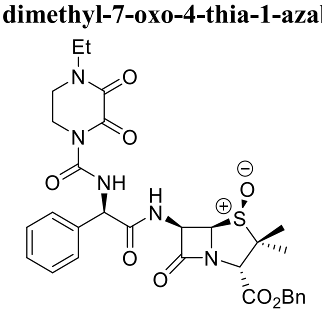

benzyl ester **24g** (1.5 g, 2.47 mmol, 1.0 equiv.) in anhydrous dichloromethane (2.0 mL) was added portion-wise mCPBA (424 mg, 2.47 mmol, 1.0 equiv.) under an ambient atmosphere at 0 °C. The reaction mixture was stirred at 10 °C for 1 h and was afterwards washed sequentially with 10%$_{w/v}$ aqueous sodium metabisulfite solution, saturated aqueous $NaHCO_3$ solution, and brine. The organic phase was dried over anhydrous Na_2SO_4, filtered, evaporated, and purified by column chromatography (10 g Sfär cartridge; 40 mL/min; initially, 100% cyclohexane (3 CV), followed by a linear gradient (20 CV): 0%→100% ethyl acetate in cyclohexane) to afford piperacillin (*S*)-sulfoxide benzyl ester **42** (1.2 g, 78%). Note, the sulfoxide was tentatively assigned the (*S*)-configuration based on literature reports on peracid (including mCPBA)-mediated penicillin ester oxidations to sulfoxides.[11,20-21] White

amorphous solid; ^{1}H NMR (600 MHz, 300 K, $CDCl_3$): δ = 9.94 (d, J = 5.7 Hz, 1H), 7.47 (d, J = 10.2 Hz, 1H), 7.42−7.33 (m, 10H), 5.97 (dd, J = 10.2, 4.4 Hz, 1H), 5.41 (d, J = 5.8 Hz, 1H), 5.28 (d, J = 12.0 Hz, 1H), 5.16 (d, J = 12.0 Hz, 1H), 4.94 (d, J = 4.4 Hz, 1H), 4.62 (s, 1H), 4.17−4.13 (m, 1H), 4.00−3.95 (m, 1H), 3.63−3.53 (m, 3H), 3.51−3.47 (m, 1H), 1.60 (s, 3H), 1.21 (t, J = 7.2 Hz, 3H), 1.02 ppm (s, 3H); ^{13}C NMR (150 MHz, 300 K, $CDCl_3$): δ = 172.9, 169.0, 167.5, 159.2, 155.7, 152.5, 135.9, 134.5, 129.2, 128.9, 128.8(3), 128.8, 128.7, 127.2, 76.5, 73.3, 67.9, 66.3, 59.4, 56.6, 43.6, 42.6, 40.6, 19.3, 18.3, 12.1 ppm; IR (film): ṽ = 3347, 3294, 3033, 2975, 2937, 1793, 1750, 1714, 1683, 1508, 1460, 1392, 1365, 1281, 1185, 1072, 1020 cm^{-1}; HRMS (ESI): *m/z* calculated for $C_{30}H_{33}O_8N_5SNa$ $[M+Na]^+$: 646.1942, found: 646.1938; $[\alpha]_D^{25}$ = +76 (c = 1.1, $CHCl_3$).

Benzyl (2*S*,5*R*,6*R*)-6-((*R*)-2-(4-ethyl-2,3-dioxopiperazine-1-carboxamido)-2-phenylacetamido)-3,3-dimethyl-7-oxo-4-thia-1-azabicyclo[3.2.0]heptane-2-carboxylate 4,4-dioxide (25g). To a solution of

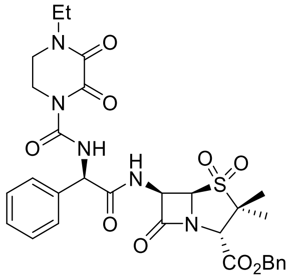

piperacillin (*S*)-sulfoxide benzyl ester **42** (200 mg, 0.32 mmol) in aqueous acetic acid (10.0 mL, 4:1$_{v/v}$, acetic acid:water) was added a solution of potassium permanganate (47 mg, 0.2 mmol, 0.7 equiv.) in water (3.3 mL) at 0 °C under an ambient atmosphere dropwise over 1 h. After completion of the addition, the reaction mixture was stirred for 90 min at 0 °C, then aqueous H_2O_2 (30%$_{w/v}$) was added; the mixture was extracted three times with dichloromethane and the combined organic extracts were sequentially washed with saturated aqueous $NaHCO_3$ solution and brine. The organic phase was dried over anhydrous Na_2SO_4, filtered, evaporated, and purified by column chromatography (10 g Sfär cartridge; 40 mL/min; initially, 100% cyclohexane (3 CV), followed by a linear gradient (15 CV): 0%→100% ethyl acetate in cyclohexane) to afford piperacillin sulfone benzyl ester **25g** (147 mg, 71%). White amorphous solid; ^{1}H NMR (600 MHz, 300 K, $CDCl_3$): δ = 9.86 (d, J = 5.5 Hz, 1H), 7.42−7.33 (m, 11H), 6.09 (dd, J = 10.6, 4.5 Hz, 1H), 5.40 (d, J = 5.6 Hz, 1H), 5.30 (d, J = 11.9 Hz, 1H), 5.18 (d, J = 12.0 Hz, 1H), 4.70 (d, J = 4.5 Hz, 1H), 4.48 (s, 1H), 4.20 (ddd, J = 13.7, 6.9, 3.7 Hz, 1H), 4.03−3.98 (m, 1H), 3.63−3.56 (m, 3H), 3.51 (ddd, J = 13.2, 6.9, 3.8 Hz, 1H), 1.50 (s, 3H), 1.23 (t, J = 7.3 Hz, 3H), 1.21 ppm (s, 3H); ^{13}C NMR (150 MHz, 300 K, $CDCl_3$): δ = 173.2, 169.2, 166.3, 159.3, 155.6, 152.6, 134.9, 134.2, 129.5, 129.2, 129.1, 128.9, 128.8(7), 127.2, 68.3, 65.7, 64.7, 63.8, 59.8, 57.3, 43.7, 42.7, 40.6, 20.0, 17.8, 12.2 ppm; IR (film): ṽ = 3395, 3294, 3033, 2976, 2939, 1804, 1755, 1714, 1684, 1510, 1459, 1393, 1365, 1324, 1283, 1185, 1071 cm^{-1}; HRMS (ESI): *m/z* calculated for $C_{30}H_{33}O_9N_5SNa$ $[M+Na]^+$: 662.1891, found: 662.1888; $[\alpha]_D^{25}$ = +38 (c = 1.4, $CHCl_3$).

Benzyl (2*S*,5*R*,6*R*)-3,3-dimethyl-7-oxo-6-(2-(perfluorophenoxy)acetamido)-4-thia-1-azabicyclo[3.2.0]heptane-2-carboxylate (24h). According to General Procedure C, penicillin benzyl ester **24h**

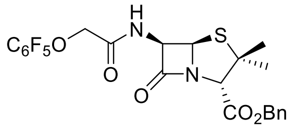

(209 mg, 79%) was obtained from (+)-6-APA benzyl ester **23** (153 mg, 0.5 mmol) and commercially-sourced (2,3,4,5,6-pentafluorophenoxy)acetic acid, following column chromatography (10 g Sfär cartridge; 40 mL/min; initially, 100% cyclohexane (3 CV), followed by a linear gradient (20 CV): 0%→100% ethyl acetate in cyclohexane). Clear pale yellow oil; ^{1}H NMR (600 MHz, 300 K, $CDCl_3$): δ = 7.42−7.37 (m, 6H), 5.78 (dd, J = 9.3, 4.3 Hz, 1H), 5.63 (d, J = 4.3 Hz, 1H), 5.23 (s, 2H), 4.74 (d, J = 15.0 Hz, 1H), 4.69 (d, J = 15.0 Hz, 1H), 4.56 (s, 1H), 1.67 (s, 3H), 1.47 ppm (s, 3H); ^{19}F NMR (565 MHz, 300 K, $CDCl_3$): δ = −156.1 (d, J = 19.0 Hz, 2F), −160.8 (t, J = 22.2 Hz, 1F), −161.7 ppm (m, 2F); ^{13}C NMR (150 MHz, 300 K, $CDCl_3$): δ = 172.7, 167.4, 166.1, 141.2 (m, 2C), 138.1 (m, 3C), 134.6, 132.3 (m), 128.8, 128.7(5) (2C), 128.7 (2C), 72.9, 70.4, 67.8, 67.6, 65.1, 58.2, 32.4, 26.8 ppm; IR (film): ṽ = 3351, 3067, 3036, 2972, 1786, 1746, 1698, 1516, 1457, 1353, 1300, 1266, 1204, 1184, 1157, 1046, 992 cm^{-1}; HRMS (ESI): *m/z* calculated for $C_{23}H_{19}O_5N_2F_5SNa$ $[M+Na]^+$: 553.0827, found: 553.0823; $[\alpha]_D^{25}$ = +117.7 (c = 0.05, $CHCl_3$).

Benzyl (2*S*,5*R*,6*R*)-3,3-dimethyl-7-oxo-6-(2-(perfluorophenoxy)acetamido)-4-thia-1-azabicyclo[3.2.0]heptane-2-carboxylate 4,4-dioxide (25h). According to General Procedure B, penicillin

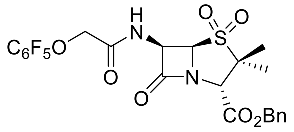

sulfone **25h** (123 mg, 56%) was obtained from penicillin benzyl ester **24h** (205 mg, 0.39 mmol), following column chromatography (10 g Sfär cartridge; 40 mL/min; initially, 100% cyclohexane (3 CV), followed by a linear gradient (20 CV): 0%→100% ethyl acetate in cyclohexane). White amorphous solid; ^{1}H NMR (600 MHz, 300 K, $CDCl_3$): δ = 8.12 (d, J = 10.5 Hz, 1H), 7.44−7.38 (m, 5H), 6.18 (dd, J = 10.5, 4.6 Hz, 1H), 5.32 (d, J = 11.9 Hz, 1H), 5.21 (d, J = 11.9 Hz, 1H), 4.82 (d, J = 4.6 Hz, 1H), 4.73 (d, J = 15.2 Hz, 1H), 4.70 (d, J = 15.2 Hz, 1H), 4.58 (s, 1H), 1.59 (s, 3H), 1.29 ppm (s, 3H); ^{19}F NMR (565 MHz, 300 K, $CDCl_3$): δ = −155.8 (d, J = 18.4 Hz, 2F), −160.9 (t, J = 21.4 Hz, 1F), −161.8 ppm (m, 2F); ^{13}C NMR (150 MHz, 300 K, $CDCl_3$): δ = 173.1, 166.8, 166.4, 141.2 (m, 2C), 138.1 (m, 3C), 134.2, 132.2 (m), 129.2, 128.9 (2C), 128.9 (2C), 72.7, 68.4, 65.5, 64.8, 63.9, 56.2, 20.0, 17.9 ppm; IR (film): ṽ = 3404, 2980, 1808, 1757, 1702, 1517, 1473, 1324, 1287, 1213, 1167, 1117, 1047, 992 cm^{-1}; HRMS (ESI): *m/z* calculated for $C_{23}H_{20}O_7N_2F_5S$ $[M+H]^+$: 563.0906, found: 563.0906; $[\alpha]_D^{25}$ = +88.5 (c = 1.05, $CHCl_3$).

Benzyl (2*S*,5*R*,6*R*)-3,3-dimethyl-7-oxo-6-(*rac*-2-phenoxypropanamido)-4-thia-1-azabicyclo[3.2.0]heptane-2-carboxylate 4,4-dioxide (25i). According to General Procedure B, penicillin sulfone **25i** (205 mg, 56%) was

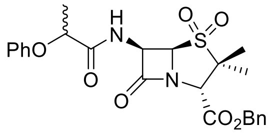

obtained as a diastereomeric mixture (1:1) from 4-benzyl (2*S*,5*R*,6*R*)-3,3-dimethyl-7-oxo-6-(*rac*-2-phenoxypropanamido)-4-thia-1-azabicyclo[3.2.0]heptane-2-carboxylate[15] (340 mg, 0.75 mmol), following column chromatography (10 g Sfär cartridge; 40 mL/min; initially, 100% cyclohexane (3 CV), followed by a linear gradient (20 CV): 0%→100% ethyl acetate in cyclohexane). Clear colorless oil; ^{1}H NMR (600 MHz, 300 K, $CDCl_3$): δ = 8.06−8.03 (m, 2H), 7.43−7.38 (m, 10H), 7.33−7.29 (m, 4H), 7.04−7.02 (m, 2H), 6.94−6.93 (m, 4H), 6.13−6.08 (m, 2H), 5.32−5.30 (m, 2H), 5.21−5.18 (m, 2H), 4.80−4.77 (m, 3H), 4.71 (d, J = 4.6 Hz, 1H), 4.53 (s, 1H), 4.52 (s, 1H), 1.62 (d, J = 6.8 Hz, 3H), 1.60 (d, J = 6.8 Hz, 3H), 1.57 (s, 3H), 1.53 (s, 3H), 1.29 (s, 3H), 1.23 ppm (s, 3H); ^{13}C NMR (150 MHz, 300 K, $CDCl_3$): δ = 173.6, 173.3, 172.3, 172.2, 166.5, 156.6, 156.5, 134.2(2), 134.2, 129.8, 129.1, 128.9(1), 128.9, 128.8(7), 122.3(4), 122.3, 115.8, 115.7, 74.8, 74.6, 68.3, 65.7, 65.6(5), 64.7, 64.6(5), 63.9, 56.7, 56.4, 20.0, 19.9(8), 18.6, 18.1, 17.9 ppm; IR (film): ṽ = 3407, 2982, 1806, 1756, 1698, 1599, 1509, 1491, 1457, 1375, 1323, 1288, 1224, 1171, 1117, 1073 cm^{-1}; HRMS (ESI): *m/z* calculated for $C_{24}H_{27}O_7N_2S$ $[M+H]^+$: 487.1533, found: 487.1533.

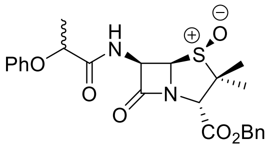

In addition to the penicillin sulfone **25i**, the corresponding penicillin sulfoxide **43** (72 mg, 20%) was isolated as a diastereomeric mixture (1:1) in purified form as a single sulfoxide diastereomer. Note, the sulfoxide was tentatively assigned the (*S*)-configuration based on literature reports on peracid (including mCPBA)-mediated penicillin ester oxidations to sulfoxides.[11,20-21] White amorphous solid; ^{1}H NMR (600 MHz, 300 K, $CDCl_3$): δ = 8.17 (d, J = 10.2 Hz, 1H), 8.08 (d, J = 10.6 Hz, 1H), 7.42−7.37 (m, 10H), 7.32−7.29 (m, 4H), 7.03−7.00 (m, 2H), 6.96−6.93 (m, 4H), 6.05−6.01 (m, 2H), 5.33−5.29 (m, 2H), 5.20−5.17 (m, 2H), 5.06 (d, J = 4.6 Hz, 1H), 4.92 (d, J = 4.6 Hz, 1H), 4.78−4.74 (m, 2H), 4.70 (s, 1H), 4.67 (s, 1H), 1.70 (s, 3H), 1.63 (s, 3H), 1.61 (d, J = 7.0 Hz, 3H), 1.60 (d, J = 7.2 Hz, 3H), 1.11 (s, 3H), 1.03 ppm (s, 3H); ^{13}C NMR (150 MHz, 300 K, $CDCl_3$): δ = 173.6, 173.3, 172.3, 172.2, 166.5, 156.6, 156.5, 134.2(2), 134.2, 129.8, 129.1, 128.9(1), 128.9, 128.8(8), 122.3(4), 122.3, 115.8, 115.7, 74.8, 74.6, 68.3, 65.7, 65.6(5), 64.7, 64.6(5), 63.9, 56.7, 56.4, 20.0, 19.9(8), 18.6, 18.1, 17.9 ppm; IR (film): ṽ = 3369, 2982, 1794, 1751, 1691, 1599, 1589, 1512, 1492, 1457, 1372, 1290, 1226, 1206, 1131, 1075, 1038 cm^{-1}; HRMS (ESI): *m/z* calculated for $C_{24}H_{27}O_6N_2S$ $[M+H]^+$: 471.1584, found: 471.1582.

4-Fluorobenzyl (2*S*,5*R*,6*R*)-3,3-dimethyl-7-oxo-6-(*rac*-2-phenoxypropanamido)-4-thia-1-azabicyclo[3.2.0]heptane-2-carboxylate (24j). According to General Procedure A, penicillin ester **24j** (340 mg, 72%) was obtained as a diastereomeric mixture (1:1) from commercially-sourced 4-fluorobenzylbromide and potassium (2*S*,5*R*,6*R*)-3,3-dimethyl-7-oxo-6-(*rac*-2-phenoxypropanamido)-4-thia-1-azabicyclo[3.2.0]heptane-2-carboxylate[15] (403 mg, 1.0 mmol), following column chromatography (10 g KPSil cartridge; 36 mL/min; initially, 100% cyclohexane (3 CV), followed by a linear gradient (20 CV): 0%→100% ethyl acetate in cyclohexane). Clear colorless oil; diastereomeric mixture (1:1) at the C6 side chain stereogenic center: ^{1}H NMR (600 MHz, 300 K, $CDCl_3$): δ = 7.38−7.35 (m, 4H), 7.33−7.30 (m, 4H), 7.23−7.20 (m, 2H), 7.10−7.06 (m, 4H), 7.05−7.01 (m, 2H), 6.94−6.92 (m, 4H), 5.67 (dd, J = 9.3, 4.2 Hz, 1H), 5.63−5.60 (m, 2H), 5.50 (d, J = 4.2 Hz, 1H), 5.19−5.14 (m, 4H), 4.78−4.73 (m, 2H), 4.45 (s, 1H), 4.44 (s, 1H), 1.62 (d, J = 6.8 Hz, 3H), 1.60 (d, J = 6.8 Hz, 3H), 1.54 (s, 3H), 1.44 (s, 3H), 1.40 (s, 3H), 1.35 ppm (s, 3H); ^{19}F NMR (565 MHz, 300 K, $CDCl_3$): δ = −112.5 ppm (m); ^{13}C NMR (150 MHz, 300 K, $CDCl_3$): δ = 173.3, 172.6, 171.8, 171.7, 167.4, 167.3(5), 162.9 (d, J = 247.7 Hz), 156.7, 156.6, 130.8 (d, J = 8.8 Hz), 130.6 (d, J = 3.2 Hz), 129.8(3), 129.8, 122.4, 122.3, 115.7 (d, J = 21.4 Hz), 115.7, 115.6, 74.9, 74.8, 70.4, 70.2, 67.9, 67.8, 66.7, 64.7, 58.6, 58.1, 32.1, 31.5, 26.8, 26.6, 18.7, 18.5 ppm; IR (film): ṽ = 3368, 2981, 2936, 1786, 1745, 1692, 1601, 1512, 1492, 1457, 1373, 1296, 1226, 1203, 1182, 1156, 1130, 1091, 1030 cm^{-1}; HRMS (ESI): m/z calculated for $C_{24}H_{25}O_5N_2FSNa$ $[M+Na]^+$: 495.1360, found: 495.1359.

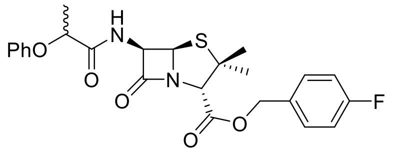

4-Fluorobenzyl (2*S*,5*R*,6*R*)-3,3-dimethyl-7-oxo-6-(*rac*-2-phenoxypropanamido)-4-thia-1-azabicyclo[3.2.0]heptane-2-carboxylate 4,4-dioxide (25j). According to General Procedure B, penicillin sulfone **25j** (223 mg, 61%) was obtained as a diastereomeric mixture (1:1) from penicillin ester **24j** (338 mg, 0.72 mmol), following column chromatography (10 g Sfär cartridge; 40 mL/min; initially, 100% cyclohexane (3 CV), followed by a linear gradient (20 CV): 0%→100% ethyl acetate in cyclohexane). White amorphous solid; ^{1}H NMR (600 MHz, 300 K, $CDCl_3$): δ = 8.06−8.03 (m, 2H), 7.39−7.37 (m, 4H), 7.33−7.29 (m, 4H), 7.12−7.09 (m, 4H), 7.04−7.02 (m, 2H), 6.94−6.92 (m, 4H), 6.13−6.08 (m, 2H), 5.28−5.26 (m, 2H), 5.18−5.15 (m, 2H), 4.80−4.76 (m, 3H), 4.71 (d, J = 4.6 Hz, 1H), 4.51 (s, 1H), 4.50 (s, 1H), 1.62 (d, J = 6.8 Hz, 3H), 1.60 (d, J = 6.8 Hz, 3H), 1.57 (s, 3H), 1.52 (s, 3H), 1.28 (s, 3H), 1.22 ppm (s, 3H); ^{19}F NMR (565 MHz, 300 K, $CDCl_3$): δ = −111.8 ppm (m); ^{13}C NMR (150 MHz, 300 K, $CDCl_3$): δ = 173.7, 173.3, 172.3, 172.2, 166.4, 163.1 (d, J = 249.1 Hz), 156.6, 156.5, 131.1 (d, J = 9.4 Hz), 130.2 (m), 129.8, 122.4, 122.3, 115.9 (d, J = 21.8 Hz), 115.8, 115.7, 74.8, 74.6, 67.5, 65.7, 65.6(5), 64.7, 64.6, 63.8, 56.7, 56.4, 20.1, 20.0, 18.6, 18.1, 17.9 ppm; IR (film): ṽ = 3407, 2982, 1806, 1756, 1697, 1601, 1513, 1492, 1461, 1375, 1323, 1288, 1226, 1172, 1158, 1117, 1073 cm^{-1}; HRMS (ESI): m/z calculated for $C_{24}H_{26}O_7N_2FS$ $[M+H]^+$: 505.1439, found: 505.1437.

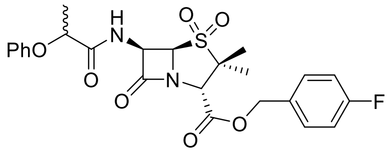

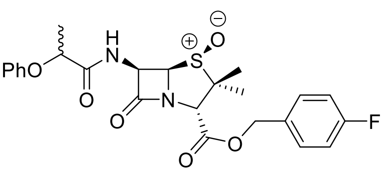

In addition to the penicillin sulfone **25j**, the corresponding penicillin sulfoxide **44** (76 mg, 22%) was isolated as a diastereomeric mixture (1:1) in purified form as a single sulfoxide diastereomer. Note, the sulfoxide was tentatively assigned the (*S*)-configuration based on literature reports on peracid (including mCPBA)-mediated penicillin ester oxidations to sulfoxides.[11,20-21] Clear colorless oil; ^{1}H NMR (600 MHz, 300 K, $CDCl_3$): δ = 8.17 (d, J = 10.3 Hz, 1H), 8.08 (d, J = 10.5 Hz, 1H), 7.40−7.37 (m, 4H), 7.32−7.28 (m, 4H), 7.11−7.07 (m, 4H), 7.03−7.00 (m, 2H), 6.96−6.93 (m, 4H), 6.05−6.01 (m, 2H), 5.28 (d, J = 12.0 Hz, 1H), 5.27 (d, J = 12.0 Hz, 1H), 5.16 (d, J = 12.0 Hz, 1H), 5.15 (d, J = 12.0 Hz, 1H), 5.06 (d, J = 4.6 Hz, 1H), 4.92 (d, J = 4.6 Hz, 1H), 4.78−4.74 (m, 2H), 4.68 (s, 1H), 4.65 (s, 1H), 1.69 (s, 3H), 1.62 (s, 3H), 1.61 (d, J = 7.0 Hz, 3H), 1.60 (d, J = 7.1 Hz, 3H), 1.10 (s, 3H), 1.02 ppm (s, 3H); ^{19}F NMR (565 MHz, 300 K, $CDCl_3$): δ = −112.1 ppm (m); ^{13}C NMR (150 MHz, 300 K, $CDCl_3$): δ = 173.2, 173.0,

172.2, 172.1, 167.7, 163.0 (d, J = 248.7 Hz), 156.8, 156.7, 131.0 (d, J = 7.8 Hz), 130.5 (m), 129.7, 129.6(9), 122.3, 122.0, 115.9, 115.8 (d, J = 21.9 Hz), 115.5, 76.6, 76.5, 75.3, 75.2(7), 74.8, 74.6, 67.2, 66.3, 66.2(5), 55.9, 55.6, 19.4, 18.7, 18.5, 18.4, 18.2 ppm; IR (film): ṽ = 3378, 2981, 1793, 1751, 1690, 1601, 1512, 1491, 1459, 1373, 1291, 1226, 1156, 1132, 1075, 1039 cm^{-1}; HRMS (ESI): *m/z* calculated for $C_{24}H_{26}O_6N_2FS$ $[M+H]^+$: 489.1490, found: 489.1490.

Benzyl (2*S*,5*R*,6*R*)-6-(2-(4-methoxyphenoxy)acetamido)-3,3-dimethyl-7-oxo-4-thia-1-azabicyclo[3.2.0]heptane-2-carboxylate (24k). According to General Procedure C, penicillin benzyl ester **24k** (158 mg, 67%) was obtained from (+)-6-APA benzyl ester **23** (153 mg, 0.5 mmol) and commercially-sourced 2-(4-methoxyphenoxy)acetic acid, following column chromatography (10 g Sfär cartridge; 40 mL/min; initially, 100% cyclohexane (3 CV), followed by a linear gradient (20 CV): 0%→100% ethyl acetate in cyclohexane). Clear pale yellow oil; ^{1}H NMR (600 MHz, 300 K, $CDCl_3$): δ = 7.39−7.34 (m, 6H), 6.89−6.86 (m, 4H), 5.75 (dd, J = 9.1, 4.1 Hz, 1H), 5.60 (d, J = 4.1 Hz, 1H), 5.24−5.20 (m, 2H), 4.55−4.48 (m, 3H), 3.79 (s, 3H), 1.60 (s, 3H), 1.44 ppm (s, 3H); ^{13}C NMR (150 MHz, 300 K, $CDCl_3$): δ = 173.1, 168.1, 167.4, 155.0, 151.2, 134.7, 128.8, 128.7, 128.6(9), 115.9, 114.9, 70.4, 68.1, 67.8, 67.6, 64.9, 58.1, 55.7, 31.9, 26.8 ppm; IR (film): ṽ = 3369, 2969, 1785, 1744, 1693, 1506, 1457, 1297, 1228, 1205, 1183, 1156, 1063, 1031 cm^{-1}; HRMS (ESI): *m/z* calculated for $C_{24}H_{27}O_6N_2S$ $[M+H]^+$: 471.1584, found: 471.1581; $[\alpha]_D^{25}$ = +117.3 (c = 0.1, $CHCl_3$).

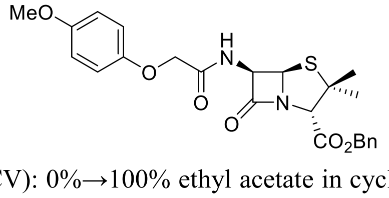

Benzyl (2*S*,5*R*,6*R*)-6-(2-(4-methoxyphenoxy)acetamido)-3,3-dimethyl-7-oxo-4-thia-1-azabicyclo[3.2.0]heptane-2-carboxylate 4,4-dioxide (25k). According to General Procedure B, penicillin sulfone **25k** (56 mg, 34%) was obtained from penicillin benzyl ester **24k** (155 mg, 0.33 mmol), following column chromatography (10 g Sfär cartridge; 40 mL/min; initially, 100% cyclohexane (3 CV), followed by a linear gradient (20 CV): 0%→100% ethyl acetate in cyclohexane). Clear pale yellow oil; ^{1}H NMR (600 MHz, 300 K, $CDCl_3$): δ = 8.16 (d, J = 10.6 Hz, 1H), 7.44−7.38 (m, 5H), 6.89−6.84 (m, 4H), 6.19 (dd, J = 10.7, 4.6 Hz, 1H), 5.32 (d, J = 11.9 Hz, 1H), 5.21 (d, J = 11.9 Hz, 1H), 4.79 (d, J = 4.6 Hz, 1H), 4.56 (s, 1H), 4.53 (d, J = 15.2 Hz, 1H), 4.50 (d, J = 15.2 Hz, 1H), 3.78 (s, 3H), 1.58 (s, 3H), 1.28 ppm (s, 3H); ^{13}C NMR (150 MHz, 300 K, $CDCl_3$): δ = 173.5, 168.6, 166.4, 154.9, 151.1, 134.2, 129.1, 128.9, 128.8(8), 116.0, 114.8, 68.3, 68.0, 65.7, 64.8, 63.9, 56.2, 55.7, 20.0, 17.9 ppm; IR (film): ṽ = 3402, 2971, 1805, 1755, 1698, 1505, 1458, 1441, 1322, 1288, 1214, 1170, 1116, 1067, 1033 cm^{-1}; HRMS (ESI): *m/z* calculated for $C_{24}H_{25}O_8N_2S$ $[M-H]^-$: 501.1337, found: 501.1329; $[\alpha]_D^{25}$ = +78.9 (c = 0.5, $CHCl_3$).

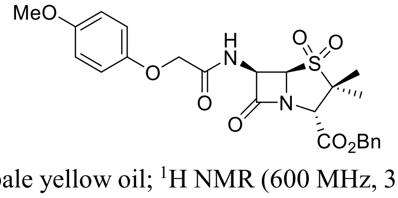

Benzyl (2*S*,5*R*,6*R*)-6-(2-(benzyloxy)acetamido)-3,3-dimethyl-7-oxo-4-thia-1-azabicyclo[3.2.0]heptane-2-carboxylate (24l). According to General Procedure C, penicillin benzyl ester **24l** (184 mg, 81%) was obtained from (+)-6-APA benzyl ester **23** (153 mg, 0.5 mmol) and commercially-sourced benzyloxyacetic acid, following column chromatography (10 g Sfär cartridge; 40 mL/min; initially, 100% cyclohexane (3 CV), followed by a linear gradient (20 CV): 0%→100% ethyl acetate in cyclohexane). Clear pale yellow oil; ^{1}H NMR (600 MHz, 300 K, $CDCl_3$): δ = 7.42−7.33 (m, 11H), 5.74 (dd, J = 9.5, 4.2 Hz, 1H), 5.58 (d, J = 4.2 Hz, 1H), 5.24−5.20 (m, 2H), 4.62 (s, 2H), 4.45 (s, 1H), 4.08 (d, J = 15.5 Hz, 1H), 4.03 (d, J = 15.5 Hz, 1H), 1.60 (s, 3H), 1.44 ppm (s, 3H); ^{13}C NMR (150 MHz, 300 K, $CDCl_3$): δ = 173.5, 169.1, 167.5, 136.6, 134.7, 128.8, 128.7, 128.6(9), 128.6, 128.2, 127.8, 73.6, 70.5, 69.1, 68.0, 67.5, 64.9, 58.0, 32.0, 26.9 ppm; IR (film): ṽ = 3379, 3064, 3033, 2970, 2931, 1788, 1744, 1693, 1513, 1455, 1373, 1297, 1265, 1204, 1184, 1156, 1101, 1027, 961 cm^{-1}; HRMS (ESI): *m/z* calculated for $C_{24}H_{26}O_5N_2SNa$ $[M+Na]^+$: 477.1455, found: 477.1453.

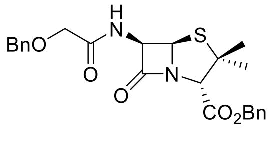

Benzyl (2*S*,5*R*,6*R*)-6-(2-(benzyloxy)acetamido)-3,3-dimethyl-7-oxo-4-thia-1-azabicyclo[3.2.0]heptane-2-carboxylate 4,4-dioxide (25l). According to General Procedure B, penicillin sulfone **25l** (60 mg, 31%) was obtained from penicillin benzyl ester **24l** (180 mg, 0.4 mmol), following column chromatography (10 g Sfär cartridge; 40 mL/min; initially, 100% cyclohexane (3 CV), followed by a linear gradient (20 CV): 0%→100% ethyl acetate in cyclohexane). Clear colorless oil; ^{1}H NMR (600 MHz, 300 K, $CDCl_3$): δ = 8.07 (d, J = 10.6 Hz, 1H), 7.44−7.31 (m, 10H), 6.15 (dd, J = 10.7, 4.5 Hz, 1H), 5.32 (d, J = 11.9 Hz, 1H), 5.21 (d, J = 11.9 Hz, 1H), 4.78 (d, J = 4.6 Hz, 1H), 4.63 (d, J = 12.1 Hz, 1H), 4.60 (d, J = 12.1 Hz, 1H), 4.56 (s, 1H), 4.05 (d, J = 15.7 Hz, 1H), 4.01 (d, J = 15.7 Hz, 1H), 1.59 (s, 3H), 1.29 ppm (s, 3H); ^{13}C NMR (150 MHz, 300 K, $CDCl_3$): δ = 173.7, 169.8, 166.4, 136.4, 134.2, 129.1, 128.9, 128.8(7), 128.6, 128.2, 127.9, 73.5, 68.9, 68.3, 65.8, 64.7, 63.9, 56.2, 20.0, 17.9 ppm; IR (film): ṽ = 3400, 3065, 3034, 2979, 1805, 1755, 1697, 1513, 1456, 1376, 1321, 1285, 1210, 1169, 1116, 1028 cm^{-1}; HRMS (ESI): *m/z* calculated for $C_{24}H_{27}O_7N_2S$ $[M+H]^+$: 487.1533, found: 487.1531; $[\alpha]_D^{25}$ = +106.5 (c = 0.2, $CHCl_3$).

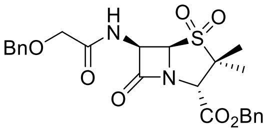

Benzyl (2*S*,5*R*,6*R*)-6-(2-(cyclohexyloxy)acetamido)-3,3-dimethyl-7-oxo-4-thia-1-azabicyclo[3.2.0]heptane-2-carboxylate (24m). According to General Procedure C, penicillin benzyl ester **24m** (143 mg, 64%) was obtained from (+)-6-APA benzyl ester **23** (153 mg, 0.5 mmol) and commercially-sourced cyclohexyloxyacetic acid, following column chromatography (10 g Sfär cartridge; 40 mL/min; initially, 100% cyclohexane (3 CV), followed by a linear gradient (20 CV): 0%→100% ethyl acetate in cyclohexane). Clear pale yellow oil; ^{1}H NMR (600 MHz, 300 K, $CDCl_3$): δ = 7.45−7.37 (m, 6H), 5.74 (dd, J = 9.6, 4.3 Hz, 1H), 5.59 (d, J = 4.3 Hz, 1H), 5.22 (s, 2H), 4.53 (s, 1H), 4.05 (d, J = 15.7 Hz, 1H), 4.00 (d, J = 15.7 Hz, 1H), 3.39−3.35 (m, 1H), 1.89−1.88 (m, 2H), 1.76−1.75 (m, 2H), 1.64 (s, 3H), 1.45 (s, 3H), 1.42−1.25 ppm (m, 6H); ^{13}C NMR (150 MHz, 300 K, $CDCl_3$): δ = 173.6, 170.1, 167.5, 134.7, 128.8, 128.7, 128.6(8), 78.6, 70.4, 68.1, 67.5, 67.2, 64.8, 58.1, 32.3, 31.9, 31.8, 26.8, 25.6, 23.6 ppm; IR (film): ṽ = 3383, 2933, 2857, 1786, 1745, 1694, 1510, 1455, 1373, 1296, 1265, 1203, 1183, 1155, 1105, 1026 cm^{-1}; HRMS (ESI): *m/z* calculated for $C_{23}H_{31}O_5N_2S$ $[M+H]^+$: 447.1948, found: 447.1942; $[\alpha]_D^{25}$ = +124.3 (c = 0.05, $CHCl_3$).

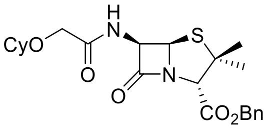

Benzyl (2*S*,5*R*,6*R*)-6-(2-(cyclohexyloxy)acetamido)-3,3-dimethyl-7-oxo-4-thia-1-azabicyclo[3.2.0]heptane-2-carboxylate 4,4-dioxide (25m). According to General Procedure B, penicillin sulfone **25m** (33 mg, 22%) was obtained from penicillin benzyl ester **24m** (140 mg, 0.32 mmol), following column chromatography (10 g Sfär cartridge; 40 mL/min; initially, 100% cyclohexane (3 CV), followed by a linear gradient (20 CV): 0%→100% ethyl acetate in cyclohexane). White amorphous solid; ^{1}H NMR (600 MHz, 300 K, $CDCl_3$): δ = 8.12 (d, J = 10.8 Hz, 1H), 7.44−7.38 (m, 5H), 6.14 (dd, J = 10.8, 4.6 Hz, 1H), 5.31 (d, J = 11.9 Hz, 1H), 5.20 (d, J = 11.9 Hz, 1H), 4.77 (d, J = 4.6 Hz, 1H), 4.55 (s, 1H), 4.06 (d, J = 15.9 Hz, 1H), 4.02 (d, J = 15.9 Hz, 1H), 3.36−3.32 (m, 1H), 1.90−1.88 (m, 2H), 1.75−1.73 (m, 2H), 1.58 (s, 3H), 1.55−1.50 (m, 1H), 1.41−1.34 (m, 2H), 1.30−1.24 ppm (m, 6H); ^{13}C NMR (150 MHz, 300 K, $CDCl_3$): δ = 174.0, 170.8, 166.5, 134.3, 129.1, 128.9, 128.8(8), 78.9, 68.3, 67.0, 65.8, 64.7, 63.8, 56.1, 31.8, 31.7, 25.5, 23.6, 20.1, 17.9 ppm; IR (film): ṽ = 3394, 2972, 2935, 2859, 1806, 1757, 1697, 1509, 1456, 1379, 1322, 1285, 1212, 1169, 1117, 1069, 954 cm^{-1}; HRMS (ESI): *m/z* calculated for $C_{23}H_{31}O_7N_2S$ $[M+H]^+$: 479.1846, found: 479.1848; $[\alpha]_D^{25}$ = +116.7 (c = 0.2, $CHCl_3$).

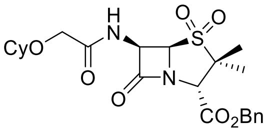

Benzyl (2*S*,5*R*,6*R*)-3,3-dimethyl-6-(2-(naphthalen-1-yloxy)acetamido)-7-oxo-4-thia-1-azabicyclo[3.2.0]heptane-2-carboxylate (24n). According to General Procedure C, penicillin benzyl ester **24n** (177 mg, 72%) was obtained from (+)-6-APA benzyl ester **23** (153 mg, 0.5 mmol) and commercially-sourced 2-(naphthalen-1-yloxy)acetic acid, following column chromatography (10 g Sfär cartridge; 40 mL/min; initially, 100% cyclohexane (3 CV), followed by a linear gradient (20 CV): 0%→100% ethyl acetate in cyclohexane). Clear orange oil; ^{1}H NMR (600 MHz, 300 K, $CDCl_3$): δ = 8.29 (d, J = 8.3 Hz, 1H), 7.86 (d, J = 8.0 Hz, 1H), 7.56−7.50 (m, 4H), 7.42−7.37 (m, 6H), 6.84 (d, J = 7.6 Hz, 1H), 5.83 (dd, J = 9.5, 4.3 Hz, 1H), 5.65 (d, J = 4.2 Hz, 1H), 5.22 (s, 2H), 4.81 (d, J = 15.0 Hz, 1H), 4.75 (d, J = 15.0 Hz, 1H), 4.54 (s, 1H), 1.53 (s, 3H), 1.43 ppm (s, 3H); ^{13}C NMR (150 MHz, 300 K, $CDCl_3$): δ = 172.8, 167.6, 167.3, 152.5, 134.7, 134.6, 128.8, 128.7, 128.6(9), 127.8, 126.7, 125.7, 125.6(7), 125.1, 122.1, 121.3, 105.7, 70.3, 68.1, 67.6, 67.3, 65.0, 58.3, 32.7, 26.6 ppm; IR (film): ṽ = 3394, 3055, 2972, 2934, 1785, 1743, 1696, 1597, 1580, 1508, 1462, 1398, 1298, 1268, 1242, 1203, 1181, 1157, 1107, 1076, 1025 cm^{-1}; HRMS (ESI): *m/z* calculated for $C_{27}H_{27}O_5N_2S$ $[M+H]^+$: 491.1635, found: 491.1632; $[\alpha]_D^{25}$ = +79.9 (c = 0.1, $CHCl_3$).

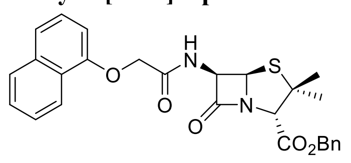

Benzyl (2*S*,5*R*,6*R*)-3,3-dimethyl-6-(2-(naphthalen-1-yloxy)acetamido)-7-oxo-4-thia-1-azabicyclo[3.2.0]heptane-2-carboxylate 4,4-dioxide (25n). According to General Procedure B, penicillin sulfone **25n** (20 mg, 11%) was obtained from penicillin benzyl ester **24n** (175 mg, 0.36 mmol), following column chromatography (10 g Sfär cartridge; 40 mL/min; initially, 100% cyclohexane (3 CV), followed by a linear gradient (20 CV): 0%→100% ethyl acetate in cyclohexane). Clear pale yellow oil; ^{1}H NMR (600 MHz, 300 K, $CDCl_3$): δ = 8.37 (d, J = 10.7 Hz, 1H), 8.33−8.31 (m, 1H), 7.83−7.81 (m, 1H), 7.55−7.51 (m, 3H), 7.45−7.37 (m, 6H), 6.80 (d, J = 7.6 Hz, 1H), 6.28 (dd, J = 10.7, 4.6 Hz, 1H), 5.33 (d, J = 11.9 Hz, 1H), 5.22 (d, J = 11.9 Hz, 1H), 4.85 (d, J = 4.6 Hz, 1H), 4.79 (d, J = 14.9 Hz, 1H), 4.73 (d, J = 14.9 Hz, 1H), 4.61 (s, 1H), 1.61 (s, 3H), 1.31 ppm (s, 3H); ^{13}C NMR (150 MHz, 300 K, $CDCl_3$): δ = 173.5, 168.1, 166.4, 152.6, 134.6, 134.2, 129.1, 128.9, 128.8(9), 127.5, 126.7, 126.0, 125.4, 125.1, 122.1, 121.7, 105.5, 68.4, 67.2, 65.7, 64.8, 63.9, 56.3, 20.1, 17.9 ppm; IR (film): ṽ = 3409, 3059, 2980, 1806, 1756, 1699, 1597, 1580, 1520, 1509, 1463, 1398, 1322, 1267, 1241, 1213, 1168, 1115, 1074, 1026 cm^{-1}; HRMS (ESI): *m/z* calculated for $C_{27}H_{25}O_7N_2S$ $[M-H]^-$: 521.1388, found: 521.1372; $[\alpha]_D^{25}$ = +58.3 (c = 0.1, $CHCl_3$).

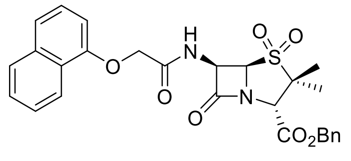

4-Benzyl (2*S*,5*R*)-6,6-dibromo-3,3-dimethyl-7-oxo-4-thia-1-azabicyclo[3.2.0]heptane-2-carboxylate 4,4-dioxide (28). To a solution of (2*S*,5*R*)-3,3-dimethyl-7-oxo-4-thia-1-azabicyclo[3.2.0]heptane-2-carboxylic acid 4,4-dioxide (6,6-dibromopenicillanic acid *S*,*S*-dioxide, **30**)[16] (150 mg, 0.39 mmol, 1.0 equiv.) in anhydrous DMF (4.0 mL) were sequentially added commercially-sourced trimethylamine (0.05 mL, 0.39 mmol, 1.0 equiv.) and benzyl bromide (0.05 mL, 0.42 mmol, 1.1 equiv.). The reaction mixture was stirred for 24 h at ambient temperature, then poured onto ice water. The mixture was extracted with ethyl acetate and the organic extract was sequentially washed with water and brine. The organic phase was dried over anhydrous Na_2SO_4, filtered, evaporated, and purified by column chromatography (5 g Sfär cartridge; initially, 100% cyclohexane (2 CV), followed by a linear gradient (25 CV): 0%→20% ethyl acetate in cyclohexane) to afford 6,6-dibromopenicillaic acid *S*,*S*-dioxide benzyl ester **28** (104 mg, 56%) along with minor amounts of impurities that were not separable. The analytical data of **28** are consistent with those reported.[24] White solid, m.p.: 143−144 °C; ^{1}H NMR (600 MHz, 300 K, $CDCl_3$): δ = 7.43−7.40 (m, 5H), 5.34 (d, J = 11.9 Hz, 1H), 5.22 (d, J = 11.9 Hz, 1H), 5.01 (s, 1H), 4.55 (s, 1H), 1.58 (s, 3H), 1.28 ppm (s, 3H); ^{13}C NMR (150 MHz, 300 K, $CDCl_3$): δ = 165.4, 164.2, 134.1, 129.2, 128.9, 128.8(7), 73.5, 68.5, 64.7, 63.0, 43.5, 19.5, 18.7 ppm; IR (film): ṽ = 3033, 2981, 2927, 1813, 1754, 1498, 1457, 1379, 1336, 1288, 1214, 1190, 1158, 1120, 1094 cm^{-1}; $[\alpha]_D^{25}$ = +157.0 (c = 0.01, $CHCl_3$).

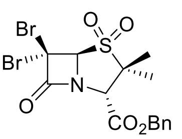

Benzyl (2*S*,5*R*,6*S*)-6-bromo-3,3-dimethyl-7-oxo-4-thia-1-azabicyclo[3.2.0]heptane-2-carboxylate 4,4-dioxide (29).

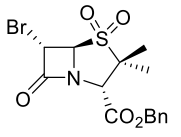

A solution of (2*S*,5*R*,6*S*)-6-bromo-3,3-dimethyl-7-oxo-4-thia-1-azabicyclo[3.2.0]heptane-2-carboxylic acid (6α-bromopenicillanic acid)[16] (1.1 g, 3.9 mmol, 1.0 equiv.) in aqueous dichloromethane (20 mL, 2:1$_{v/v}$, dichloromethane:water) was adjusted to pH = 7.2 by the addition of aqueous sodium hydroxide solution (1 M). The phases were separated and the aqueous phase was cooled to 0 °C, then a solution of potassium permanganate (1.2 g, 7.8 mmol l, 2.0 equiv.) in aqueous acetic acid (0.9 mL acetic acid in 30 mL water) was added dropwise over 30 min under an ambient atmosphere. The reaction mixture was stirred afterwards for 30 min at 0 °C, then it was acidified with aqueous HCl solution (1 M) to pH = 1. Aqueous H_2O_2 (30%$_{w/v}$) was added to the mixture which was then extracted three times with ethyl acetate. The combined organic extracts were dried over anhydrous Na_2SO_4, filtered and evaporated to afford the corresponding 6α-bromopenicillanic acid 4,4-dioxide which was used in the following reaction without further purification.
To a solution of the crude 6α-bromopenicillanic acid 4,4-dioxide (100 mg, 0.32 mmol, 1.0 equiv.) in anhydrous DMF (4.0 mL) were sequentially added commercially-sourced trimethylamine (0.04 mL, 0.32 mmol, 1.0 equiv.) and benzyl bromide (0.04 mL, 0.35 mmol, 1.1 equiv.). The reaction mixture was stirred for 24 h at ambient temperature, then poured onto ice water. The mixture was extracted with ethyl acetate; the organic extract was sequentially washed with water and brine, dried over anhydrous Na_2SO_4, filtered, evaporated, and purified by column chromatography (5 g Sfär cartridge; initially, 100% cyclohexane (2 CV), followed by a linear gradient (15 CV): 0%→10% ethyl acetate in cyclohexane) to afford 6α-bromopenicillaic acid *S*,*S*-dioxide benzyl ester **29** (73 mg, 57%). The analytical data of **29** are consistent with those reported.[25] Clear colorless oil; ^{1}H NMR (600 MHz, 300 K, $CDCl_3$): δ = 7.44−7.40 (m, 5H), 5.32 (d, J = 11.9 Hz, 1H), 5.22 (d, J = 11.9 Hz, 1H), 5.17 (d, J = 1.1 Hz, 1H), 4.69 (d, J = 1.0 Hz, 1H), 4.46 (s, 1H), 1.57 (s, 3H), 1.29 ppm (s, 3H); ^{13}C NMR (150 MHz, 300 K, $CDCl_3$): δ = 166.5, 165.9, 134.2, 129.1, 128.9, 128.8(7), 68.9, 68.4, 63.3, 63.2, 40.0, 19.9, 18.7 ppm; IR (film): ṽ = 2981, 2889, 1805, 1755, 1458, 1381, 1328, 1287, 1193, 1157, 1117, 1085 cm^{-1}; $[\alpha]_D^{25}$ = +136.0 (c = 1.0, $CHCl_3$).

4-Nitrobenzyl (2*S*,5*R*)-6,6-dibromo-3,3-dimethyl-7-oxo-4-thia-1-azabicyclo[3.2.0]heptane-2-carboxylate 4,4-dioxide (31).

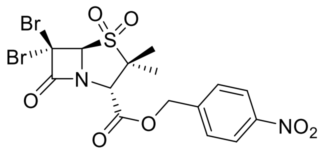

According to General Procedure B, penicillin V sulfone **31** (171 mg, 65%) was obtained from 4-nitrobenzyl (2*S*,5*R*)-6,6-dibromo-3,3-dimethyl-7-oxo-4-thia-1-azabicyclo[3.2.0]heptane-2-carboxylate (4-nitrobenzyl 6,6-dibromopenicillanate, **27**)[26] (247 mg, 0.5 mmol), following column chromatography (10 g Sfär cartridge; 40 mL/min; initially, 100% cyclohexane (3 CV), followed by a linear gradient (20 CV): 0%→80% ethyl acetate in cyclohexane). White solid, m.p.: >190 °C (decomposition); ^{1}H NMR (600 MHz, 300 K, $CDCl_3$): δ = 8.30−8.28 (m, 2H), 7.58 (d, J = 8.7 Hz, 2H), 5.39 (d, J = 12.9 Hz, 1H), 5.34 (d, J = 12.9 Hz, 1H), 5.03 (s, 1H), 4.60 (s, 1H), 1.63 (s, 3H), 1.37 ppm (s, 3H); ^{13}C NMR (150 MHz, 300 K, $CDCl_3$): δ = 165.3, 164.2, 148.2, 140.9, 129.1, 124.1, 73.6, 66.8, 64.7, 63.0, 43.5, 19.7, 18.8 ppm; IR (film): ṽ = 3114, 3083, 2982, 2943, 1813, 1758, 1608, 1522, 1463, 1378, 1343, 1288, 1214, 1188, 1158, 1121, 1095, 1014 cm^{-1}; $[\alpha]_D^{25}$ = +128.0 (c = 0.8, ethyl acetate).

4-Nitrobenzyl (2*S*,4*R*,5*R*)-6,6-dibromo-3,3-dimethyl-7-oxo-4-thia-1-azabicyclo[3.2.0]heptane-2-carboxylate 4-oxide (32).

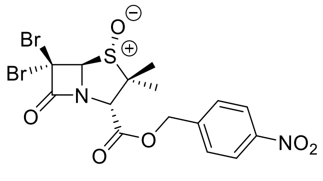

To a solution of 4-nitrobenzyl (2*S*,5*R*)-6,6-dibromo-3,3-dimethyl-7-oxo-4-thia-1-azabicyclo[3.2.0]heptane-2-carboxylate (4-nitrobenzyl 6,6-dibromopenicillanate, **27**) (250 mg, 0.51 mmol, 1.0 equiv.)[26] in anhydrous dichloromethane (5.0 mL) was added portion-wise mCPBA (88 mg, 0.51 mmol, 1.0 equiv.) under an ambient atmosphere at 10 °C. The reaction mixture was stirred at 10 °C for 1 h, then diluted with dichloromethane and washed three times with brine. The organic phase was dried over anhydrous Na_2SO_4, filtered, evaporated, and purified by column chromatography (5

g Sfär cartridge; 18 mL/min; initially, 100% cyclohexane (2 CV), followed by a linear gradient (40 CV): 0%→30% ethyl acetate in cyclohexane) to afford the corresponding (*R*)-sulfoxide benzyl ester **32** (120 mg, 46%). Note, the sulfoxide was tentatively assigned the (*R*)-configuration based on literature reports on peracid (including mCPBA)-mediated 6,6-halopenicillin ester oxidations to sulfoxides.[27-29] White solid, m.p.: 133−134 °C; ^{1}H NMR (600 MHz, 300 K, $CDCl_3$): δ = 8.28 (d, J = 8.6 Hz, 2H), 7.60 (d, J = 8.6 Hz, 2H), 5.36 (d, J = 12.9 Hz, 1H), 5.34 (s, 1H), 5.32 (d, J = 12.9 Hz, 1H), 4.69 (s, 1H), 1.58 (s, 3H), 1.40 ppm (s, 3H); ^{13}C NMR (150 MHz, 300 K, $CDCl_3$): δ = 165.7, 162.3, 148.2, 141.1, 129.1, 124.0, 94.1, 72.9, 67.4, 66.7, 47.8, 24.7, 17.1 ppm; IR (film): ṽ = 3115, 3082, 2971, 2935, 1803, 1752, 1607, 1521, 1457, 1373, 1347, 1302, 1263, 1212, 1185, 1129, 1108, 1065, 1035, 1016 cm^{-1}; $[\alpha]_D^{25}$ = +89.0 (c = 1.1, $CHCl_3$).

Benzyl (2*S*,5*R*)-3,3-dimethyl-7-oxo-4-thia-1-azabicyclo[3.2.0]heptane-2-carboxylate 4,4-dioxide (34). To a solution of commercially sourced sulbactam (630 mg, 2.7 mmol, 1.0 equiv.) and sodium carbonate (158 mg, 1.5 mmol, 0.55 equiv.) in anhydrous DMF (8.0 mL) was added dropwise benzyl bromide (530 mg, 3.1 mmol, 1.15 equiv.) at ambient temperature under an ambient atmosphere. The reaction mixture was stirred for 2 h, then poured onto ice water (30 mL). The mixture was extracted with diethyl ether. The organic phase was washed twice with saturated aqueous $NaHCO_3$ solution, dried over anhydrous Na_2SO_4, filtered, evaporated, and purified by column chromatography (25 g KP-Sil cartridge; 50 mL/min; initially, 100% cyclohexane (3 CV), followed by a linear gradient (15 CV): 0%→80% ethyl acetate in cyclohexane) to afford sulbactam benzyl ester **34** (714 mg, 82%). The analytical data of **34** are in agreement with those reported.[25] Clear colorless oil; ^{1}H NMR (600 MHz, 300 K, $CDCl_3$): δ = 7.43−7.38 (m, 5H), 5.31 (d, J = 12.0 Hz, 1H), 5.19 (d, J = 12.0 Hz, 1H), 4.61 (dd, J = 4.2, 1.8 Hz, 1H), 4.43 (s, 1H), 3.50 (dd, J = 16.2, 4.2 Hz, 1H), 3.45 (dd, J = 16.2, 1.3 Hz, 1H), 1.57 (s, 3H), 1.30 ppm (s, 3H); ^{13}C NMR (150 MHz, 300 K, $CDCl_3$): δ = 170.7, 166.8, 134.3, 129.0, 128.8(3), 128.8, 68.2, 63.2, 62.7, 61.0, 38.3, 20.1, 18.6 ppm; IR (film): ṽ = 3033, 2981, 2940, 1793, 1753, 1499, 1458, 1396, 1319, 1289, 1187, 1157, 1118, 1085, 999, 951 cm^{-1}; HRMS (ESI): *m/z* calculated for $C_{15}H_{17}O_5NSNa$ $[M+Na]^+$: 346.0720, found: 346.0721; $[\alpha]_D^{25}$ = +162.7 (c = 1.0, $CHCl_3$).

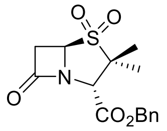

6. References

1. Zhang, J.-H.; Chung, T. D. Y.; Oldenburg, K. R., A simple statistical parameter for use in evaluation and validation of high throughput screening assays. *J. Biomol. Screen.* **1999,** *4* (2), 67-73.
2. Baldwin, J. E.; Lowe, C.; Schofield, C. J.; Lee, E., A γ-lactam analogue of penems possessing antibacterial activity. *Tetrahedron Lett.* **1986,** *27* (30), 3461-3464.
3. Baldwin, J. E.; Norris, W. J.; Freeman, R. T.; Bradley, M.; Adlington, R. M.; Long-Fox, S.; Schofield, C. J., γ-Lactam formation from tripeptides with isopenicillin N synthase. *J. Chem. Soc., Chem. Commun.* **1988,** (16), 1128-1130.
4. Bartolami, E.; Gilles, A.; Dumy, P.; Ulrich, S., Synthesis of α-PNA containing a functionalized triazine as nucleobase analogue. *Tetrahedron Lett.* **2015,** *56* (18), 2319-2323.
5. Dess, D. B.; Martin, J. C., Readily accessible 12-I-5 oxidant for the conversion of primary and secondary alcohols to aldehydes and ketones. *J. Org. Chem.* **1983,** *48* (22), 4155-4156.
6. Wang, Q.; Linhardt, R. J., Synthesis of a serine-based neuraminic acid C-glycoside. *J. Org. Chem.* **2003,** *68* (7), 2668-2672.
7. Maki, Y.; Sako, M.; Kurahashi, N.; Hirota, K., A simple and efficient synthesis of the γ-lactam analogue of β-lactam antibiotics. Ring-expansion of penicillins to homopenicillins. *J. Chem. Soc., Chem. Commun.* **1988,** (2), 110-111.
8. Malla, T. R.; Tumber, A.; John, T.; Brewitz, L.; Strain-Damerell, C.; Owen, C. D.; Lukacik, P.; Chan, H. T. H.; Maheswaran, P.; Salah, E.; Duarte, F.; Yang, H.; Rao, Z.; Walsh, M. A.; Schofield, C. J., Mass spectrometry reveals potential of β-lactams as SARS-CoV-2 Mpro inhibitors. *Chem. Commun.* **2021,** *57* (12), 1430-1433.
9. Zhao, Y.; Fang, C.; Zhang, Q.; Zhang, R.; Zhao, X.; Duan, Y.; Wang, H.; Zhu, Y.; Feng, L.; Zhao, J.; Shao, M.; Yang, X.; Zhang, L.; Peng, C.; Yang, K.; Ma, D.; Rao, Z.; Yang, H., Crystal structure of SARS-CoV-2 main protease in complex with protease inhibitor PF-07321332. *Protein Cell* **2021**, doi.org/10.1007/s13238-021-00883-2.
10. Redhead, M. A.; Owen, C. D.; Brewitz, L.; Collette, A. H.; Lukacik, P.; Strain-Damerell, C.; Robinson, S. W.; Collins, P. M.; Schäfer, P.; Swindells, M.; Radoux, C. J.; Hopkins, I. N.; Fearon, D.; Douangamath, A.; von Delft, F.; Malla, T. R.; Vangeel, L.; Vercruysse, T.; Thibaut, J.; Leyssen, P.; Nguyen, T.-T.; Hull, M.; Tumber, A.; Hallett, D. J.; Schofield, C. J.; Stuart, D. I.; Hopkins, A. L.; Walsh, M. A., Bispecific repurposed medicines targeting the viral and immunological arms of COVID-19. *Sci. Rep.* **2021,** *11* (1), 13208.
11. Baldwin, J. E.; Chakravarti, B.; Field, L. D.; Murphy, J. A.; Whitten, K. R.; Abraham, E. P.; Jayatilake, G., The synthesis of l-α-aminoadipyl-l-cysteinyl-d-3,4-didehydrovaline, a potent inhibitor of isopenicillin synthetase. *Tetrahedron* **1982,** *38* (18), 2773-2776.
12. Baldwin, J. E.; Abraham, E. P.; Adlington, R. M.; Crimmin, M. J.; Field, L. D.; Jayatilake, G. S.; White, R. L.; Usher, J. J., The synthesis and reactions of a monocyclic β-lactam tripeptide, 1-[(1R)-carboxy-2-methylpropyl]-(3R-[(5S-5-amino-5-carboxypentanamido]-(4R)-mercaptoazetidin-2-one, a putative intermediate in penicillin biosynthesis. *Tetrahedron* **1984,** *40* (10), 1907-1918.
13. Woydziak, Z. R., The use of lipohilic beta-lactam antibiotics and carboxylate esters for the treatment of bacterial infections within citrus and other plant species. **2020**, WO2020/154250A1.
14. Claes, P.; Vlietinck, A.; Roets, E.; Vanderhaeghe, H.; Toppet, S., Preparation and deoxygenation of 6-epi-penicillin S-oxides. *J. Chem. Soc., Perkin Trans. 1* **1973**, 932-937.
15. Aube, J.; Nathan, C.; Gold, B. S.; Hanson, P.; Liu, C.; Mitscher, L. A.; Pingle, M.; Schoenen, F. J., Cephalosporin derivatives and methods of use. **2014**, WO2014/071283A1.
16. Zhang, H. L.; Zhang, Y. M.; Liu, P.; Wang, Y. Q., Synthesis and crystal of methyl 6,6-dihydropenicillanate

S,S-dioxide. *Synth. React. Inorg. Met. Org. Chem.* **2012,** *42* (8), 1083-1086.

17. El-Faham, A.; Albericio, F., COMU: a third generation of uronium-type coupling reagents. *J. Pept. Sci.* **2010,** *16* (1), 6-9.
18. Ananda, G. D. S.; Stoodley, R. J., Studies related to penicillins. Part 27. A strategy for the conversion of 1,1-dioxides of penicillanates into 1,1-dioxides of 3-methylceph-3-em-4-carboxylates. *J. Chem. Soc., Perkin Trans. 1* **1988,** (12), 3359-3365.
19. Chow, A. W.; Hall, N. M.; Hoover, J. R. E., Penicillin sulfoxides and sulfones. *J. Org. Chem.* **1962,** *27* (4), 1381-1383.
20. Cooper, R. D. G.; DeMarco, P. V.; Cheng, J. C.; Jones, N. D., Structural studies on penicillin derivatives. I. Configuration of phenoxymethylpenicillin sulfoxide. *J. Am. Chem. Soc.* **1969,** *91* (6), 1408-1415.
21. Shin, W.; Kim, J.; Kim, J., Structure of penicillin V benzyl ester sulfoxide. *Acta Cryst. C* **1992,** *48* (8), 1449-1451.
22. Wolfe, S.; Ro, S.; Kim, C.-K.; Shi, Z., Synthesis and decarboxylation of Δ2-cephem-4,4-dicarboxylic acids. *Can. J. Chem.* **2001,** *79* (8), 1238-1258.
23. Gomis, P.; Izquierdo, M.; Jurado, A., Preparation and properties of some phenoxymethylpenicillin esters. *Bull. Soc. Chim. Fr.* **1968,** *1*, 420-424.
24. Marfat, A.; Mcleod, G. D., Beta-lactamase inhibitor prodrug. WO2004/018484.
25. Brennan, J.; Hussain, F. H. S., The synthesis of penicillanate esters from ultrasonically formed organozinc intermediates. *Synthesis* **1985,** *1985* (08), 749-751.
26. Miyashita, K.; Massova, I.; Taibi, P.; Mobashery, S., Design, synthesis, and evaluation of a potent mechanism-based inhibitor for the TEM β-lactamase with implications for the enzyme mechanism. *J. Am. Chem. Soc.* **1995,** *117* (45), 11055-11059.
27. Belinzoni, D. U.; Setti, E. L.; Mascaretti, O. A., Sterecontrolled access to (R)-sulfoxides and (S)-sulfoxides of penam derivatives and conformational analysis by H-1 and C-13 NMR-spectroscopy of (pivaloyloxy)methyl 6,6-dihalogenopenicillanates and 6-halogeno-penicillanates and their sulfoxides and sulfones. *J. Chem. Res. (S)* **1988**, 176-177.
28. Danelon, G. O.; Mata, E. G.; Mascaretti, O. A., Selective oxidation of penicillin derivatives to penicillin (1R) and (1S)-sulfoxides using dimethyldioxirane. *Tetrahedron Lett.* **1993,** *34* (49), 7877-7880.
29. Herak, J. J.; Vinković, M.; Lukić, I., Functional derivatives of 4-oxoazetidine-2-sulfinic acids in asymmetric synthesis of 2-azacepham sulfoxides and their transformation. *Tetrahedron* **1995,** *51* (17), 5083-5092.

7. ^{1}H and ^{13}C NMR spectra of novel penicillin sulfones prepared for this study

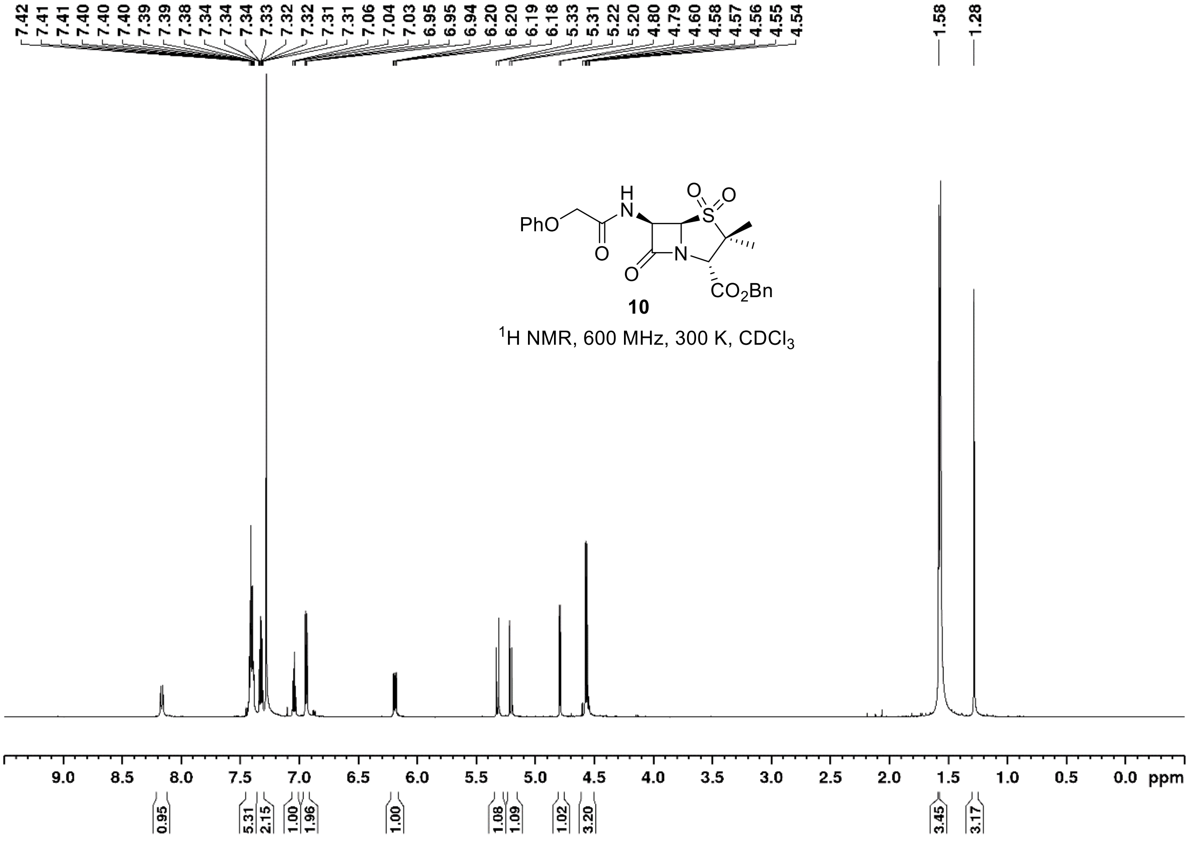

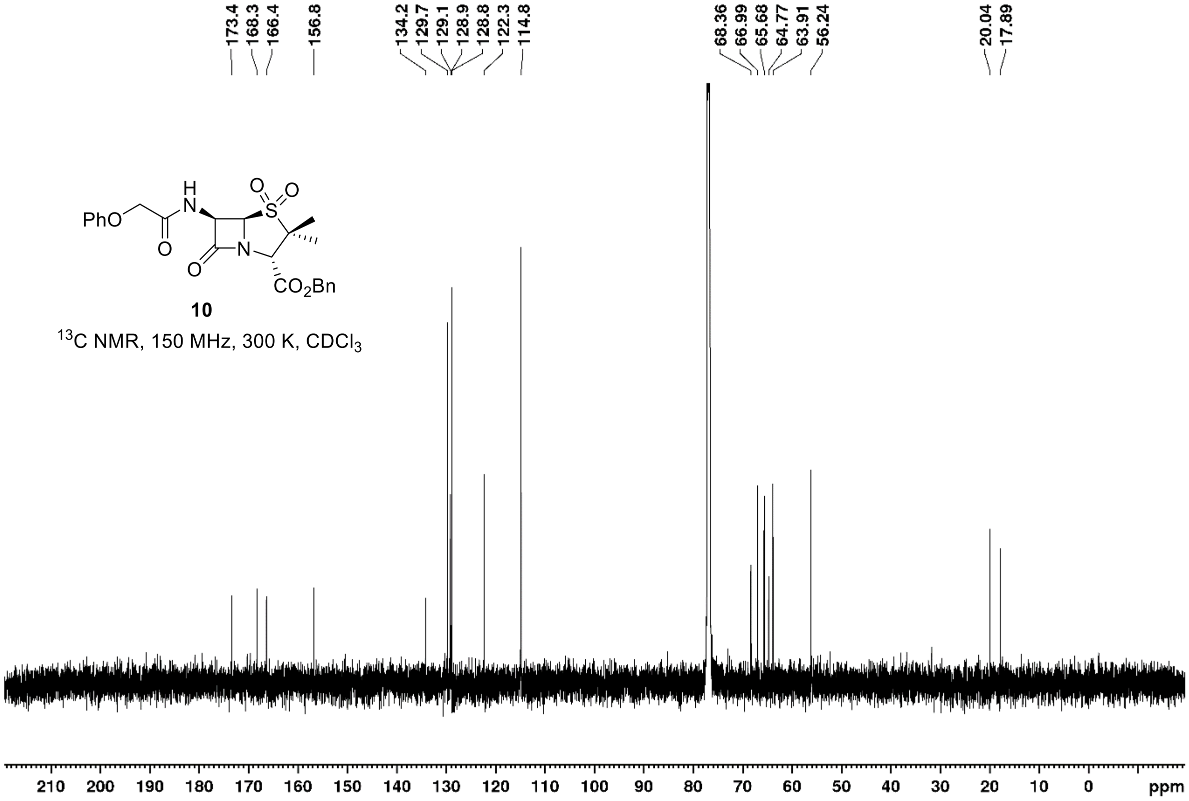

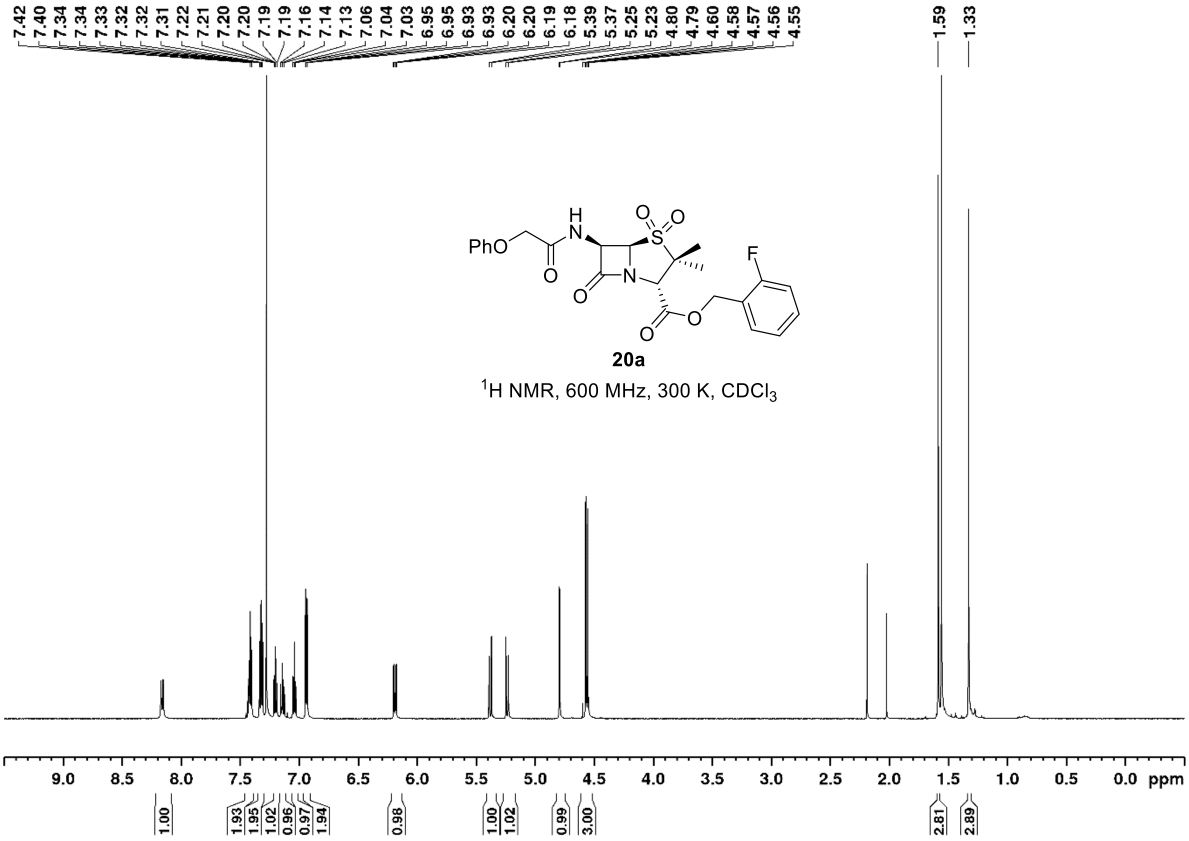
7.42 7.40 7.34 7.34 7.33 7.32 7.32 7.31 7.22 7.21 7.20 7.20 7.19 7.19 7.16 7.14 7.13 7.06 7.04 7.03 6.95 6.93 6.93 6.20 6.20 6.19 6.18 5.39 5.37 5.25 5.23 4.80 4.79 4.60 4.58 4.57 4.56 4.55
1.59
1.33
PhO
H
N
O
S
O
O
N
O
O
O
F
20a
^{1}H NMR, 600 MHz, 300 K, $CDCl_3$
9.0 8.5 8.0 7.5 7.0 6.5 6.0 5.5 5.0 4.5 4.0 3.5 3.0 2.5 2.0 1.5 1.0 0.5 0.0 ppm
1.00 1.93 1.95 1.02 0.96 0.97 1.94 0.98 1.00 1.02 0.99 3.00 2.81 2.89

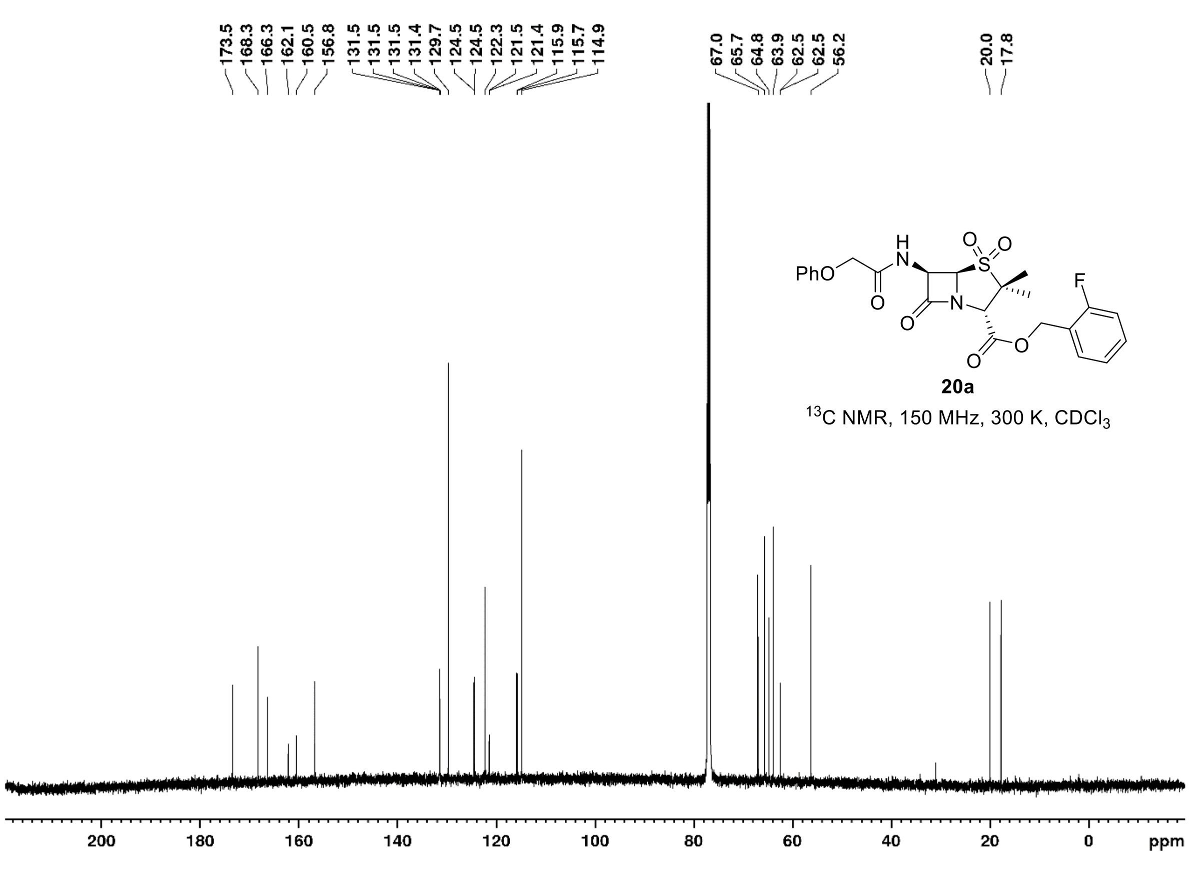
173.5 168.3 166.3 162.1 160.5 156.8
131.5 131.5 131.5 131.4 129.7 124.5 124.5 122.3 121.5 121.4 115.9 115.7 114.9
67.0 65.7 64.8 63.9 62.5 62.5 56.2
20.0 17.8
PhO
H
N
O
S
O
O
N
O
O
O
F
20a
^{13}C NMR, 150 MHz, 300 K, $CDCl_3$
200 180 160 140 120 100 80 60 40 20 0 ppm

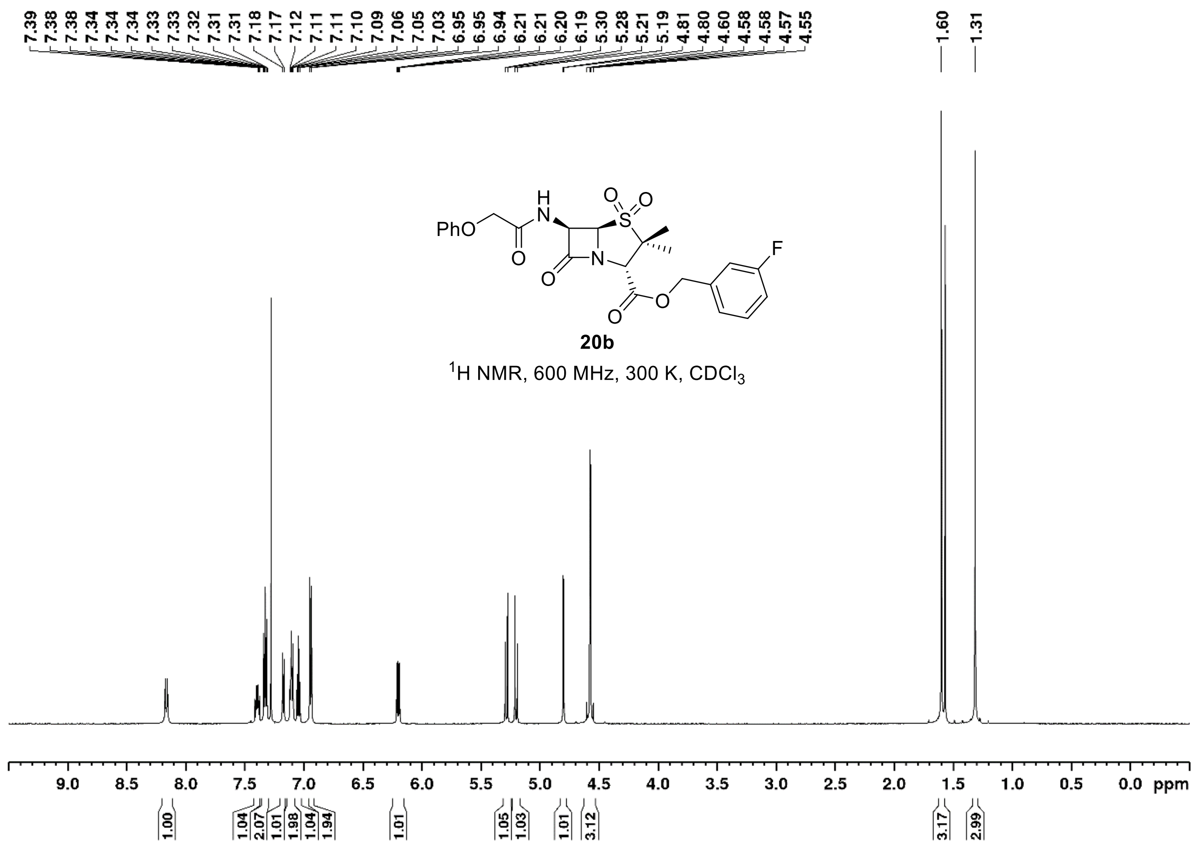

20b

^{1}H NMR, 600 MHz, 300 K, $CDCl_3$

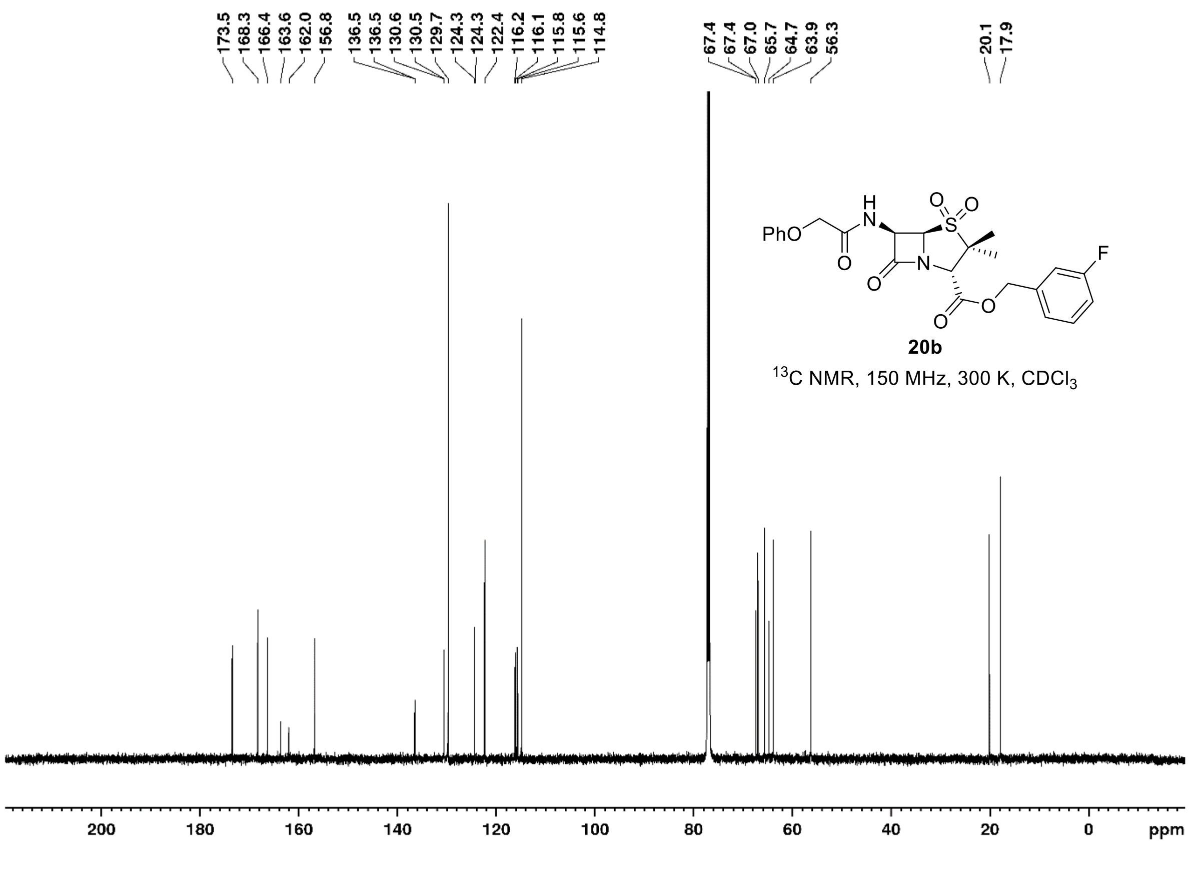

20b

^{13}C NMR, 150 MHz, 300 K, $CDCl_3$

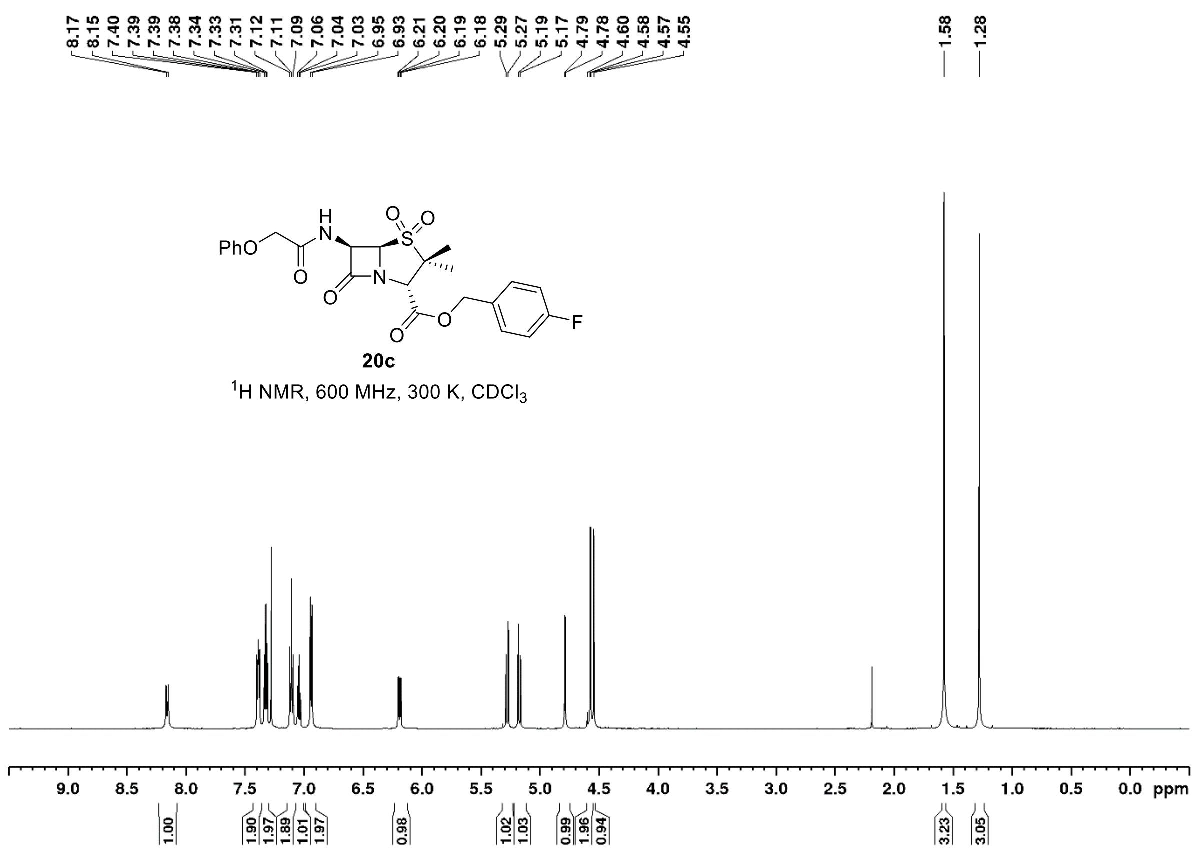
8.17 8.15 7.40 7.39 7.39 7.38 7.34 7.33 7.31 7.12 7.11 7.09 7.06 7.04 7.03 6.95 6.93 6.21 6.20 6.19 6.18 5.29 5.27 5.19 5.17 4.79 4.78 4.60 4.58 4.57 4.55
1.58 1.28
PhO
H
N
O
S
N
F
20c
^{1}H NMR, 600 MHz, 300 K, $CDCl_3$
9.0 8.5 8.0 7.5 7.0 6.5 6.0 5.5 5.0 4.5 4.0 3.5 3.0 2.5 2.0 1.5 1.0 0.5 0.0 ppm
1.00 1.90 1.97 1.89 1.01 1.97 0.98 1.02 1.03 0.99 1.96 0.94 3.23 3.05

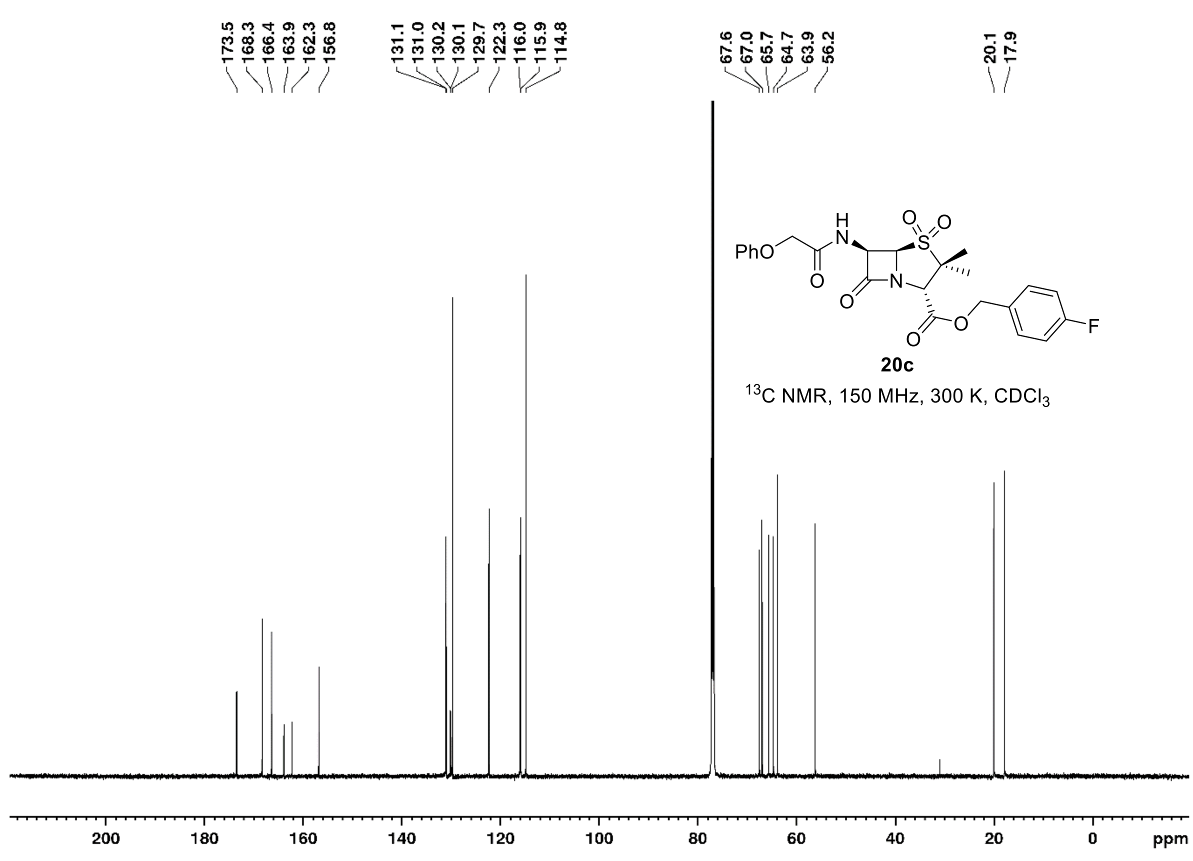
173.5 168.3 166.4 163.9 162.3 156.8
131.1 131.0 130.2 130.1 129.7 122.3 116.0 115.9 114.8
67.6 67.0 65.7 64.7 63.9 56.2
20.1 17.9
PhO
H
N
O
S
N
F
20c
^{13}C NMR, 150 MHz, 300 K, $CDCl_3$
200 180 160 140 120 100 80 60 40 20 0 ppm

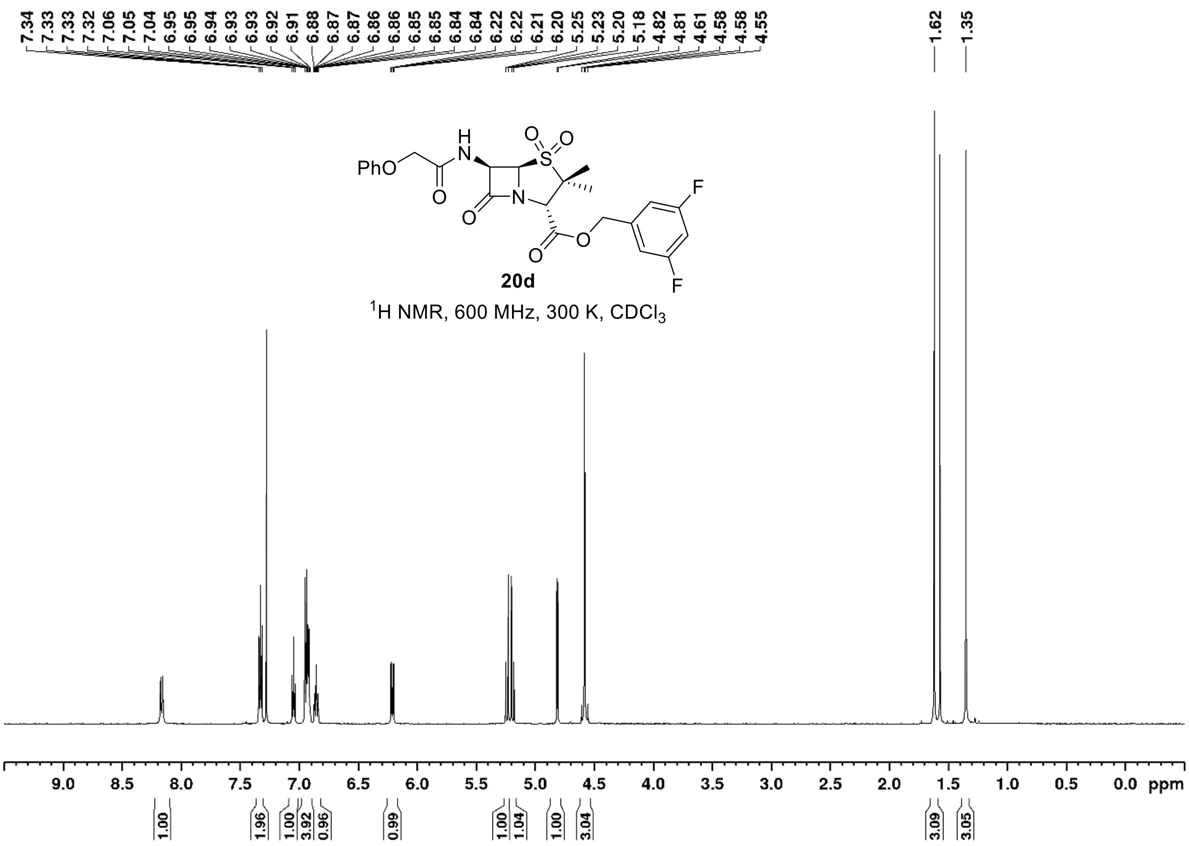
7.34
7.33
7.33
7.32
7.06
7.05
7.04
6.95
6.95
6.94
6.93
6.93
6.92
6.91
6.88
6.87
6.87
6.86
6.86
6.85
6.85
6.84
6.22
6.22
6.21
6.20
5.25
5.23
5.20
5.18
4.82
4.81
4.61
4.58
4.58
4.55
1.62
1.35
PhO
H
N
O
S
O
O
N
O
O
O
F
F
20d
1H NMR, 600 MHz, 300 K, CDCl3
9.0 8.5 8.0 7.5 7.0 6.5 6.0 5.5 5.0 4.5 4.0 3.5 3.0 2.5 2.0 1.5 1.0 0.5 0.0 ppm
1.00
1.96
1.00
3.92
0.96
0.99
1.00
1.04
1.00
3.04
3.09
3.05

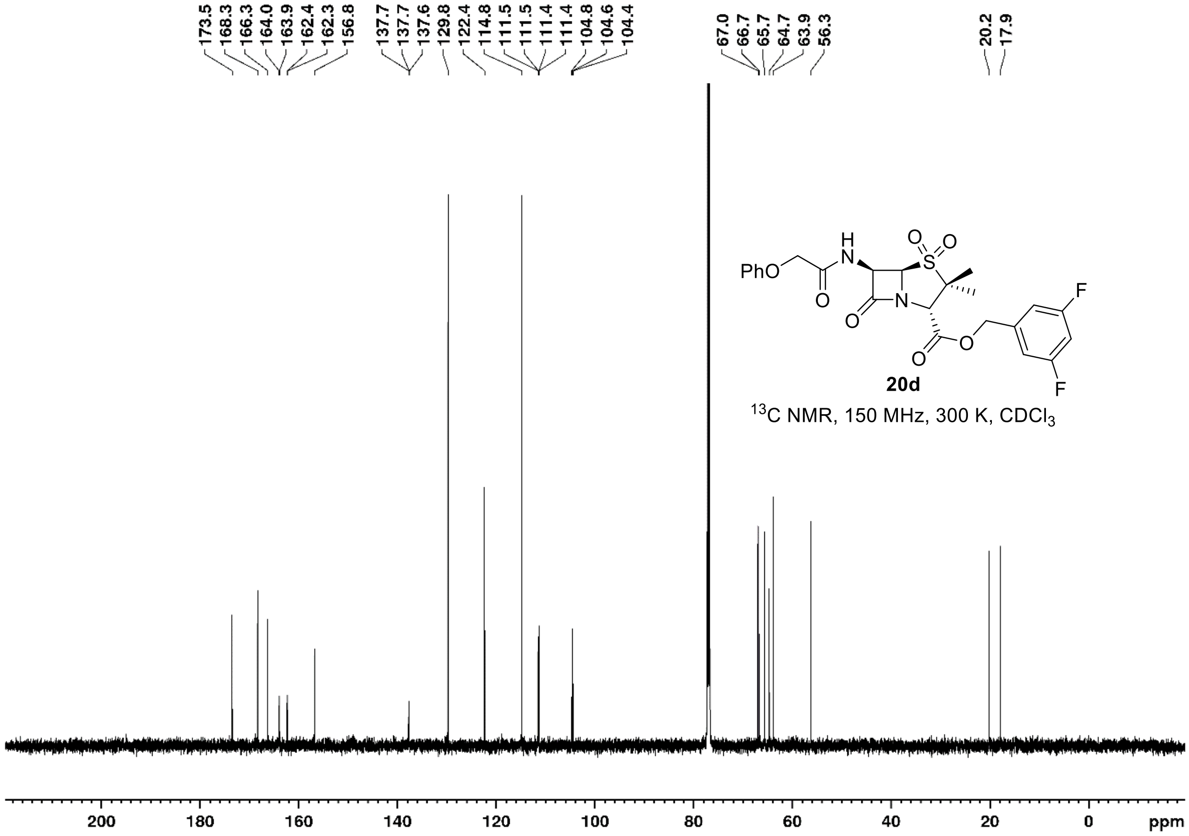
173.5
168.3
166.3
164.0
163.9
162.4
162.3
156.8
137.7
137.7
137.6
129.8
122.4
114.8
111.5
111.5
111.4
111.4
104.8
104.6
104.4
67.0
66.7
65.7
64.7
63.9
56.3
20.2
17.9
PhO
H
N
O
S
O
O
N
O
O
O
F
F
20d
13C NMR, 150 MHz, 300 K, CDCl3
200 180 160 140 120 100 80 60 40 20 0 ppm

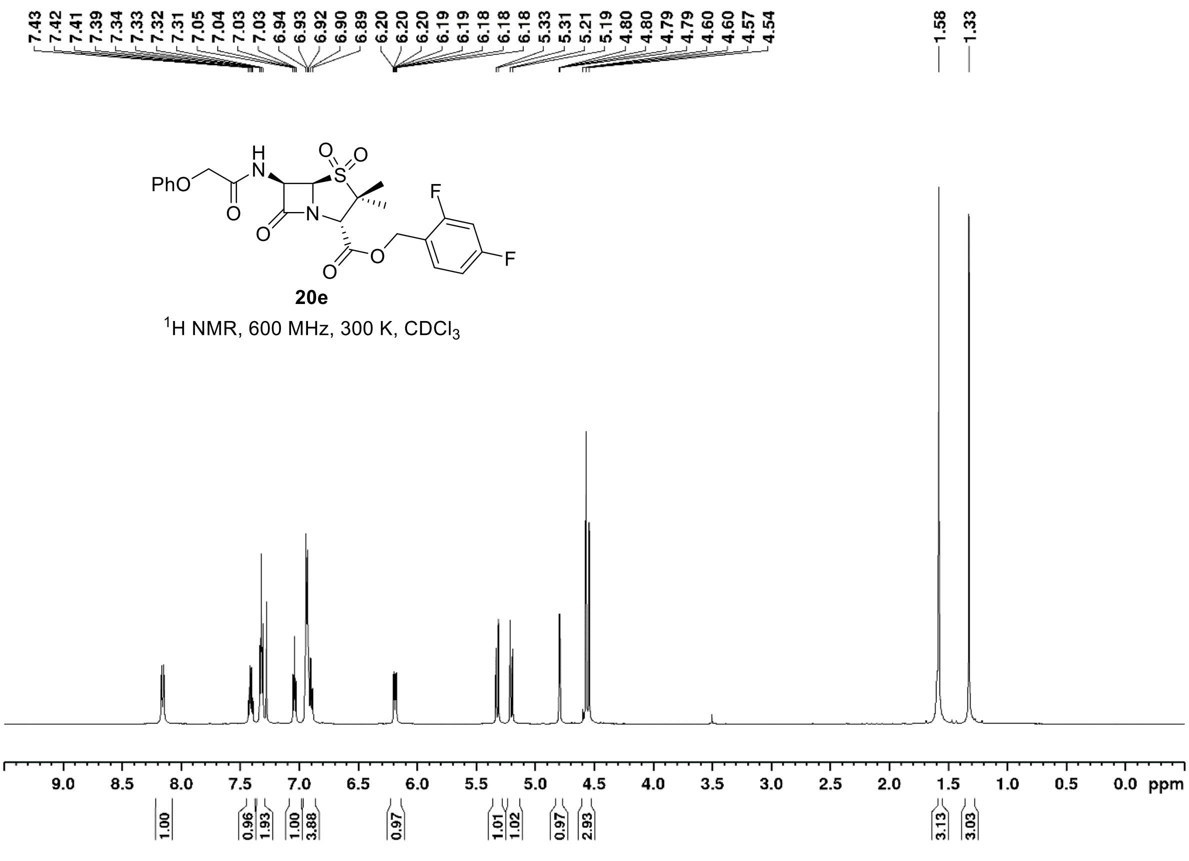
7.43 7.42 7.41 7.39 7.34 7.33 7.32 7.31 7.05 7.04 7.03 6.94 6.93 6.92 6.90 6.89 6.20 6.20 6.20 6.19 6.19 6.18 6.18 6.18 5.33 5.31 5.21 5.19 4.80 4.80 4.79 4.79 4.60 4.60 4.57 4.54
1.58 1.33
PhO
H
N
O
S
O
O
N
O
F
F
20e
1H NMR, 600 MHz, 300 K, CDCl3
9.0 8.5 8.0 7.5 7.0 6.5 6.0 5.5 5.0 4.5 4.0 3.5 3.0 2.5 2.0 1.5 1.0 0.5 0.0 ppm
1.00 0.96 1.93 1.00 3.88 0.97 1.01 1.02 0.97 2.93 3.13 3.03

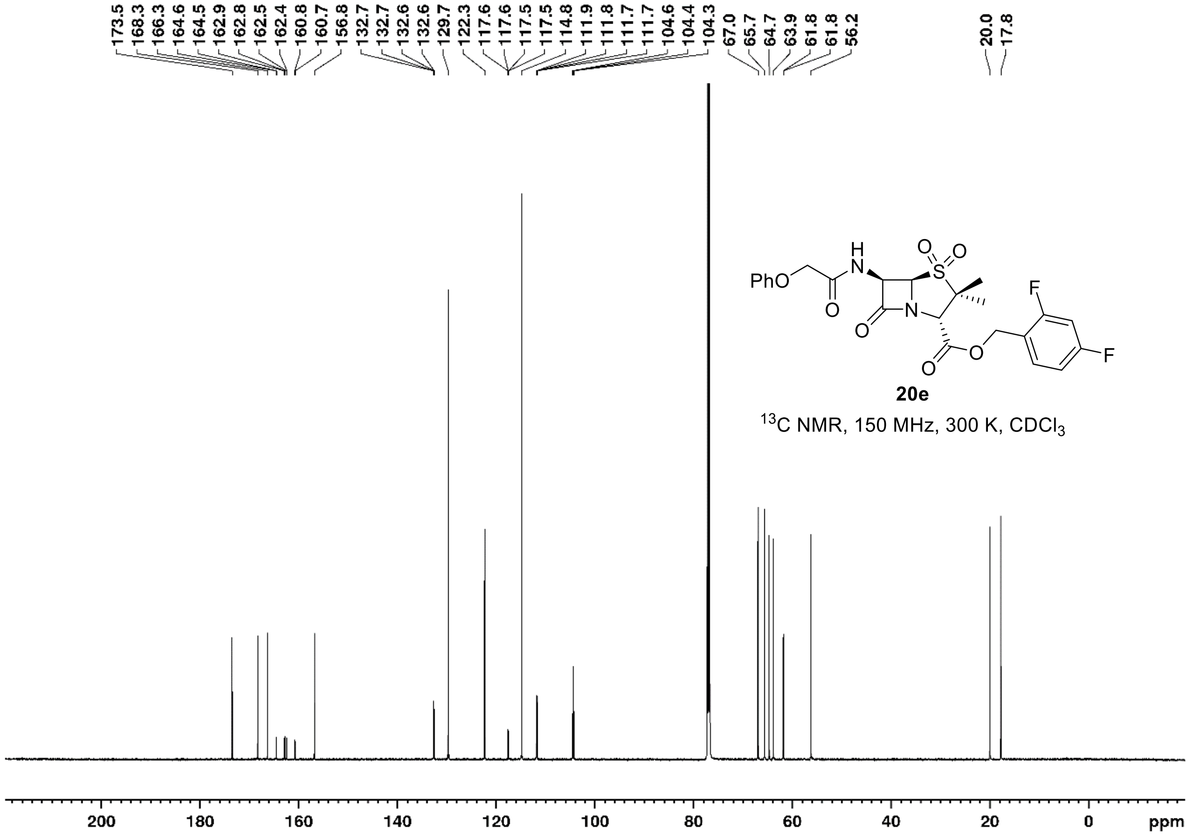
173.5 168.3 166.3 164.6 164.5 162.9 162.8 162.5 162.4 160.8 160.7 156.8 132.7 132.7 132.6 132.6 129.7 122.3 117.6 117.6 117.5 117.5 114.8 111.9 111.8 111.7 111.7 104.6 104.4 104.3 67.0 65.7 64.7 63.9 61.8 56.2
20.0 17.8
PhO
H
N
O
S
O
O
N
O
F
F
20e
13C NMR, 150 MHz, 300 K, CDCl3
200 180 160 140 120 100 80 60 40 20 0 ppm

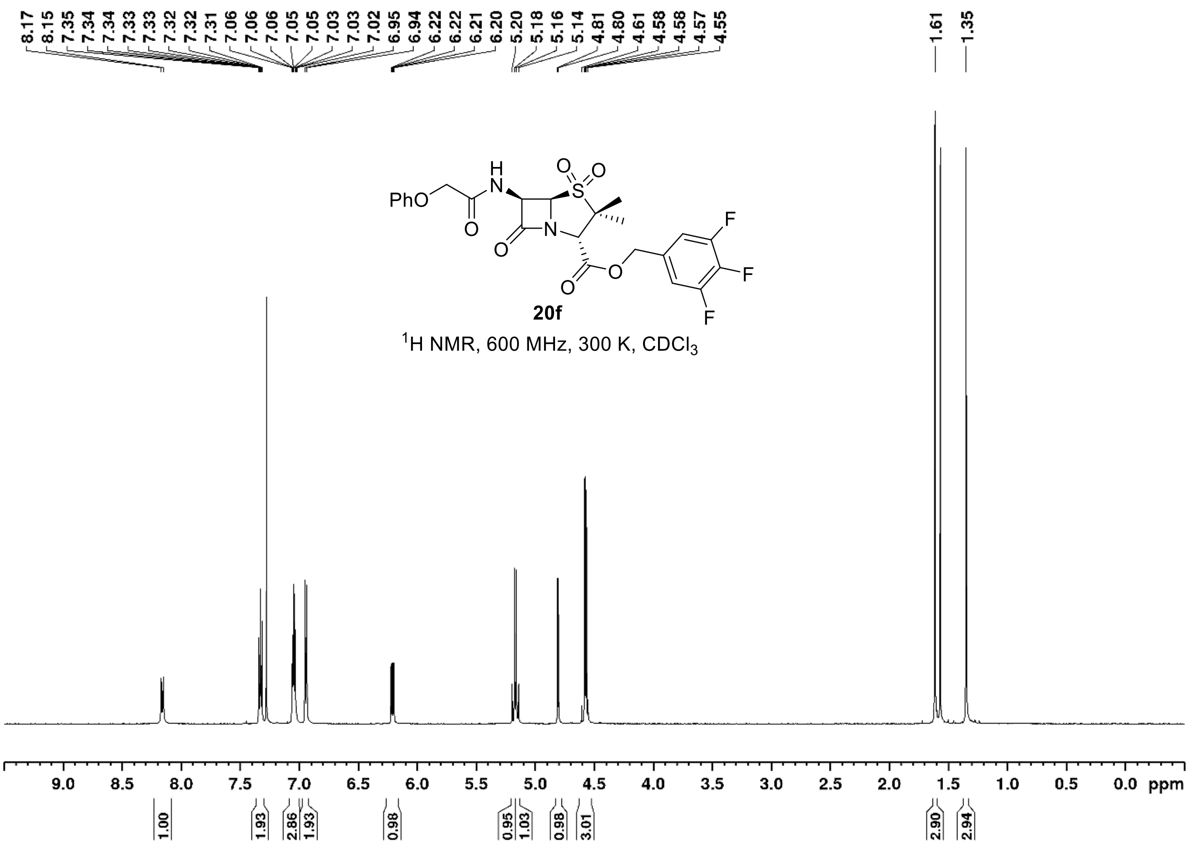

20f

^1H NMR, 600 MHz, 300 K, $CDCl_3$

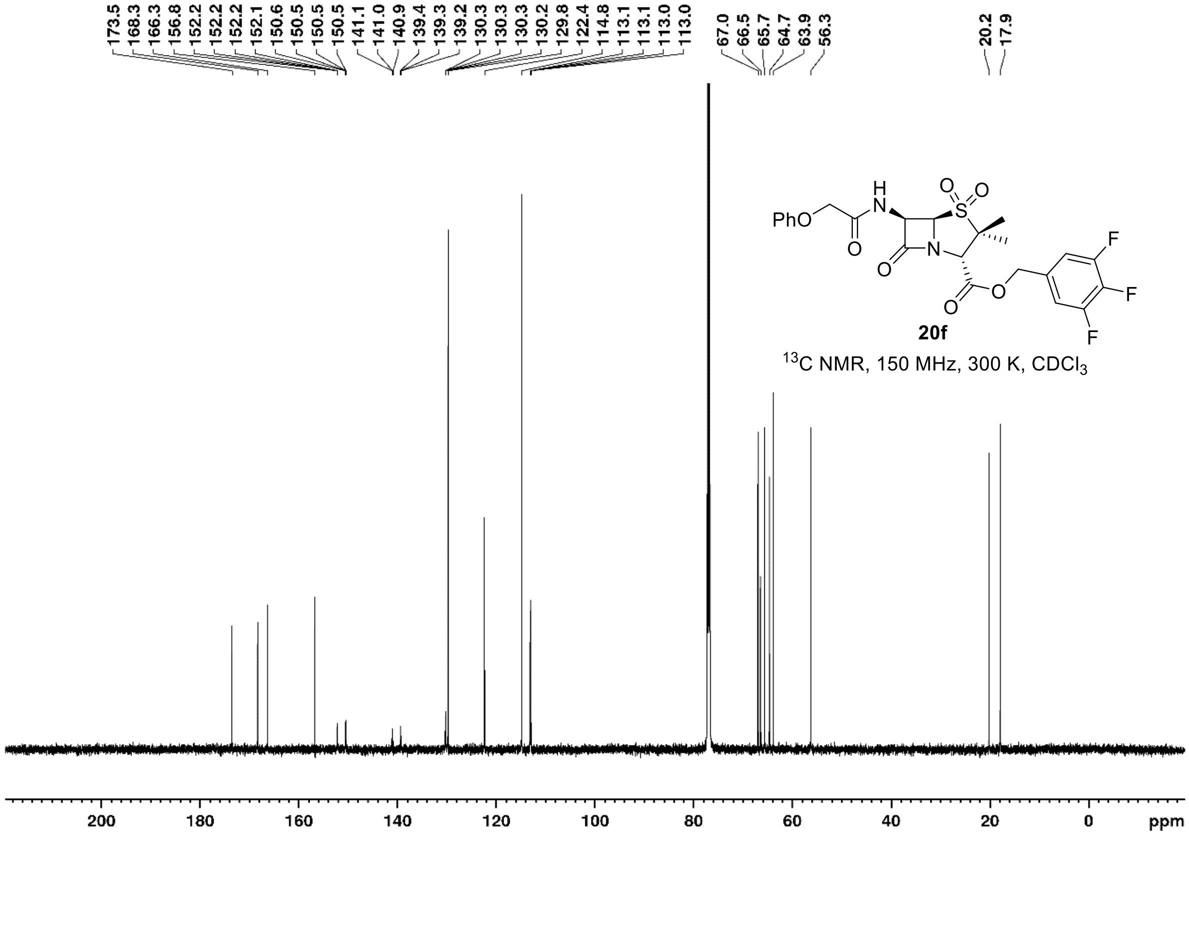

20f

^{13}C NMR, 150 MHz, 300 K, $CDCl_3$

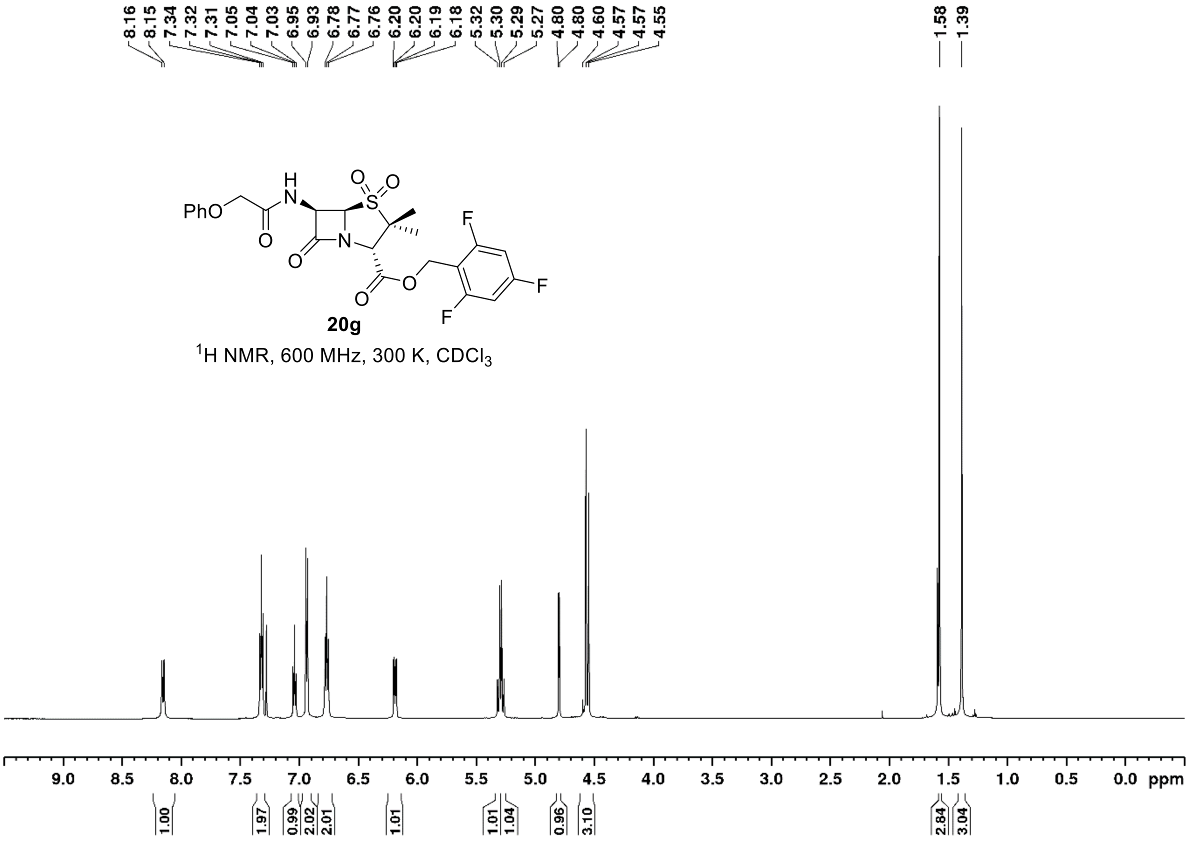
8.16 8.15 7.34 7.32 7.31 7.05 7.04 7.03 6.95 6.93 6.78 6.77 6.76 6.20 6.20 6.19 6.18 5.32 5.30 5.29 5.27 4.80 4.80 4.60 4.57 4.57 4.55
1.58 1.39
PhO
H
N
O
S
N
F
20g
1H NMR, 600 MHz, 300 K, CDCl3
9.0 8.5 8.0 7.5 7.0 6.5 6.0 5.5 5.0 4.5 4.0 3.5 3.0 2.5 2.0 1.5 1.0 0.5 0.0 ppm
1.00 1.97 0.99 2.02 2.01 1.01 1.01 1.04 0.96 3.10 2.84 3.04

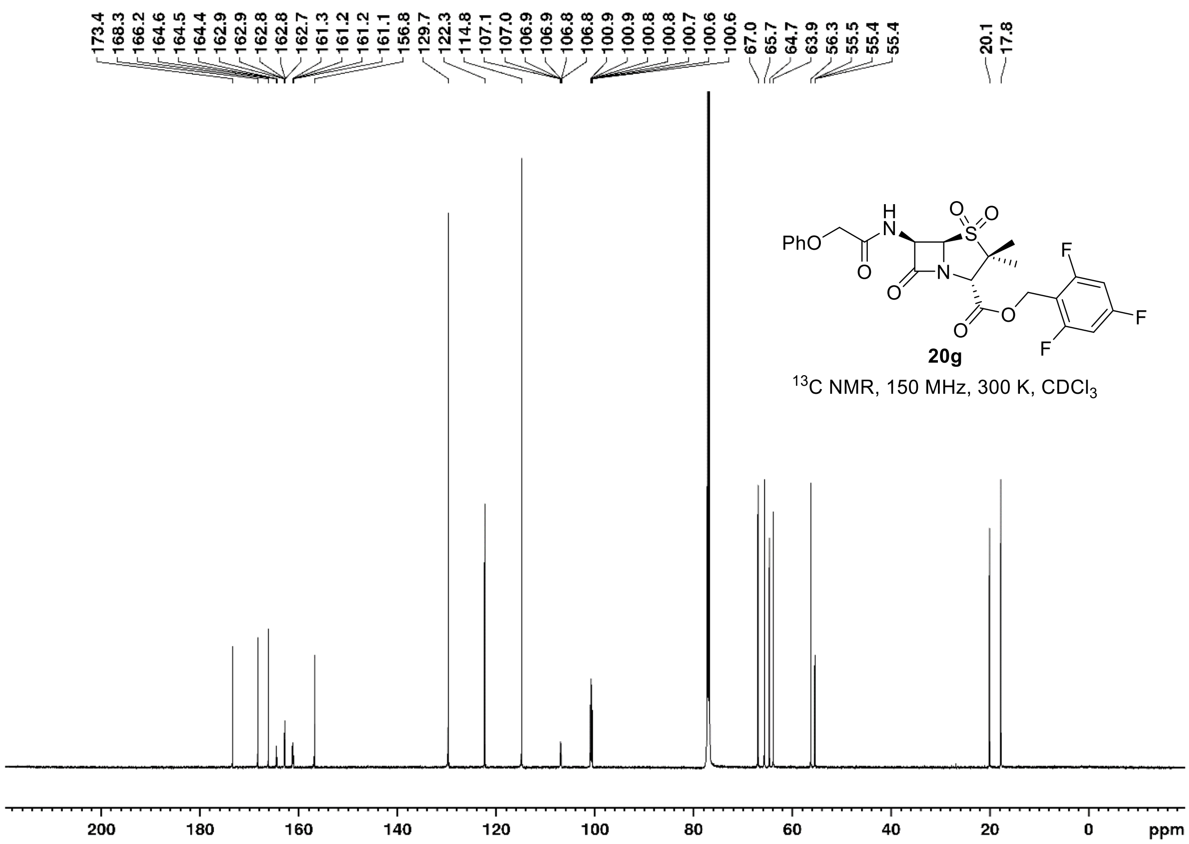
173.4 168.3 166.2 164.6 164.5 164.4 162.9 162.9 162.8 162.8 162.7 161.3 161.2 161.2 161.1 156.8 129.7 122.3 114.8 107.1 107.0 106.9 106.9 106.8 106.8 100.9 100.9 100.8 100.8 100.7 100.6 100.6 67.0 65.7 64.7 63.9 56.3 55.5 55.4 55.4
20.1 17.8
PhO
H
N
O
S
N
F
20g
13C NMR, 150 MHz, 300 K, CDCl3
200 180 160 140 120 100 80 60 40 20 0 ppm

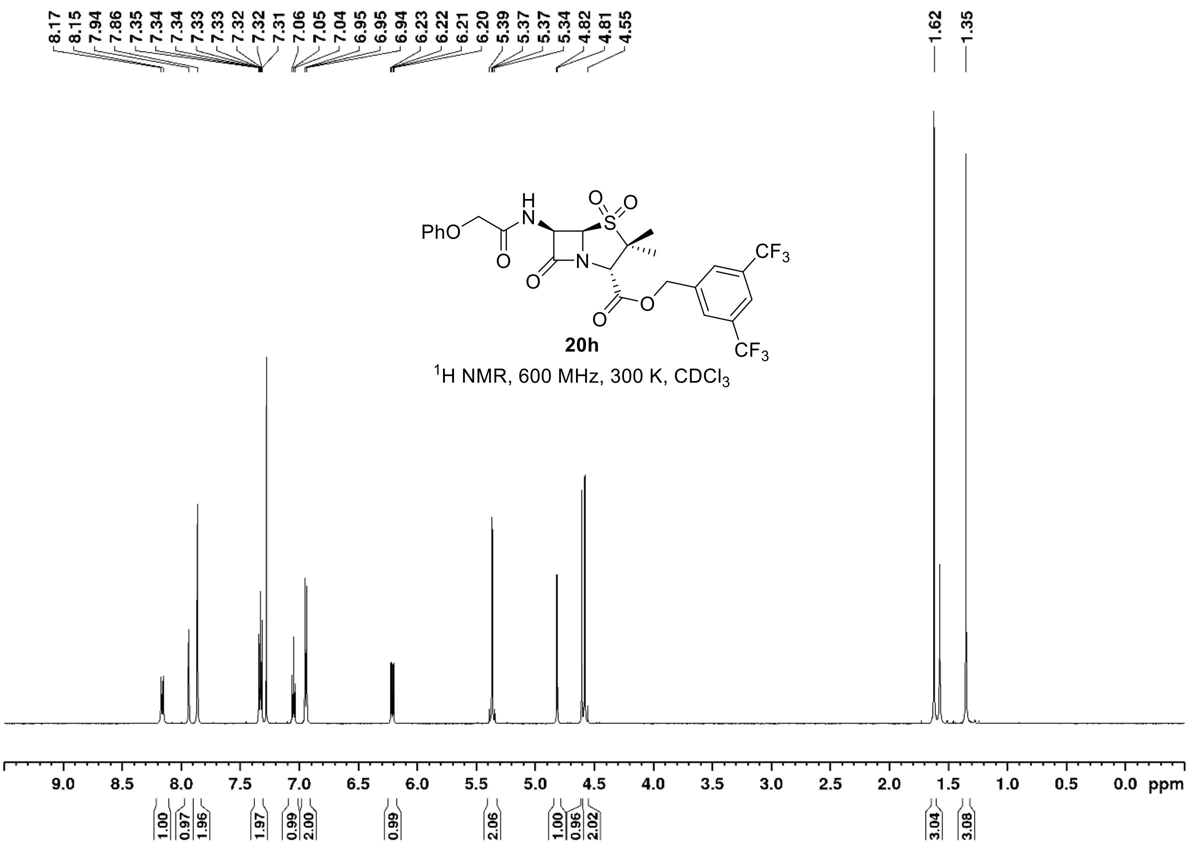

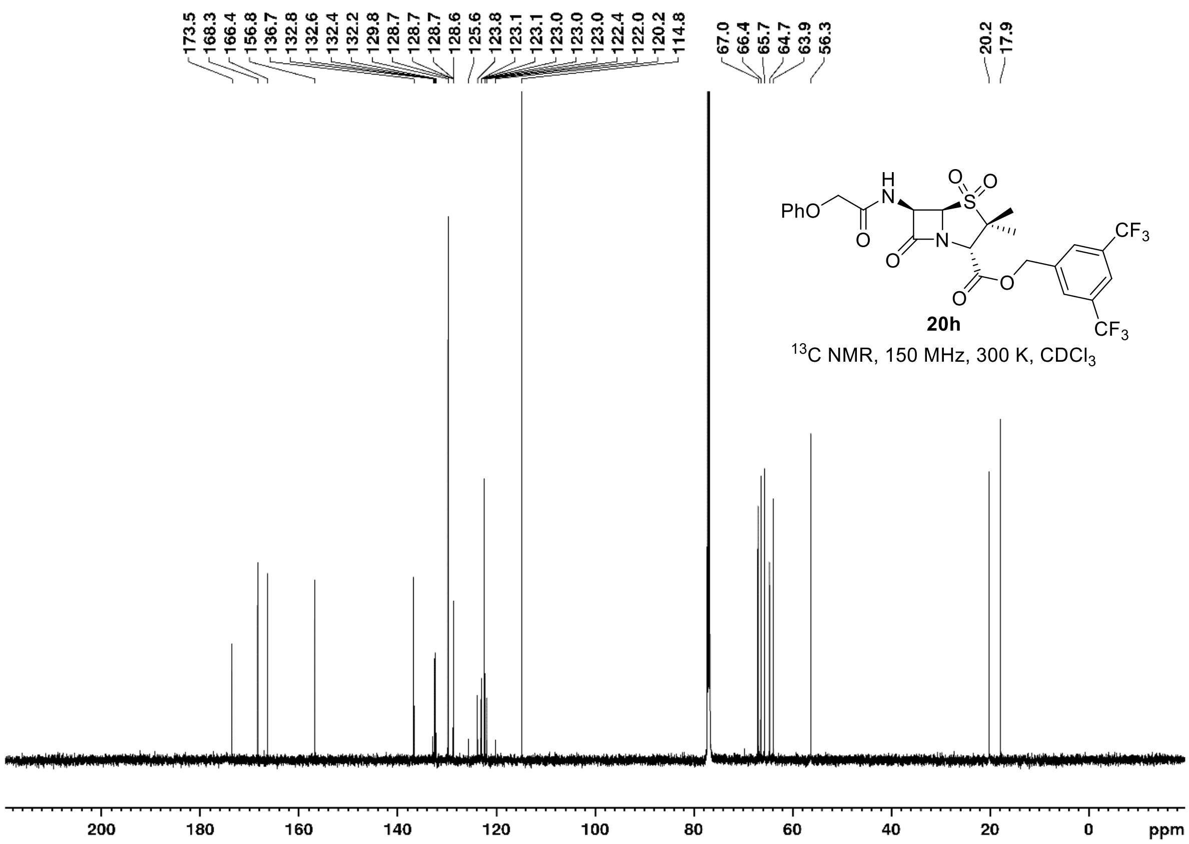

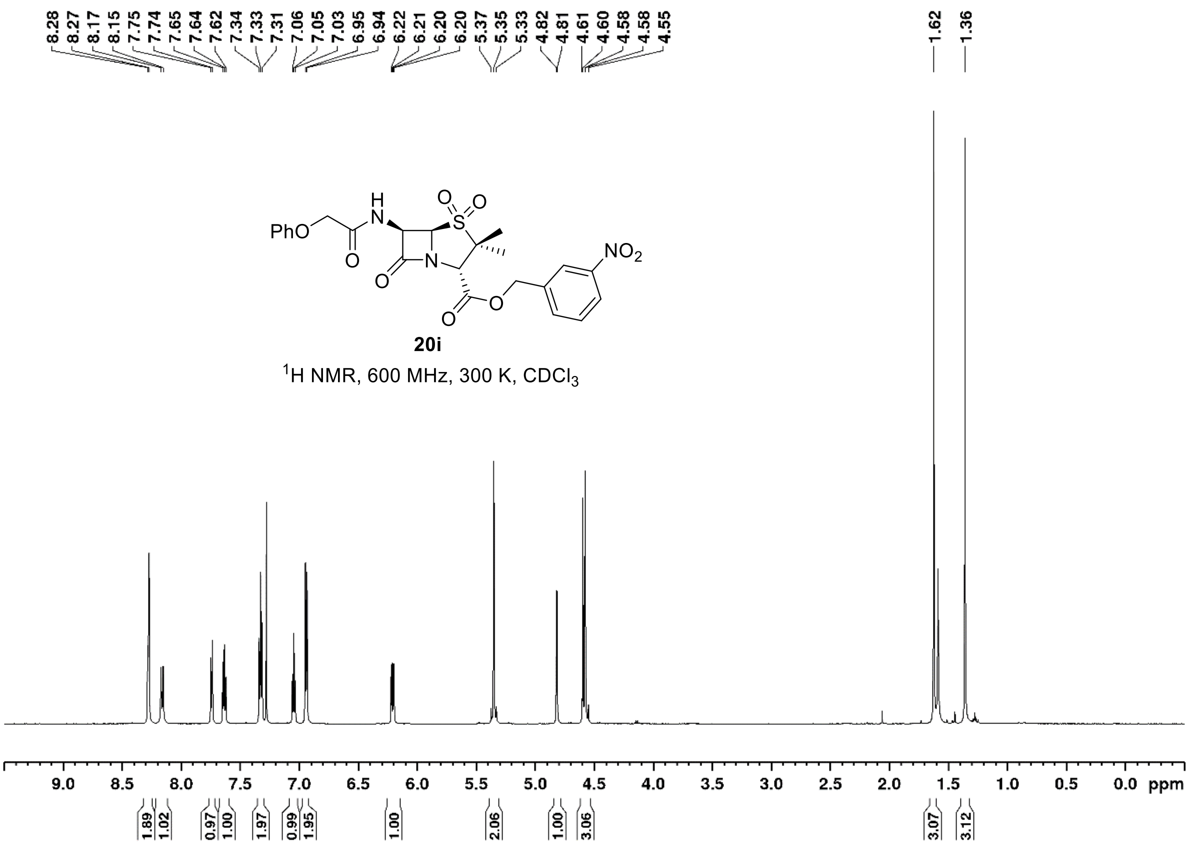
8.28 8.27 8.17 8.15 7.75 7.74 7.65 7.64 7.62 7.34 7.33 7.31 7.06 7.05 7.03 6.95 6.94 6.22 6.21 6.20 6.20 5.37 5.35 5.33 4.82 4.81 4.61 4.60 4.58 4.58 4.55
1.62
1.36
PhO
H
N
O
S
NO2
20i
^{1}H NMR, 600 MHz, 300 K, CDCl$_3$
1.89 1.02 0.97 1.00 1.97 0.99 1.95 1.00 2.06 1.00 3.06 3.07 3.12
9.0 8.5 8.0 7.5 7.0 6.5 6.0 5.5 5.0 4.5 4.0 3.5 3.0 2.5 2.0 1.5 1.0 0.5 0.0 ppm

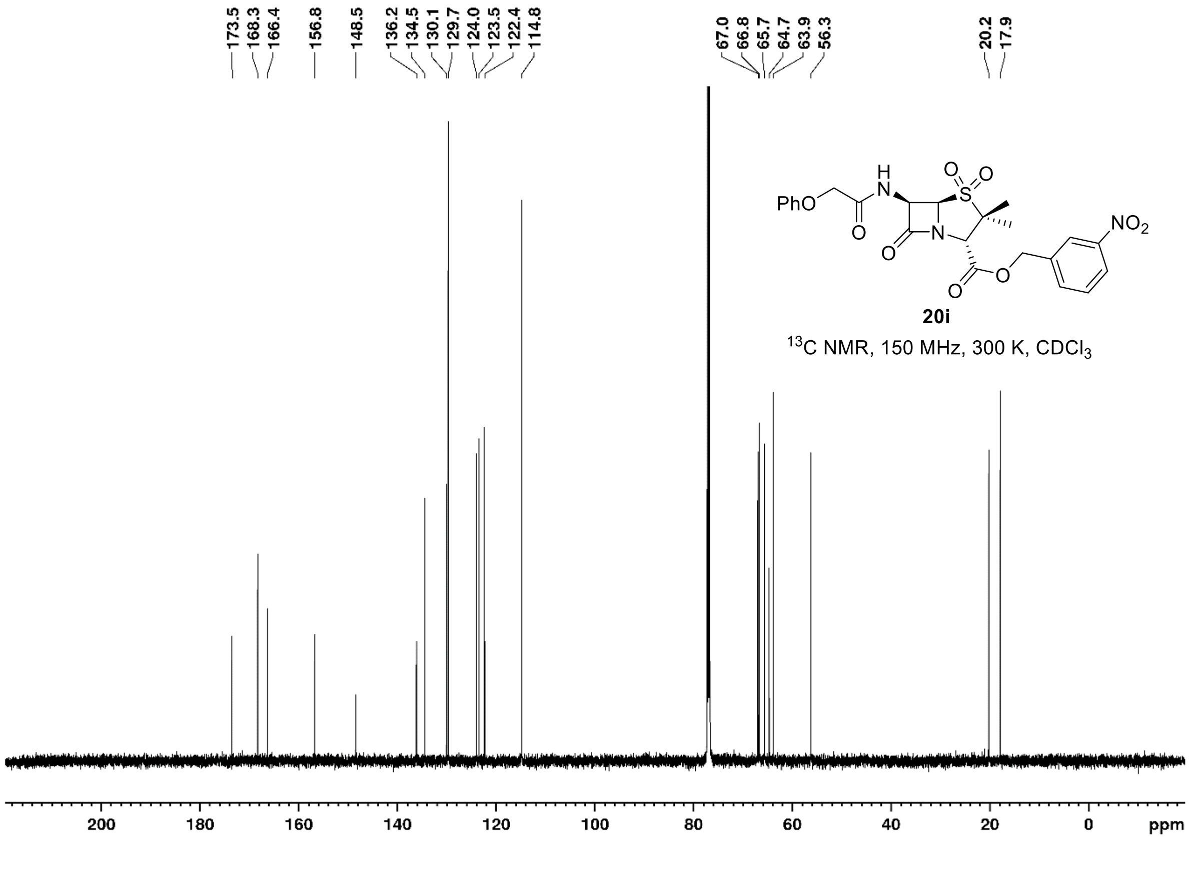
173.5 168.3 166.4 156.8 148.5 136.2 134.5 130.1 129.7 124.0 123.5 122.4 114.8
67.0 66.8 65.7 64.7 63.9 56.3
20.2 17.9
PhO
H
N
O
S
NO2
20i
^{13}C NMR, 150 MHz, 300 K, CDCl$_3$
200 180 160 140 120 100 80 60 40 20 0 ppm

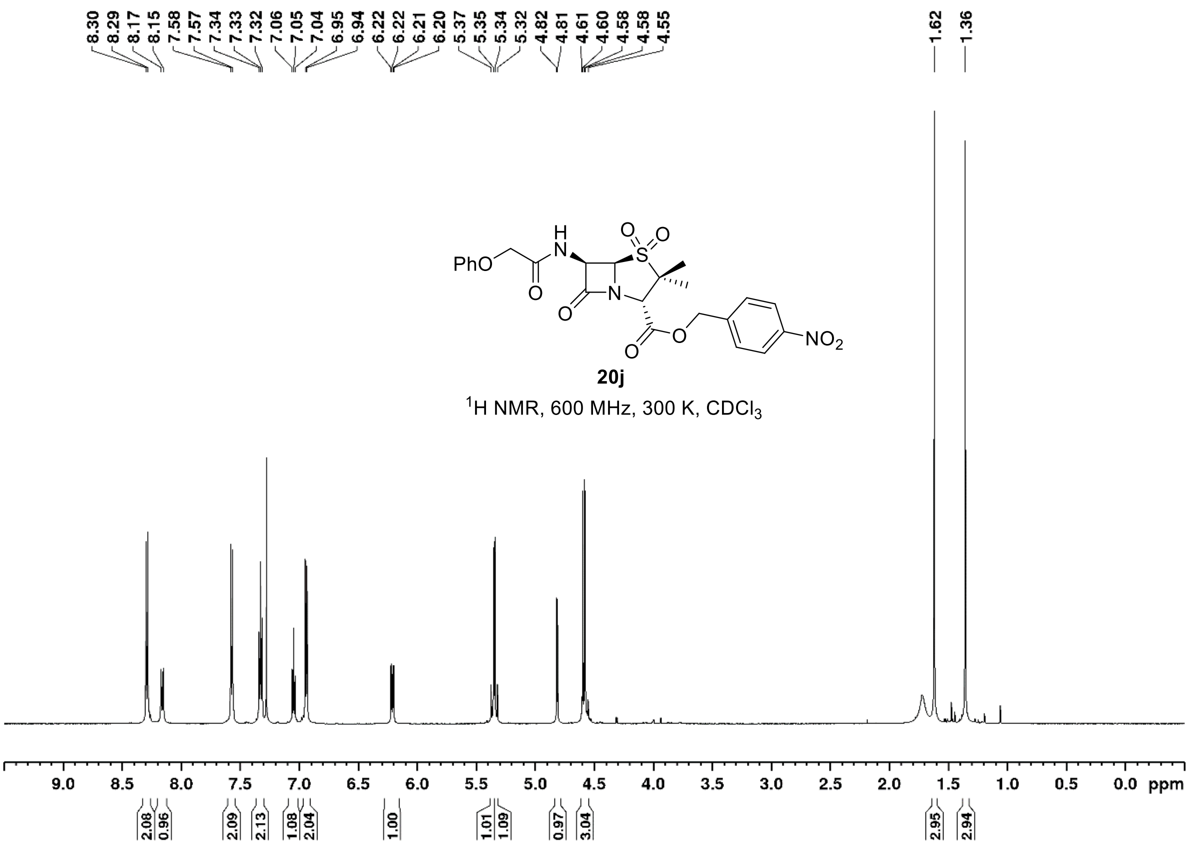
8.30 8.29 8.17 8.15 7.58 7.57 7.34 7.33 7.32 7.06 7.05 7.04 6.95 6.94 6.22 6.22 6.21 6.20 5.37 5.35 5.34 5.32 4.82 4.81 4.61 4.60 4.58 4.58 4.55
1.62 1.36
PhO
H
N
O
S
N
NO2
20j
1H NMR, 600 MHz, 300 K, CDCl3
9.0 8.5 8.0 7.5 7.0 6.5 6.0 5.5 5.0 4.5 4.0 3.5 3.0 2.5 2.0 1.5 1.0 0.5 0.0 ppm
2.08 0.96 2.09 2.13 1.08 2.04 1.00 1.01 1.09 0.97 3.04 2.95 2.94

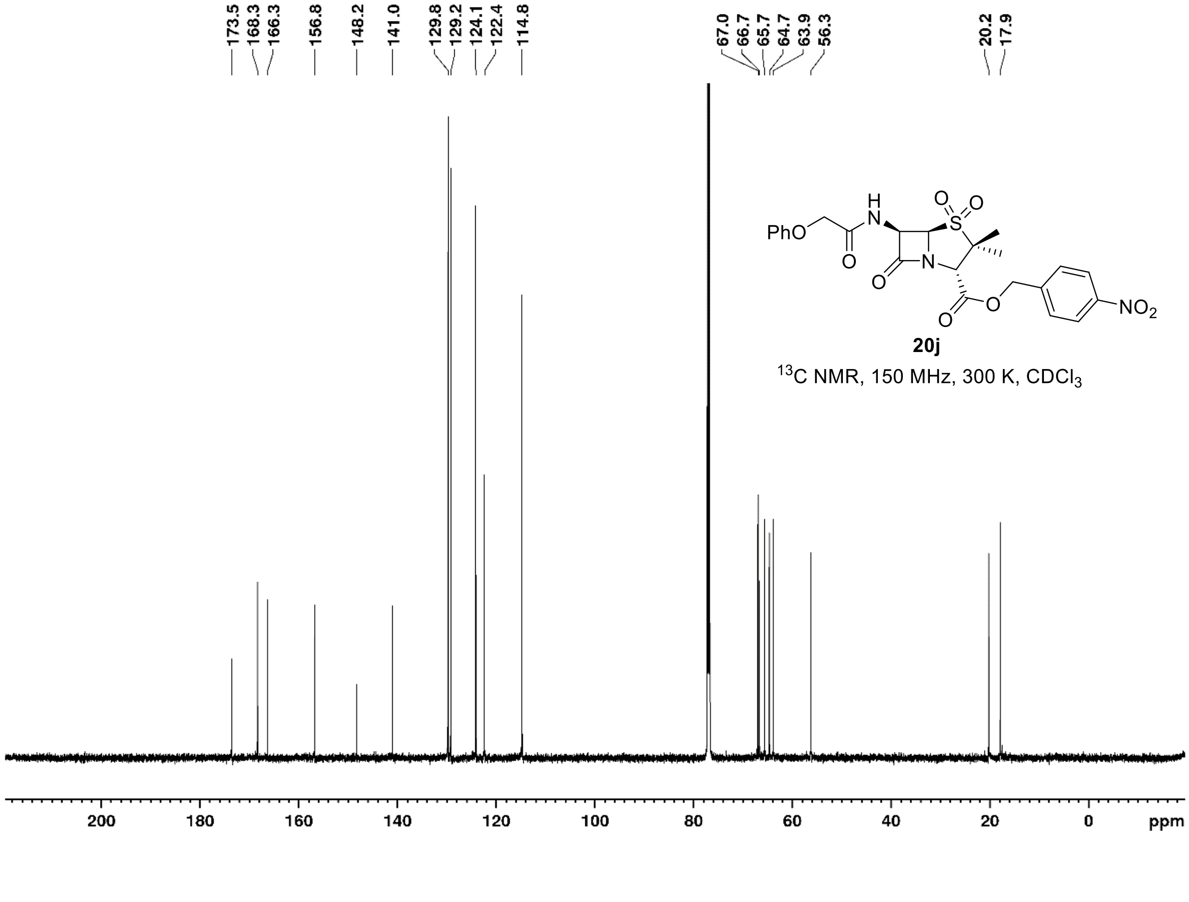
173.5 168.3 166.3 156.8 148.2 141.0 129.8 129.2 124.1 122.4 114.8
67.0 66.7 65.7 64.7 63.9 56.3
20.2 17.9
PhO
H
N
O
S
N
NO2
20j
13C NMR, 150 MHz, 300 K, CDCl3
200 180 160 140 120 100 80 60 40 20 0 ppm

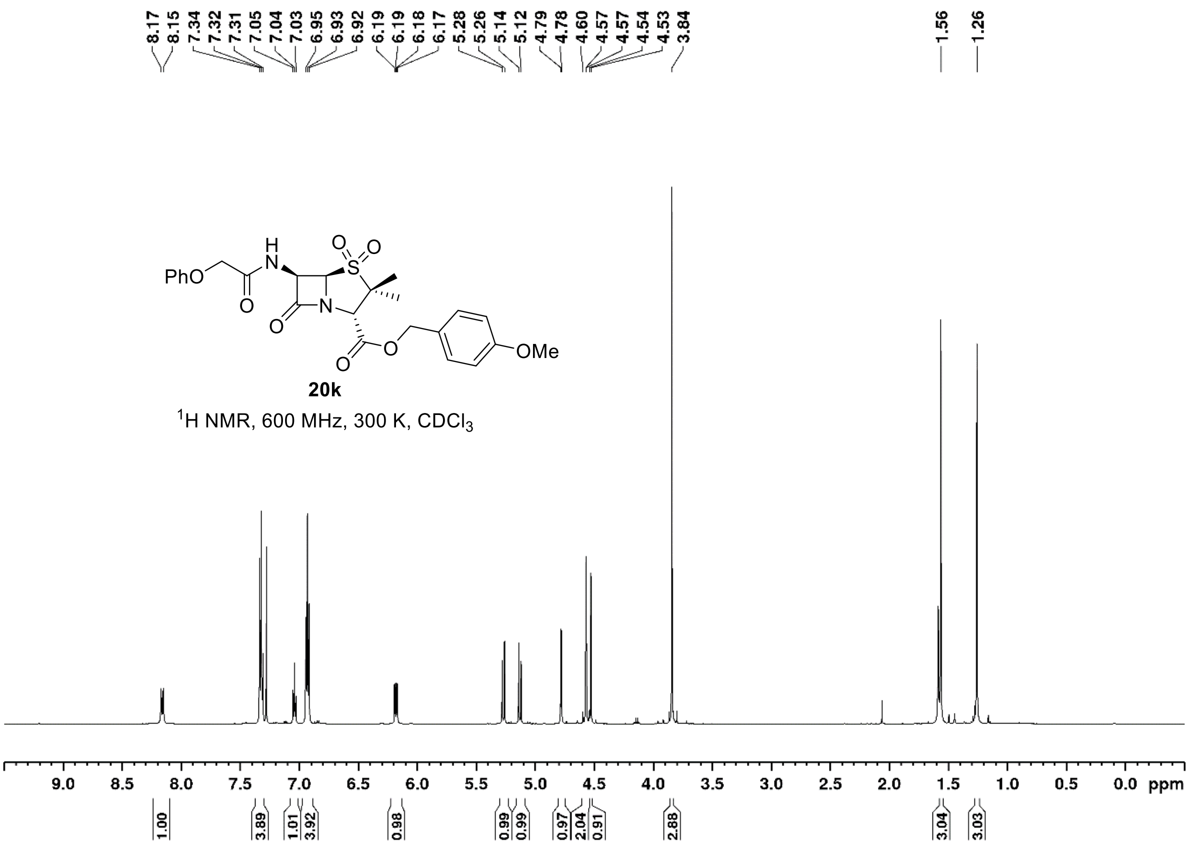
8.17 8.15 7.34 7.32 7.31 7.05 7.04 7.03 6.95 6.93 6.92 6.19 6.19 6.18 6.17 5.28 5.26 5.14 5.12 4.79 4.78 4.60 4.57 4.57 4.54 4.53 3.84
1.56 1.26
PhO
H
N
O
O
S
O
N
O
O
O
OMe
20k
^{1}H NMR, 600 MHz, 300 K, $CDCl_3$
9.0 8.5 8.0 7.5 7.0 6.5 6.0 5.5 5.0 4.5 4.0 3.5 3.0 2.5 2.0 1.5 1.0 0.5 0.0 ppm
1.00 3.89 1.01 3.92 0.98 0.99 0.99 0.97 2.04 0.91 2.88 3.04 3.03

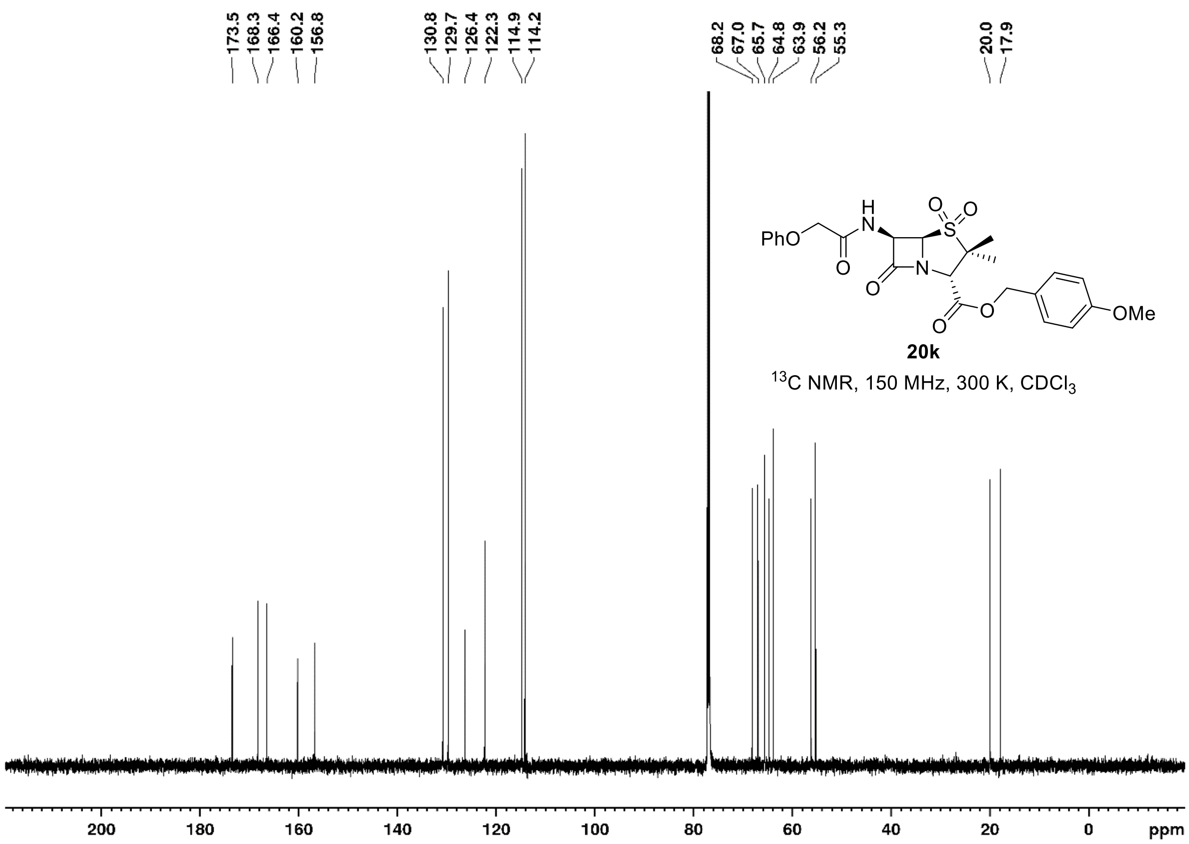
173.5 168.3 166.4 160.2 156.8 130.8 129.7 126.4 122.3 114.9 114.2 68.2 67.0 65.7 64.8 63.9 56.2 55.3 20.0 17.9
PhO
H
N
O
O
S
O
N
O
O
O
OMe
20k
^{13}C NMR, 150 MHz, 300 K, $CDCl_3$
200 180 160 140 120 100 80 60 40 20 0 ppm

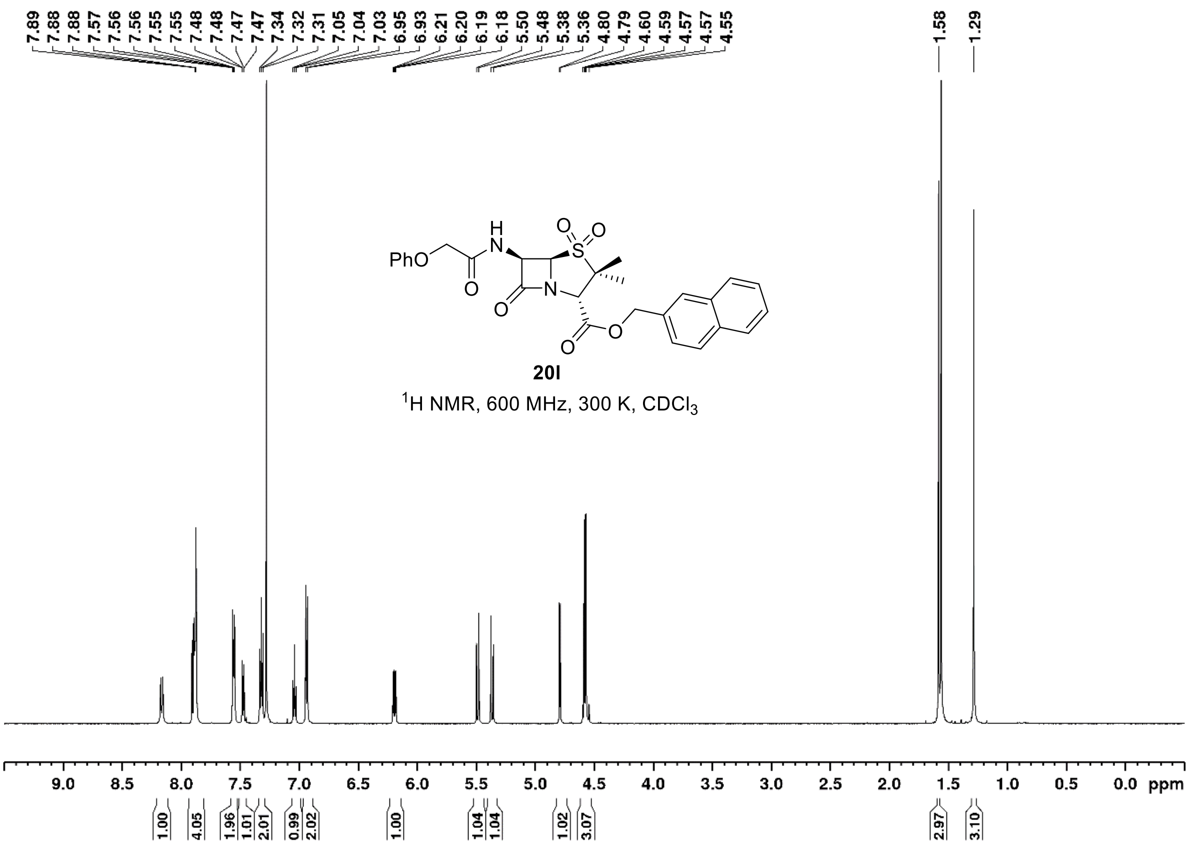
PhO
20l
^{1}H NMR, 600 MHz, 300 K, $CDCl_3$

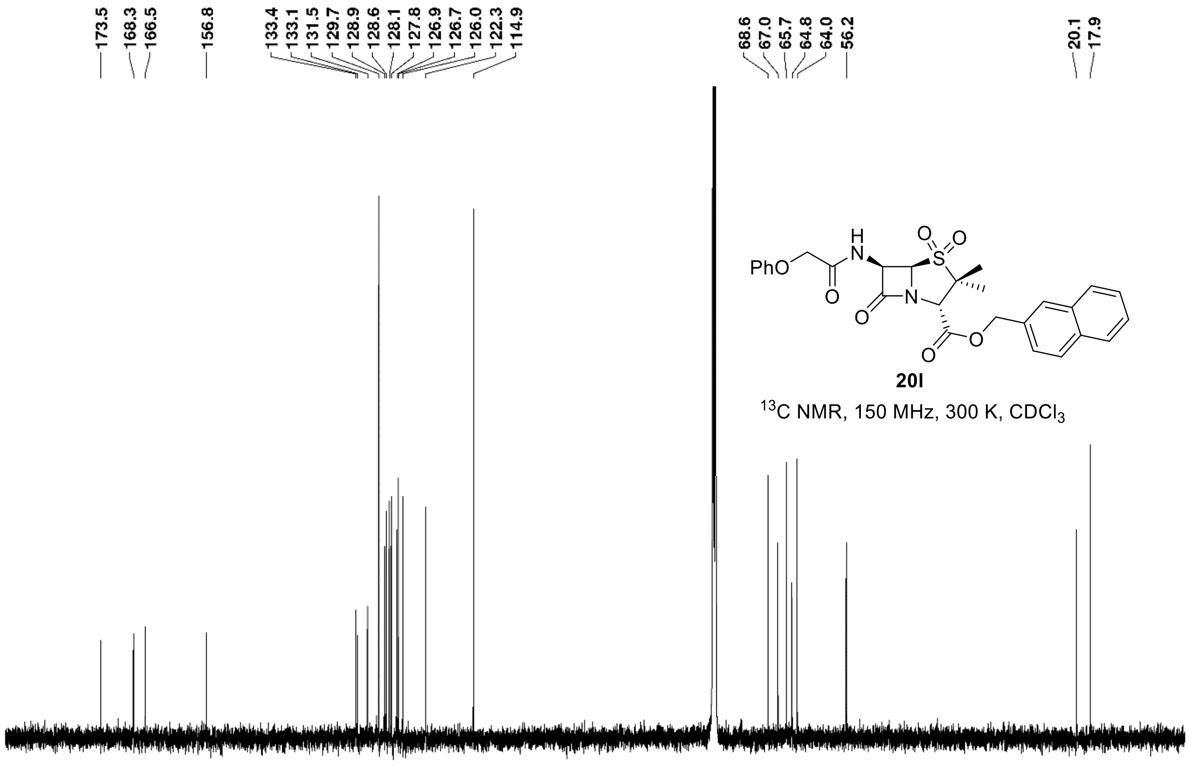
PhO
20l
^{13}C NMR, 150 MHz, 300 K, $CDCl_3$

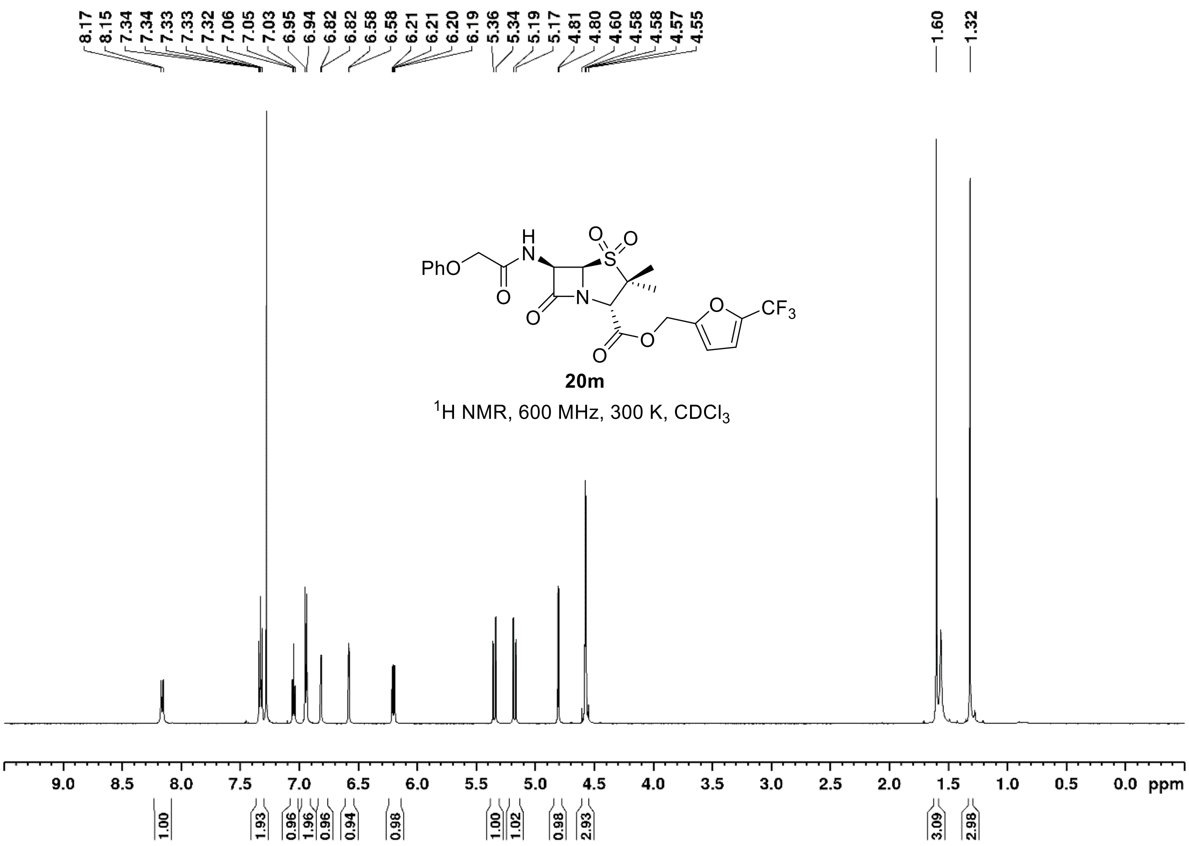
8.17
8.15
7.34
7.33
7.33
7.32
7.06
7.05
7.03
6.95
6.94
6.82
6.82
6.58
6.58
6.21
6.21
6.20
6.19
5.36
5.34
5.19
5.17
4.81
4.80
4.60
4.58
4.58
4.57
4.55
1.60
1.32
PhO
H
N
O
S
O
O
N
O
O
O
O
CF3
20m
1H NMR, 600 MHz, 300 K, CDCl3
9.0 8.5 8.0 7.5 7.0 6.5 6.0 5.5 5.0 4.5 4.0 3.5 3.0 2.5 2.0 1.5 1.0 0.5 0.0 ppm
1.00
1.93
0.96
1.96
0.96
0.94
0.98
1.00
1.02
0.98
2.93
3.09
2.98

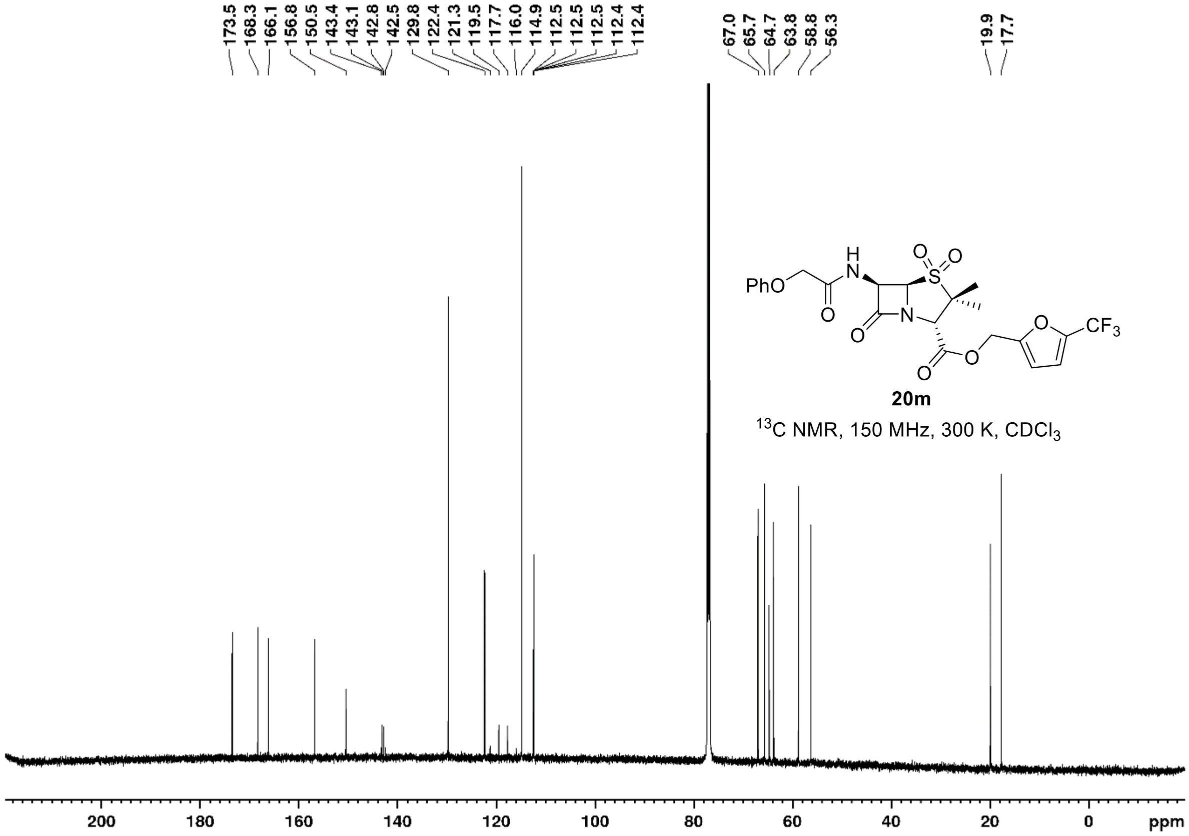
173.5
168.3
166.1
156.8
150.5
143.4
143.1
142.8
142.5
129.8
122.4
121.3
119.5
117.7
116.0
114.9
112.5
112.5
112.5
112.4
112.4
67.0
65.7
64.7
63.8
58.8
56.3
19.9
17.7
PhO
H
N
O
S
O
O
N
O
O
O
O
CF3
20m
13C NMR, 150 MHz, 300 K, CDCl3
200 180 160 140 120 100 80 60 40 20 0 ppm

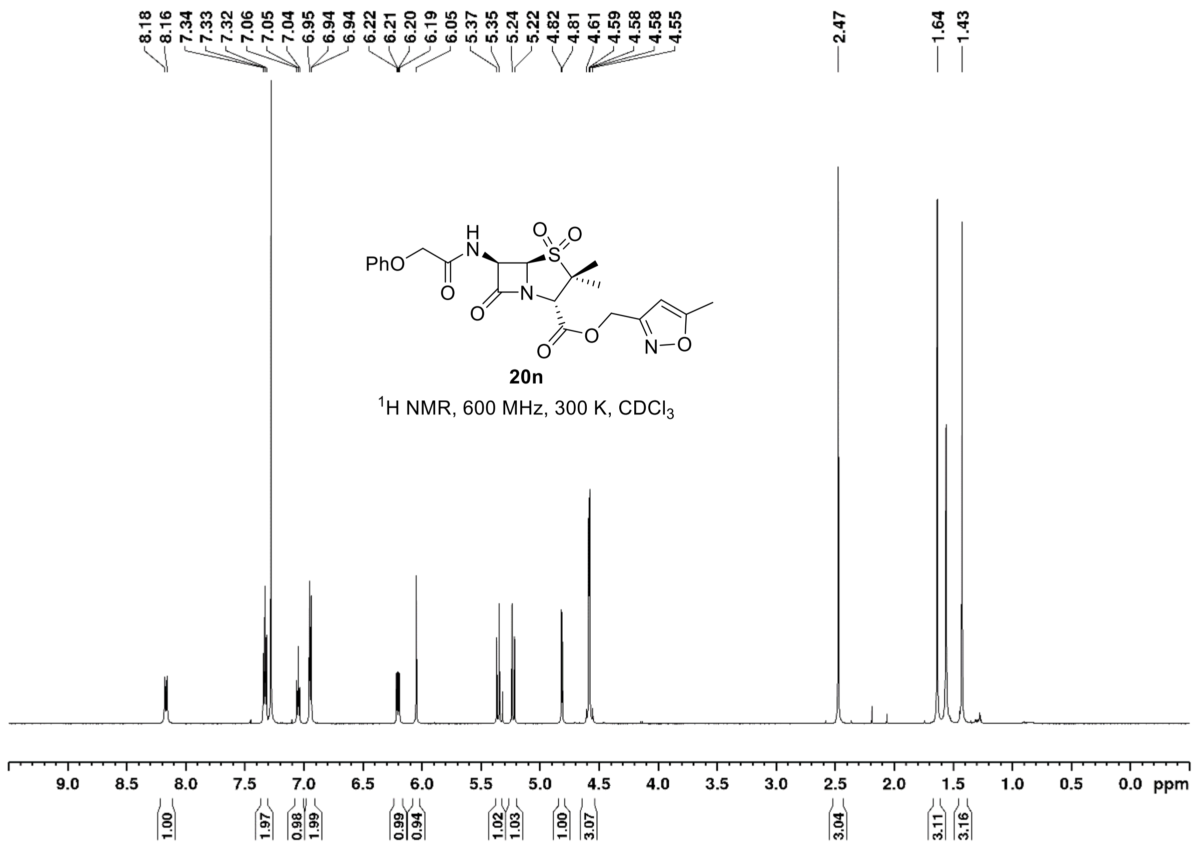

8.18
8.16
7.34
7.33
7.32
7.06
7.05
7.04
6.95
6.94
6.94
6.22
6.21
6.20
6.19
6.05
5.37
5.35
5.24
5.22
4.82
4.81
4.61
4.59
4.58
4.58
4.55
2.47
1.64
1.43
PhO
H
N
O
S
N
O
O
O
O
O
N–O
20n
^{1}H NMR, 600 MHz, 300 K, $CDCl_3$
9.0
8.5
8.0
7.5
7.0
6.5
6.0
5.5
5.0
4.5
4.0
3.5
3.0
2.5
2.0
1.5
1.0
0.5
0.0
ppm
1.00
1.97
0.98
1.99
0.99
0.94
1.02
1.03
1.00
3.07
3.04
3.11
3.16

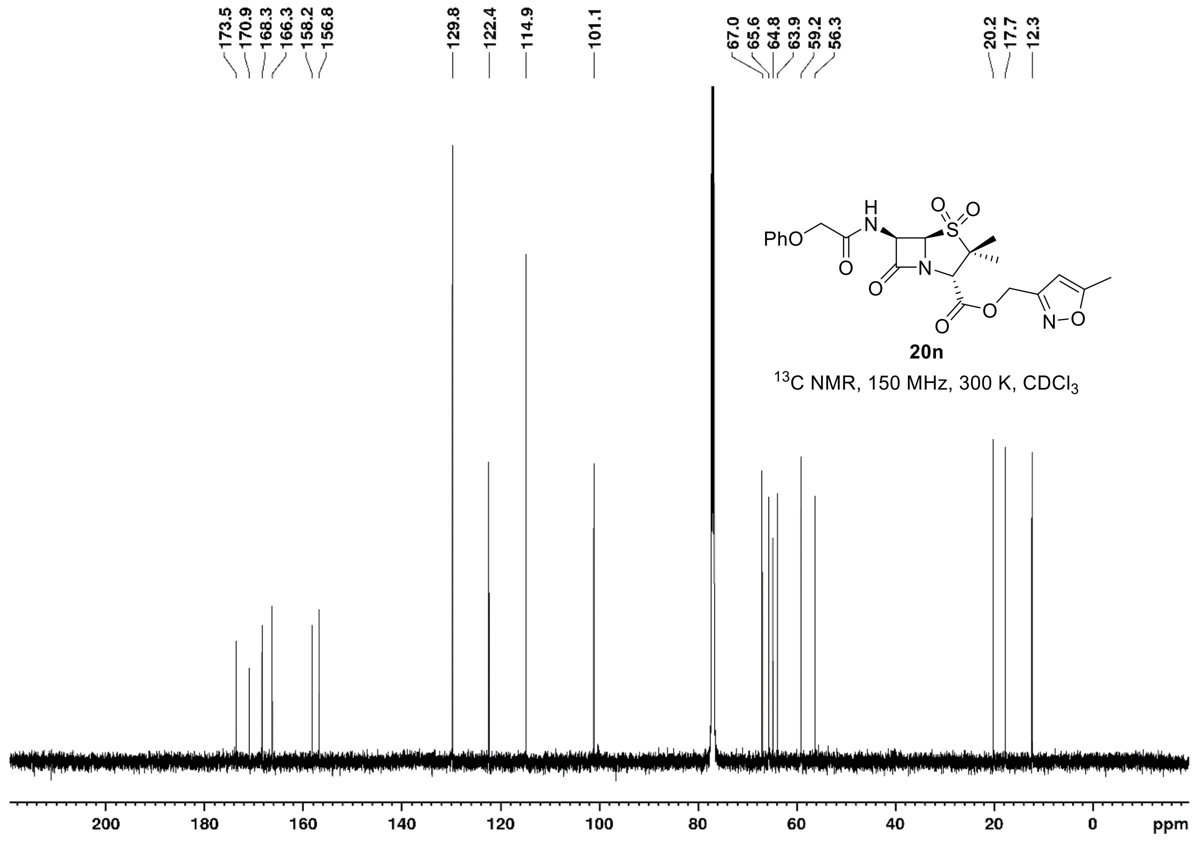

173.5
170.9
168.3
166.3
158.2
156.8
129.8
122.4
114.9
101.1
67.0
65.6
64.8
63.9
59.2
56.3
20.2
17.7
12.3
PhO
H
N
O
S
N
O
O
O
O
O
N–O
20n
^{13}C NMR, 150 MHz, 300 K, $CDCl_3$
200
180
160
140
120
100
80
60
40
20
0
ppm

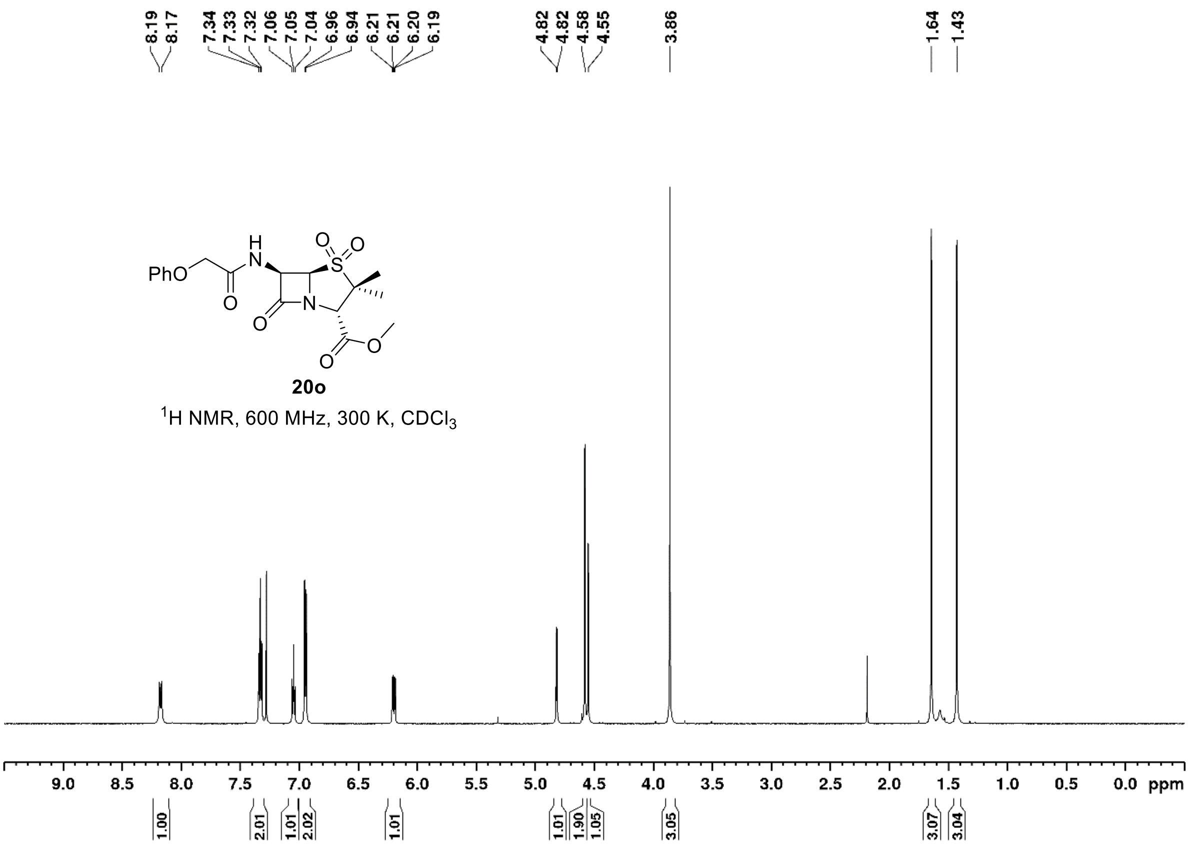
8.19
8.17
7.34
7.33
7.32
7.06
7.05
7.04
6.96
6.94
6.21
6.21
6.20
6.19
4.82
4.82
4.58
4.55
3.86
1.64
1.43
PhO
H
N
O
O
S
O
O
N
O
O
20o
^{1}H NMR, 600 MHz, 300 K, $CDCl_3$
9.0
8.5
8.0
7.5
7.0
6.5
6.0
5.5
5.0
4.5
4.0
3.5
3.0
2.5
2.0
1.5
1.0
0.5
0.0
ppm
1.00
2.01
1.01
2.02
1.01
1.01
1.90
1.05
3.05
3.07
3.04

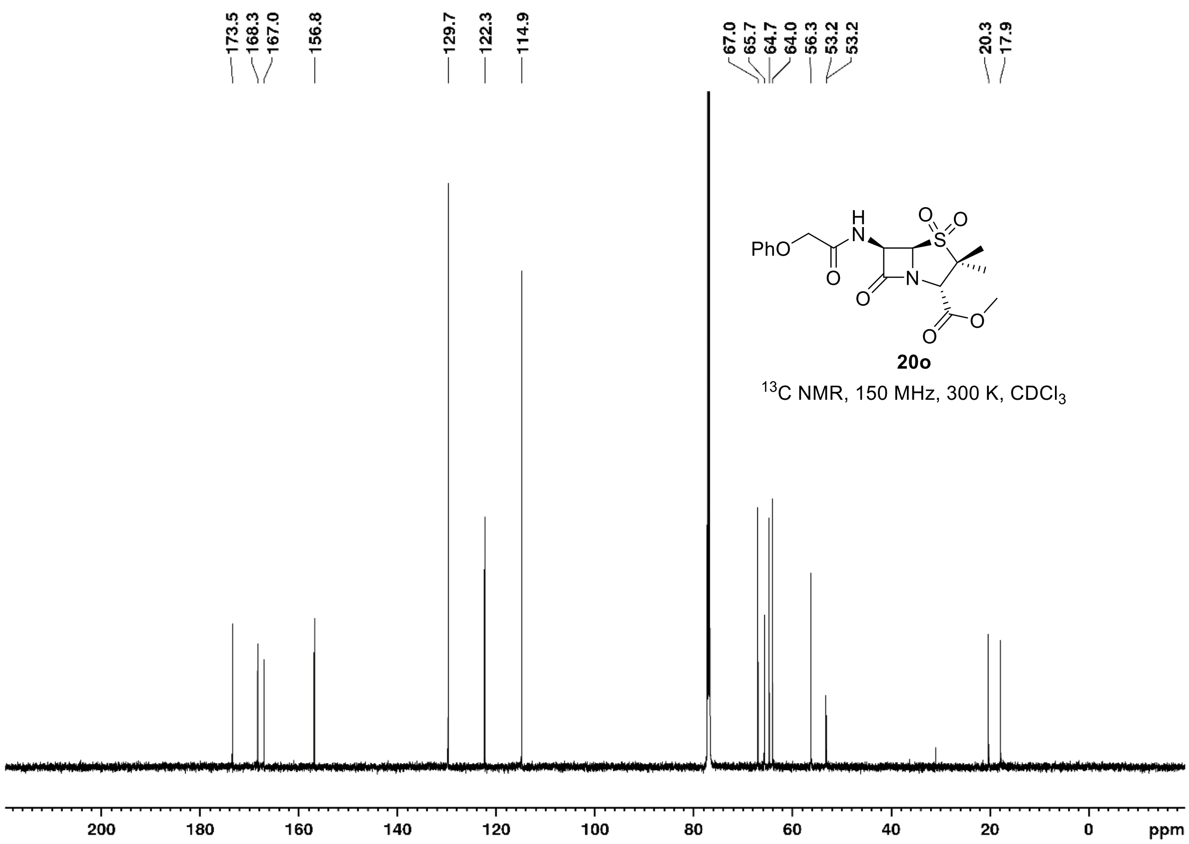
173.5
168.3
167.0
156.8
129.7
122.3
114.9
67.0
65.7
64.7
64.0
56.3
53.2
53.2
20.3
17.9
PhO
H
N
O
O
S
O
O
N
O
O
20o
^{13}C NMR, 150 MHz, 300 K, $CDCl_3$
200
180
160
140
120
100
80
60
40
20
0
ppm

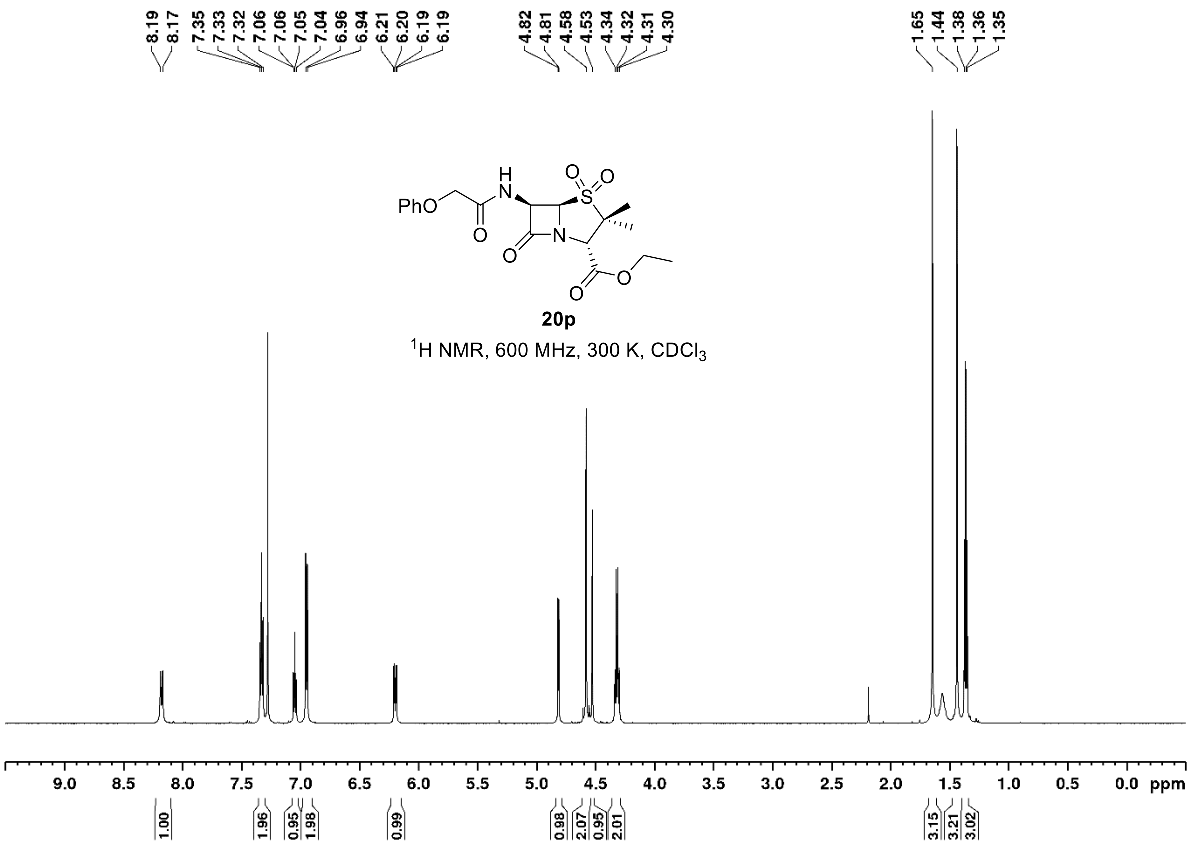

20p

^{1}H NMR, 600 MHz, 300 K, $CDCl_3$

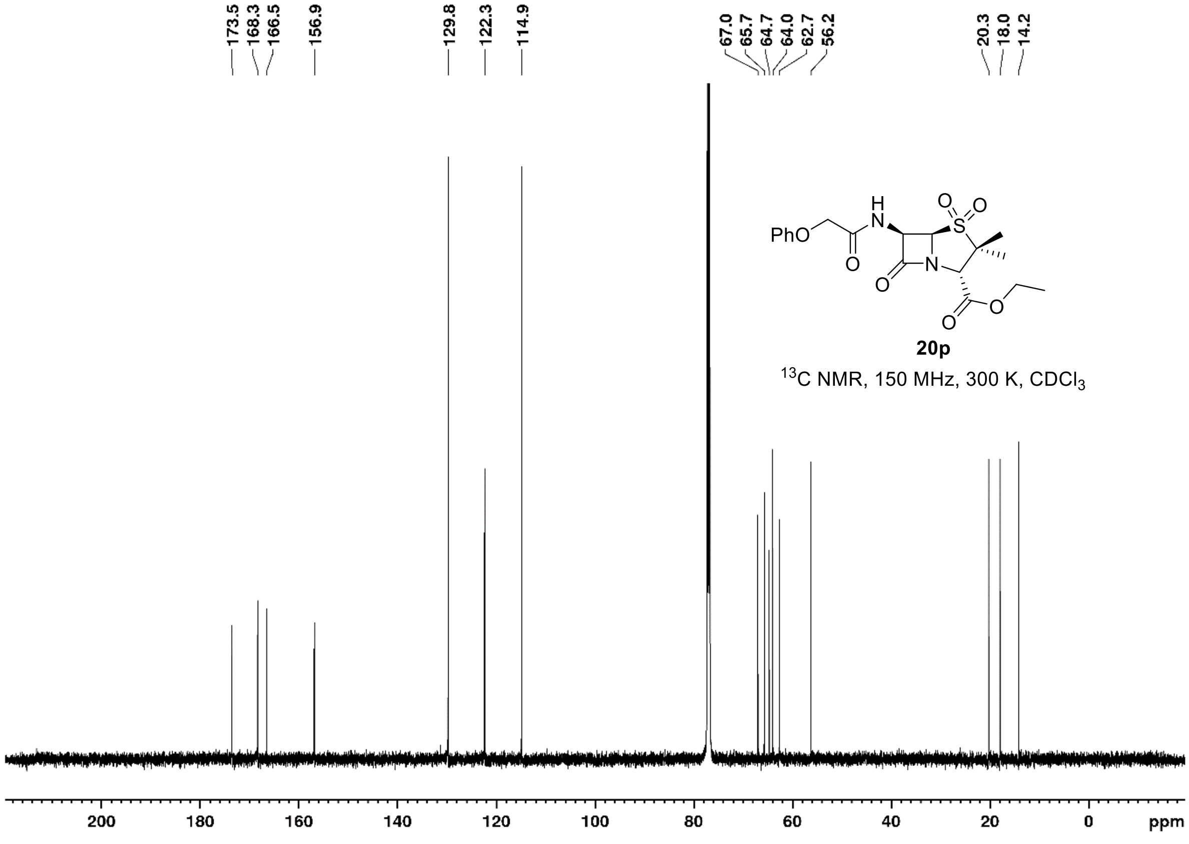

20p

^{13}C NMR, 150 MHz, 300 K, $CDCl_3$

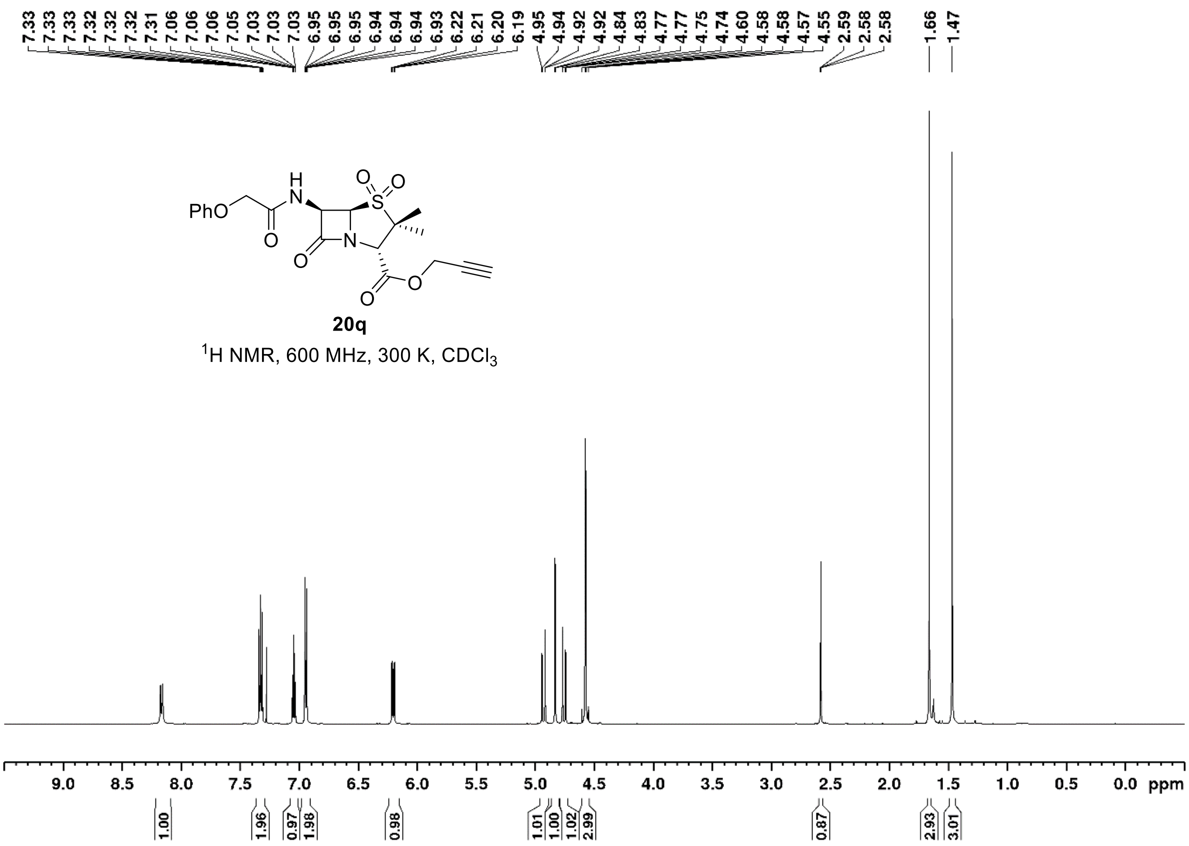
7.33 7.33 7.33 7.32 7.32 7.32 7.31 7.06 7.06 7.06 7.05 7.03 7.03 7.03 6.95 6.95 6.95 6.94 6.94 6.94 6.93 6.22 6.21 6.20 6.19 4.95 4.94 4.92 4.92 4.84 4.83 4.77 4.77 4.75 4.74 4.60 4.58 4.58 4.57 4.55 2.59 2.58 2.58 1.66 1.47
PhO
H
N
O
S
O
N
O
O
O
O
20q
^{1}H NMR, 600 MHz, 300 K, $CDCl_3$
9.0 8.5 8.0 7.5 7.0 6.5 6.0 5.5 5.0 4.5 4.0 3.5 3.0 2.5 2.0 1.5 1.0 0.5 0.0 ppm
1.00 1.96 0.97 1.98 0.98 1.01 1.00 1.02 2.99 0.87 2.93 3.01

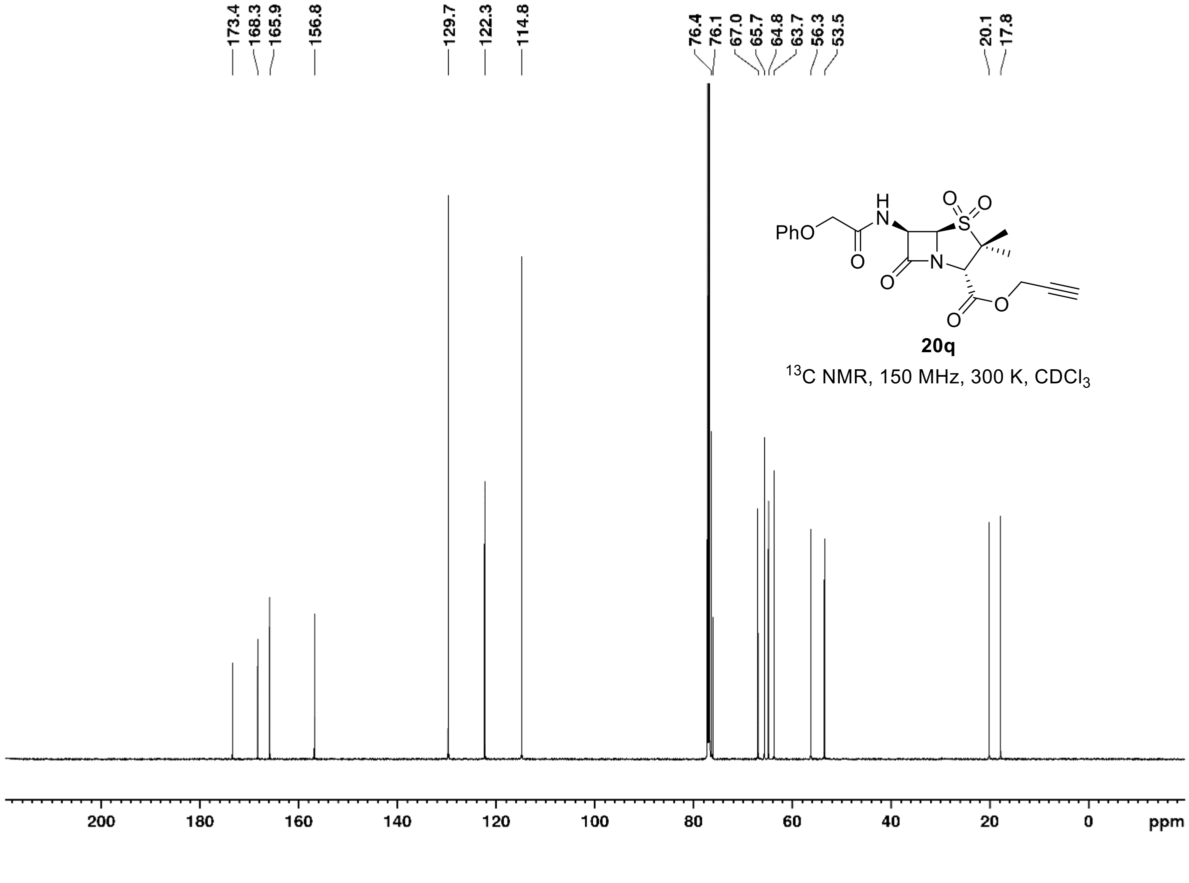
173.4 168.3 165.9 156.8 129.7 122.3 114.8 76.4 76.1 67.0 65.7 64.8 63.7 56.3 53.5 20.1 17.8
PhO
H
N
O
S
O
N
O
O
O
O
20q
^{13}C NMR, 150 MHz, 300 K, $CDCl_3$
200 180 160 140 120 100 80 60 40 20 0 ppm

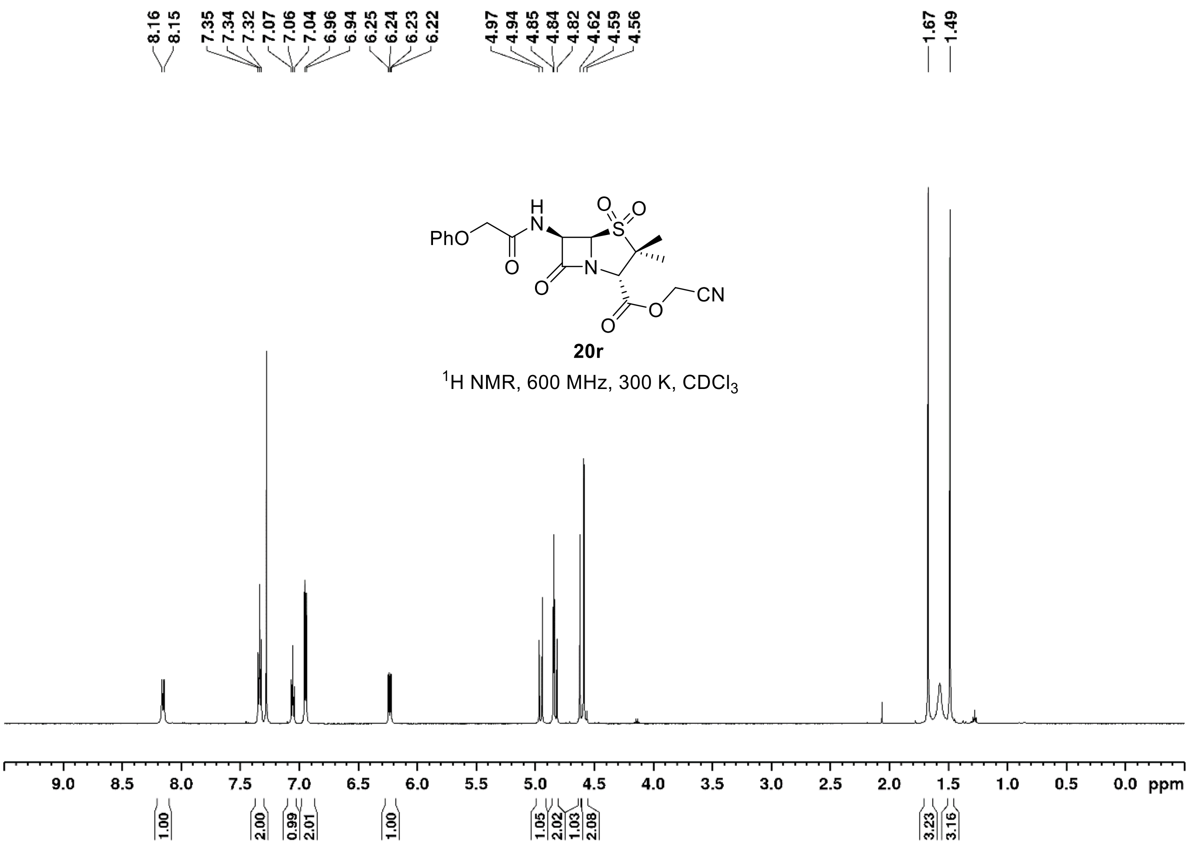
8.16
8.15
7.35
7.34
7.32
7.07
7.06
7.04
6.96
6.94
6.25
6.24
6.23
6.22
4.97
4.94
4.85
4.84
4.82
4.62
4.59
4.56
1.67
1.49
PhO
H
N
O
O
S
O
N
O
O
O
CN
20r
^{1}H NMR, 600 MHz, 300 K, $CDCl_3$
9.0 8.5 8.0 7.5 7.0 6.5 6.0 5.5 5.0 4.5 4.0 3.5 3.0 2.5 2.0 1.5 1.0 0.5 0.0 ppm
1.00
2.00
0.99
2.01
1.00
1.05
2.02
1.03
2.08
3.23
3.16

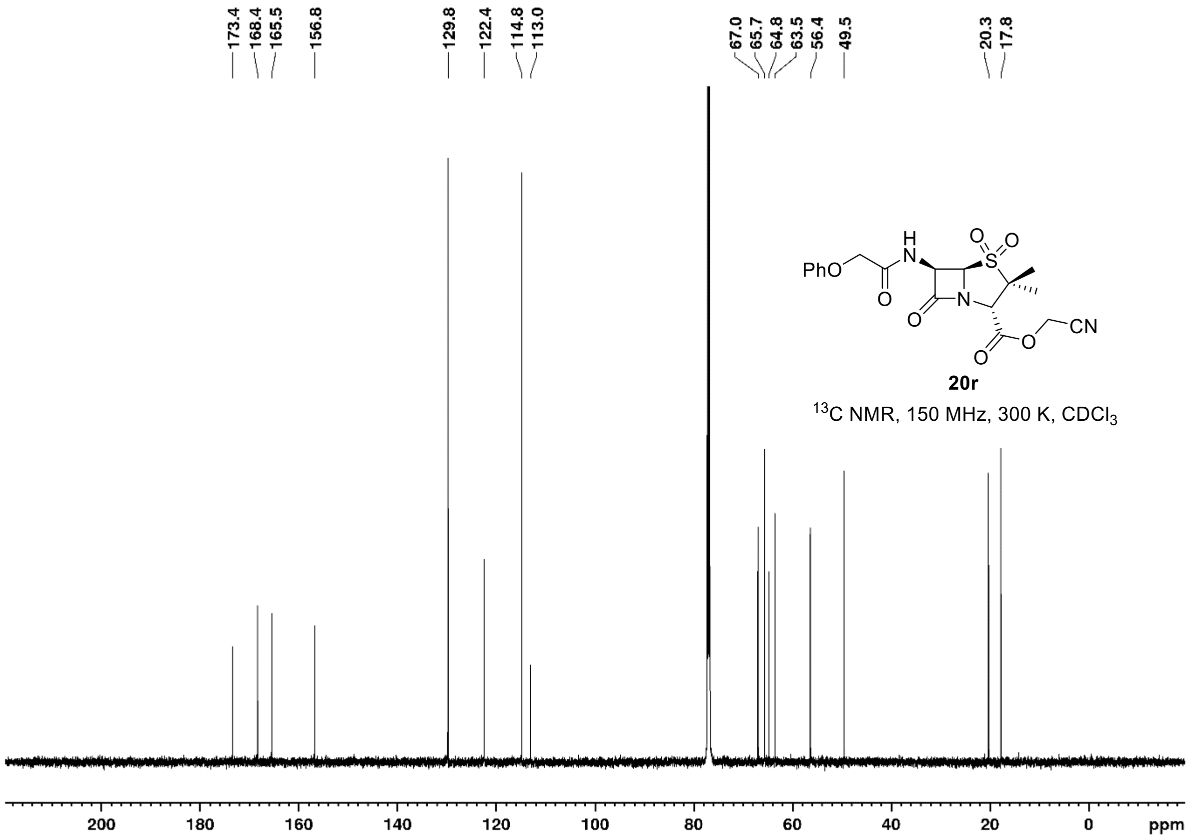
173.4
168.4
165.5
156.8
129.8
122.4
114.8
113.0
67.0
65.7
64.8
63.5
56.4
49.5
20.3
17.8
PhO
H
N
O
O
S
O
N
O
O
O
CN
20r
^{13}C NMR, 150 MHz, 300 K, $CDCl_3$
200 180 160 140 120 100 80 60 40 20 0 ppm

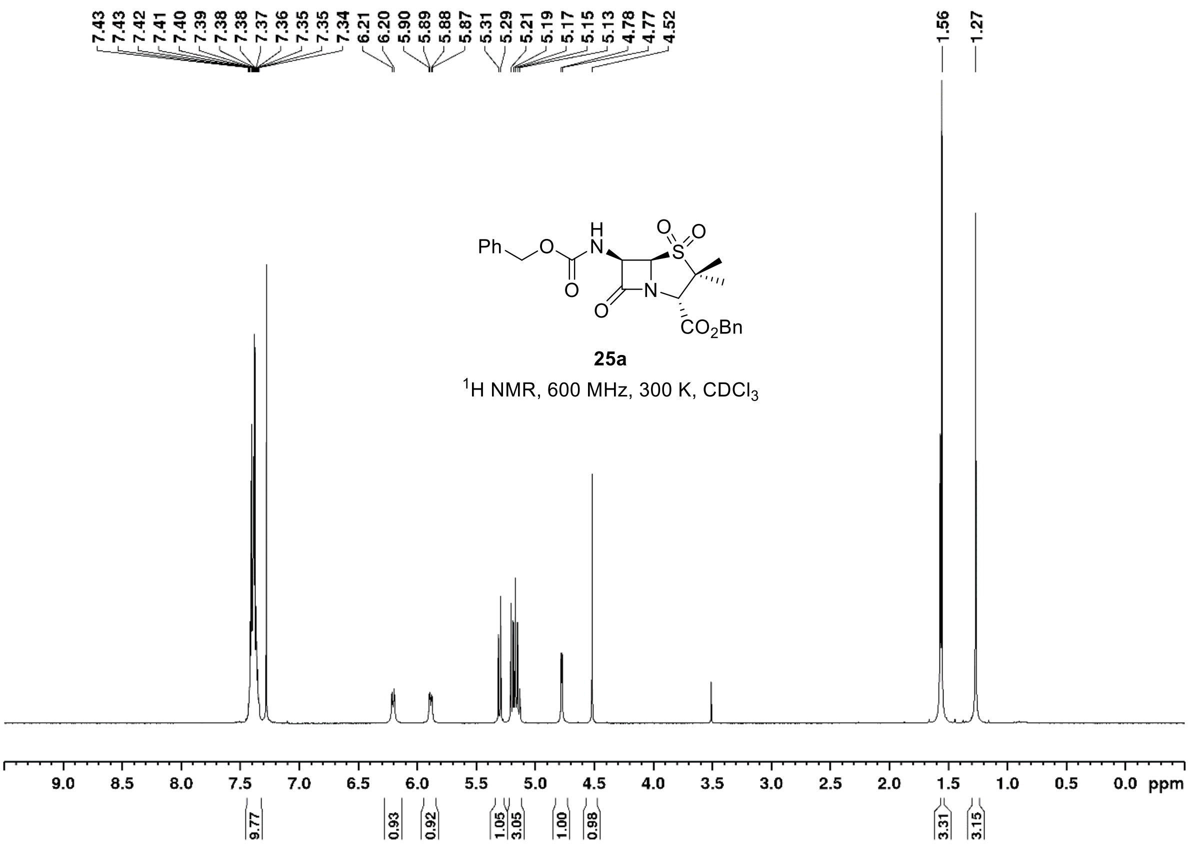
7.43 7.43 7.42 7.41 7.40 7.39 7.38 7.38 7.37 7.36 7.35 7.35 7.34 6.21 6.20 5.90 5.89 5.88 5.87 5.31 5.29 5.21 5.19 5.17 5.15 5.13 4.78 4.77 4.52
1.56 1.27
Ph O N H O O S O O N CO2Bn
25a
1H NMR, 600 MHz, 300 K, CDCl3
9.0 8.5 8.0 7.5 7.0 6.5 6.0 5.5 5.0 4.5 4.0 3.5 3.0 2.5 2.0 1.5 1.0 0.5 0.0 ppm
9.77 0.93 0.92 1.05 3.05 1.00 0.98 3.31 3.15

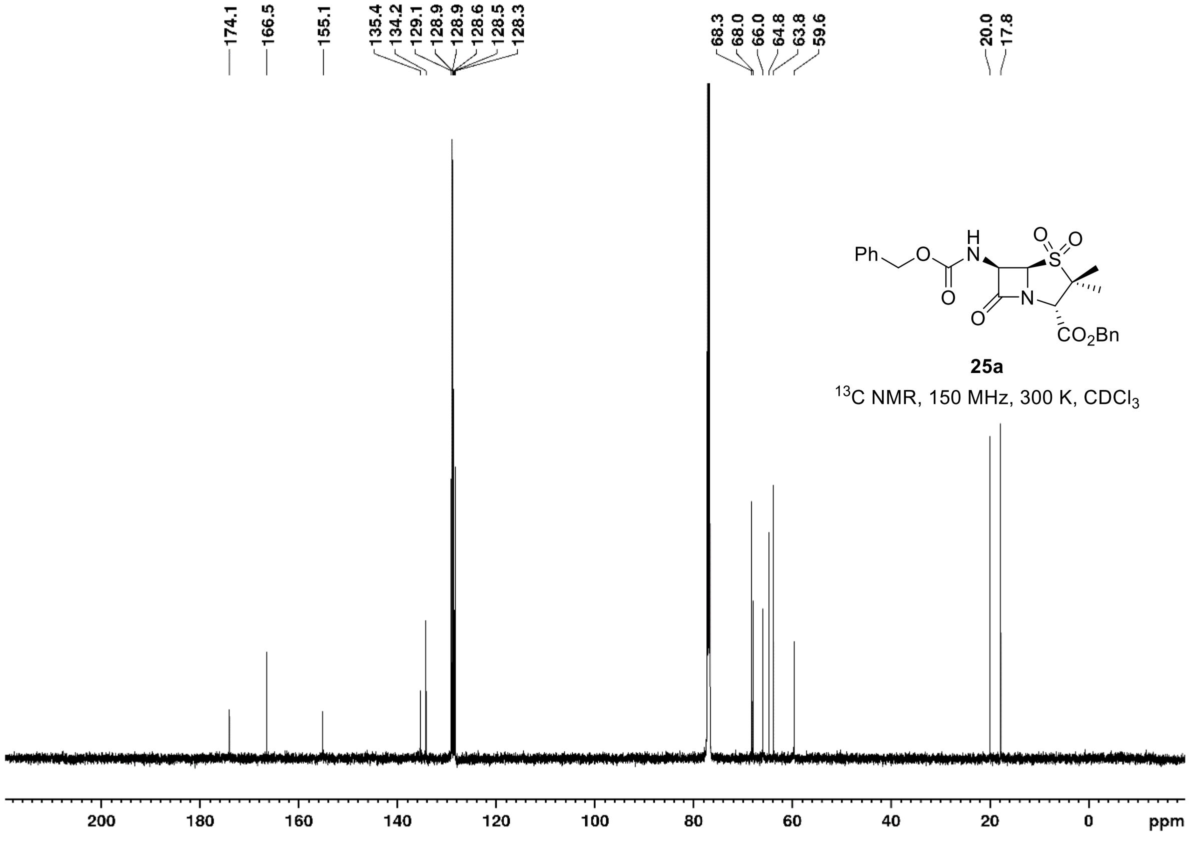
174.1 166.5 155.1 135.4 134.2 129.1 128.9 128.9 128.6 128.5 128.3 68.3 68.0 66.0 64.8 63.8 59.6 20.0 17.8
Ph O N H O O S O O N CO2Bn
25a
13C NMR, 150 MHz, 300 K, CDCl3
200 180 160 140 120 100 80 60 40 20 0 ppm

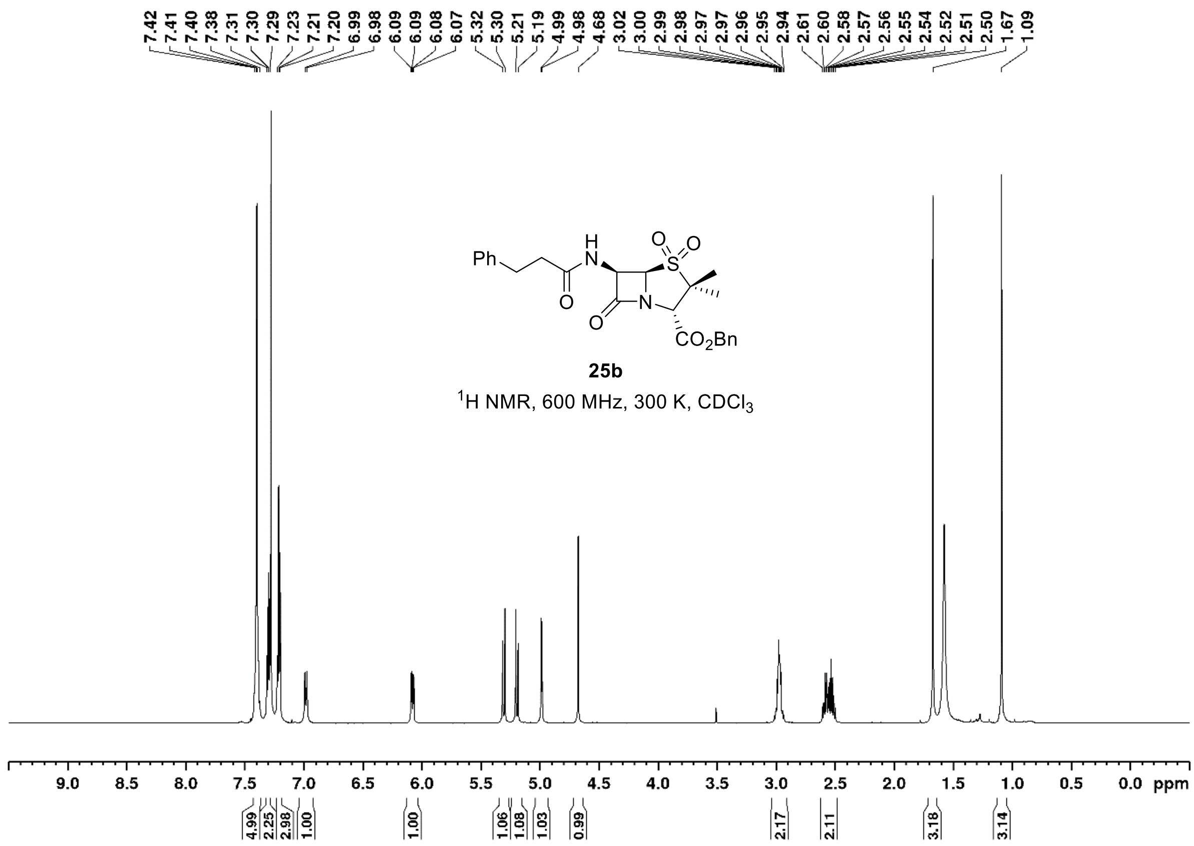
7.42 7.41 7.40 7.38 7.31 7.30 7.29 7.23 7.21 7.20 6.99 6.98 6.09 6.09 6.08 6.07 5.32 5.30 5.21 5.19 4.99 4.98 4.68 3.02 3.00 2.99 2.98 2.97 2.97 2.96 2.95 2.94 2.61 2.60 2.58 2.57 2.56 2.55 2.54 2.52 2.51 2.50 1.67 1.09
Ph
H
N
O
S
O O
N
O
CO2Bn
25b
1H NMR, 600 MHz, 300 K, CDCl3
9.0 8.5 8.0 7.5 7.0 6.5 6.0 5.5 5.0 4.5 4.0 3.5 3.0 2.5 2.0 1.5 1.0 0.5 0.0 ppm
4.99 2.25 2.98 1.00 1.00 1.06 1.08 1.03 0.99 2.17 2.11 3.18 3.14

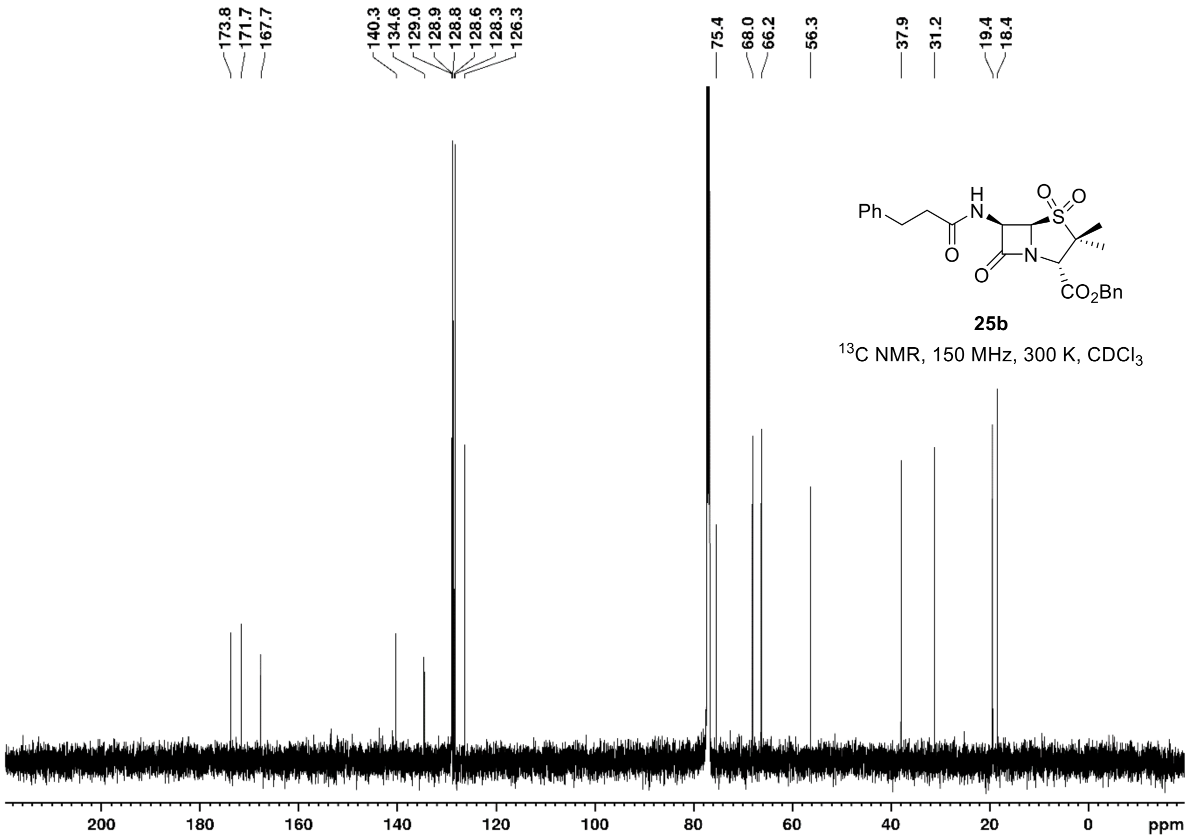
173.8 171.7 167.7 140.3 134.6 129.0 128.9 128.8 128.6 128.3 126.3 75.4 68.0 66.2 56.3 37.9 31.2 19.4 18.4
Ph
H
N
O
S
O O
N
O
CO2Bn
25b
13C NMR, 150 MHz, 300 K, CDCl3
200 180 160 140 120 100 80 60 40 20 0 ppm

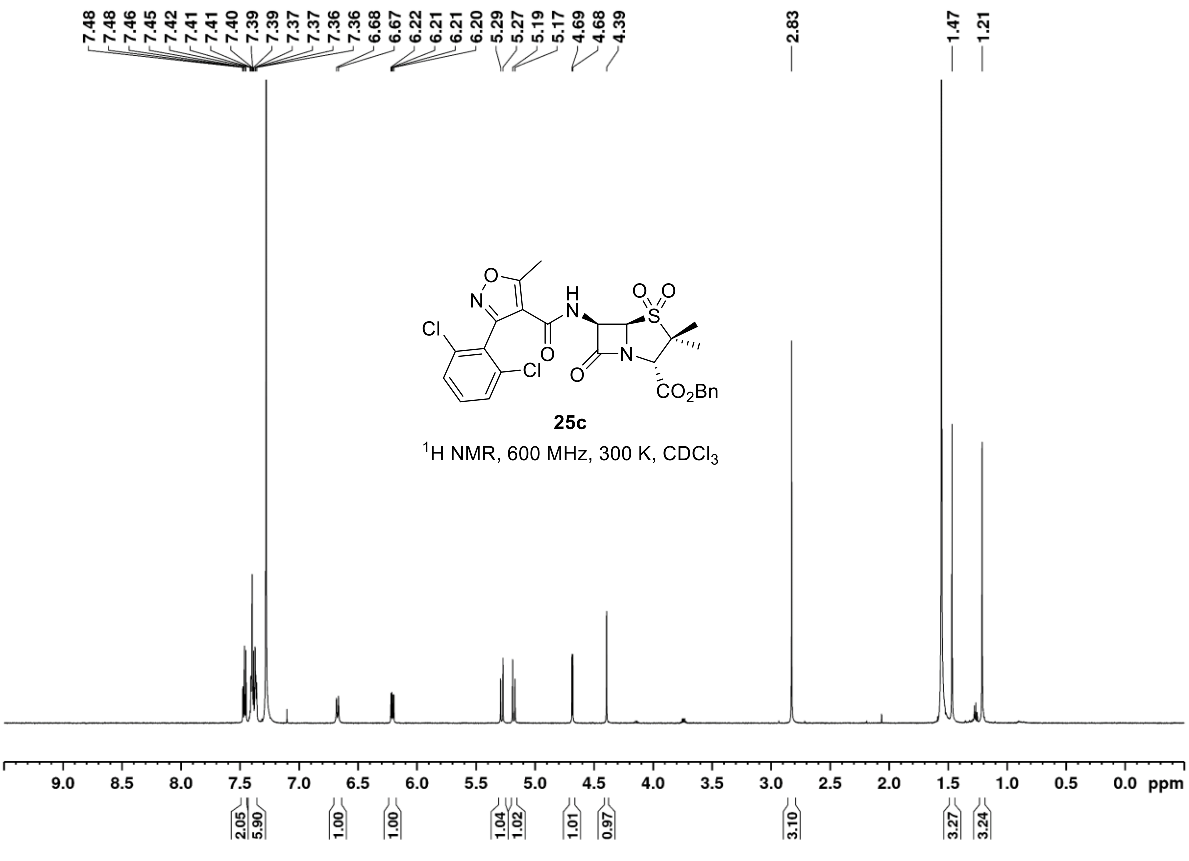
7.48
7.48
7.46
7.45
7.42
7.41
7.41
7.40
7.39
7.39
7.37
7.37
7.36
7.36
6.68
6.67
6.22
6.21
6.21
6.20
5.29
5.27
5.19
5.17
4.69
4.68
4.39
2.83
1.47
1.21
25c
^{1}H NMR, 600 MHz, 300 K, $CDCl_3$
9.0 8.5 8.0 7.5 7.0 6.5 6.0 5.5 5.0 4.5 4.0 3.5 3.0 2.5 2.0 1.5 1.0 0.5 0.0 ppm
2.05
5.90
1.00
1.00
1.04
1.02
1.01
0.97
3.10
3.27
3.24

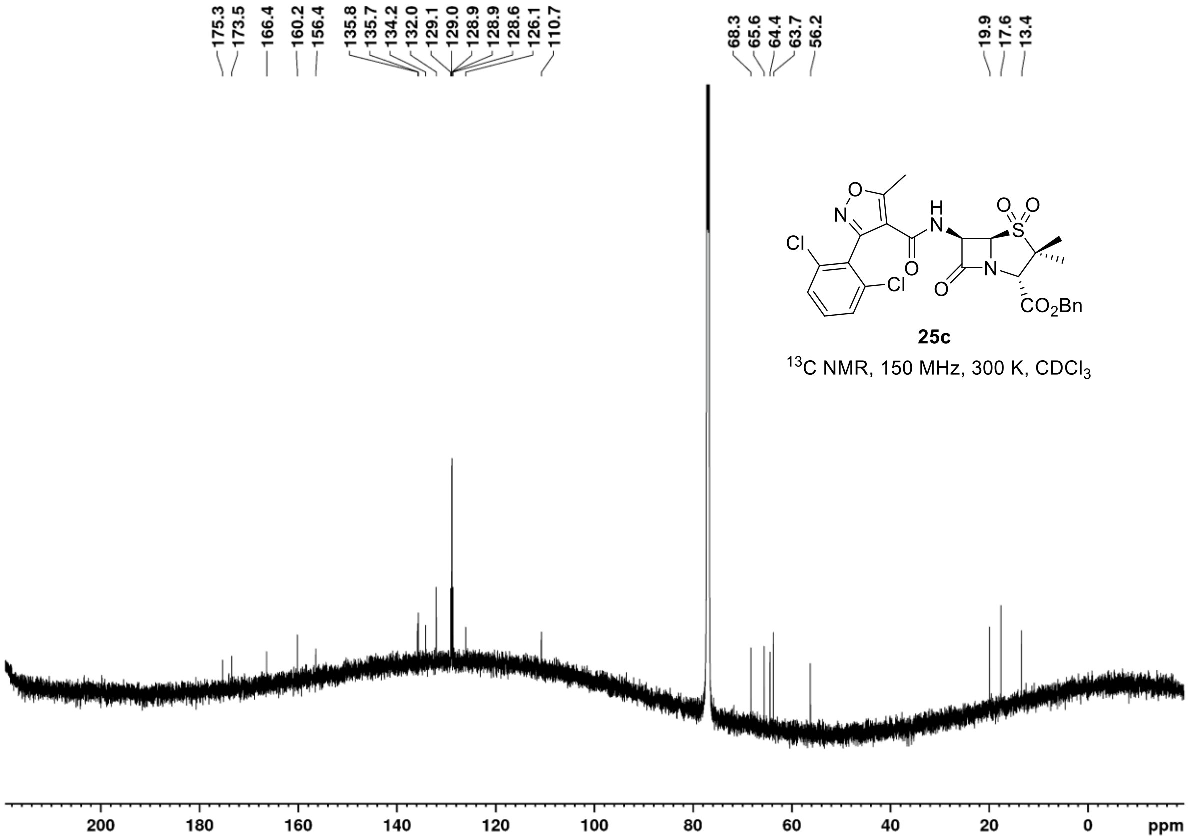
175.3
173.5
166.4
160.2
156.4
135.8
135.7
134.2
132.0
129.1
129.0
128.9
128.9
128.6
126.1
110.7
68.3
65.6
64.4
63.7
56.2
19.9
17.6
13.4
25c
^{13}C NMR, 150 MHz, 300 K, $CDCl_3$
200 180 160 140 120 100 80 60 40 20 0 ppm

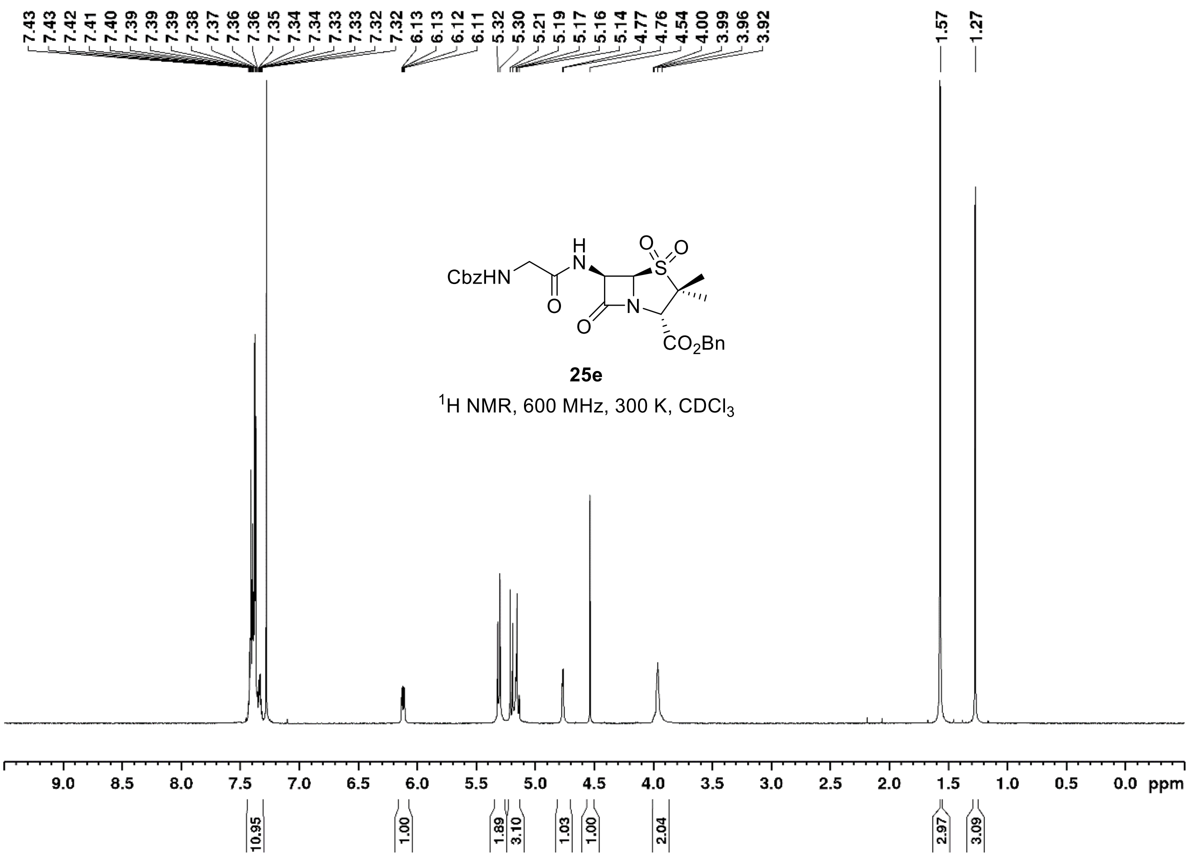
CbzHN
H
N
O
S
O
O
N
O
CO2Bn
25e
1H NMR, 600 MHz, 300 K, CDCl3
ppm

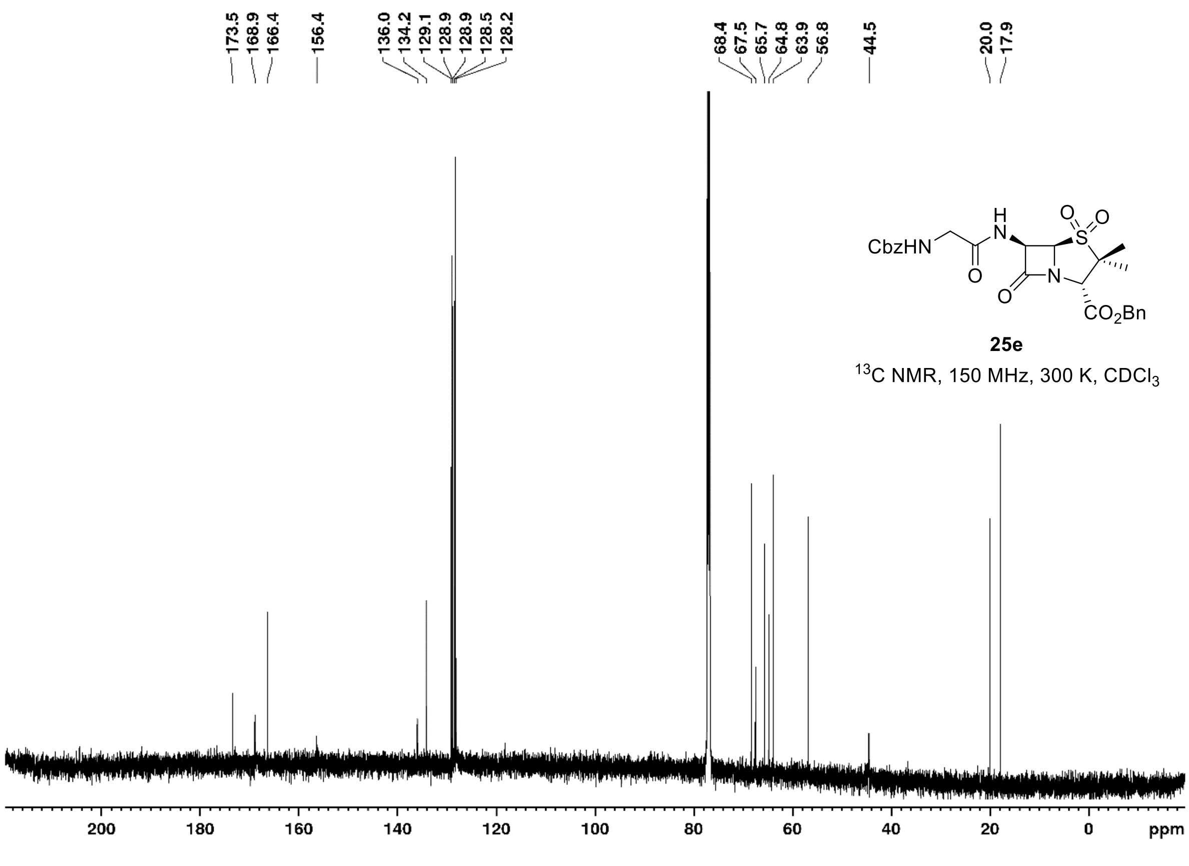
CbzHN
H
N
O
S
O
O
N
O
CO2Bn
25e
13C NMR, 150 MHz, 300 K, CDCl3
ppm

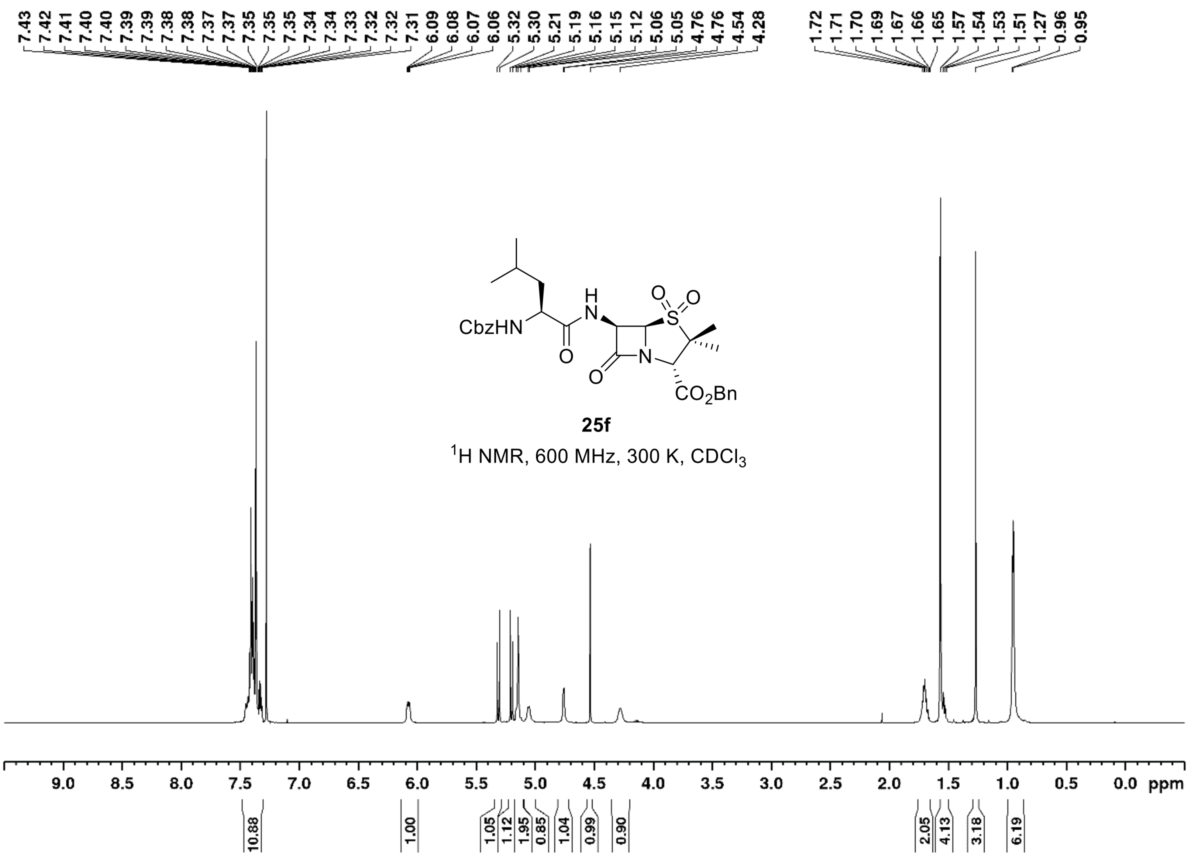
7.43 7.42 7.41 7.40 7.40 7.39 7.39 7.38 7.38 7.37 7.35 7.35 7.35 7.34 7.34 7.33 7.32 7.32 7.31 6.09 6.08 6.07 6.06 5.32 5.30 5.21 5.19 5.16 5.15 5.12 5.06 5.05 4.76 4.76 4.54 4.28
1.72 1.71 1.70 1.69 1.67 1.66 1.65 1.57 1.54 1.53 1.51 1.27 0.96 0.95
CbzHN
H
N
O
S
O
O
N
O
CO2Bn
25f
1H NMR, 600 MHz, 300 K, CDCl3
9.0 8.5 8.0 7.5 7.0 6.5 6.0 5.5 5.0 4.5 4.0 3.5 3.0 2.5 2.0 1.5 1.0 0.5 0.0 ppm
10.88 1.00 1.05 1.12 1.95 0.85 1.04 0.99 0.90 2.05 4.13 3.18 6.19

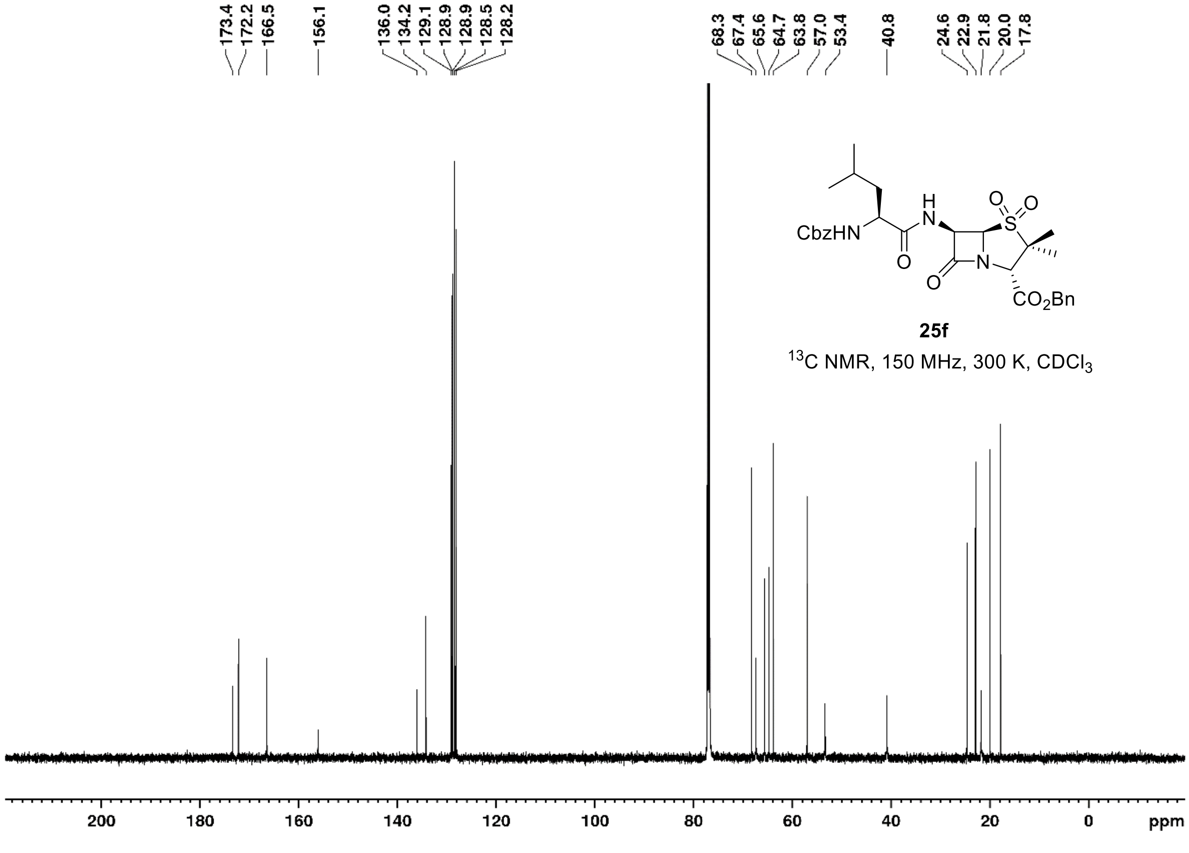
173.4 172.2 166.5 156.1 136.0 134.2 129.1 128.9 128.9 128.5 128.2 68.3 67.4 65.6 64.7 63.8 57.0 53.4 40.8 24.6 22.9 21.8 20.0 17.8
CbzHN
H
N
O
S
O
O
N
O
CO2Bn
25f
13C NMR, 150 MHz, 300 K, CDCl3
200 180 160 140 120 100 80 60 40 20 0 ppm

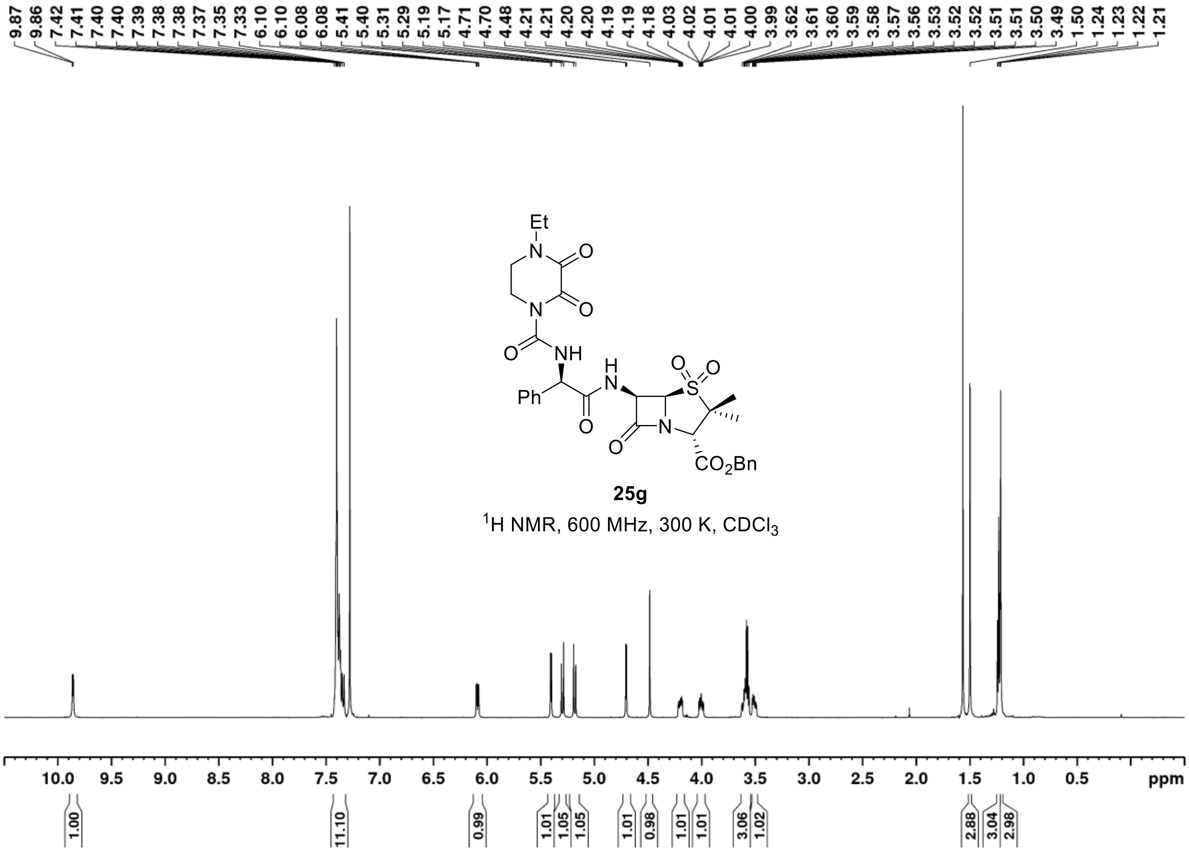
9.87 9.86 7.42 7.41 7.40 7.40 7.39 7.38 7.38 7.37 7.35 7.33 6.10 6.10 6.08 6.08 5.41 5.40 5.31 5.29 5.19 5.17 4.71 4.70 4.48 4.21 4.21 4.20 4.20 4.19 4.19 4.18 4.03 4.02 4.01 4.01 4.00 3.99 3.62 3.61 3.60 3.59 3.58 3.57 3.56 3.53 3.52 3.52 3.51 3.51 3.50 3.49 1.50 1.24 1.23 1.22 1.21
Et
N
O
N
O
O
NH
H
N
O
S
O
Ph
O
N
O
CO2Bn
25g
1H NMR, 600 MHz, 300 K, CDCl3
10.0 9.5 9.0 8.5 8.0 7.5 7.0 6.5 6.0 5.5 5.0 4.5 4.0 3.5 3.0 2.5 2.0 1.5 1.0 0.5 ppm
1.00 11.10 0.99 1.01 1.05 1.05 1.01 0.98 1.01 1.01 3.06 1.02 2.88 3.04 2.98

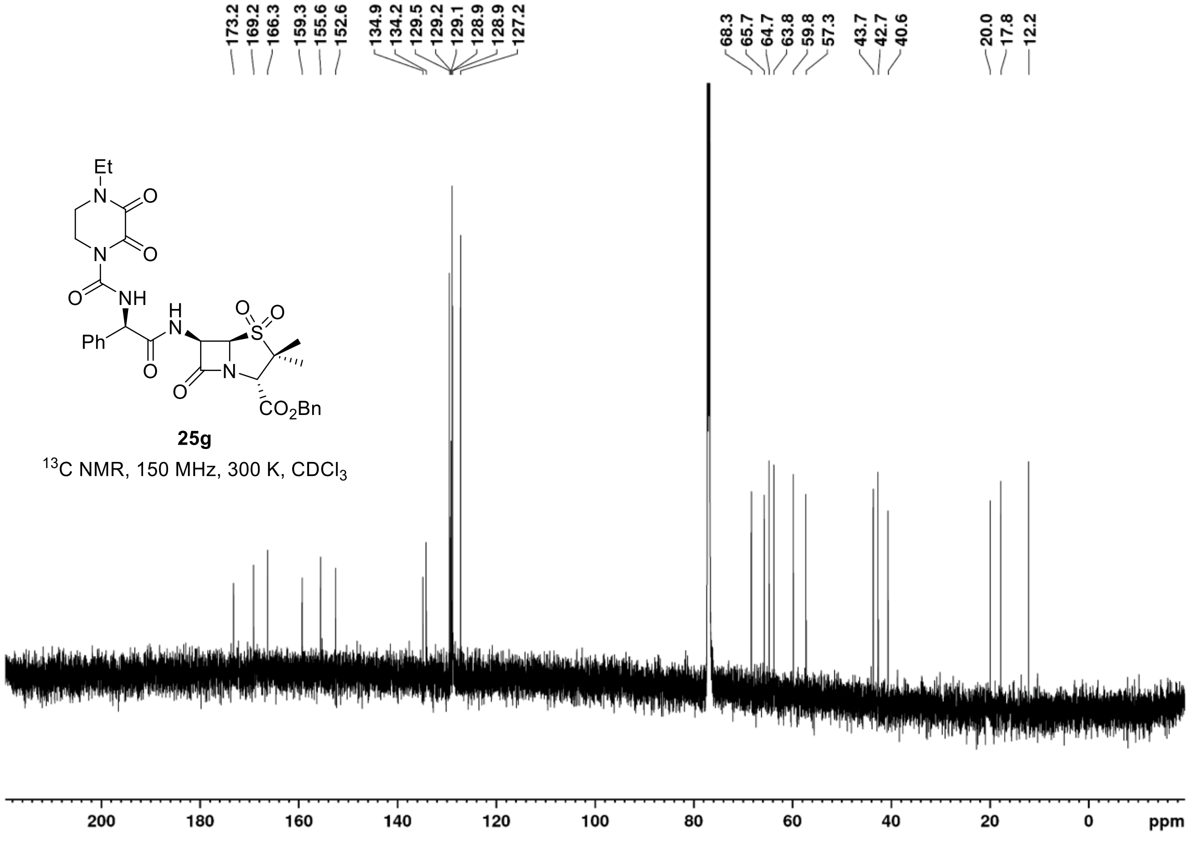
173.2 169.2 166.3 159.3 155.6 152.6 134.9 134.2 129.5 129.2 129.1 128.9 128.9 127.2 68.3 65.7 64.7 63.8 59.8 57.3 43.7 42.7 40.6 20.0 17.8 12.2
Et
N
O
N
O
O
NH
H
N
O
S
O
Ph
O
N
O
CO2Bn
25g
13C NMR, 150 MHz, 300 K, CDCl3
200 180 160 140 120 100 80 60 40 20 0 ppm

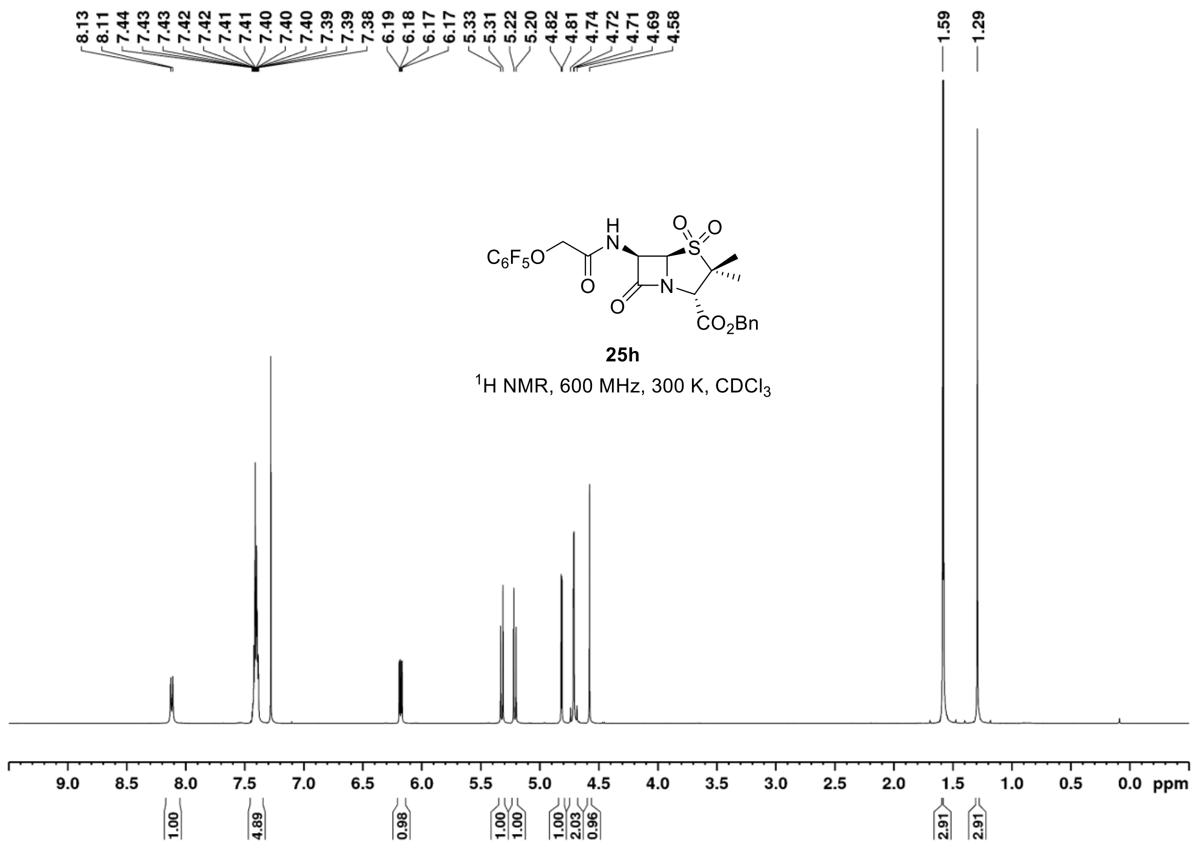
8.13 8.11 7.44 7.43 7.43 7.42 7.42 7.41 7.41 7.40 7.40 7.40 7.39 7.39 7.38 6.19 6.18 6.17 6.17 5.33 5.31 5.22 5.20 4.82 4.81 4.74 4.72 4.71 4.69 4.58
1.59
1.29
C6F5O
N
H
S
O
O
N
O
O
CO2Bn
25h
1H NMR, 600 MHz, 300 K, CDCl3
9.0 8.5 8.0 7.5 7.0 6.5 6.0 5.5 5.0 4.5 4.0 3.5 3.0 2.5 2.0 1.5 1.0 0.5 0.0 ppm
1.00 4.89 0.98 1.00 1.00 1.00 2.03 0.96 2.91 2.91

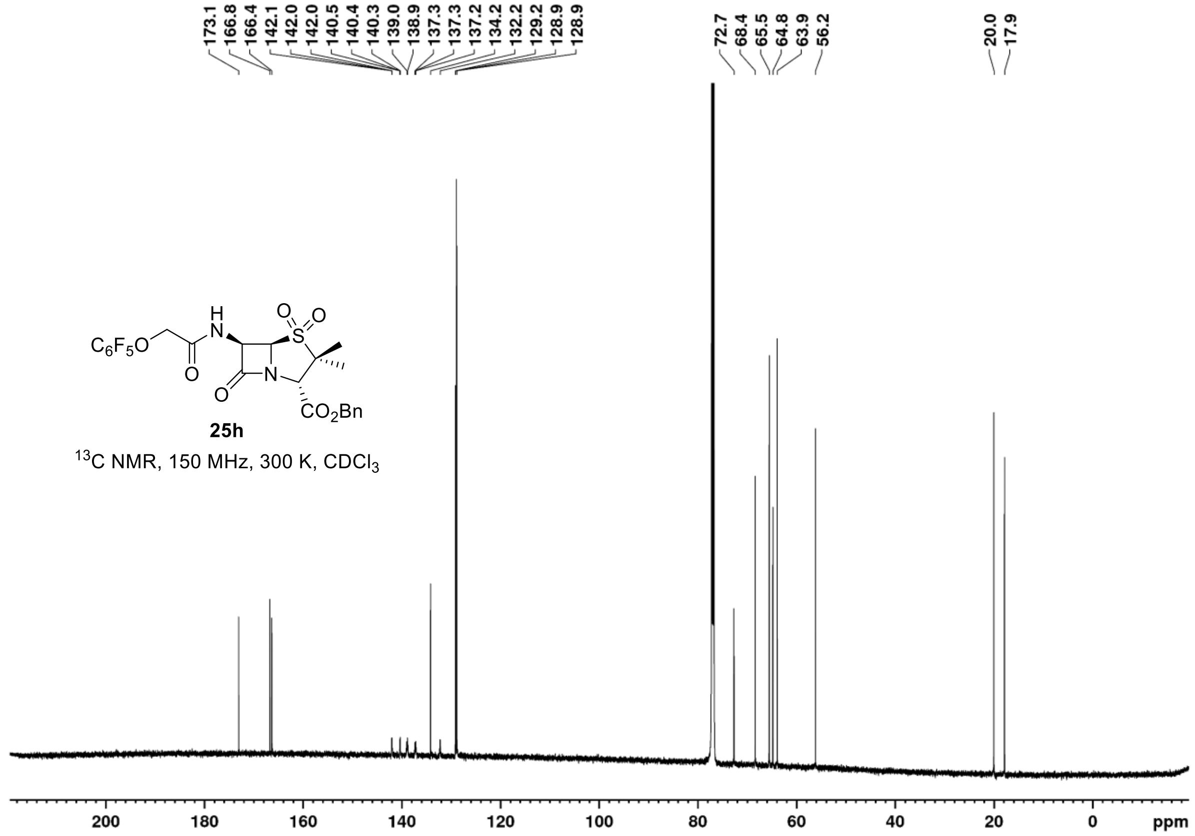
173.1 166.8 166.4 142.1 142.0 142.0 140.5 140.4 140.3 139.0 138.9 137.3 137.3 137.2 134.2 132.2 129.2 128.9 128.9
72.7 68.4 65.5 64.8 63.9 56.2
20.0 17.9
C6F5O
N
H
S
O
O
N
O
O
CO2Bn
25h
13C NMR, 150 MHz, 300 K, CDCl3
200 180 160 140 120 100 80 60 40 20 0 ppm

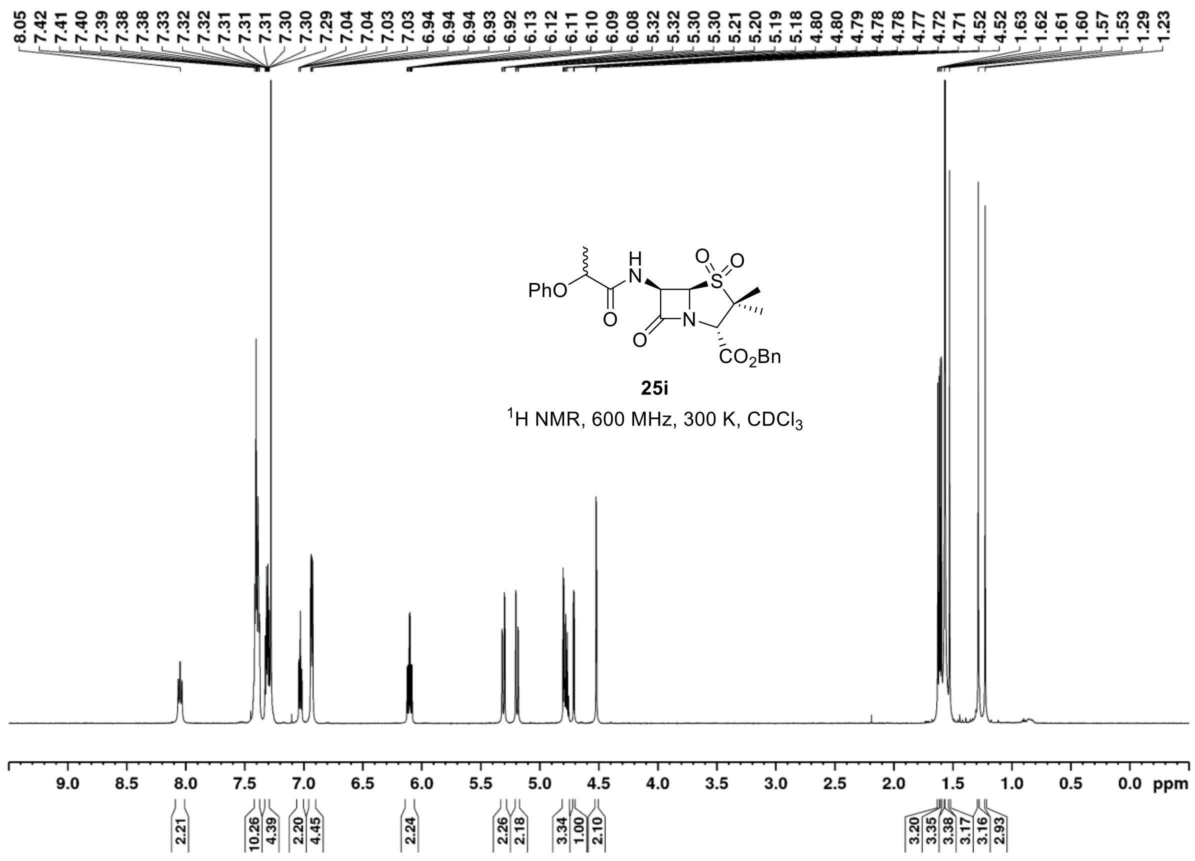
8.05 7.42 7.41 7.40 7.39 7.38 7.38 7.33 7.32 7.32 7.31 7.31 7.31 7.30 7.30 7.29 7.04 7.04 7.03 7.03 6.94 6.94 6.94 6.93 6.92 6.13 6.12 6.11 6.10 6.09 6.08 5.32 5.32 5.30 5.30 5.21 5.20 5.19 5.18 4.80 4.79 4.78 4.78 4.77 4.72 4.71 4.52 4.52 1.63 1.62 1.61 1.60 1.57 1.53 1.29 1.23
H
O
S
O
PhO
O
N
O
CO2Bn
25i
1H NMR, 600 MHz, 300 K, CDCl3
9.0 8.5 8.0 7.5 7.0 6.5 6.0 5.5 5.0 4.5 4.0 3.5 3.0 2.5 2.0 1.5 1.0 0.5 0.0 ppm
2.21 10.26 4.39 2.20 4.45 2.24 2.26 2.18 3.34 1.00 2.10 3.20 3.35 3.38 3.17 3.16 2.93

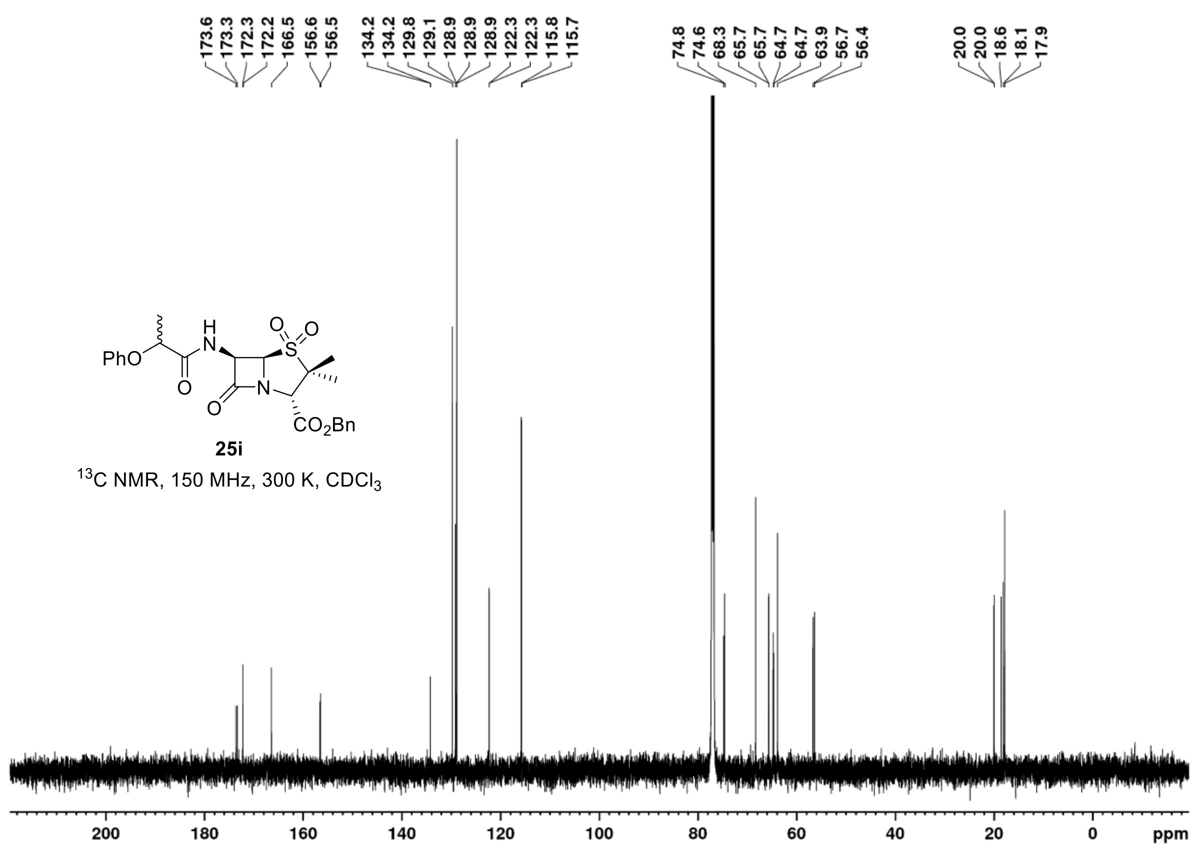
173.6 173.3 172.3 172.2 166.5 156.6 156.5 134.2 134.2 129.8 129.1 128.9 128.9 128.9 122.3 122.3 115.8 115.7 74.8 74.6 68.3 65.7 65.7 64.7 64.7 63.9 56.7 56.4 20.0 20.0 18.6 18.1 17.9
H
O
S
O
PhO
O
N
O
CO2Bn
25i
13C NMR, 150 MHz, 300 K, CDCl3
200 180 160 140 120 100 80 60 40 20 0 ppm

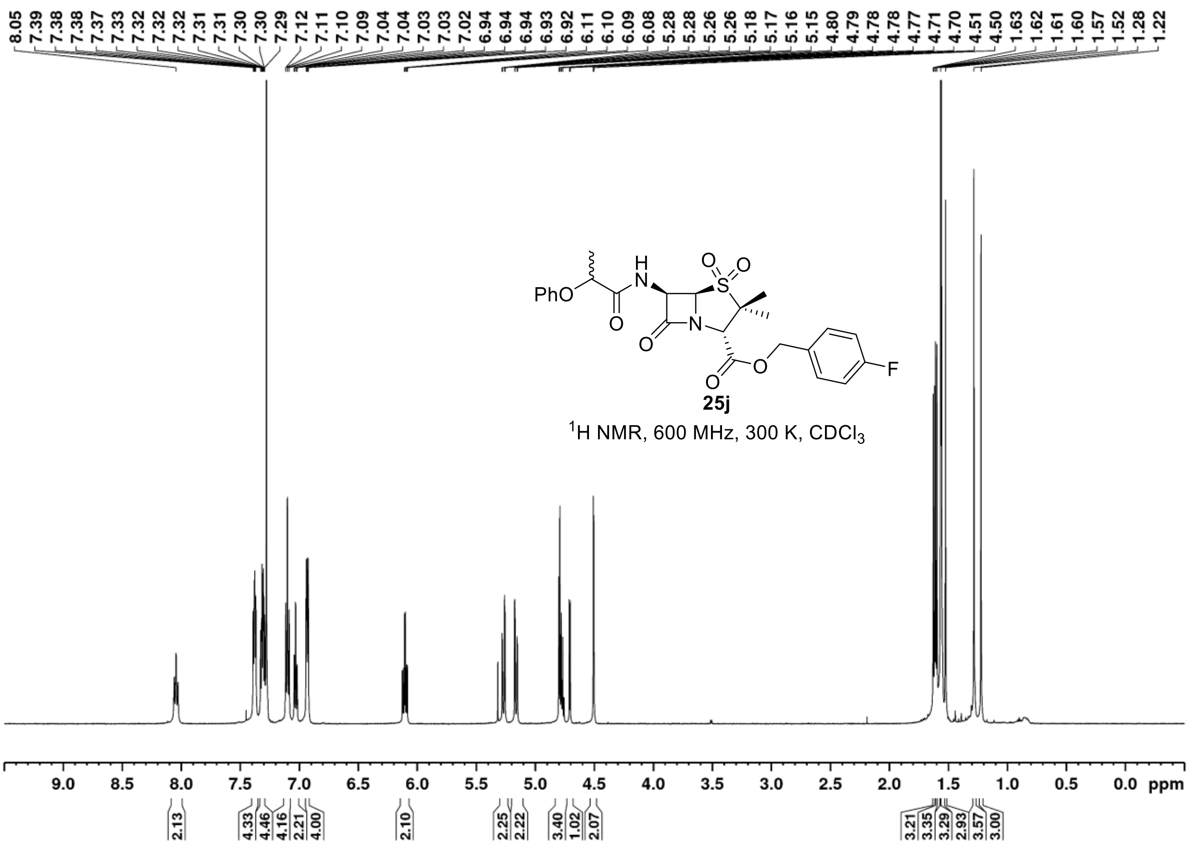
8.05 7.39 7.38 7.38 7.37 7.33 7.32 7.32 7.31 7.31 7.30 7.29 7.12 7.11 7.10 7.09 7.04 7.04 7.03 7.03 7.02 6.94 6.94 6.93 6.92 6.11 6.10 6.09 6.08 5.28 5.28 5.26 5.26 5.18 5.17 5.16 5.15 4.80 4.79 4.78 4.78 4.77 4.71 4.70 4.51 4.50 1.63 1.62 1.61 1.60 1.57 1.52 1.28 1.22
PhO
H
N
O
S
O
O
N
O
O
O
F
25j
^{1}H NMR, 600 MHz, 300 K, CDCl$_3$
9.0 8.5 8.0 7.5 7.0 6.5 6.0 5.5 5.0 4.5 4.0 3.5 3.0 2.5 2.0 1.5 1.0 0.5 0.0 ppm
2.13 4.33 4.46 4.16 2.21 4.00 2.10 2.25 2.22 3.40 1.02 2.07 3.21 3.35 3.29 2.93 3.57 3.00

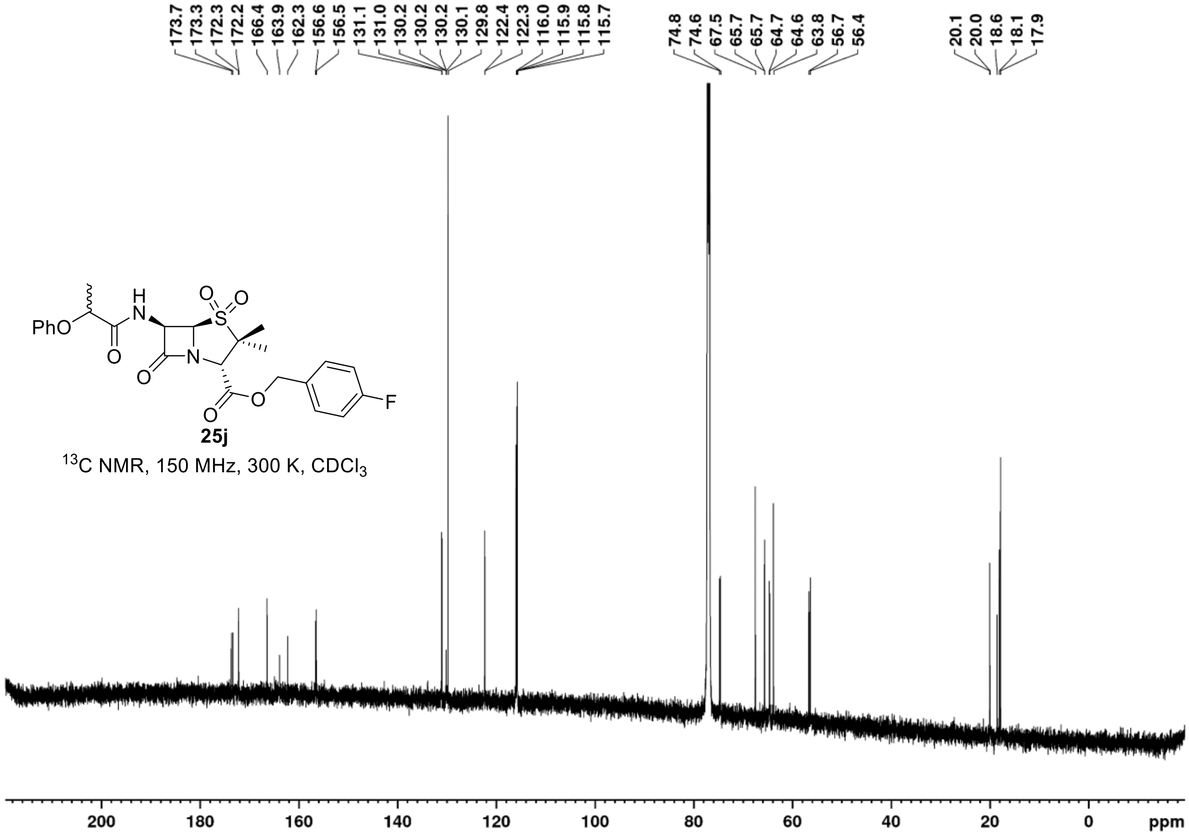
173.7 173.3 172.3 172.2 166.4 163.9 162.3 156.6 156.5 131.1 131.0 130.2 130.2 130.1 129.8 122.4 122.3 116.0 115.9 115.8 115.7 74.8 74.6 67.5 65.7 65.7 64.7 64.6 63.8 56.7 56.4 20.1 20.0 18.6 18.1 17.9
PhO
H
N
O
S
O
O
N
O
O
O
F
25j
^{13}C NMR, 150 MHz, 300 K, CDCl$_3$
200 180 160 140 120 100 80 60 40 20 0 ppm

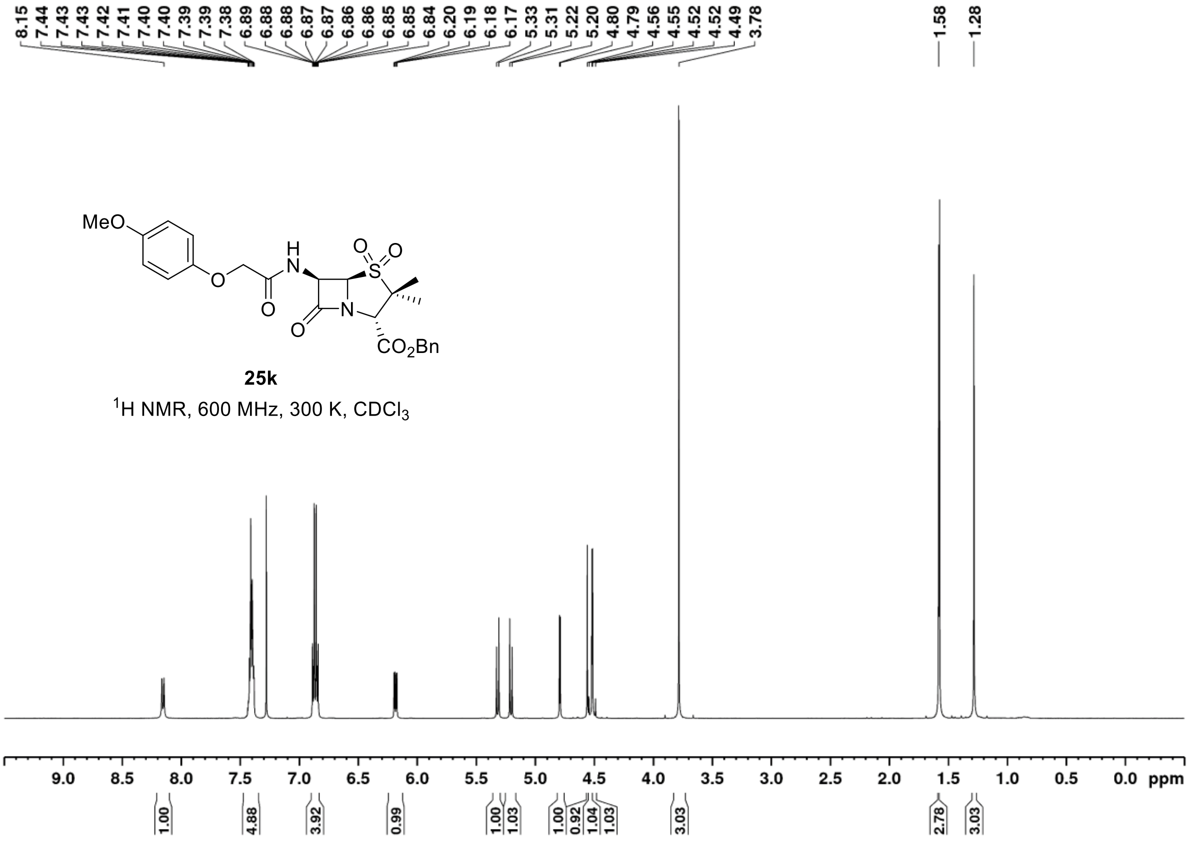
8.15 7.44 7.43 7.43 7.42 7.41 7.40 7.40 7.39 7.39 7.38 6.89 6.88 6.88 6.87 6.87 6.86 6.86 6.85 6.85 6.84 6.20 6.19 6.18 6.17 5.33 5.31 5.22 5.20 4.80 4.79 4.56 4.55 4.52 4.52 4.49 3.78
1.58
1.28
MeO
H
N
O
O
O
S
O
O
N
CO2Bn
25k
1H NMR, 600 MHz, 300 K, CDCl3
9.0 8.5 8.0 7.5 7.0 6.5 6.0 5.5 5.0 4.5 4.0 3.5 3.0 2.5 2.0 1.5 1.0 0.5 0.0 ppm
1.00 4.88 3.92 0.99 1.00 1.03 1.00 0.92 1.04 1.03 3.03 2.78 3.03

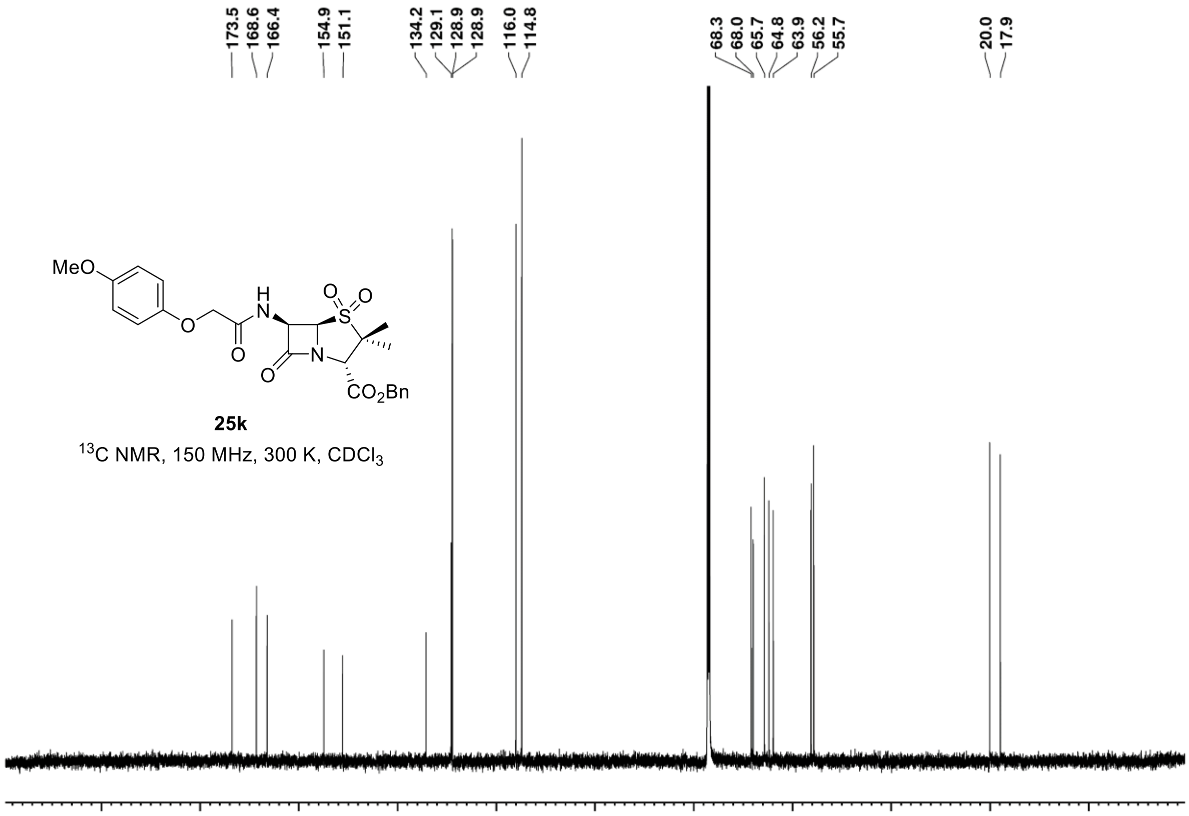
173.5 168.6 166.4 154.9 151.1 134.2 129.1 128.9 128.9 116.0 114.8 68.3 68.0 65.7 64.8 63.9 56.2 55.7 20.0 17.9
MeO
H
N
O
O
O
S
O
O
N
CO2Bn
25k
13C NMR, 150 MHz, 300 K, CDCl3
200 180 160 140 120 100 80 60 40 20 0 ppm

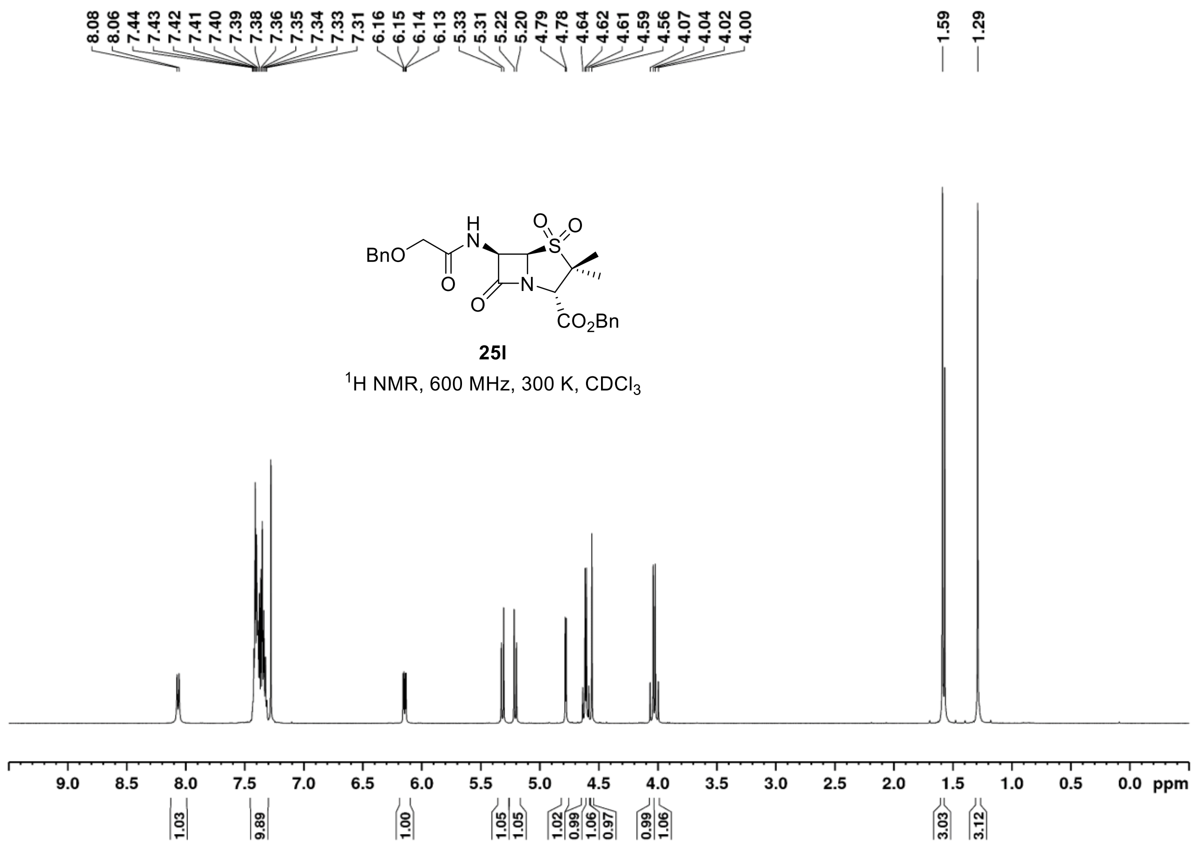
8.08
8.06
7.44
7.43
7.42
7.41
7.40
7.39
7.38
7.36
7.35
7.34
7.33
7.31
6.16
6.15
6.14
6.13
5.33
5.31
5.22
5.20
4.79
4.78
4.64
4.62
4.61
4.59
4.56
4.07
4.04
4.02
4.00
1.59
1.29
BnO
H
N
O
S
O
O
N
O
CO2Bn
25l
1H NMR, 600 MHz, 300 K, CDCl3
9.0
8.5
8.0
7.5
7.0
6.5
6.0
5.5
5.0
4.5
4.0
3.5
3.0
2.5
2.0
1.5
1.0
0.5
0.0
ppm
1.03
9.89
1.00
1.05
1.05
1.02
0.99
1.06
0.97
0.99
1.06
3.03
3.12

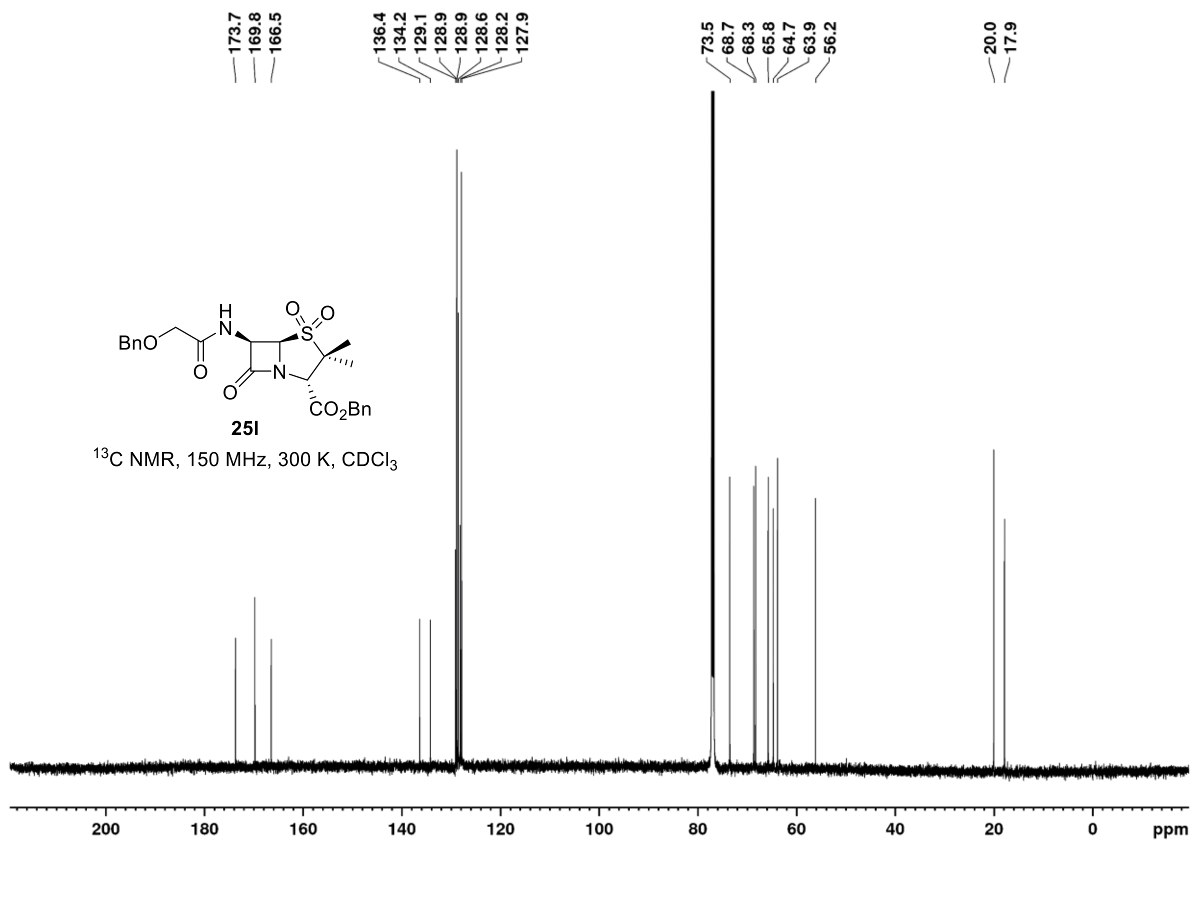
173.7
169.8
166.5
136.4
134.2
129.1
128.9
128.9
128.6
128.2
127.9
73.5
68.7
68.3
65.8
64.7
63.9
56.2
20.0
17.9
BnO
H
N
O
S
O
O
N
O
CO2Bn
25l
13C NMR, 150 MHz, 300 K, CDCl3
200
180
160
140
120
100
80
60
40
20
0
ppm

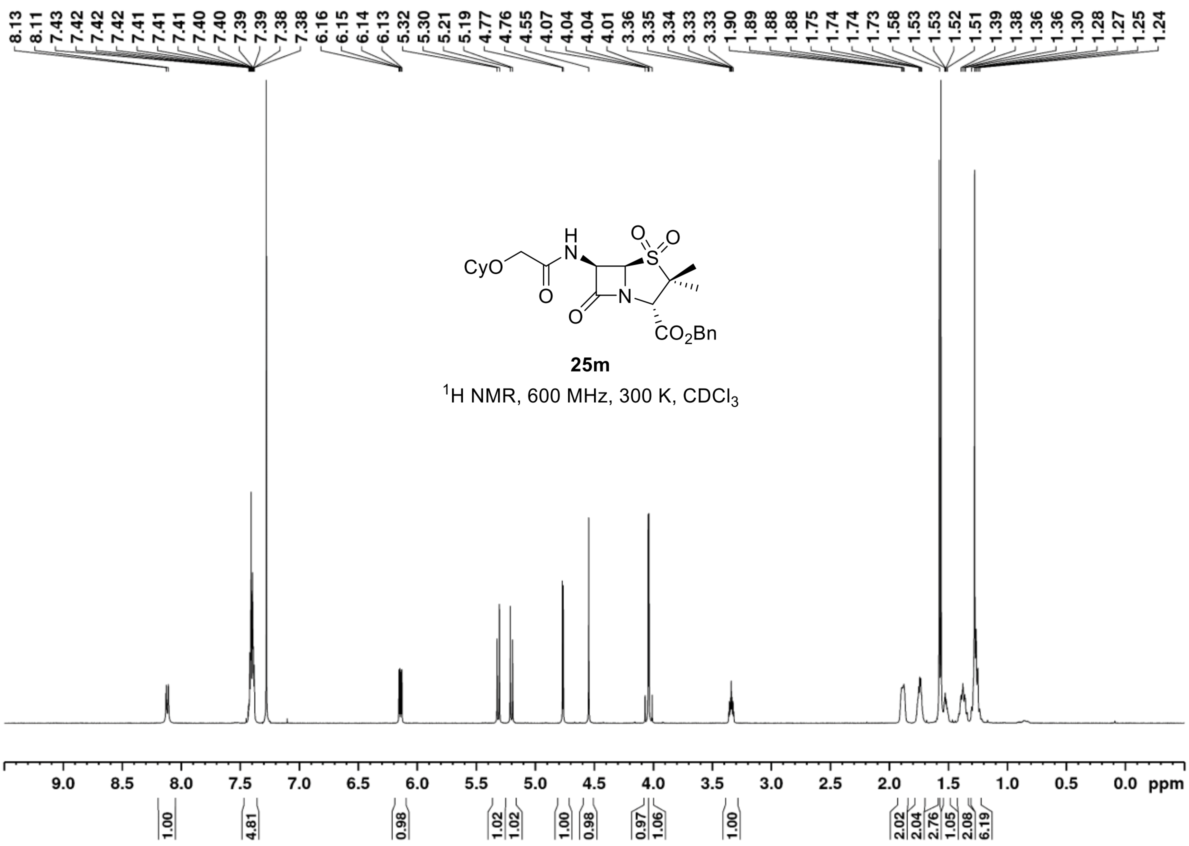
8.13 8.11 7.43 7.42 7.42 7.42 7.41 7.41 7.41 7.40 7.40 7.39 7.39 7.38 7.38 6.16 6.15 6.14 6.13 5.32 5.30 5.21 5.19 4.77 4.76 4.55 4.07 4.04 4.04 4.01 3.36 3.35 3.34 3.33 3.33 1.90 1.89 1.88 1.88 1.75 1.74 1.74 1.73 1.58 1.53 1.53 1.52 1.51 1.39 1.38 1.36 1.36 1.30 1.28 1.27 1.25 1.24
CyO
H
N
O
S
O
O
N
CO2Bn
25m
1H NMR, 600 MHz, 300 K, CDCl3
9.0 8.5 8.0 7.5 7.0 6.5 6.0 5.5 5.0 4.5 4.0 3.5 3.0 2.5 2.0 1.5 1.0 0.5 0.0 ppm
1.00 4.81 0.98 1.02 1.02 1.00 0.98 0.97 1.06 1.00 2.02 2.04 2.76 1.05 2.08 6.19

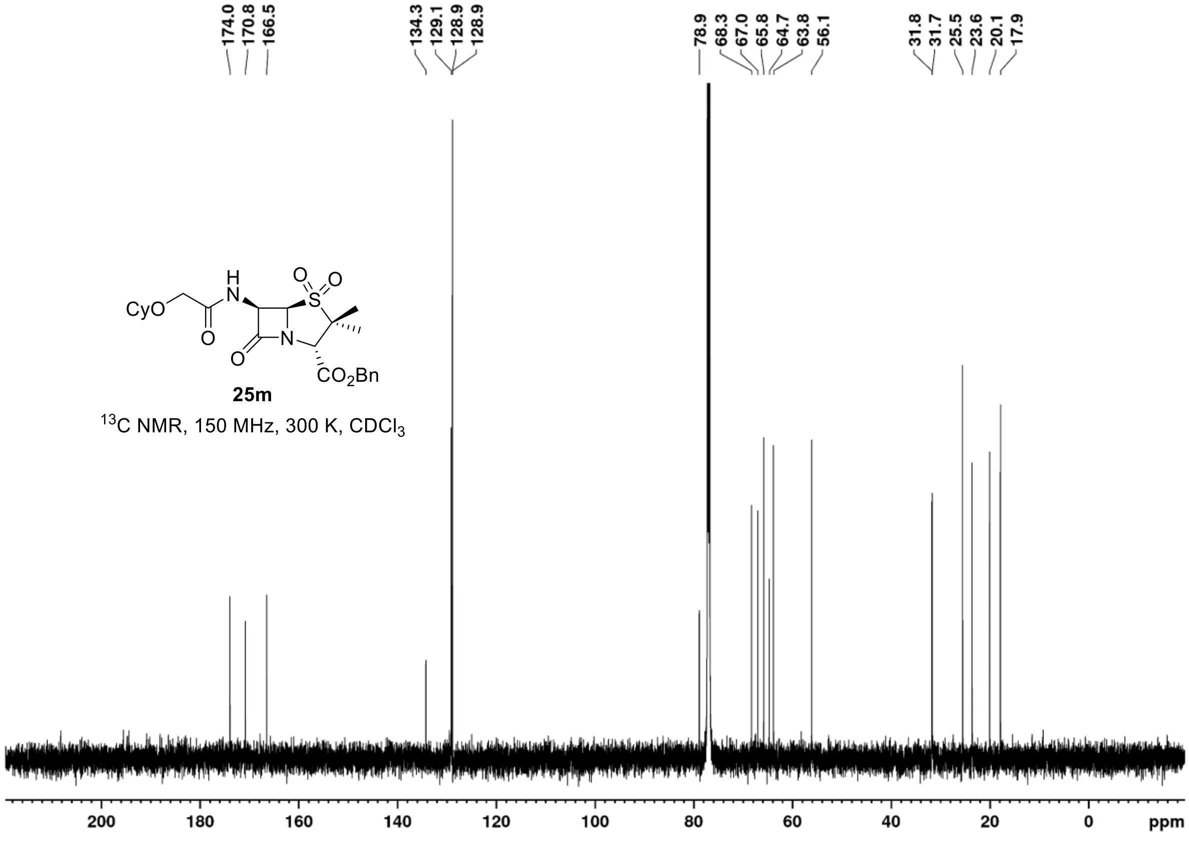
174.0 170.8 166.5 134.3 129.1 128.9 128.9 78.9 68.3 67.0 65.8 64.7 63.8 56.1 31.8 31.7 25.5 23.6 20.1 17.9
CyO
H
N
O
S
O
O
N
CO2Bn
25m
13C NMR, 150 MHz, 300 K, CDCl3
200 180 160 140 120 100 80 60 40 20 0 ppm

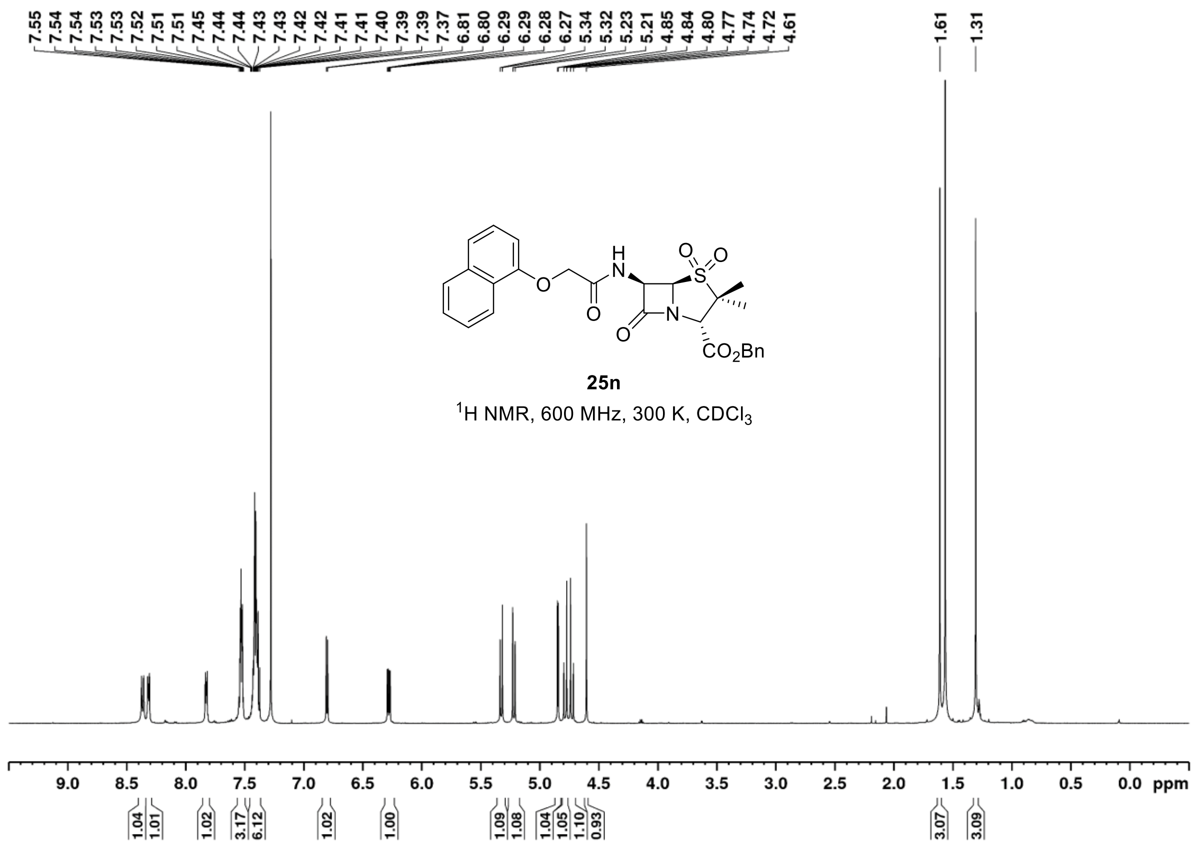
7.55
7.54
7.54
7.53
7.53
7.52
7.51
7.51
7.45
7.44
7.44
7.43
7.43
7.42
7.42
7.41
7.41
7.40
7.39
7.39
7.37
6.81
6.80
6.29
6.29
6.28
6.27
5.34
5.32
5.23
5.21
4.85
4.84
4.80
4.77
4.74
4.72
4.61
1.61
1.31
H
N
O
O
O
O
S
N
O
CO2Bn
25n
1H NMR, 600 MHz, 300 K, CDCl3
9.0
8.5
8.0
7.5
7.0
6.5
6.0
5.5
5.0
4.5
4.0
3.5
3.0
2.5
2.0
1.5
1.0
0.5
0.0
ppm
1.04
1.01
1.02
3.17
6.12
1.02
1.00
1.09
1.08
1.04
1.05
1.10
0.93
3.07
3.09

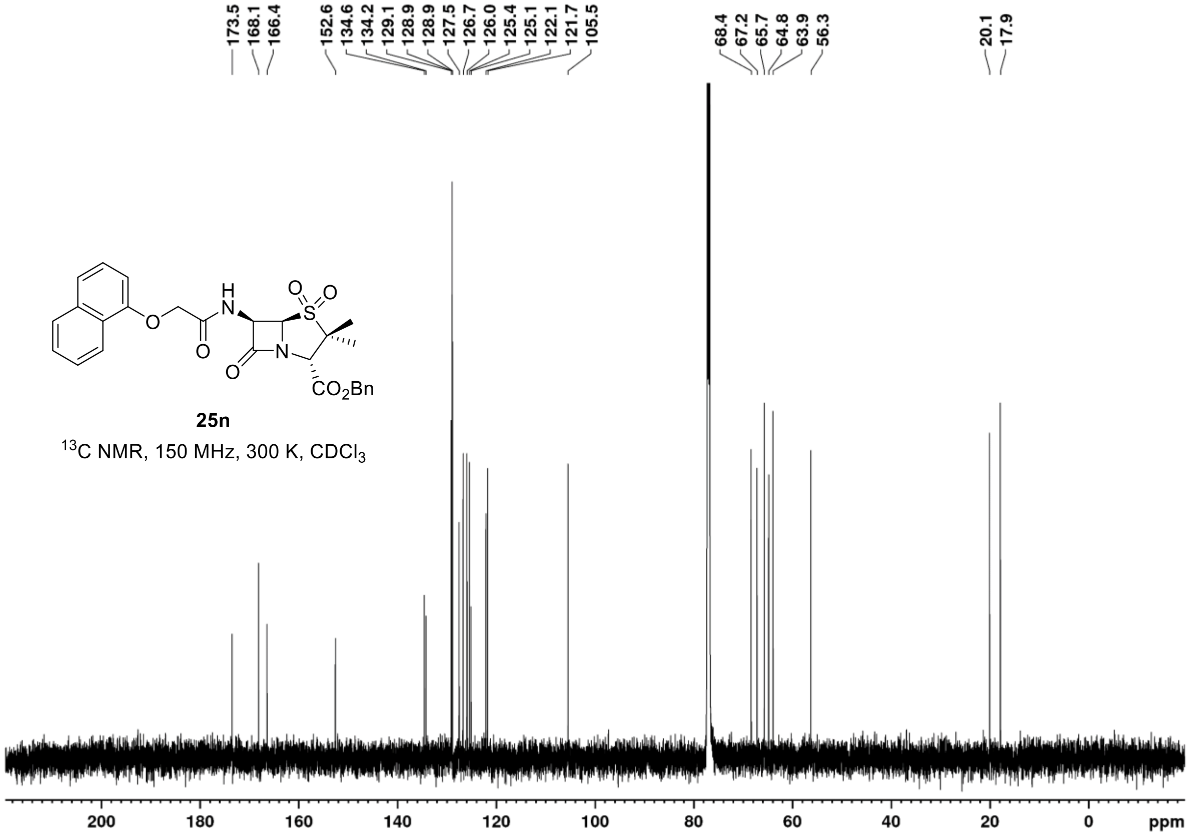
173.5
168.1
166.4
152.6
134.6
134.2
129.1
128.9
128.9
127.5
126.7
126.0
125.4
125.1
122.1
121.7
105.5
68.4
67.2
65.7
64.8
63.9
56.3
20.1
17.9
H
N
O
O
O
O
S
N
O
CO2Bn
25n
13C NMR, 150 MHz, 300 K, CDCl3
200
180
160
140
120
100
80
60
40
20
0
ppm

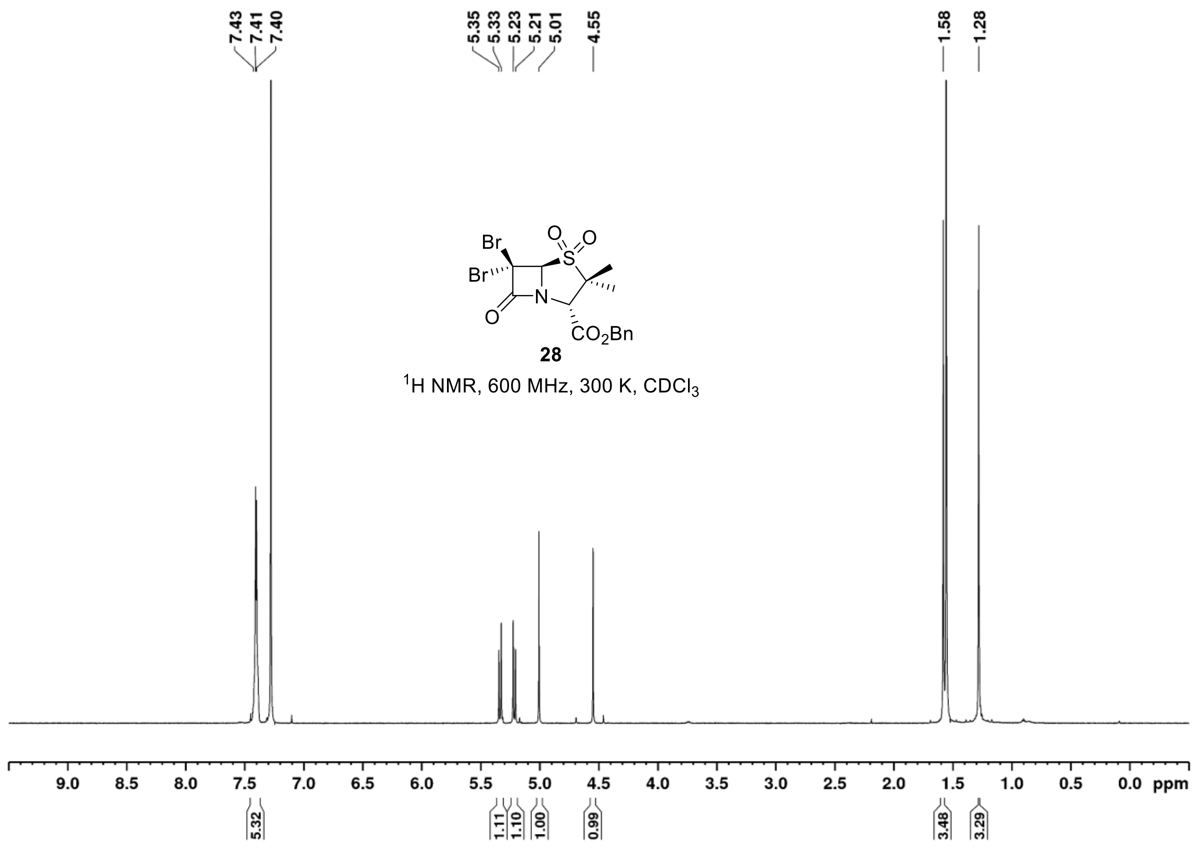
7.43
7.41
7.40
5.35
5.33
5.23
5.21
5.01
4.55
1.58
1.28
Br
Br
O
O
S
O
N
CO2Bn
28
1H NMR, 600 MHz, 300 K, CDCl3
9.0
8.5
8.0
7.5
7.0
6.5
6.0
5.5
5.0
4.5
4.0
3.5
3.0
2.5
2.0
1.5
1.0
0.5
0.0
ppm
5.32
1.11
1.10
1.00
0.99
3.48
3.29

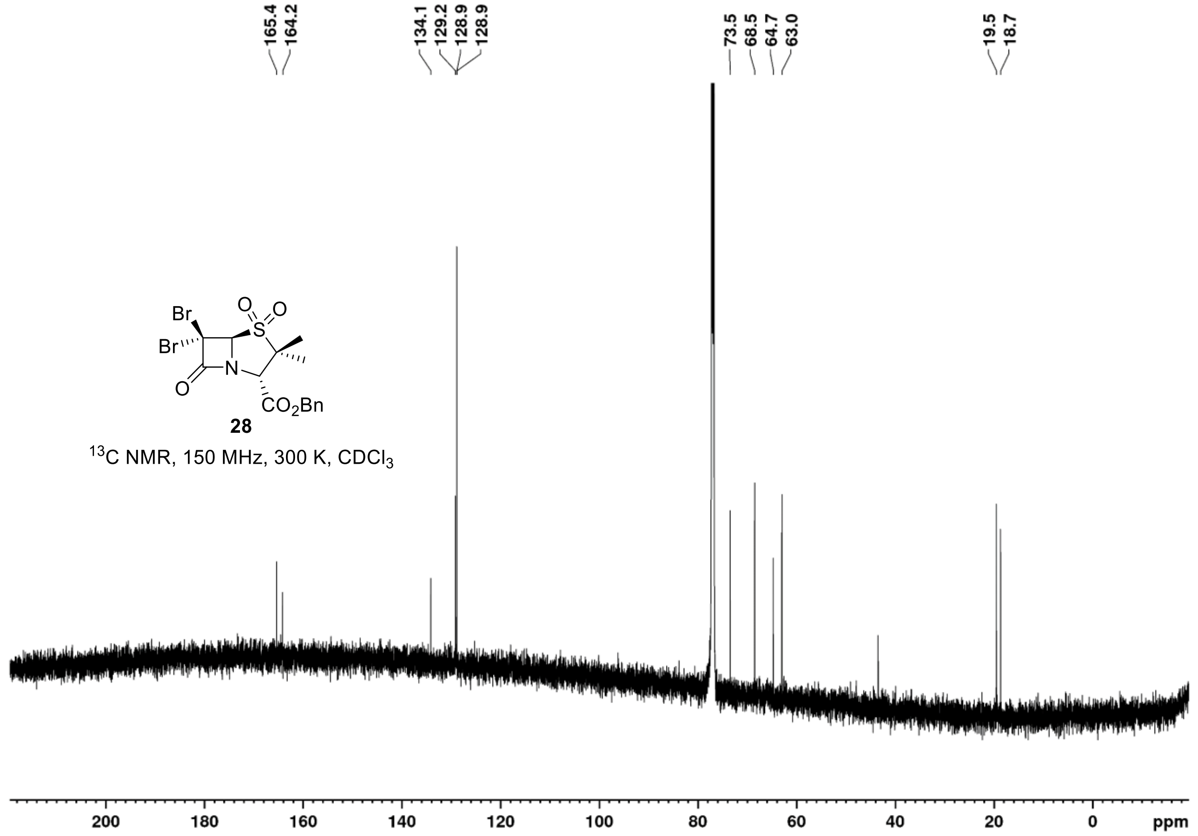
165.4
164.2
134.1
129.2
128.9
128.9
73.5
68.5
64.7
63.0
19.5
18.7
Br
Br
O
O
S
O
N
CO2Bn
28
13C NMR, 150 MHz, 300 K, CDCl3
200
180
160
140
120
100
80
60
40
20
0
ppm

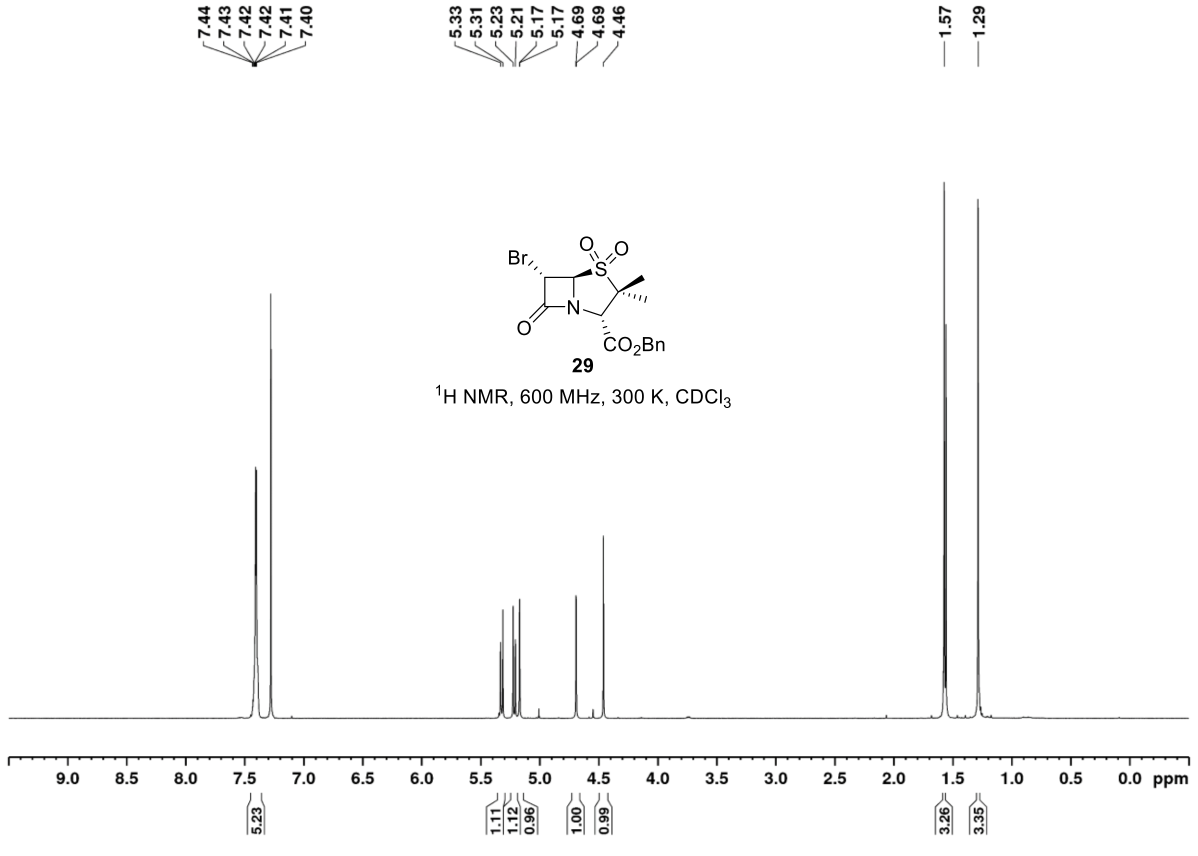
7.44
7.43
7.42
7.42
7.41
7.40
5.33
5.31
5.23
5.21
5.17
5.17
4.69
4.69
4.46
1.57
1.29
Br
O
S
O
N
O
CO2Bn
29
1H NMR, 600 MHz, 300 K, CDCl3
9.0 8.5 8.0 7.5 7.0 6.5 6.0 5.5 5.0 4.5 4.0 3.5 3.0 2.5 2.0 1.5 1.0 0.5 0.0 ppm
5.23
1.11
1.12
0.96
1.00
0.99
3.26
3.35

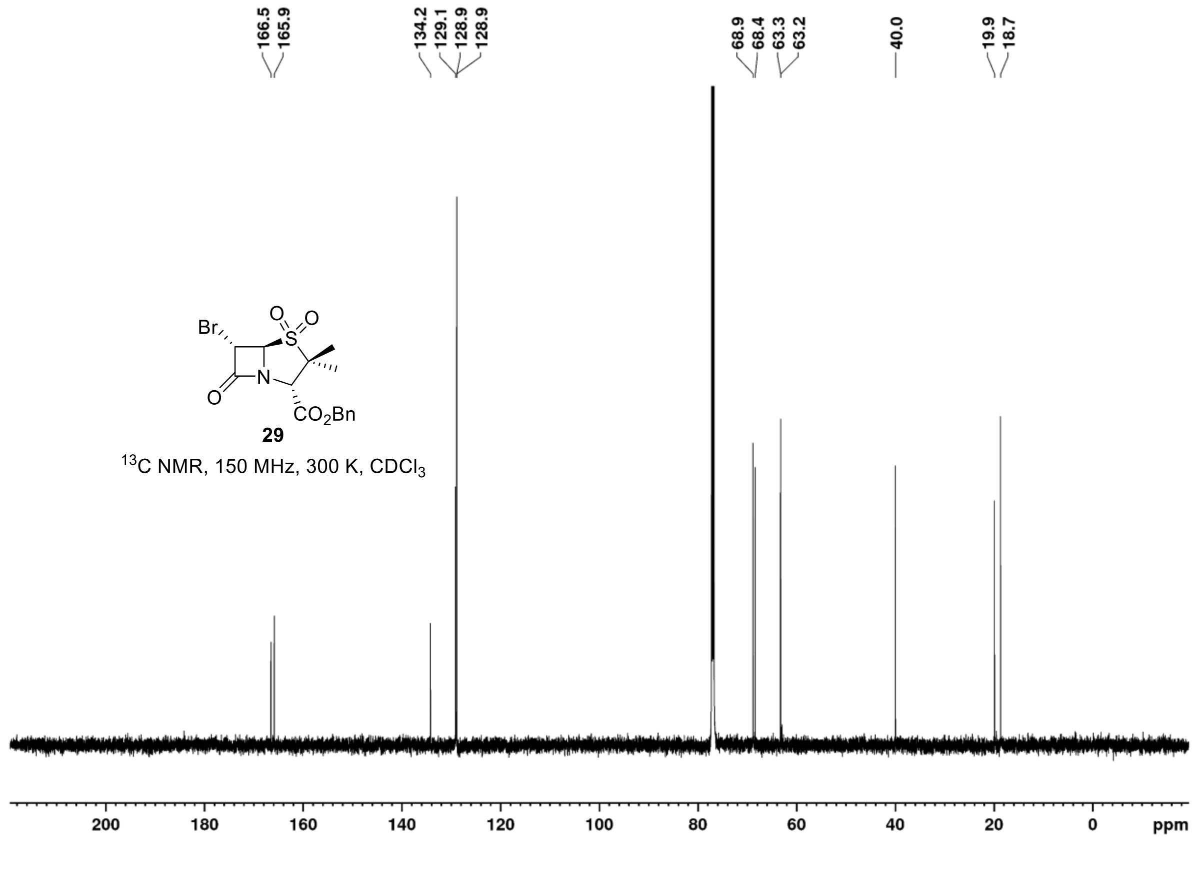
166.5
165.9
134.2
129.1
128.9
128.9
68.9
68.4
63.3
63.2
40.0
19.9
18.7
Br
O
S
O
N
O
CO2Bn
29
13C NMR, 150 MHz, 300 K, CDCl3
200 180 160 140 120 100 80 60 40 20 0 ppm

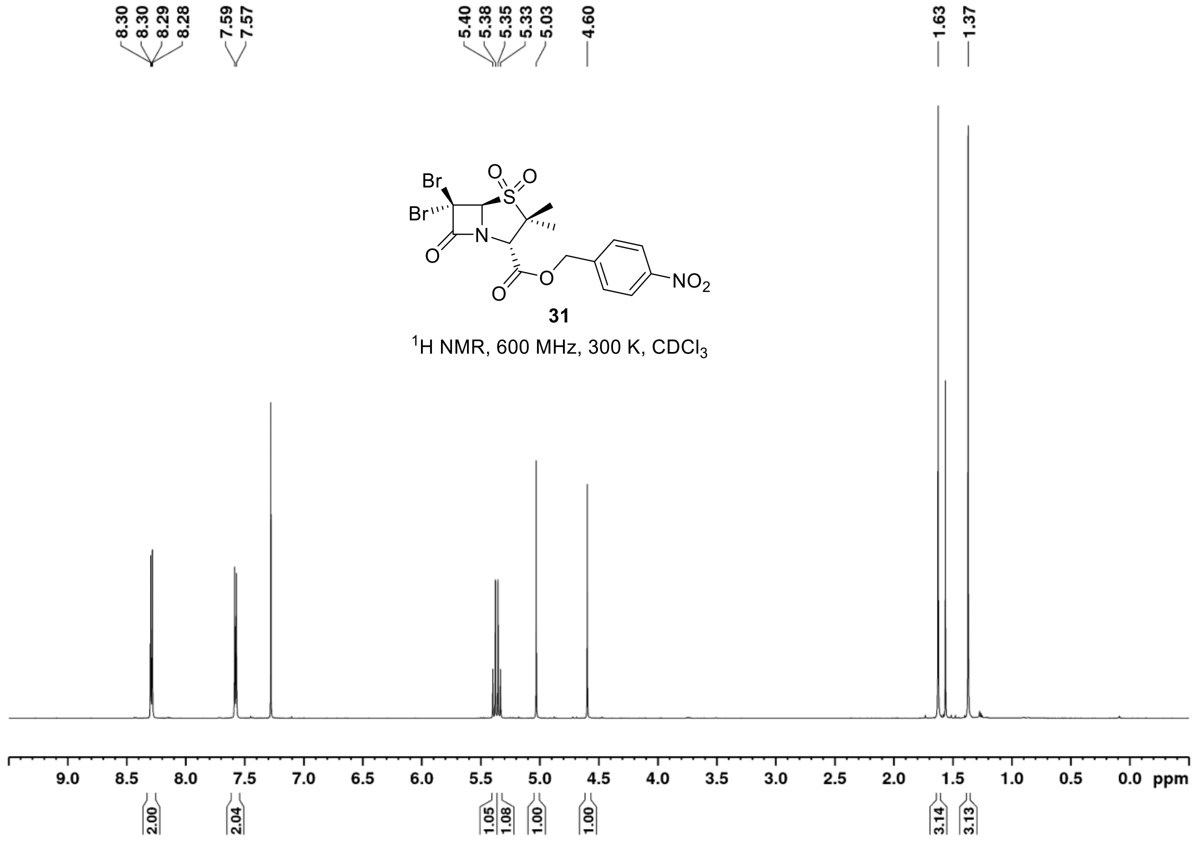
8.30
8.30
8.29
8.28
7.59
7.57
5.40
5.38
5.35
5.33
5.03
4.60
1.63
1.37
Br
Br
O
O
S
O
N
O
O
NO$_2$
31
^{1}H NMR, 600 MHz, 300 K, CDCl$_3$
9.0
8.5
8.0
7.5
7.0
6.5
6.0
5.5
5.0
4.5
4.0
3.5
3.0
2.5
2.0
1.5
1.0
0.5
0.0
ppm
2.00
2.04
1.05
1.08
1.00
1.00
3.14
3.13

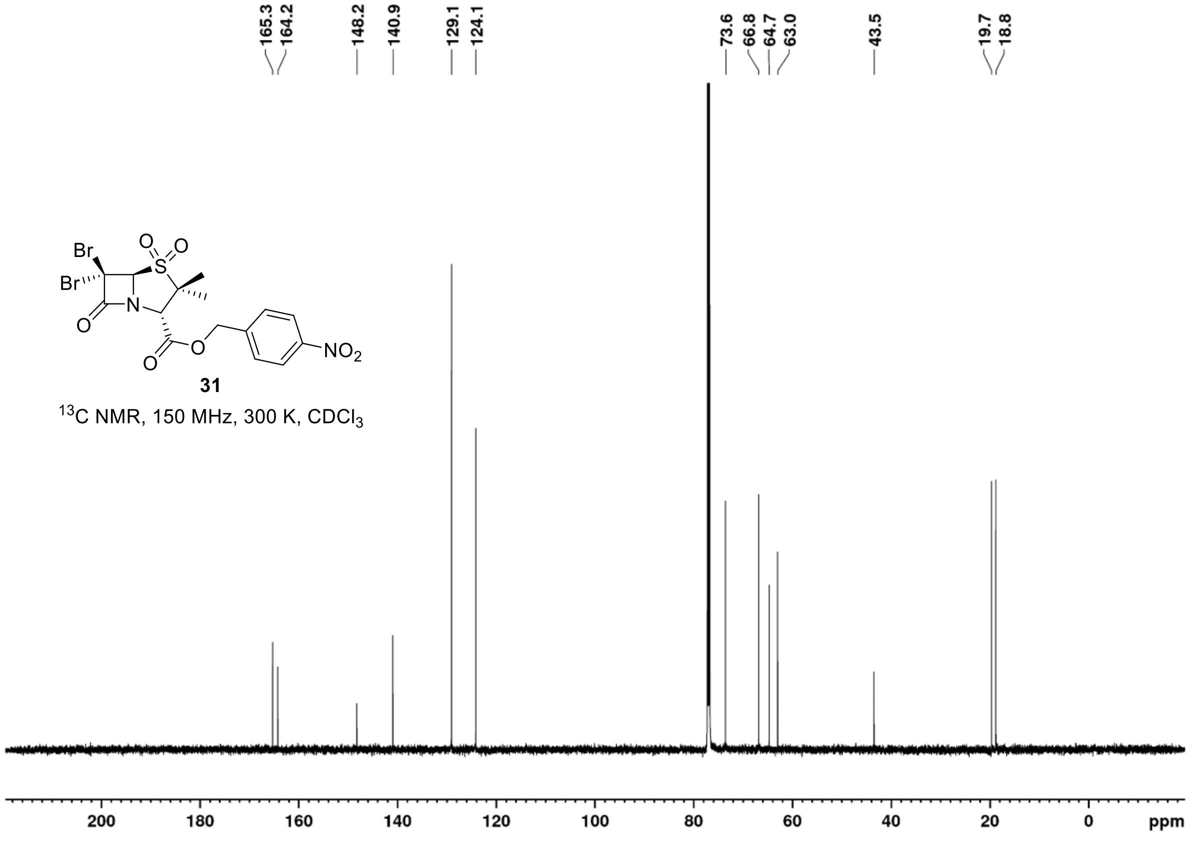
165.3
164.2
148.2
140.9
129.1
124.1
73.6
66.8
64.7
63.0
43.5
19.7
18.8
Br
Br
O
O
S
O
N
O
O
NO$_2$
31
^{13}C NMR, 150 MHz, 300 K, CDCl$_3$
200
180
160
140
120
100
80
60
40
20
0
ppm

8. HPLC traces of selected penicillin sulfones prepared for this study. HPLC traces were recorded using a semi-preparative HPLC machine (Shimadzu UK Ltd.) equipped with a reverse phase column (ACE 5 C18, dimensions: 100 mm length, 21.2 mm inner diameter, 5.0 µm particle size). A linear gradient (2−98%$_{v/v}$ over 15 min) of acetonitrile in water (each containing 0.1%$_{v/v}$ trifluoroacetic acid) was used as eluent (flow rate: 12 mL/min; wavelength monitored: 220 nm). The area% of the major peak (labelled with the retention time, t_R) is ≥95% with respect to the sum of the area% of all peaks detected (excluding the injection peak at ~2.5 min).

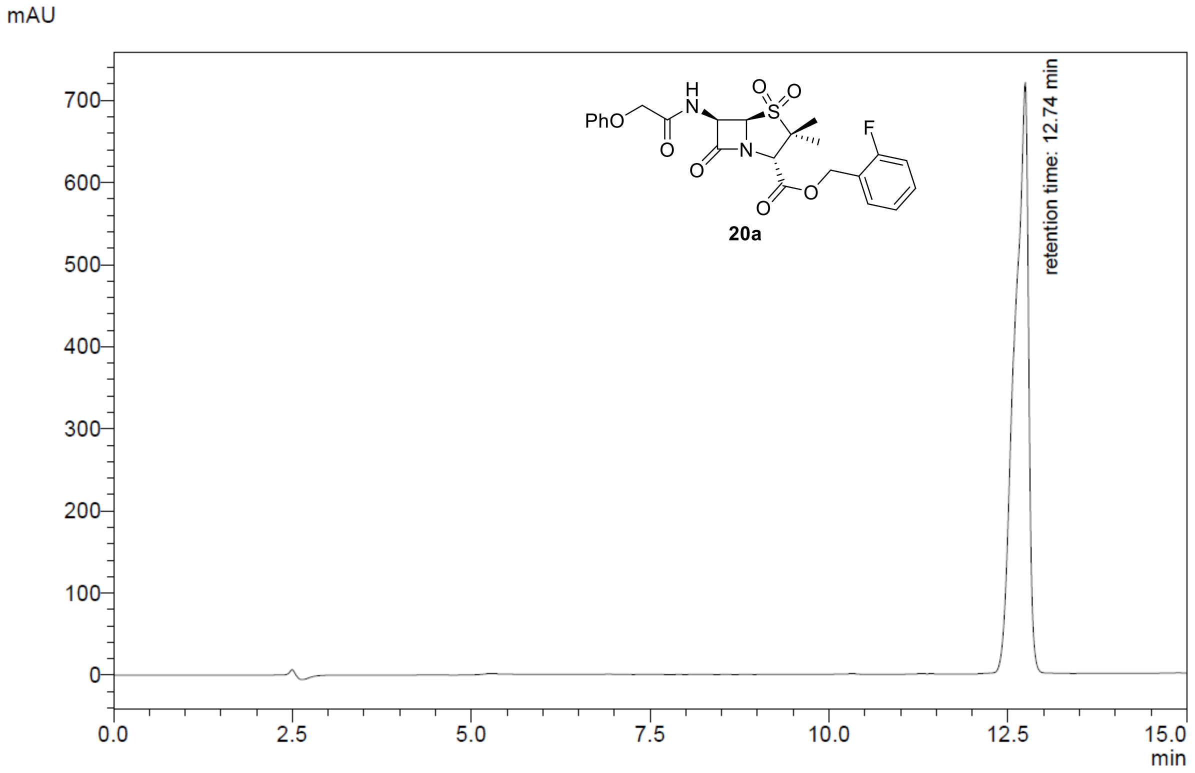

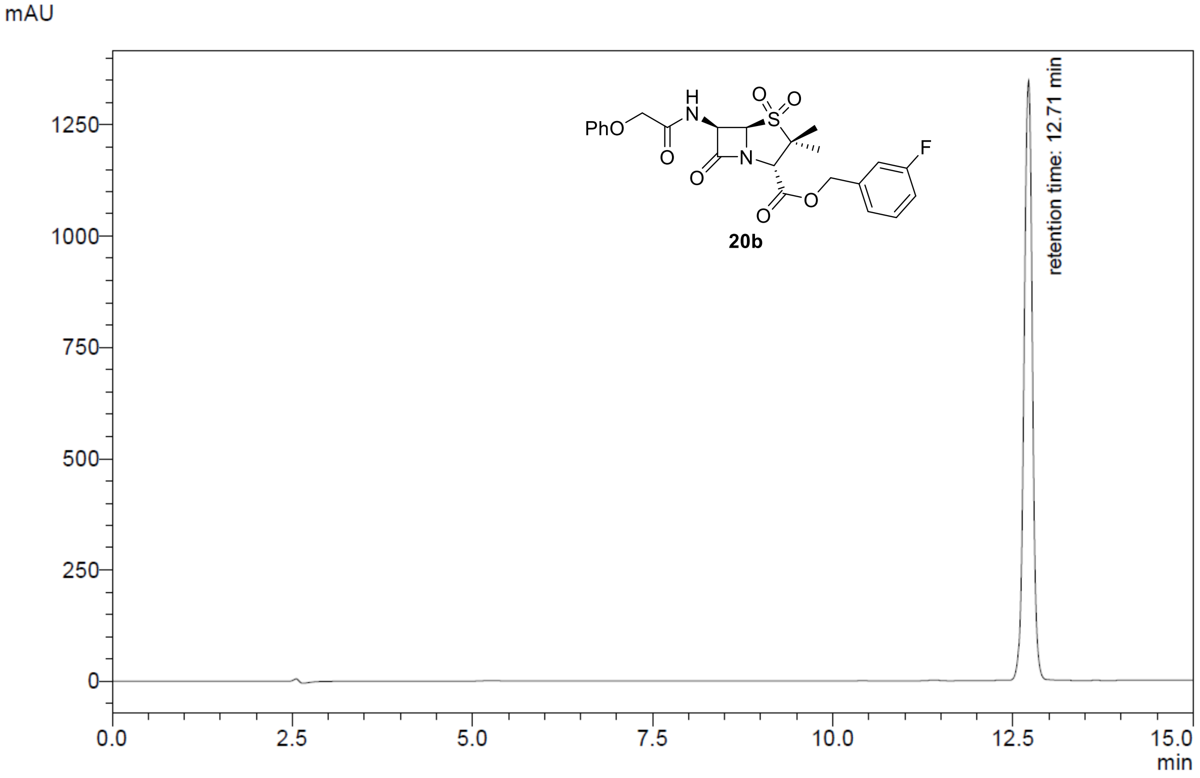

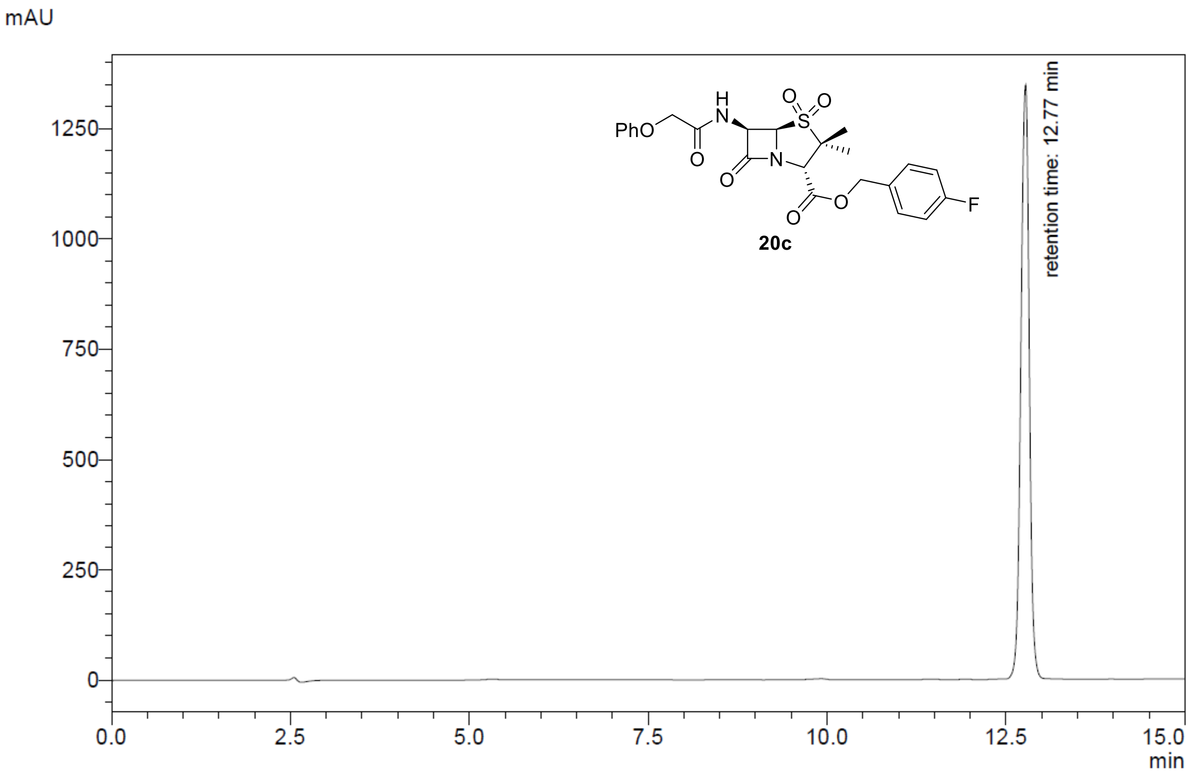
mAU
1250
1000
750
500
250
0
0.0
2.5
5.0
7.5
10.0
12.5
15.0
min
retention time: 12.77 min
PhO
H
N
O
O
S
O
N
O
O
O
F
20c

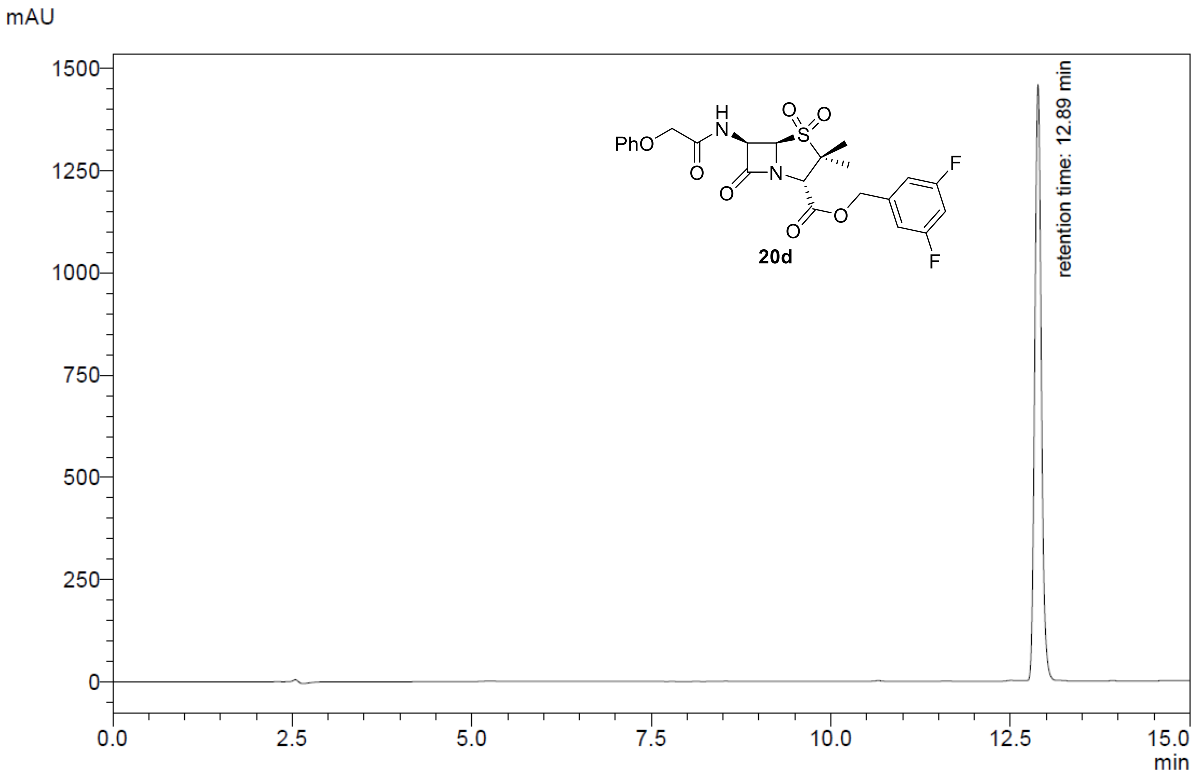
mAU
1500
1250
1000
750
500
250
0
0.0
2.5
5.0
7.5
10.0
12.5
15.0
min
retention time: 12.89 min
PhO
H
N
O
O
S
O
N
O
O
O
F
F
20d

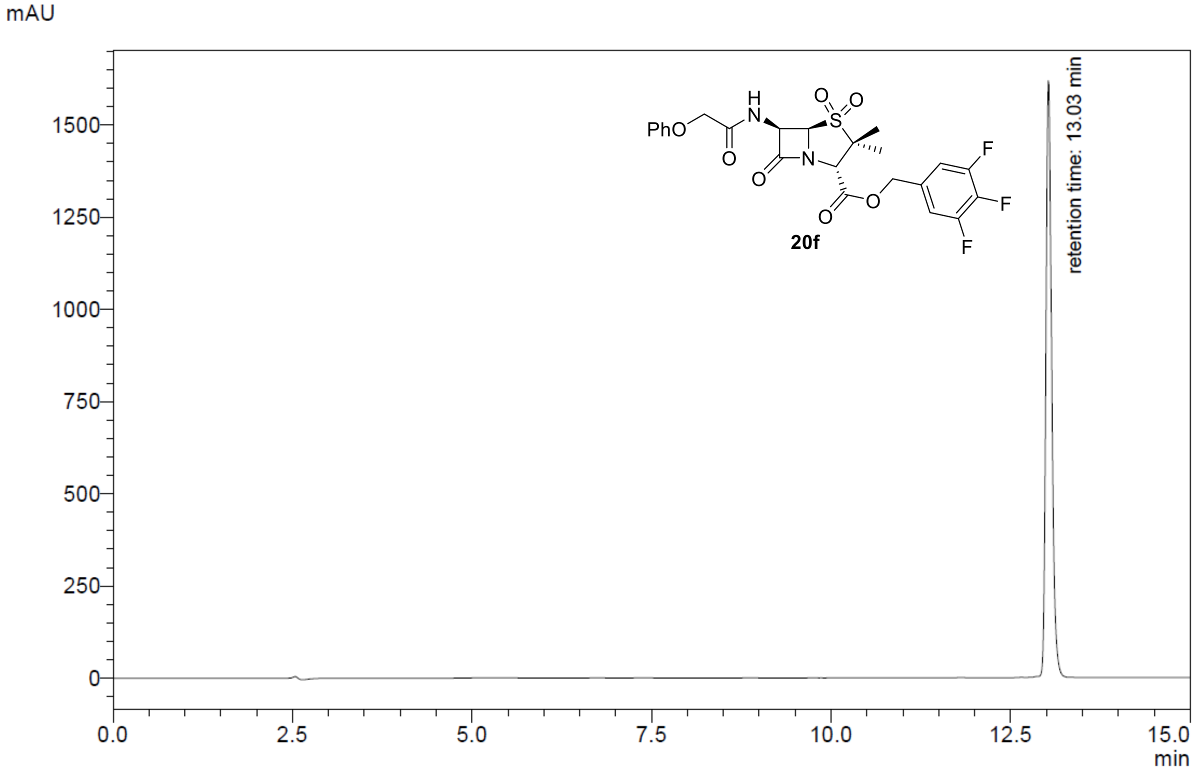
mAU
PhO
H
N
O
S
O
O
N
O
O
F
F
F
20f
retention time: 13.03 min
min

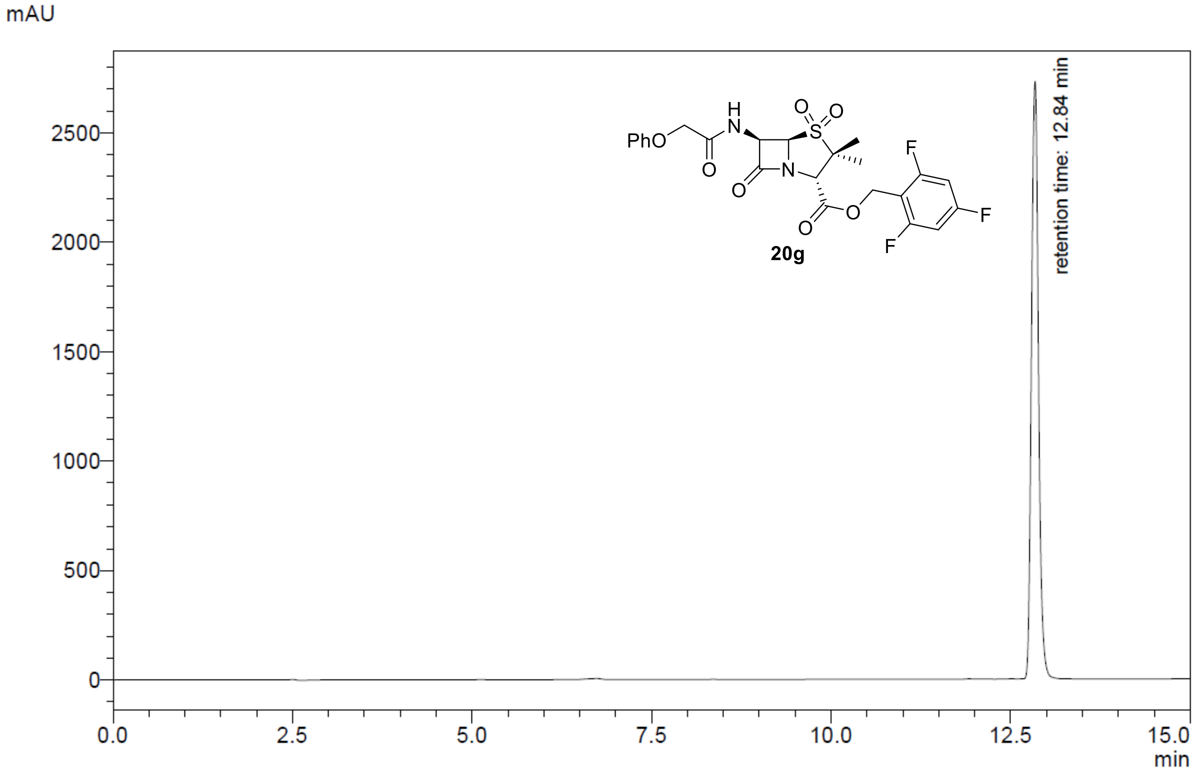
mAU
PhO
H
N
O
S
O
O
N
O
O
F
F
F
20g
retention time: 12.84 min
min

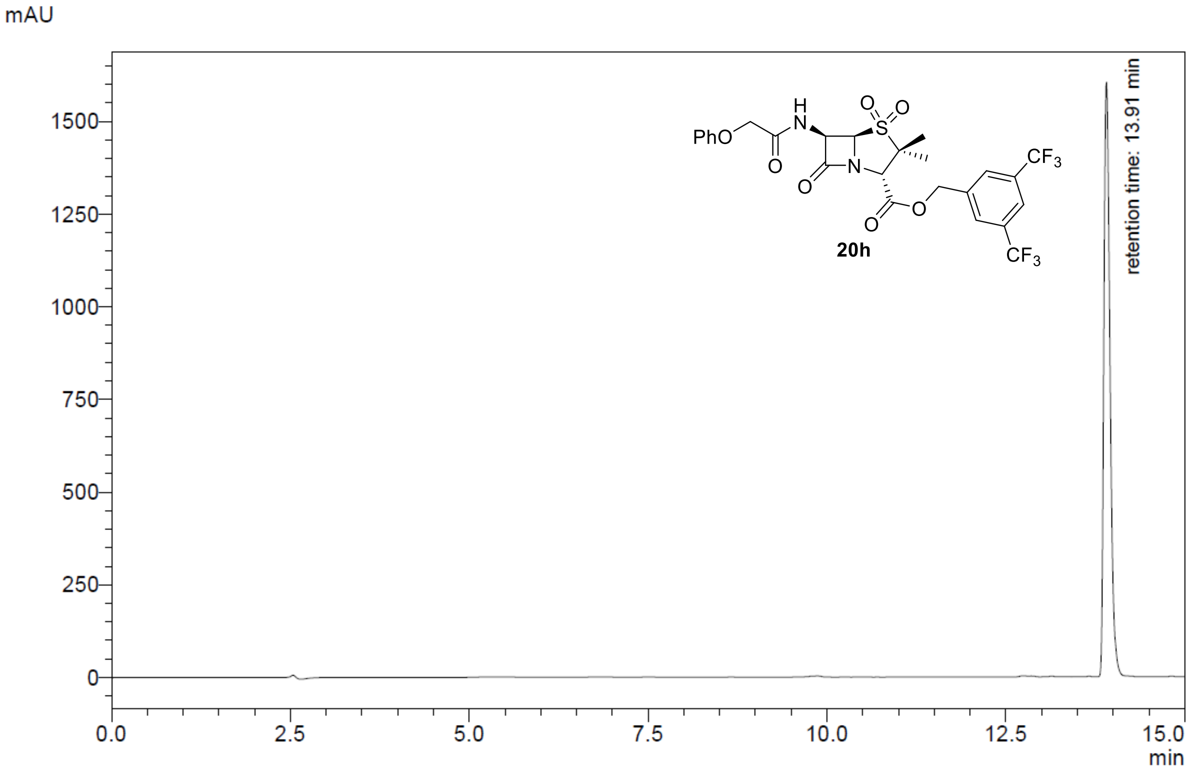
mAU
1500
1250
1000
750
500
250
0
0.0
2.5
5.0
7.5
10.0
12.5
15.0
min
PhO
H
N
O
O
S
O
O
N
O
O
CF3
CF3
20h
retention time: 13.91 min

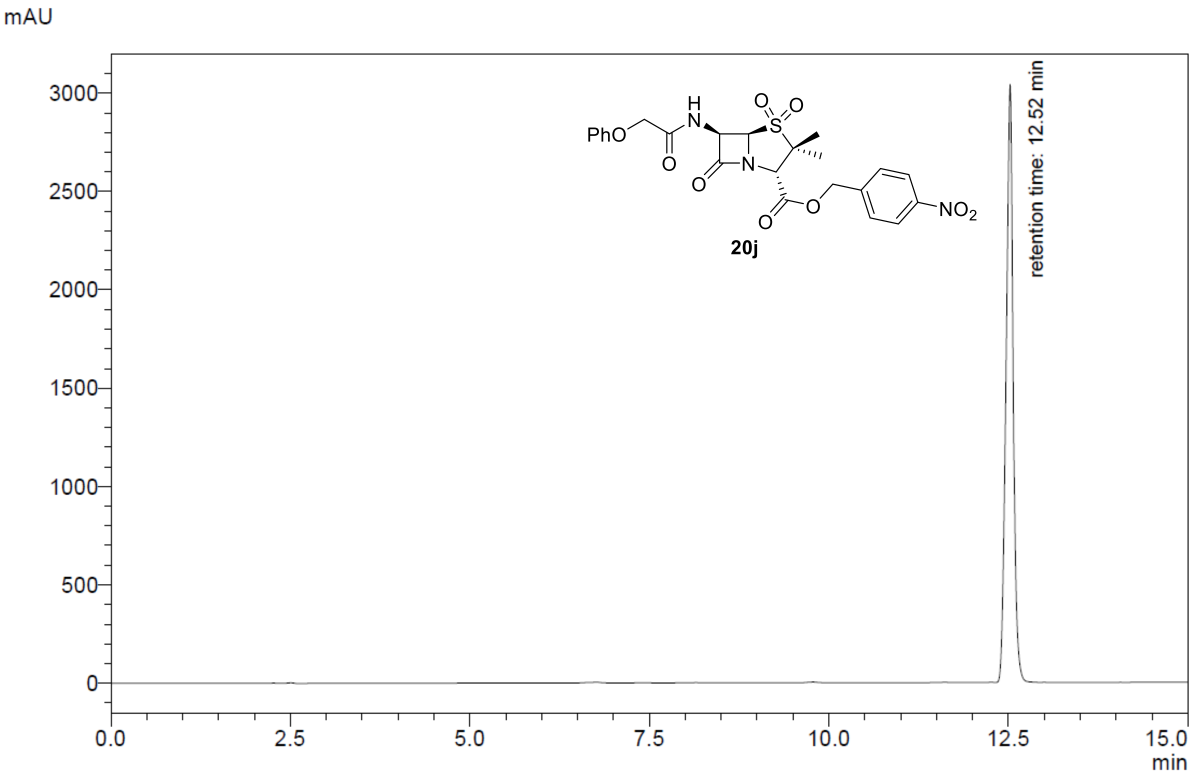
mAU
3000
2500
2000
1500
1000
500
0
0.0
2.5
5.0
7.5
10.0
12.5
15.0
min
PhO
H
N
O
O
S
O
O
N
O
O
NO2
20j
retention time: 12.52 min

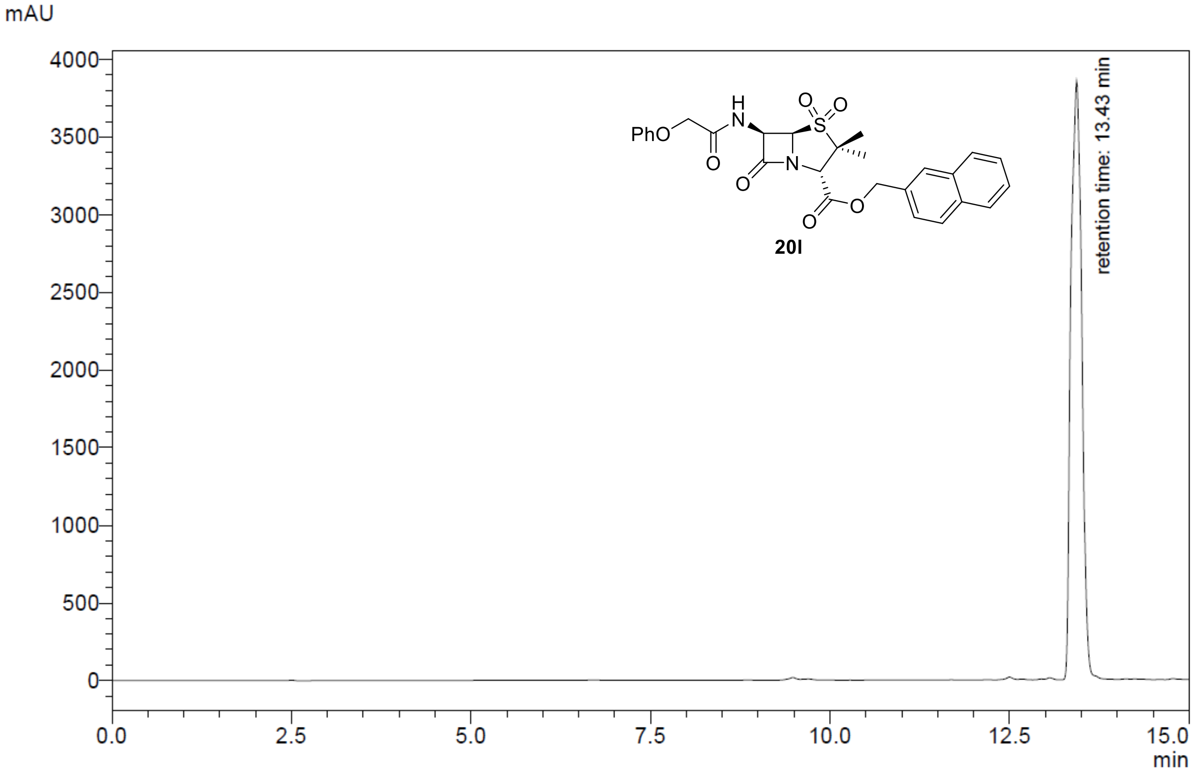
mAU
4000
3500
3000
2500
2000
1500
1000
500
0
0.0
2.5
5.0
7.5
10.0
12.5
15.0
min
retention time: 13.43 min
PhO
H
N
O
O
S
O
O
N
O
O
20l

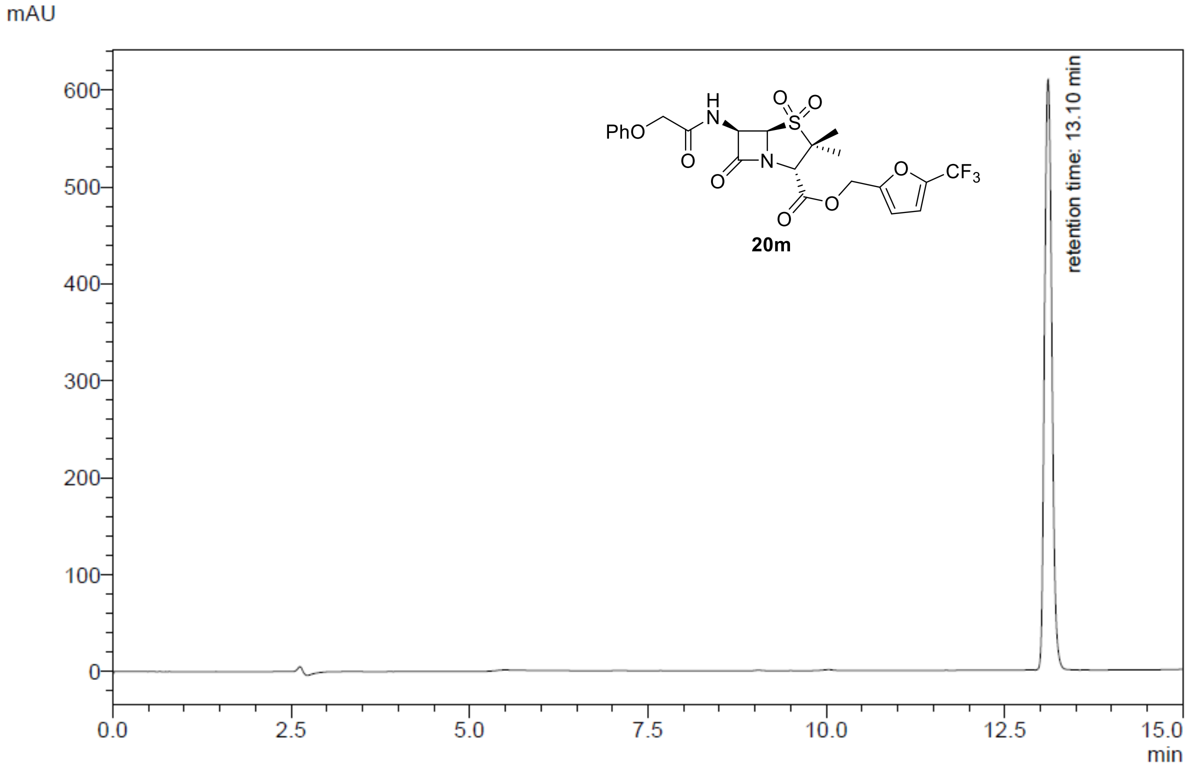
mAU
600
500
400
300
200
100
0
0.0
2.5
5.0
7.5
10.0
12.5
15.0
min
retention time: 13.10 min
PhO
H
N
O
O
S
O
O
N
O
O
O
CF_3
20m

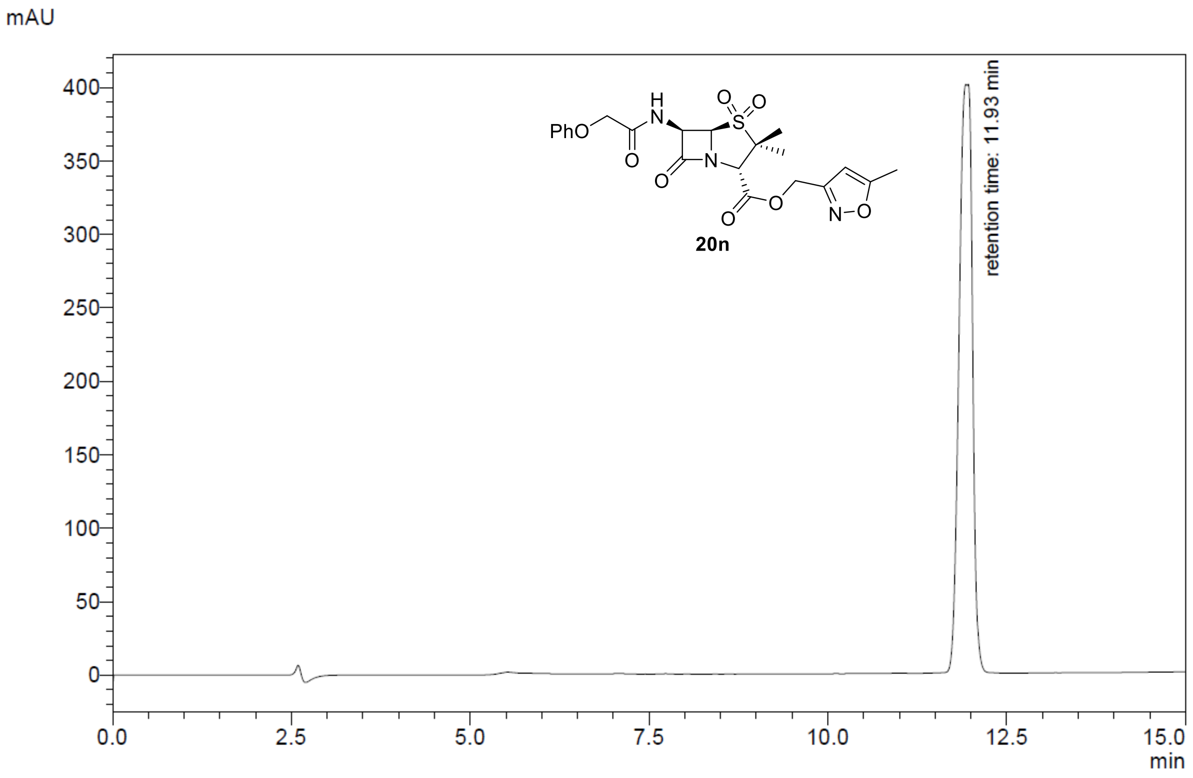
mAU
400
350
300
250
200
150
100
50
0
0.0
2.5
5.0
7.5
10.0
12.5
15.0
min
retention time: 11.93 min
PhO
H
N
O
S
O
N
O
O
O
N-O
20n

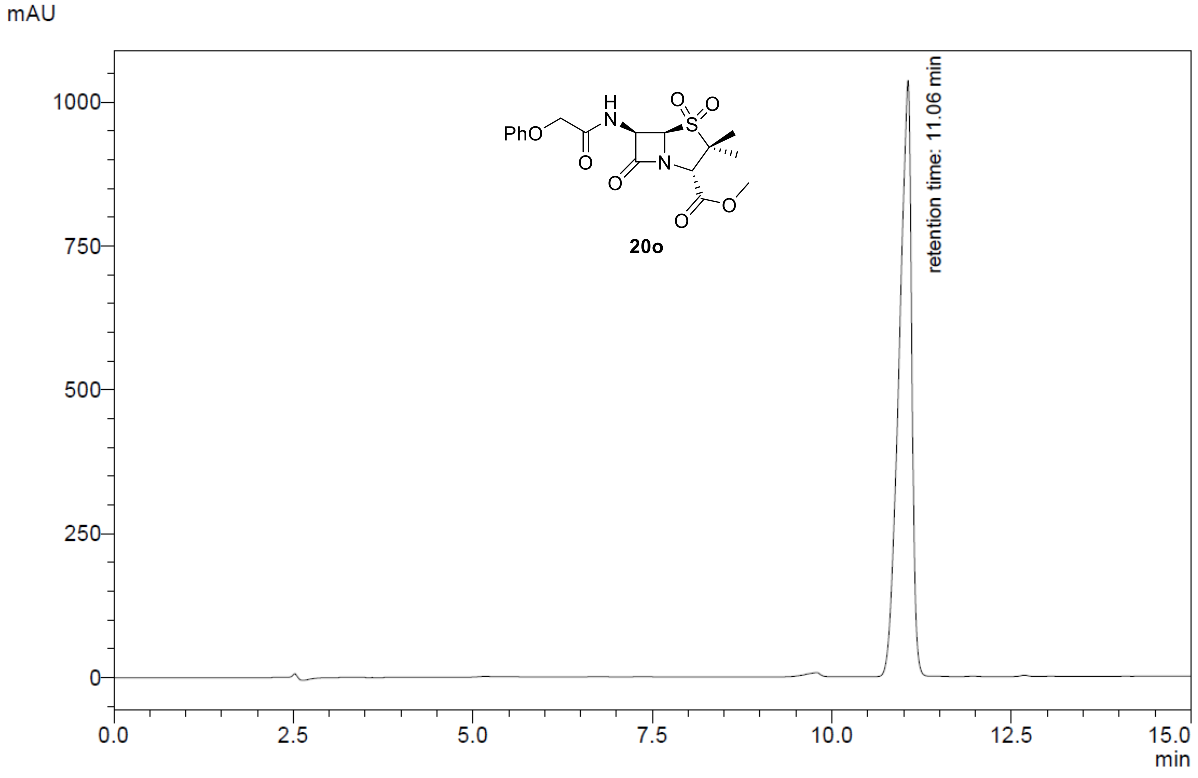
mAU
1000
750
500
250
0
0.0
2.5
5.0
7.5
10.0
12.5
15.0
min
retention time: 11.06 min
PhO
H
N
O
S
O
N
O
O
O
20o

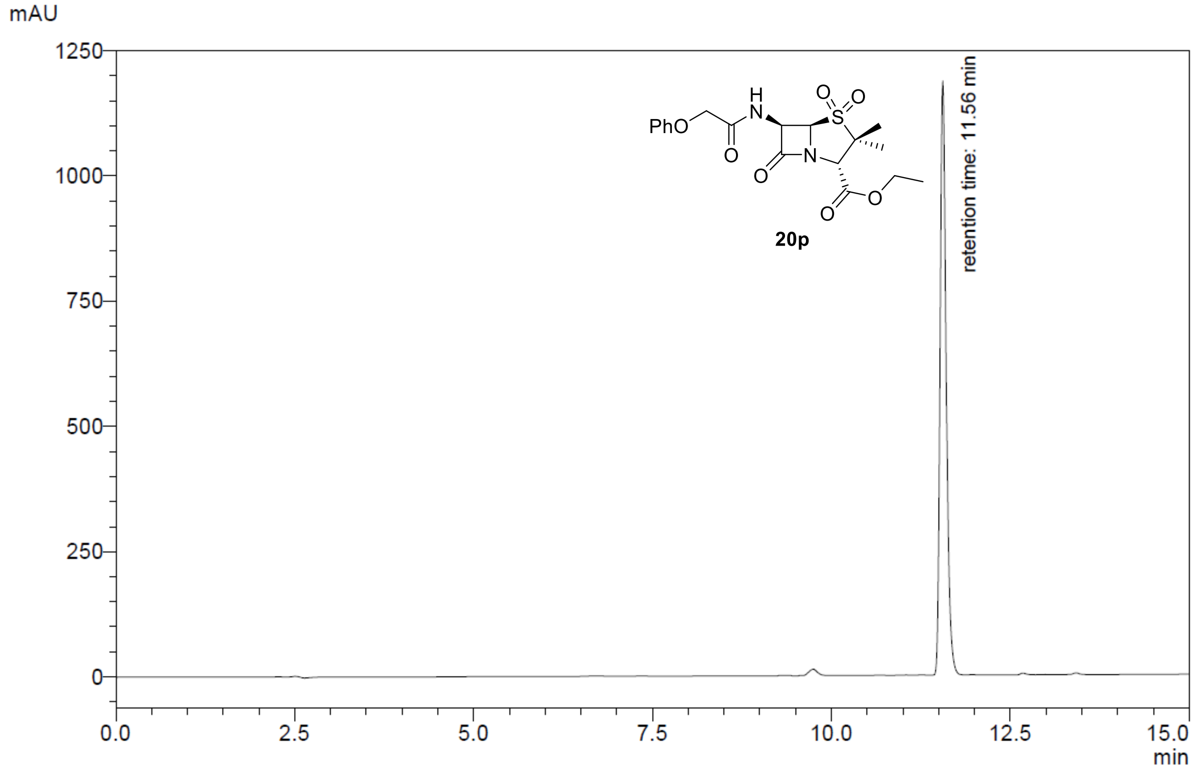
mAU
1250
1000
750
500
250
0
0.0
2.5
5.0
7.5
10.0
12.5
15.0
min
retention time: 11.56 min
PhO
H
N
O
S
O
N
20p

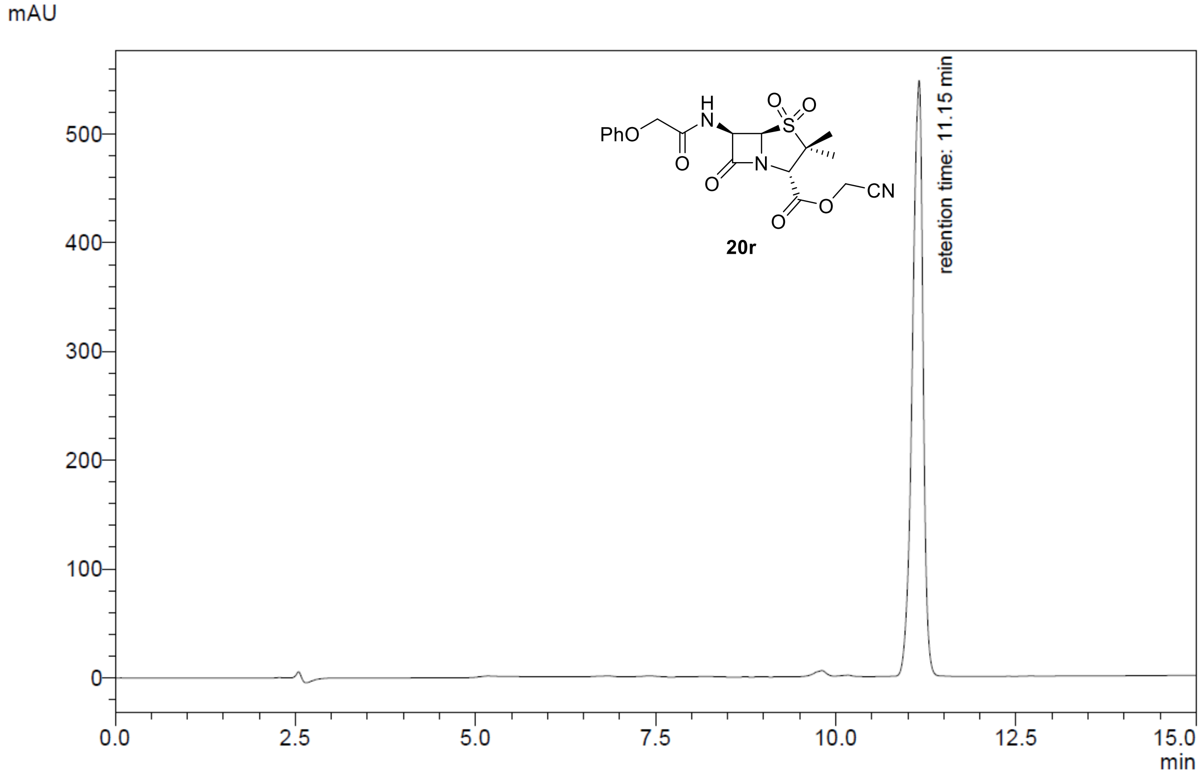
mAU
500
400
300
200
100
0
0.0
2.5
5.0
7.5
10.0
12.5
15.0
min
retention time: 11.15 min
PhO
H
N
O
S
O
N
CN
20r

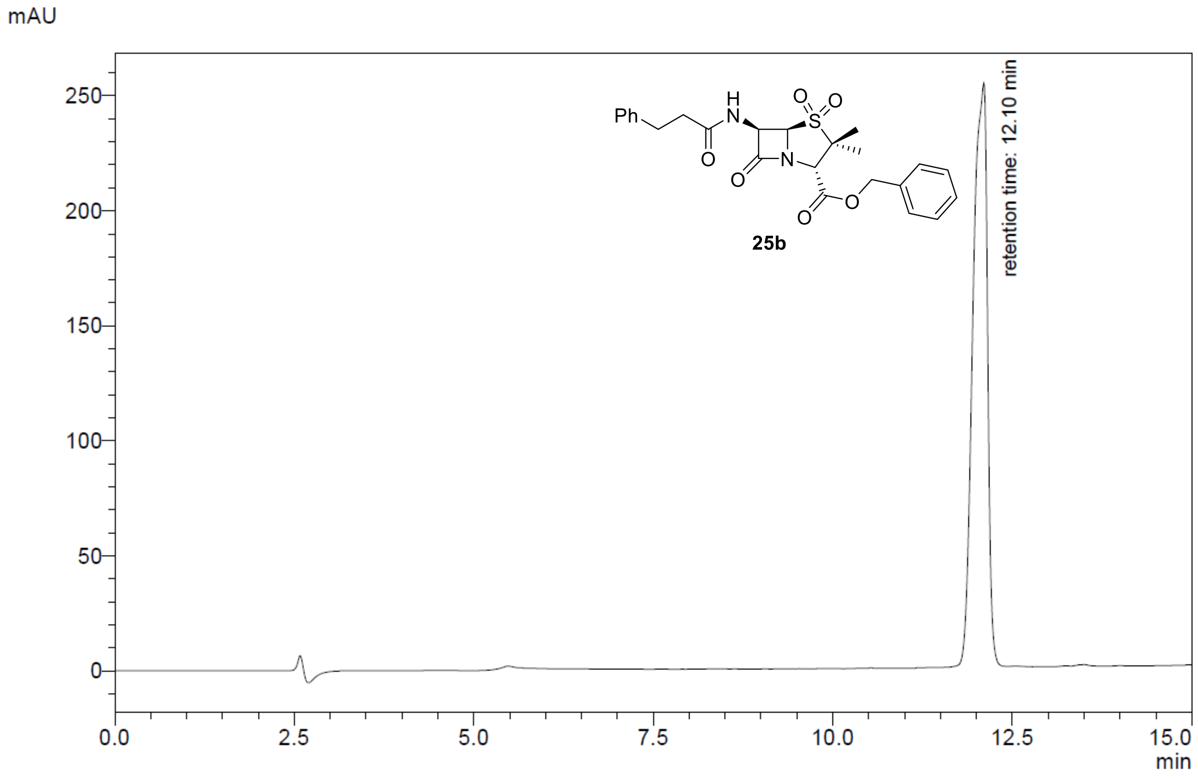

mAU
250
200
150
100
50
0
0.0
2.5
5.0
7.5
10.0
12.5
15.0
min
Ph
H
N
O
S
N
O
O
25b
retention time: 12.10 min

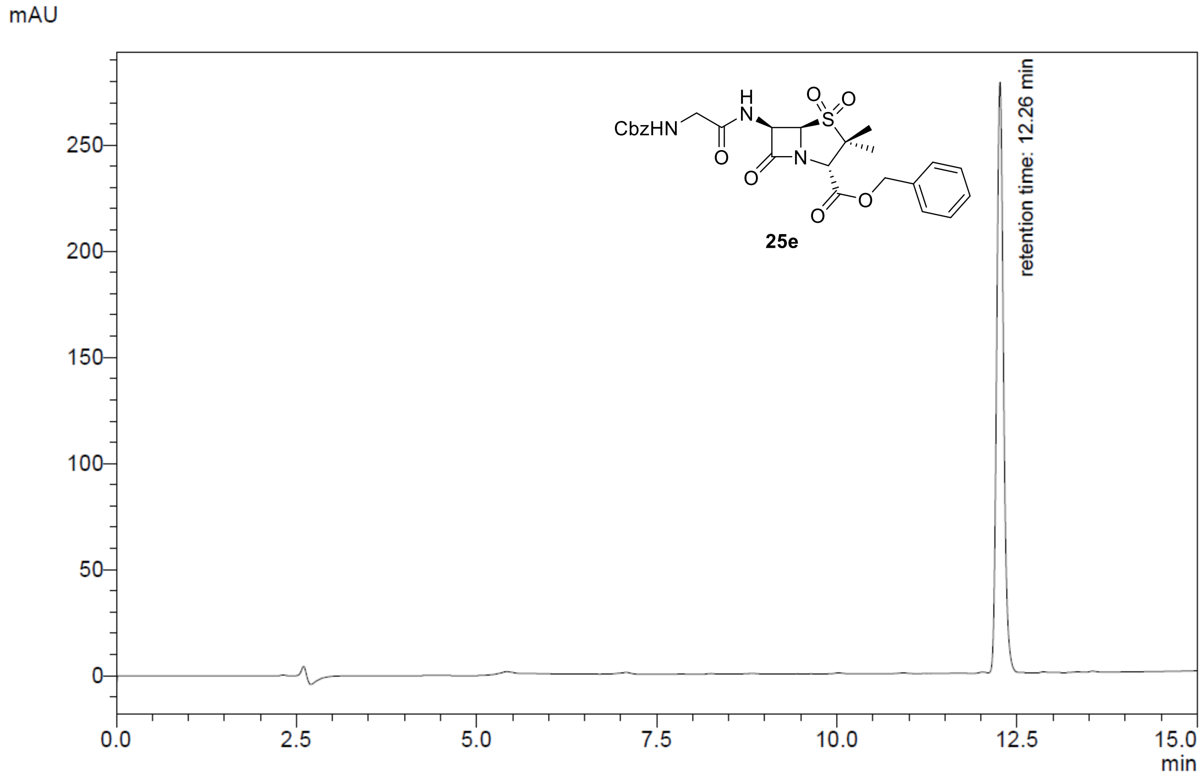

mAU
250
200
150
100
50
0
0.0
2.5
5.0
7.5
10.0
12.5
15.0
min
CbzHN
H
N
O
S
N
O
O
25e
retention time: 12.26 min

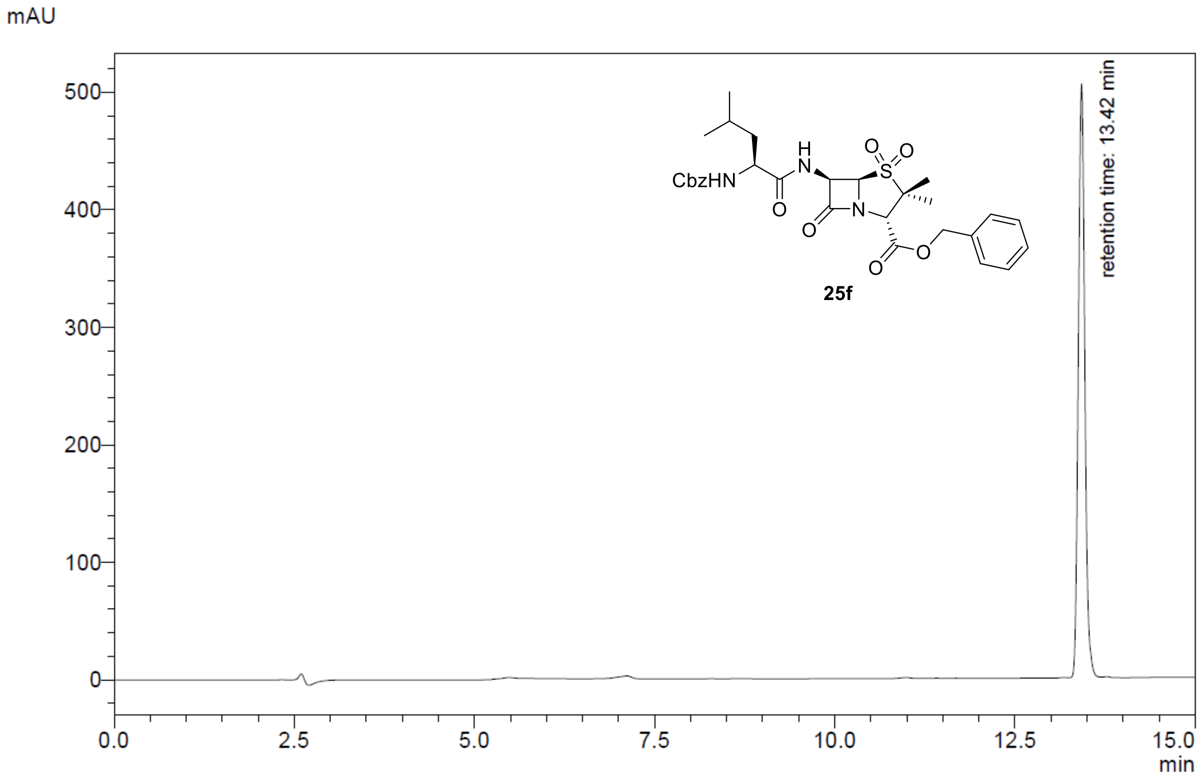
mAU
500
400
300
200
100
0
0.0
2.5
5.0
7.5
10.0
12.5
15.0
min
retention time: 13.42 min
CbzHN
H
N
O
S
O
O
N
O
O
O
25f

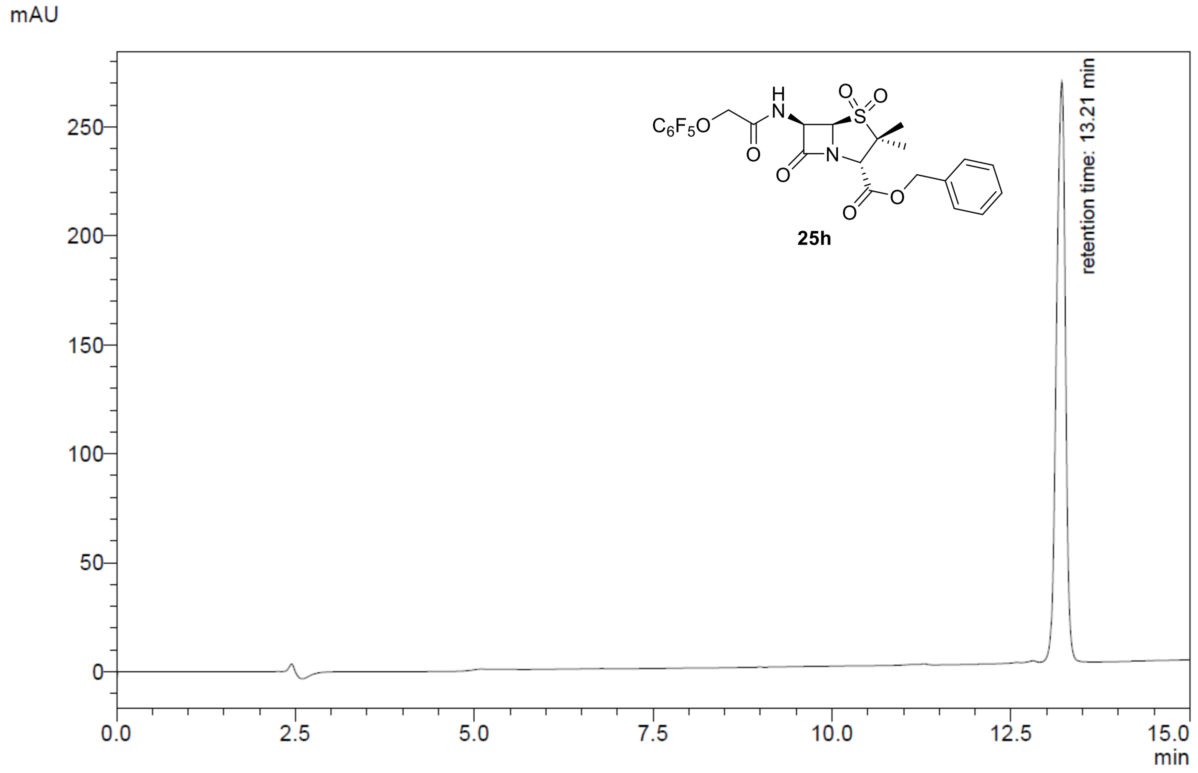
mAU
250
200
150
100
50
0
0.0
2.5
5.0
7.5
10.0
12.5
15.0
min
retention time: 13.21 min
C_6F_5O
H
N
O
S
O
O
N
O
O
O
25h

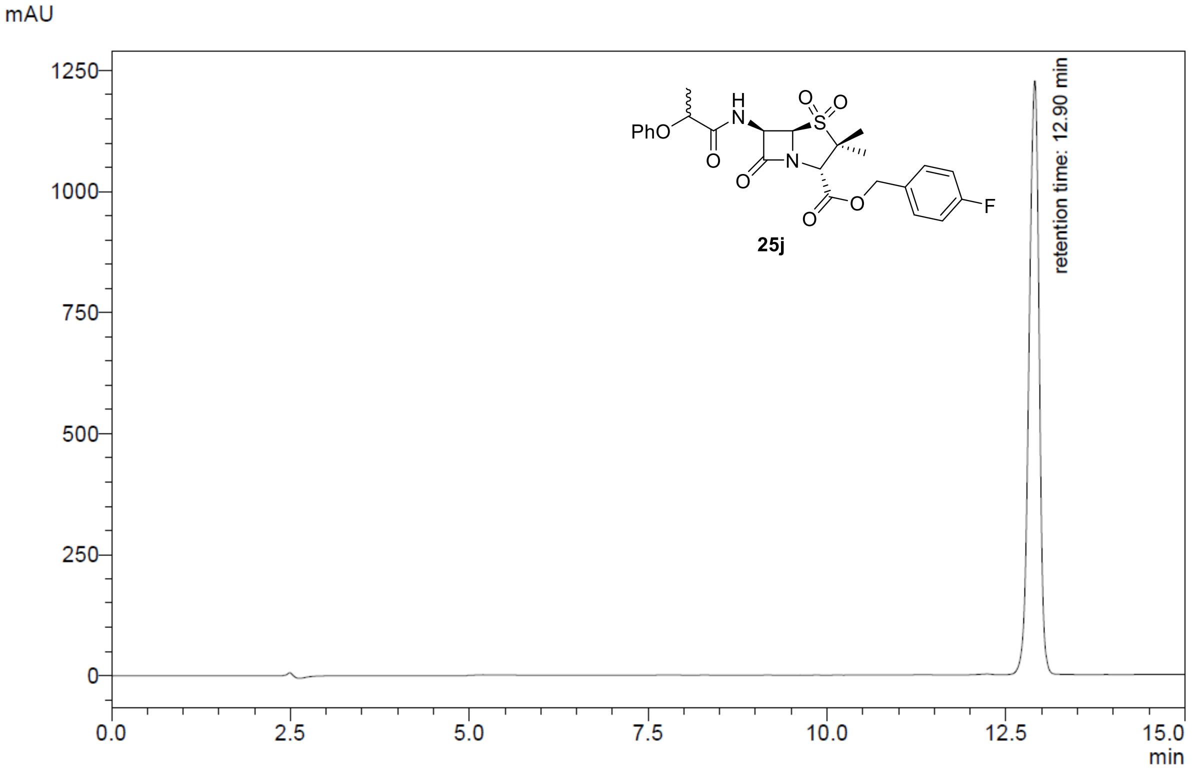
mAU
1250
1000
750
500
250
0
0.0
2.5
5.0
7.5
10.0
12.5
15.0
min
retention time: 12.90 min
PhO
H
N
O
O
S
O
N
O
O
O
F
25j

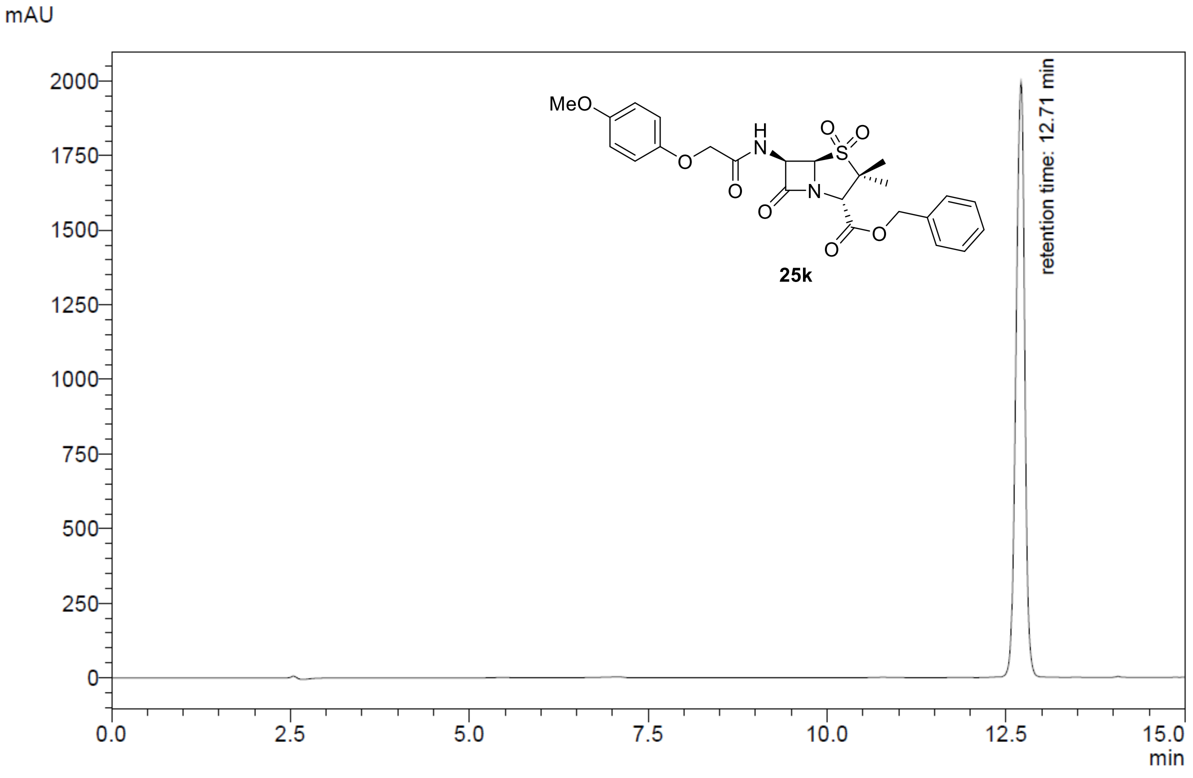
mAU
2000
1750
1500
1250
1000
750
500
250
0
0.0
2.5
5.0
7.5
10.0
12.5
15.0
min
retention time: 12.71 min
MeO
O
H
N
O
O
S
O
N
O
O
O
25k

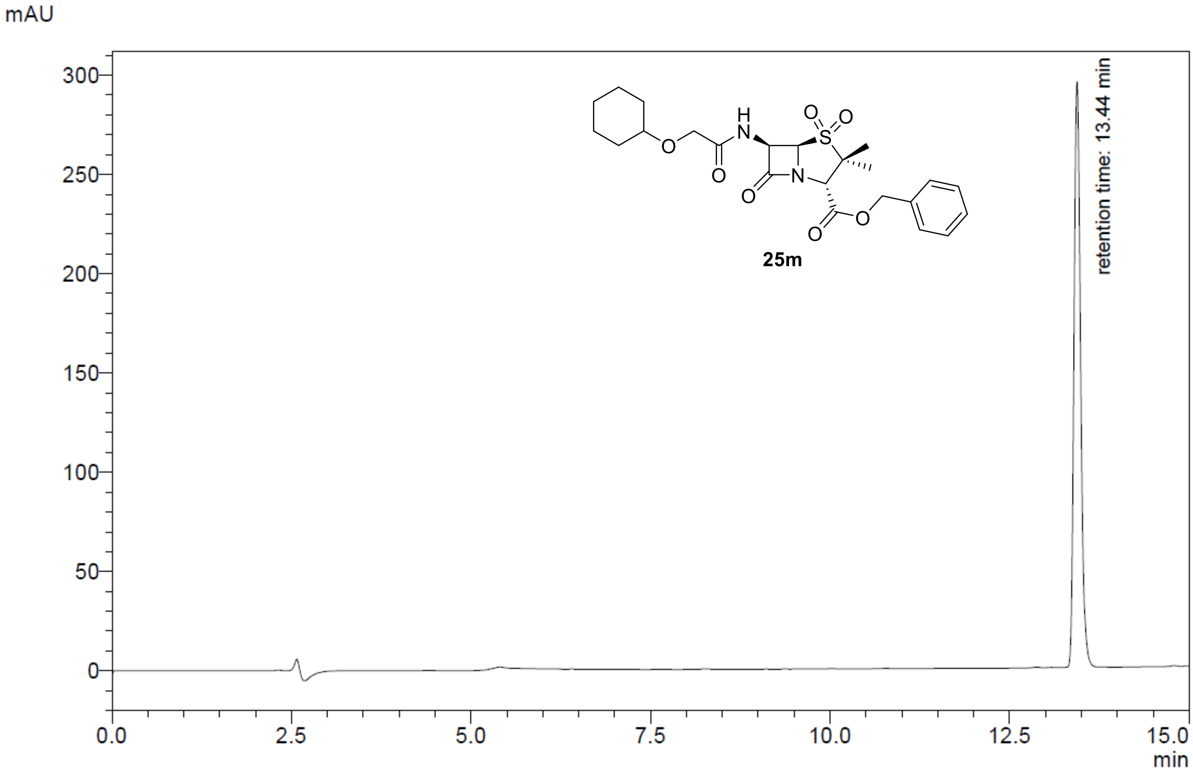
mAU
retention time: 13.44 min
25m
min

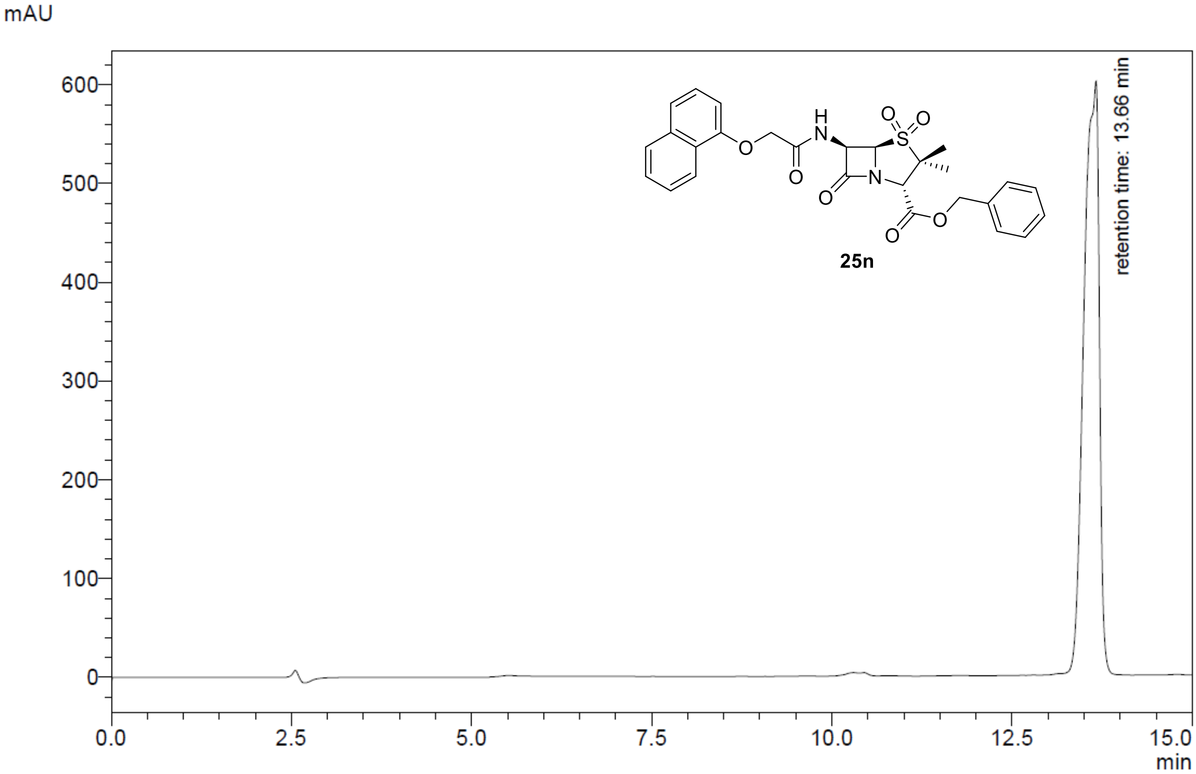
mAU
retention time: 13.66 min
25n
min
